# Major point and nonpoint sources of nutrient pollution to surface water have declined throughout the Chesapeake Bay watershed

**DOI:** 10.1088/2515-7620/ac5db6

**Published:** 2022-05-06

**Authors:** Robert D Sabo, Breck Sullivan, Cuiyin Wu, Emily Trentacoste, Qian Zhang, Gary W Shenk, Gopal Bhatt, Lewis C Linker

**Affiliations:** 1 U.S. Environmental Protection Agency, Office of Research and Development, Center for Public Health and Environmental Assessment, Washington, DC, United States of America; 2 U.S. Geological Survey, Chesapeake Research Consortium, Chesapeake Bay Program Office, Annapolis, MD, United States of America; 3 ERT, Inc., Laurel, MD, United States of America; 4 U.S. Environmental Protection Agency, Office of Research and Development, Immediate Office of the Assistant Administrator, Washington, DC, United States of America; 5 University of Maryland Center for Environmental Science, Chesapeake Bay Program Office, Annapolis, MD, United States of America; 6 U.S. Geological Survey, Chesapeake Bay Program Office, Annapolis, MD, United States of America; 7 Pennsylvania State University, Chesapeake Bay Program Office, Annapolis, MD, United States of America

**Keywords:** nitrogen, phosphorus, water, nutrients, agriculture, wastewater, atmospheric deposition

## Abstract

Understanding drivers of water quality in local watersheds is the first step for implementing targeted restoration practices. Nutrient inventories can inform water quality management decisions by identifying shifts in nitrogen (N) and phosphorus (P) balances over space and time while also keeping track of the likely urban and agricultural point and nonpoint sources of pollution. The Chesapeake Bay Program’s Chesapeake Assessment Scenario Tool (CAST) provides N and P balance data for counties throughout the Chesapeake Bay watershed, and these data were leveraged to create a detailed nutrient inventory for all the counties in the watershed from 1985–2019. This study focuses on three primary watershed nutrient balance components—agricultural surplus, atmospheric deposition, and point source loads—which are thought to be the leading anthropogenic drivers of nutrient loading trends across the watershed. All inputs, outputs, and derived metrics (n=53) like agricultural surplus and nutrient use efficiency, were subjected to short- and long-term trend analyses to discern how sources of pollution to surface water have changed over time. Across the watershed from 1985–2019, downward trends in atmospheric deposition were ubiquitous. Though there are varying effects, long-term declines in agricultural surplus were observed, likely because nutrients are being managed more efficiently. Multiple counties’ point source loads declined, primarily associated with upgrades at major cities that discharge treated wastewater directly to tidal waters. Despite all of these positive developments, recent increases in agricultural surpluses from 2009–2019 highlight that water quality gains may soon be reversed in many agricultural areas of the basin. Besides tracking progress and jurisdictional influence on pollution sources, the nutrient inventory can be used for retrospective water quality analysis to highlight drivers of past improvement/degradation of water quality trends and for decision makers to develop and track their near- and long-term watershed restoration strategies.

## Introduction

Excess nutrient loading to surface waters, attendant seasonal hypoxia, and the periodic occurrence of harmful algal blooms in estuaries and lakes across the globe compromise fisheries, diminish recreational opportunities, and endanger public health (Diaz *et al* 2001). Estuaries and lakes are often fed by large watersheds that can transcend multiple physiographic provinces and political boundaries, which makes the development, implementation, and coordination of restoration plans difficult (Billen *et al* 2013, Galloway *et al* 2003). Despite these challenges, signs of recovery are beginning to emerge in one of the largest and most productive estuaries in the world—the Chesapeake Bay. Over the 1985–2019 time period, declines in total nitrogen (TN) loads have been reported in nearly all of the major tributaries to the Chesapeake Bay (Chanat *et al* 2016, Moyer 2016). Positive ecological responses such as increases in submerged aquatic vegetation acreage, decreases in the summertime hypoxic extent, declines in chlorophyll-*a* concentrations, and improvements in water clarity have since been observed in the estuary (Lefcheck *et al* 2018, Murphy *et al* 2011, Zhang *et al* 2018). Understanding the major sources of nutrients and the drivers of water quality in the estuary and local surface waters is imperative to inform tailored restoration plans and efficiently maximize water quality benefits in the Chesapeake Bay and its watershed (Keisman *et al* 2015, Sabo *et al* 2019, Sabo *et al* 2021a).

Multiple models have attributed different potential drivers of N loading trends in the Chesapeake Bay watershed. These models have generally assessed three categories of likely drivers, changes in (1) total atmospheric N deposition, (2) point source loads, and (3) nonpoint source loads from agricultural, developed, and forested areas (Ator *et al* 2019, Eshleman and Sabo 2016, Fanelli *et al* 2019, Shenk and Linker 2013). Simple linear and non-linear regression models have related changes in atmospheric deposition to observed declines in nitrate loads in both forested and mixed land use basins throughout the Bay watershed. These kinetic N saturation models suggested that nitrogen oxides (NO_x_) emission controls and subsequent declines in atmospheric deposition primarily drove declines in nonpoint source loads for selected forested and agricultural watersheds draining to the Chesapeake Bay (Eshleman and Sabo 2016, Eshleman *et al* 2013). While the declines in atmospheric deposition inputs exceeded observed declines in instream N loads and atmospherically deposited nitrate has also been found to be potentially lost at a greater rate than other N inputs (Eshleman and Sabo 2016, Eshleman *et al* 2013, Sabo *et al* 2016), this study was limited by the fact that it did not incorporate potentially co-occurring shifts in point source loads or agricultural surplus (i.e., agricultural inputs minus crop removal) during the study period.

The statistical/process-based model SPAtially Referenced Regressions On Watershed (SPARROW) results, simultaneously calibrated for 1992, 2002, and 2012, suggested that reduction in point source loads explained >80% of the riverine water-quality improvement with the small remainder tied to improvements in nonpoint source loads (Ator *et al* 2019). However, this specific application of SPARROW did not account for changes in likely sources of nonpoint source pollution around the Bay, instead basing their attribution inference on the interpretation of year-specific land use constants which may miss important developments in agricultural management (Ator *et al* 2019). Furthermore, it is unclear how important declines in point source loads were in driving nutrient trends upstream of tidally influenced waters, where most Bay restoration activities need to be implemented (Zhang *et al* 2015a, 2015b) and where many dramatic declines in nutrient loads have occurred.

The more deterministic Chesapeake Bay Program’s Watershed Model (version 5.3) indicated that point source loads were indeed important for modeled improvements in water quality, but declines in nonpoint source loads from agricultural and forested areas actually explained slightly more improvement from 1985–2009 (Shenk and Linker 2013). It was unclear what drove the declines in the modeled nonpoint source pollution, though authors speculated this may be from a combination of decreased atmospheric deposition and increased nutrient use efficiency in agricultural production (Shenk and Linker 2013). Both the kinetic N saturation and SPARROW models were limited by the fact they did not explicitly incorporate many of the major sources of pollution, whereas the complexity of the Chesapeake Bay Program’s Watershed Model limited insight into the likely drivers of nonpoint source loads (besides atmospheric deposition resulting in a modeled decrease in forest N loads).

Similarly, varying attributions of drivers to observed long-term trends in phosphorus (P) loads to the Chesapeake Bay have also been reported. Recent SPARROW modeling work suggest that declines in P load, where observed, was essentially driven by decreased point source loads with essentially no change in non-point source loads (Ator *et al* 2019). Likewise, recent short-term trend analyses highlighted watersheds where orthophosphate concentrations and loads declined generally corresponded with declines in point source loads (Fanelli *et al* 2019). However, modeling results from Shenk and Linker (2013) suggested that the agricultural sector decreased TP loads by 21% from 1985 to 2009. Others have highlighted the potential effects of urbanization, allocation of crop acreage to the cropland reserve program, and legacy P on observed P loading trends (Fanelli *et al* 2019, Kleinman *et al* 2019). All in all, future analyses looking to explain past water quality changes for both N and P could benefit from a foundational dataset and analysis that systematically accounts for nutrient inputs and observed instream water quality outputs across space and time (e.g., Burns *et al* 2021, Keisman *et al* 2018).

Recent work has highlighted the power and utility of systematically accounting for nutrient inputs and outputs across space and time through nutrient inventories (Byrnes *et al* 2020, Sabo *et al* 2019, Sabo *et al* 2021b, Swaney *et al* 2018, Zhang *et al* 2016). These interpretable and largely empirically-based inventories demonstrate the source and magnitude of likely pollution sources within a study area and can also be used to develop management-relevant metrics that decision makers and stakeholders could potentially use to monitor progress more clearly within their local jurisdiction (Sabo *et al* 2021a). The Chesapeake Bay Program has developed comprehensive estimates of N and P fluxes at county and subbasin scales from 1985–2019 (CBP 2020). These estimates can be downloaded via the Chesapeake Assessment Scenario Tool (CAST). However, CAST was not specifically designed to succinctly communicate changes in N and P fluxes in and out of the terrestrial compartment of the watershed across space and time, thus it is unclear how inputs of point and nonpoint sources of pollutions have changed across the watershed. This limitation can be remedied by developing dedicated N and P inventory databases to effectively communicate shifts in the inputs and outputs of these nutrients through time, as done in other federal and academic research efforts (Sabo *et al* 2019, Sabo *et al* 2021b, Byrnes *et al* 2020, Swaney and Howarth 2019). In addition to tracking the magnitudes and trends of various nutrient sources in a watershed, ranging from agricultural and urban fertilizer application to point source loads (tables S1, S2 (available online at stacks.iop.org/ERC/4/045012/mmedia and in the attached supplemental files)), inventories can be used to derive key metrics that identify inefficiencies in the use or handling of N and P that are potentially driving water quality impairment in certain locales (Sabo *et al* 2021a). For example, identifying areas in the watershed that display low nutrient use efficiency and high agricultural surplus could lead to more effective prioritization of resources to decrease nutrient inputs or implement best management practices to attenuate excess nutrients remaining in farm fields after harvest.

In order to fill a critical research gap, we focus our efforts on describing the spatiotemporal changes in atmospheric deposition, point source loads, and agricultural surplus—the primary drivers of nutrient loading trends in the Chesapeake Bay identified by the models described above (Ator *et al* 2019, Eshleman and Sabo 2016, Eshleman *et al* 2013, Shenk and Linker 2013). However, the new county-level inventory includes additional urban and agricultural nutrient sources and is made available in the supplemental database to researchers and other users for future explorations (tables S1–S2). Overall, we expected coincident declines in atmospheric deposition, point source loads, and agricultural surplus, providing evidence that efforts to reduce point *and* nonpoint source pollution have all contributed to observed water quality improvements. The general and succinct communication of annual inputs and surpluses as well as long- and short-term trends in the fluxes and metrics will be key for informing future research initiatives and restoration strategies. This will be accomplished by detecting where the management of N and P is improving across the watershed and highlighting areas where further improvement can be achieved.

## Methods

### Overview

County level estimates of land use acreage and nutrient input/outputs were acquired from the CAST database. Spanning the 1985–2019 period, these data were lumped into general land use categories to succinctly communicate the magnitude of likely pollution sources as well as shifts in the handling and management of N and P in natural, urban, and agricultural domains (described further below). Besides maps illustrating the magnitude of various variables for the years 1985 and 2019, all input, output, and derived metrics were subjected to long and short-term trend analysis to succinctly illustrate trajectories of likely point and non-point sources of pollution across the Chesapeake Bay (please see figures S1–S108), but this analysis primarily focuses on spatiotemporal patterns of point source loads, atmospheric deposition, and agricultural surplus. Other components of the county level nutrient inventory, like urban fertilizer or biosolid application, can be subjected to further exploration and interpretationusing the attached supplemental database.

### Development of the county-level nutrient inventory

Land use acreage (developed, natural, and agricultural), population, septic, point source loading, atmospheric deposition, crop removal with pasture removal, crop removal without pasture removal, crop fixation, agricultural fertilizer, urban fertilizer, biosolids, legacy P in soils, and livestock/poultry manure data were downloaded using the 1) Loads, 2) Atmospheric Deposition, and 3) Nutrients Available Reports feature on the Chesapeake Bay Program’s CAST website (https://cast.chesapeakebay.net/; Version CAST-2019)—see Tables S1–S2 and the supplemental database.

The databases covering the period 1985–2019 were further simplified by summing the mass of the respective N or P flux onto specific land uses into general land use categories (e.g., all agricultural land use classes into agricultural land use). Nutrient allocations to specific land uses in CAST are used to allow flexibility in estimating loads from various land uses and tracking the effects of various best management practices (BMPs) (e.g., cover crops). However, the sheer volume of information inhibits general and succinct communication of changes in N and P cycling of a county and/or subbasin to a wider audience, thus limiting its interpretability and impact (Keisman *et al* 2015). The decision to aggregate to larger land use categories was further validated through consultations with various workgroups of the Chesapeake Bay Program and positive feedback received from regional conferences and meetings with stakeholders.

Estimates of fluxes included in this nutrient inventory are generally empirical to semi-empirical due to the fact the foundational data that drive these estimates are largely observed. For example, the fertilizer application rates are constrained by reported county/state sales data and Census of Agricultural chemical expenditure data. Drawing from the Census of Agriculture, livestock and poultry associated fluxes are based on reported animal populations within a county, and county crop removal rates are based on farmer-reported crop yields and reported pasture acreage. Likewise, a large proportion of point source loads are based on reported effluent volumes and nutrient concentrations with only a small proportion of the total estimated loads relying on assumed nutrient concentrations based on facility type and treatment level. Even the hybrid modeling of atmospheric N deposition is constrained by empirical observations, with the wet deposition component being determined by regression models and the more deterministic modeling of dry deposition being partially constrained to observed dry deposition rates (Burns *et al* 2021, Linker *et al* 2013). The traceable empirical foundations of these estimates are a large asset in confidently communicating shifts in the handling and management of N and P throughout the Chesapeake Bay watershed. Detailed documentation of methodologies can be found on the CAST website: (https://cast.chesapeakebay.net/ - table S1–S2).

For this work, we focused our analysis on evaluating the likely drivers of surface water quality trends—point source loads, atmospheric deposition, and agricultural surplus. However, we wish to emphasize dozens of other input, output, and derived variables are available to be further evaluated in the supplemental database. We calculated total point source loads by summing municipal and industrial wastewater treatment loads, combined sewage overflows, and septic. Total atmospheric N deposition was the sum of wet and dry depositions, with total atmospheric oxidized and reduced N on land, and oxidized, reduced, and organic N deposition on water. Agricultural N surplus is the difference between the sum of agricultural N inputs (i.e., legume N fixation + poultry and livestock manure N applied + atmospheric deposition on agricultural land + agricultural N fertilizer) and crop N removal (excluding pasture removal). Agricultural P surplus is similar but lacks atmospheric deposition and fixation input terms. In addition to the agricultural surplus, we also calculated agricultural N and P use efficiency (NUE and PUE), which is the ratio of crop removal to agricultural inputs described above (Sabo *et al* 2021a, Swaney *et al* 2018, Zhang *et al* 2015a, 2015b). We also calculated agricultural surplus and nutrient use efficiency with estimates of pasture removal and direct deposition of manure onto pasture if the user wishes to track the potential impacts of these pasture fluxes on the mass balance in the supplemental database. Overall, the agricultural surplus represents the amount of N and P that is lost to the environment in a given year in runoff, volatilization, taken up by vegetative BMPs, denitrified (for N), or added to soil storage. This surplus is the ultimate source of agricultural nonpoint source pollution within a watershed. The annual agricultural surplus may have near-immediate impacts on nutrient loads, or it may be stored and released later in the catchment (i.e., a legacy effect) (Chang *et al* 2021, Van Meter *et al* 2017). Regardless of the timing of water quality impacts, limiting the amount of surplus of N and P is imperative to reducing surface water N and P loads and generally the most cost-effective where application rates can be feasibly adjusted (Dupas *et al* 2020, Sabo *et al* 2021a, Sinha and Michalak 2016). It should be noted, the magnitude and short/long-term trend maps described below have been illustrated for all components of the nutrient inventory (figures S1–S108). The complete county level N and P inventories, themselves ranging from 1985 to 2019, is also attached in the supplemental database.

### Statistical analyses

For the 1985–2019 and 2009–2019 periods, individual county level trends for all annual nutrient fluxes and derived variables were evaluated in R using the non-parametric Mann-Kendall trend test and Senn-slope estimator to provide a long- and short-term summary of trends (R Core Team 2021). All fluxes were normalized by the county area in order to facilitate comparison across jurisdictions. County-level, watershed-wide trend maps of all variables in this nutrient inventory are available for download in the supplemental material. Additionally, watershed-wide maps were made to visually represent the individual nutrient input for the first year of the nutrient inventory and one for the last year (i.e., 1985 and 2019) as well as the estimated trend and statistical significances. For this article, we primarily focus our presentation on long-term (i.e., 1985–2019) trends of the variables described. Short-term (i.e., 2009–2019) trend results for the variables of interest are available in the supplemental database. In addition, all other short-term and long-term trend maps for other mass balance variables and various derived metric are available in the supplemental material (figures S1–S108).

It should be noted these reports are available at the county-scale, thus some of the counties that are situated along the watershed boundary will have fluxes and surpluses falling outside of the drainage area. As such, summed county input, removal, and surplus values at the scale of a state or the Chesapeake Bay watershed will be overestimated, though inference of trends will not be impacted since the county scale data, if disaggregated, is simply apportioned to the county area within the watershed. Furthermore, down-scaling does not necessarily provide any new information as trends and the magnitude of inputs, output, and other derived metrics simply track with the county level data. Geographic information system analyses, of various sophistication, can be used to apportion county-level estimates to the portion that falls within the watershed though these efforts are not regularly done or available to the public (e.g., (Hamlin *et al* 2020, Hong *et al* 2013), whereas this county level database is updated in regular intervals. Recently, a database that disaggregates CAST mass balance into ~2.1 km^2^ subbasins has been released (Devereux *et al* 2022), and this work can guide the use of the down-scaled mass balance data.

## Results and discussion

### Statewide sources of nutrient pollution largely declined

**Figure 1.**
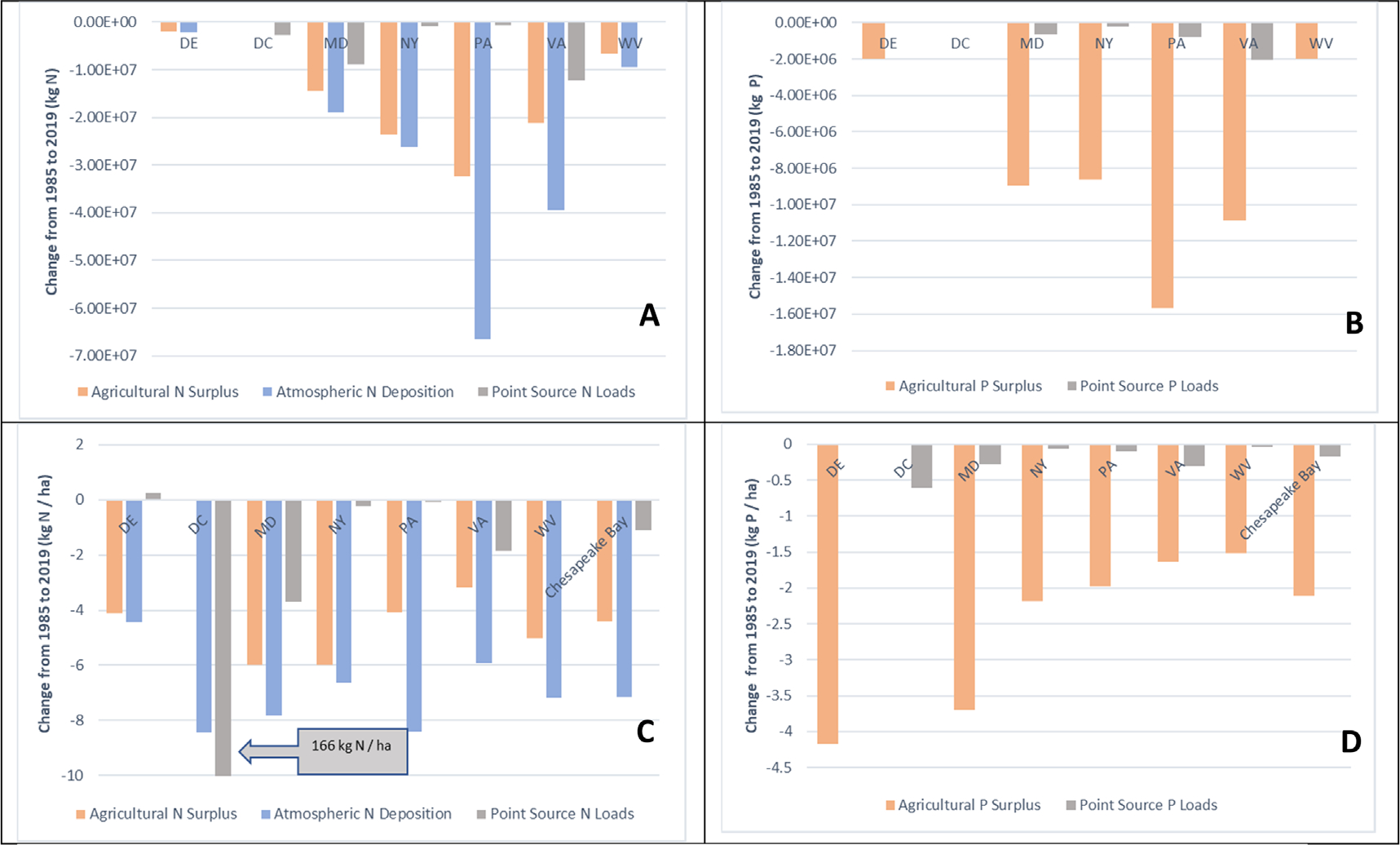
Long-term shifts in atmospheric N deposition, agricultural surplus of N and P, and point source loads of N and P on a pure mass basis and normalized by state area (in units of kg for panels A/B or kg ha^−1^ for panels C/D for counties within a state occurring, falling completely, or partly within the Chesapeake Bay watershed and Washington, DC). Values below zero indicated a decline over time. Please note, bars not visible are reflective of small changes in a variable within a state relative to others.

Across the Chesapeake Bay watershed, major sources of point and nonpoint N and P pollution to surface water have declined from 1985–2019 (figure 1). On a mass basis (kg), Pennsylvania had the largest declines in atmospheric N deposition and agricultural N and P surplus, followed by New York, Virginia and Maryland (figures 1(A)–(B)). On an areal intensity basis (kg ha^−1^), however, declines in agricultural N were highest in New York and Maryland. For P surplus, declines were greatest in Maryland and Delaware (figures 1(C)–(D)). Maryland, Pennsylvania, and Washington, DC have benefited most from federal efforts to decrease NO_x_ emissions (Lloret and Valiela 2016), generally showing decreased N inputs from atmospheric deposition of roughly 8 kg N ha^−1^ since 1985 with other states close behind (figure 1(C)). Declines in atmospheric deposition have likely decreased instream delivery of nitrate (NO_3_-N) loads in predominantly forested and mixed land use basins (Sabo *et al* 2016, Bostic *et al* 2021) and have potentially decreased the magnitude of N inputs and/or agricultural surpluses on agricultural and urban land as well (Eshleman and Sabo 2016, Eshleman *et al* 2013, Sinha and Michalak 2016).

Watershed-wide, agricultural N and P surplus declined by roughly 4 and 2 kg ha^−1^, respectively, with correspondingly large declines in atmospheric deposition (7 kgN ha^−1^ or 163 million kilograms, figure 1), providing evidence that changes in nonpoint source loads from forest and agricultural areas of the watershed may have declined in response to changes in surplus and deposition, potentially contributing to observed water quality trends in the Chesapeake Bay.

The largest declines in point source N and P loads on a mass basis occurred in Virginia (figures 1(A)–(B)). However, on an areal intensity basis, Maryland decreased point source N loads greatest among the states (figure 1(C)). While maintaining wastewater treatment services to many bourgeoning Virginia and Maryland suburbs in addition to its own residents (~1.6 million people, not including workers that live outside these service areas), Washington, DC’s efforts to upgrade treatment technologies and eliminate combined sewer overflows have resulted in dramatic declines in point source loading into the tidal portions of the Potomac River (CBP, 2020), the second largest freshwater tributary to Chesapeake Bay (166 kg N ha^−1^, figure 1(C)). Overall these declines in point source loads occurred even with an increase in the human population by ~5 million residents in the Chesapeake Bay watershed (CBP, 2020; Supplemental Database).

### Long-term improvements in point and nonpoint sources of pollution driven by specific counties

**Figure 2.**
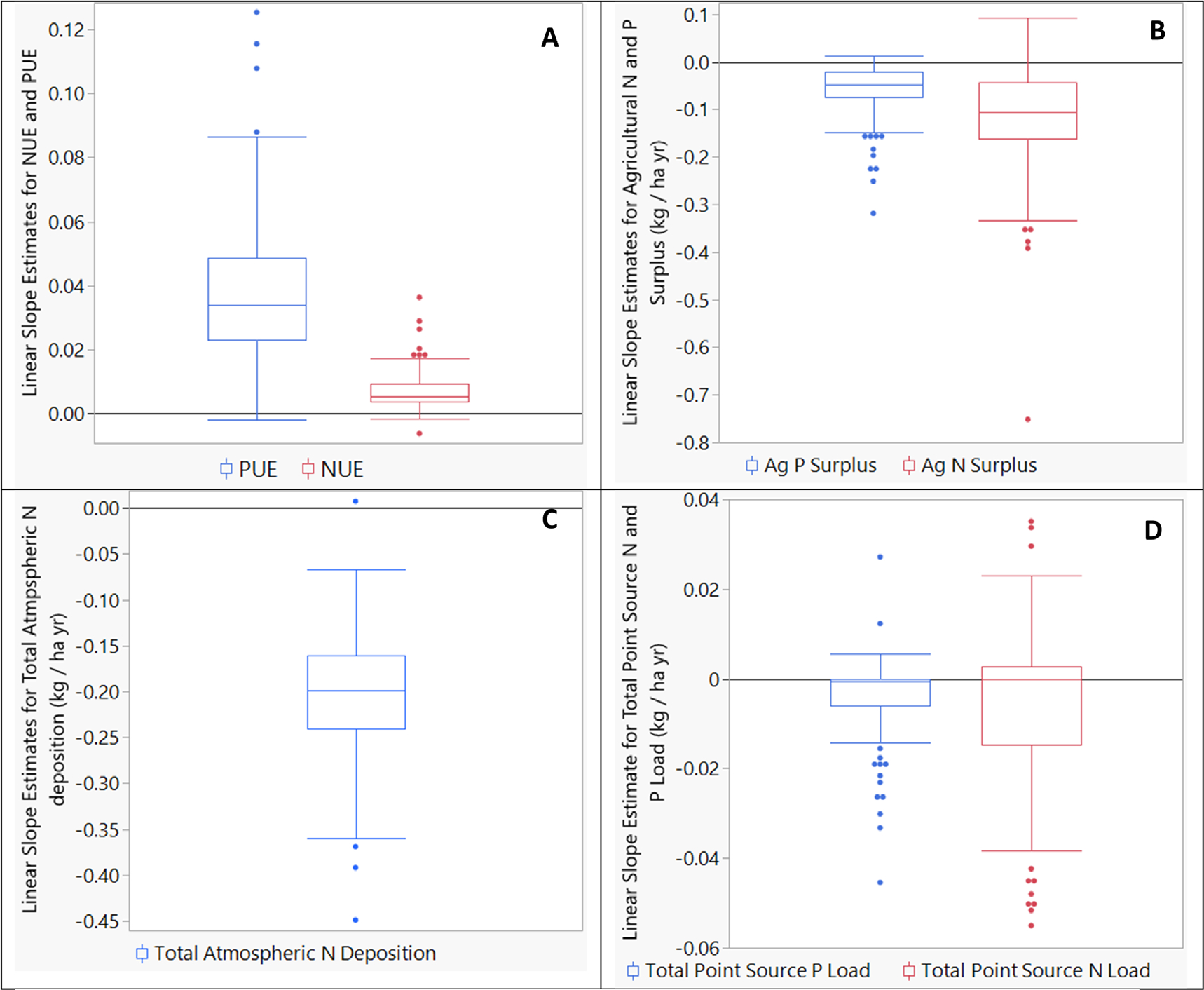
Boxplots of county-level non-parametric linear Sen slope estimates for N and P use efficiency (NUE and PUE, respectively), agricultural N and P surplus, total atmospheric N deposition, and total point source N and P loads for the 1985–2019 period.

**Figure 3.**
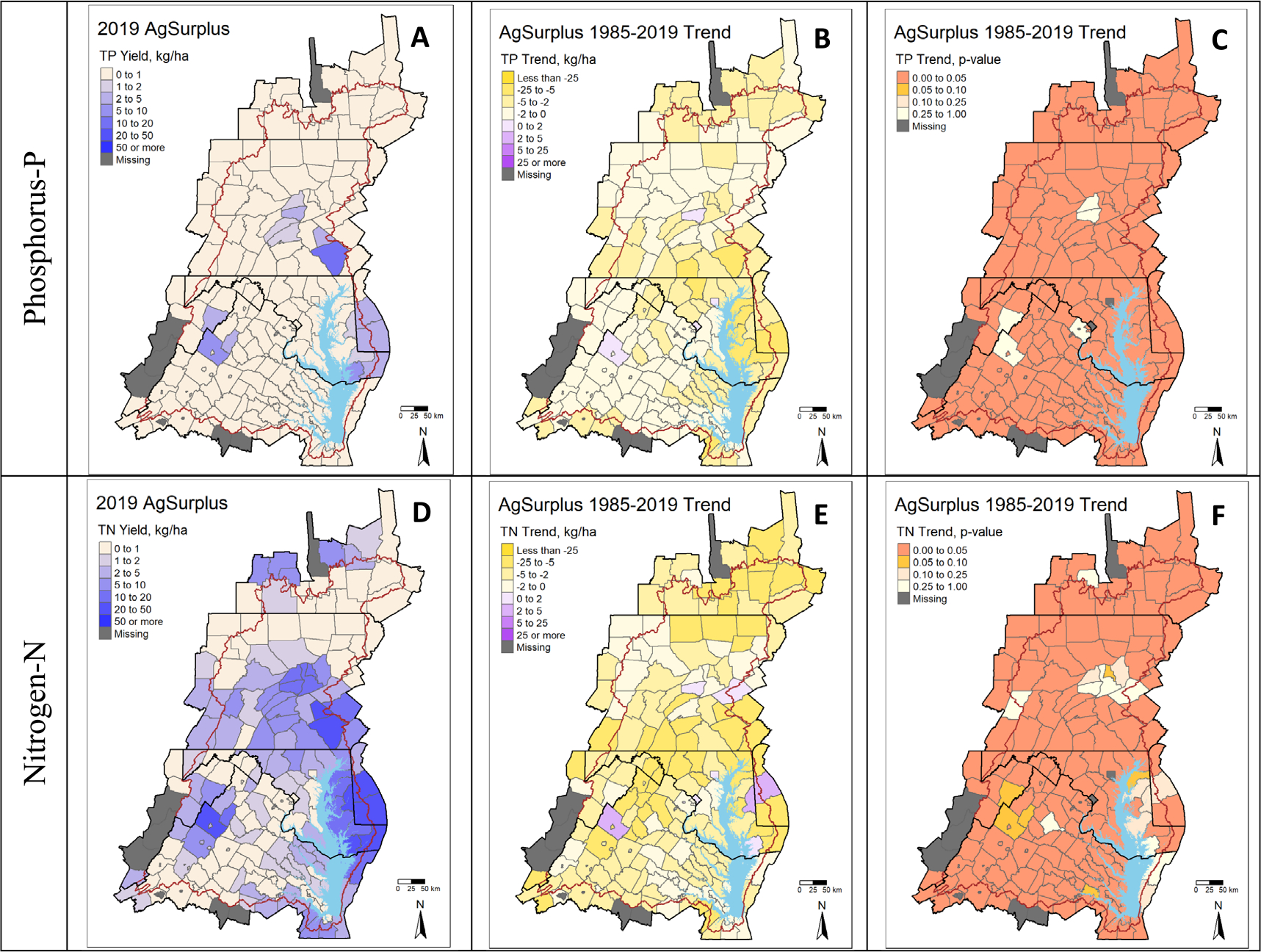
2019 agricultural surplus, the estimated Sen linear slope change in agricultural surplus from 1985–2019, and the significance of trend results by county.

Most counties in the Chesapeake Bay watershed experienced declines in atmospheric deposition and agricultural surplus, and these declines corresponded with increases in nutrient use efficiency (figures 2(A)–(C), 3(B), (E), figures S101–108). Importantly, declines in agricultural N and P surplus were not driven by declines in livestock or crop production as manure inputs and crop removal values, watershed-wide, have largely increased over the period of record. For N, the increase in manure application has been largely offset by declines in fertilizer use (Shenk and Linker 2013). This observation combined with increased crop removal and decreased atmospheric deposition has led to promising increases in NUE in most of the counties. In the few counties where efficiency declined (figure 2(A), figures S101–108), agricultural N surpluses increased (purple counties, figure 3(E)). Increases in NUE in the Chesapeake Bay watershed counties are consistent with long-term and short-term nation-wide increases in the United States (Zhang *et al* 2021, Sabo *et al* 2019, Zhang *et al* 2015a, 2015b).

Gains in P use efficiency (PUE) in agricultural production far exceeded that of N (figure 2(A)), resulting in the surprisingly approximate reductions in agricultural N and P surplus at the state and watershed levels despite the large differences in plant nutritional requirements and much lower P inputs. These gains may be tied to the fact that P fertilizer use peaked in the 1970s and 1980s in the United States, and many areas of the country, including the Chesapeake Bay watershed, are removing more P with crop harvest and pasture removal than is applied in a year (Figure S109, (Fixen *et al* 2012)). This ‘mining’ of legacy P in soils is imperative to decreasing future P export to surface water (Chen *et al* 2018). Indeed, legacy P pools in the Chesapeake Bay watershed have correspondingly declined (Soil P, figures S85–88).

The declines in agricultural N and P surpluses have likely already decreased or will eventually decrease agricultural nonpoint source loads to surface water, which is the largest source of nutrient pollution to the Chesapeake Bay. Future work could elucidate the role of changing agricultural surpluses on observed water quality trends. However, a simple comparison of declines in agricultural surpluses with the large relative declines (~10%–40%) in N and P export in many agricultural catchments provides compelling evidence for the role of improved use and management of N and P in agriculture in restoring water quality in the Chesapeake Bay (Moyer 2016). Lacking major declines in point source loads, the Monocacy River and Antietam Creek in central Maryland and the Conestoga River in southeastern Pennsylvania are good examples (CBP, 2020).

The coincidence of large declines in nutrient export in predominantly agricultural catchments with decreases in agricultural surplus certainly needs to be further explored as nutrient management plans, if properly implemented, can be the most cost-efficient of BMPs to implement, providing direct incentives and benefits to both farmers and restoration efforts (CBP 2020, Sabo *et al* 2021a). It should be noted that in CAST, agricultural nutrient input data are influenced by the reporting of the adoption of nutrient management plans. As such, states that have reported and credited nutrient management BMPs are more likely to display improving surpluses and nutrient use efficiency through time. Maryland in the early 2000s passed the Nutrient Management Law, which required all farms grossing over $2,500 to have certified nutrient management plans developed and approved by the Maryland Department of Agriculture (Dotterer 2017). Likewise, the passage of the Pennsylvania Nutrient Management Act in 1993, may have contributed to observed improvements in nutrient surpluses in major agricultural counties in the lower Susquehanna and upper Potomac reaches in southern Pennsylvania. All of these agricultural areas have experienced large improvements in TN export over the period of 1985–2019 (Moyer 2016). The varying adoption of nutrient management plans combined with increasing crop yields resulted in different surplus and nutrient use efficiency trajectories across jurisdictions, but this inventory highlights improved agricultural surpluses as a likely source of water quality improvement upstream of tidally influenced waters.

**Figure 4.**
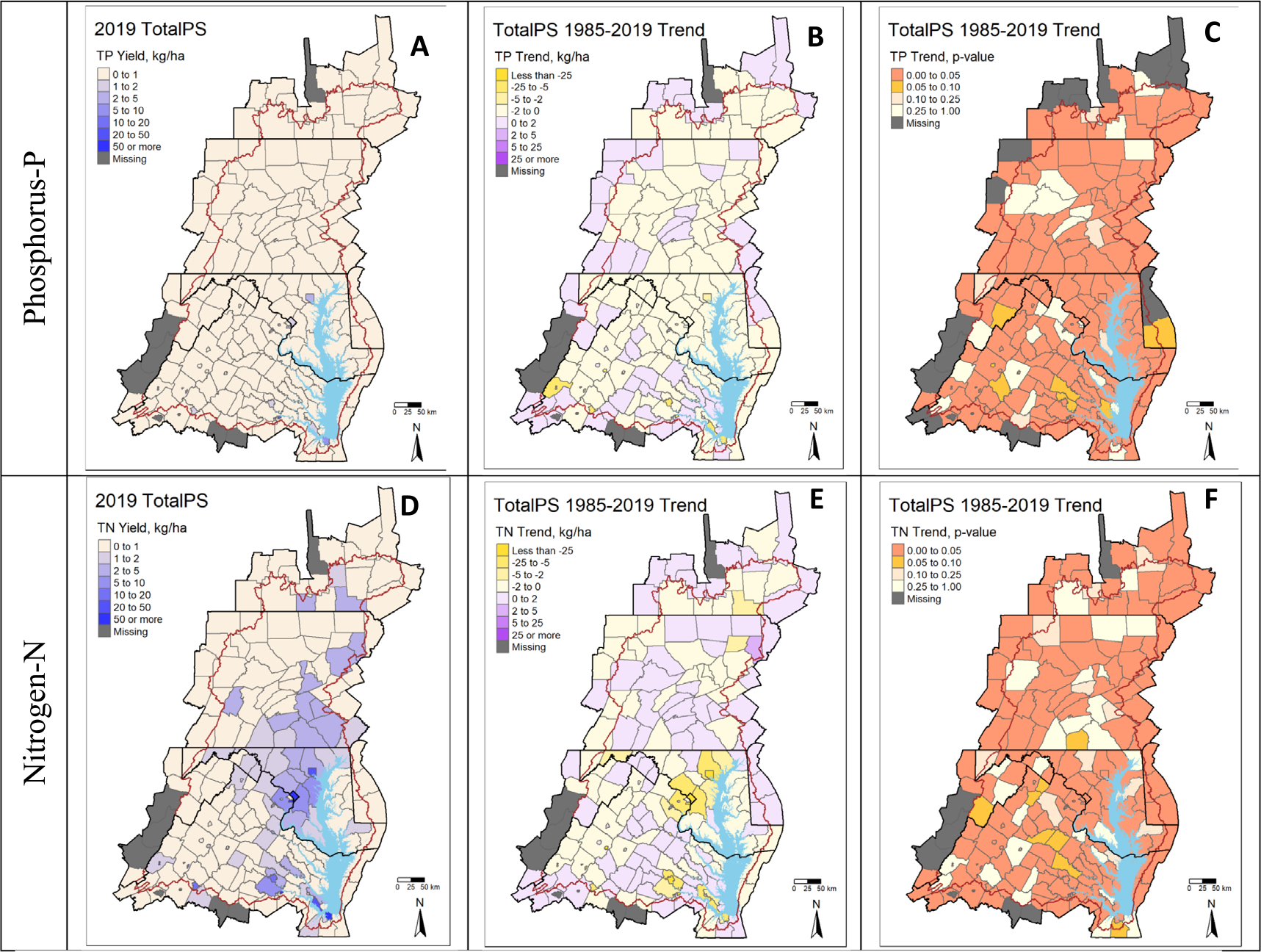
2019 point source loads, estimated Sen slope change in point source loads from 1985–2019, and the significance of trend results by county.

In contrast to the widespread declines in atmospheric N deposition and agricultural surplus, a much smaller fraction of counties in the Chesapeake Bay watershed demonstrated declines in point source loads (figure 2(D), figures 4(B), (E)); though these counties ultimately drove state- and watershed-level declines in this important pollution source despite population and economic growth over the 1985–2019 period (figure 1). This observation is tied to the fact that large, urban areas are likely to have the largest wastewater treatment plants. Declines in point source loads occurred in major urban areas like Hampton Roads, VA, Richmond, VA, Washington, D.C., and Baltimore, MD, and the declines were due to upgrading wastewater treatment technologies and decreasing the occurrence of combined sewage overflows (CBP, 2020). However, large declines also occurred in small cities like Waynesboro and Hopewell, VA, and these decreases are tied to both a decline in industrial discharges and improvement in municipal wastewater treatment (CBP 2020). Unlike agricultural surplus and atmospheric deposition, shifts in point source loads have near immediate impacts on water quality as the nutrients are directly discharged into the river, stream, or tidal Bay (Chang *et al* 2021). In this regard, municipal wastewater treatment plants with the largest nutrient declines (e.g., those in Richmond, VA, Washington, D.C., and Baltimore, MD) discharge downstream of the fall line into or near tidal waters of the Chesapeake Bay and have likely had a more immediate postive influence on water quality in the estuary (e.g., figure 4(E), Murphy *et al* 2021). However, in many areas of the watershed upstream of the fall line (i.e., upstream of tidally influenced areas), changes in point source loads are likely not the primary driver for water quality trends, illustrated by little to no changes in observed trends (figure 4). Recent semi-empirical to more processed based modeling work is consistent with this speculation in that changes in non-point source loads explained the majority of improvement in many of the major river basins in the Chesapeake Bay (Sabo 2018, Chang *et al* 2021).

While future work can more directly link observed nutrient water quality loading trends to these nutrient input inventories, our results highlight that agricultural nonpoint sources of pollution and atmospheric deposition across the watershed have indeed declined and lend clear evidence to the likely important contribution of decreasing nonpoint source pollution to water quality improvement across much of the Chesapeake Bay watershed (Chang *et al* 2021, Eshleman and Sabo 2016, Shenk and Linker 2013). Larger declines in point sources were also evident, and probably had bigger impact on water quality trends downstream of the fall line in tidal waters (Ator *et al* 2019). These observations highlight that efforts to decrease NO_x_ emissions, increase agricultural nutrient use efficiency, and investments to upgrade municipal and industrial wastewater treatment plants (as well as industrial decline) likely made or have the potential to make positive impacts on water quality trends (Ator *et al* 2019, Eshleman and Sabo 2016, Shenk and Linker 2013). This database will allow future research to link changes in the nutrient inventories to observed water quality changes, and may be useful for determining the responsiveness of watersheds to management actions and legacy effects (Sabo *et al* 2021a, Van Meter *et al* 2017). In addition to future analyses linking changes in water quality to the nutrient inventory and land use trends (available in the supplemental database), future research could also consider the influence of climatic variability and BMPs (Chanat and Yang 2018, Fanelli *et al* 2019) and the potential role of deacidification in forests (Lawrence *et al* 2020, Sabo *et al* 2020).

### Declining nutrient use efficiency in the 2010s and challenges ahead

Progress in decreasing nutrient pollution is not guaranteed. Continued urbanization, growing population, and continued evolution of agricultural practices are major challenges that will make it increasingly difficult to manage the various sources of nutrient pollution, especially under conditions of a changing climate (Ator *et al* 2020). Outside of the fluxes and metrics reported in this paper, users can explore other county-level nutrient inputs (e.g., urban fertilizer and biosolids) and land use terms (urban, forest, and developed) to appreciate how the N and P cycle are evolving through time across the Bay watershed.

**Figure 5.**
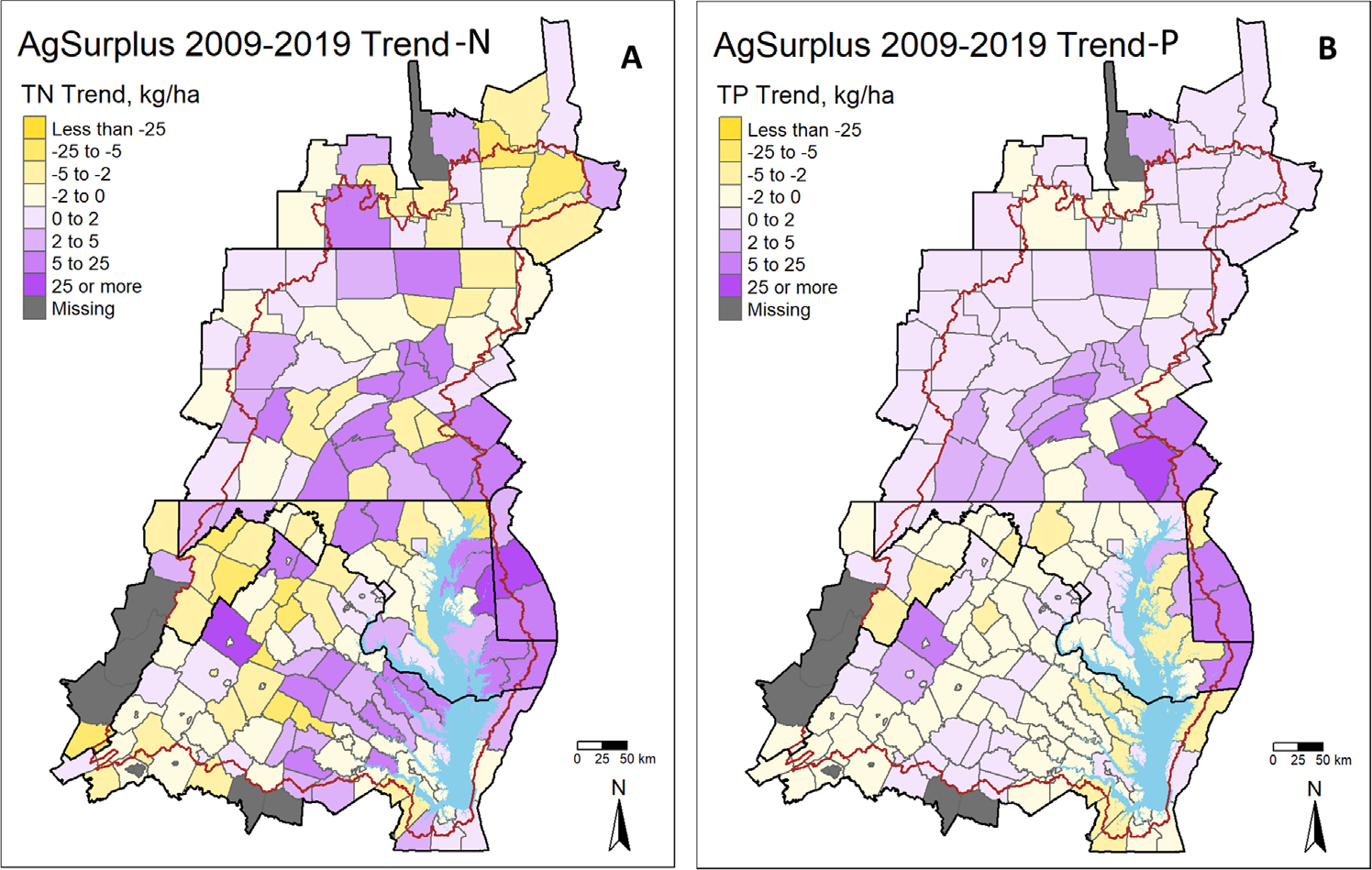
Estimated Sen slope change in agricultural N and P surplus from 2009–2019.

Efforts to further increase nutrient use efficiency and decrease agricultural surpluses will be key for attaining water quality goals under these changing conditions. Some available empirical evidence suggests that the instream response of N loads to shifts in nutrient balances ranges from 1–15 years (Chang *et al* 2021, Dupas *et al* 2020, Hong *et al* 2012, Sinha and Michalak 2016), thus many of the promising long-term declines in N export in agriculturally intensive areas of the Chesapeake Bay may continue to decline even without a further decline in surpluses over the next decade or more. However, a troubling trend for agricultural N and P surplus has emerged in the past decade. Agricultural N and P balances have increased in the eastern and northern parts of the watersheds, most consistently in Pennsylvania (figure 5, see all other inventory short-term trends in the supplemental material). Increases in agricultural surpluses are likely to put further pressure on existing BMPs designed to attenuate excess N and P left in the fields after harvest and may ultimately counteract many of the water quality gains observed over the past two decades. Efforts to maintain and restore long-term trajectories of increasing agricultural nutrient use efficiency and declining surplus in these counties will be key to Chesapeake Bay restoration.

## Conclusion

Overall, the county-level nutrient inventories developed for the Chesapeake Bay watershed provide compelling evidence of spatiotemporal changes in nutrient inputs, balances, and point source loads that coincide with sometimes large relative changes in nutrient loads. Long-term declines in atmospheric deposition were observed throughout the Chesapeake Bay watershed, whereas major declines in point source loads largely occurred in tidal portions of the watershed. Unexpectedly, long-term improvements in nutrient use efficiency with corresponding declines in agricultural surplus were observed. The declines in agricultural surplus were substantial Bay-wide, and potentially explain a large fraction of observed improvements in long-term N and P loading trends in the watershed. Further research efforts should consider explicitly exploring the long and short- term impacts of declining agricultural surpluses on nutrient loads. Regardless, the report of improved agricultural nutrient management and its further promotion will help limit a major non-point source of pollution to waterways. Moving beyond identifying major drivers of water quality change in the Chesapeake Bay, users of this inventory can reference the >100 short and long-term trends of other fluxes and derived metrics that may be more locally relevant for them in achieving their local water quality goals (e.g., urban fertilizer, figures S1–S108), and use the database to develop communication products to local stakeholders to highlight success stories and ongoing/emerging challenges (Supplemental Database). These inventories provide insights into drivers of water quality across the landscape across natural, urban, and agricultural domains, lay the necessary foundation to explore links between water quality and management actions, and inform watershed management decisions moving forward.

## Supplementary Material

s1

s2

## Data Availability

All data that support the findings of this study are included within the article (and any supplementary files).
