# Supplementary material for: Major point and nonpoint sources of nutrient pollution to surface water have declined throughout the Chesapeake Bay watershed
Source: Environ Res Commun. Author manuscript; Available in PMC 2023 May 6. (PMC10116850; doi:10.1088/2515-7620/ac5db6)
Supplement: s1 [file NIHMS1875283-supplement-s1.docx]

Supplementary Information: Major point and nonpoint sources of nutrient pollution to surface water have declined throughout the Chesapeake Bay watershed

# Robert D. Sabo^1*^, Breck Sullivan^2^, Cuiyin Wu^3^, Emily Trentacoste^4^, Qian Zhang^5^, Gary W. Shenk^6^, Gopal Bhatt^7^, and Lewis C. Linker^4^

^1^ U.S. Environmental Protection Agency, Office of Research and Development, Center for Public Health and Environmental Assessment, Washington, DC

^2^ Chesapeake Research Consortium, Chesapeake Bay Program Office, Annapolis, MD

^3^ ERT, Inc., Laurel, MD

^4^ U.S. Environmental Protection Agency, Office of Research and Development, Immediate Office of the Assistant Administrator, Washington, DC

^5^ University of Maryland Center for Environmental Science, Chesapeake Bay program Office, Annapolis, MD

^6^ U.S. Geological Survey, Chesapeake Bay Program Office, Annapolis, MD

^7^ Pennsylvania State University, Chesapeake Bay program Office, Annapolis, MD

^*^Corresponding author. Address USEPA Headquarters, 1200 Penn Ave NW, Mailcode 8623-P, Washington DC 20460. [Sabo.robert@epa.gov](mailto:Sabo.robert@epa.gov)

**Supplementary Tables and Figures:**

1. Pages 2-3—Tables S1-S2
2. Pages 4-112—Figures S1-S109
3. County Level Nitrogen and Phosphorus Database Separately Attached in the Supplementary Data Section (Supplemental_data_N_P.xlsx)

Table S1: List of nitrogen fluxes and other derived variables as well as the data source and the name of the section presenting detailed methodologies at <https://cast.chesapeakebay.net/Documentation/ModelDocumentation>. This table is also reference for column headers in the county level supplemental database (1985-2019).

| Variable Name | Abbreviation | Original Units | Unit in Supplemental Database | Source | Access to Description of Methodology |
| --- | --- | --- | --- | --- | --- |
| Municipal Wastewater Treatment Plant Load | MWTP | N lbs yr^-1^ | N kg yr^-1^ | CAST Loads Report | CAST Model Documentation |
| Industrial Wastewater Treatment Plant Load | IWTP | N lbs yr^-1^ | N kg yr^-1^ | CAST Loads Report | CAST Model Documentation |
| Combined Sewer Overflow Loads | CSO | N lbs yr^-1^ | N kg yr^-1^ | CAST Loads Report | CAST Model Documentation |
| Septic | Septic | N lbs yr^-1^ | N kg yr^-1^ | CAST Loads Report | CAST Model Documentation |
| Total point source load = MWTP+IWTP+CSO+Septic | TotalPS | N lbs yr^-1^ | N kg yr^-1^ | CAST Loads Report | Derived Variable |
| Total N removed by crop | NCropRemoval | N lbs yr^-1^ | N kg yr^-1^ | CAST Loads Report (with linear scalar adjustment) | CAST Model Documentation |
| Pasture N Removal | NPastureCropRemoval | N lbs yr^-1^ | N kg yr^-1^ | CAST Loads Report (with linear scalar adjustment) | CAST Loads Report (with linear scalar adjustment) |
| Atmospheric Reduced Nitrogen Deposition | NH3 | N lbs yr^-1^ | N kg yr^-1^ | CAST Atmospheric Deposition Report | CAST Model Documentation |
| Atmospheric Oxidized Nitrogen Deposition | NO3 | N lbs yr^-1^ | N kg yr^-1^ | CAST Atmospheric Deposition Report | CAST Model Documentation |
| Atmospheric Organic Nitrogen Deposition (deposited to surface water only) | ORGN | N lbs yr^-1^ | N kg yr^-1^ | CAST Atmospheric Deposition Report | CAST Model Documentation |
| Total Atmospheric Nitrogen Deposition | TotalNDep | N lbs yr^-1^ | N kg yr^-1^ | CAST Atmospheric Deposition Report | CAST Model Documentation |
| Poultry Manure Applied | PoultryManureApplied | N lbs yr^-1^ | N kg yr^-1^ | CAST Nutrients Available Report | CAST Model Documentation |
| Livestock Manure Applied | LivestockManureApplied | N lbs yr^-1^ | N kg yr^-1^ | CAST Nutrients Available Report | CAST Model Documentation |
| Poultry and Livestock Manure Applied (Poultry + Livestock) | PoultryLivestockManureApplied | N lbs yr^-1^ | N kg yr^-1^ | CAST Nutrients Available Report | CAST Model Documentation |
| Livestock Manure Deposited into Stream/Riparian Area | RiparianPastureNutrient | N lbs yr^-1^ | N kg yr^-1^ | CAST Nutrients Available Report | CAST Model Documentation |
| Agriculture Biosolids | AgBiosolids | N lbs yr^-1^ | N kg yr^-1^ | CAST Nutrients Available Report | CAST Model Documentation |
| Urban Biosolids | DevBiosolids | N lbs yr^-1^ | N kg yr^-1^ | CAST Nutrients Available Report | CAST Model Documentation |
| Direct Manure Deposited on Pasture | DirectDepositManureN | N lbs yr^-1^ | N kg yr^-1^ | CAST Nutrients Available Report | CAST Model Documentation |
| Agriculture Fertilizer | AgFertilizerN | N lbs yr^-1^ | N kg yr^-1^ | CAST Nutrients Available Report | CAST Model Documentation |
| Urban Fertilizer | UrbanFertilizerN | N lbs yr^-1^ | N kg yr^-1^ | CAST Nutrients Available Report | CAST Model Documentation |
| Total Agricultural Application (PoultryLivestock + Agricultural Fertilizer) | TotalAgApplication | N lbs yr^-1^ | N kg yr^-1^ | CAST Nutrients Available Report | Derived Variable |
| Total N Fixed by Legumes | Legume | N lbs yr^-1^ | N kg yr^-1^ | CAST Nutrients Available Report | CAST Model Documentation |
| Total Nitrogen Input (Legume+ PoultryLivestock+ RiparianPastureNutrient+ TotalPS+ TotalNDep+ UrbanFertilizerN+ AgFertilizerN) | TN | N lbs yr^-1^ | N kg yr^-1^ | CAST Nutrients Available Report | Derived Variable |
| Agrilcultural Surplus (Legume+ PoultryLivestock+ Atm Dep On Ag Land+ AgFertilizerN- NCropRemoval) | AgSurplus | N lbs yr^-1^ | N kg yr^-1^ | CAST Nutrients Available Report | Derived Variable |
| Nitrogen Use Efficiency (NCropRemoval/(TotalAgApplication + Atm Dep On Ag Land)) | NUE | Unitless | N kg yr^-1^ | CAST Nutrients Available Report | Derived Variable |

Table S2: List of phosphorus fluxes and other derived variables as well as the data source and the name of the section presenting detailed methodologies at <https://cast.chesapeakebay.net/Documentation/ModelDocumentation>. This table is also reference for column headers in the county level supplemental database (1985-2019).

| Variable Name | Abbreviation | Original Unit | Unit in Supplemental Database | Source | Access to Description of Methodology |
| --- | --- | --- | --- | --- | --- |
| Municipal Wastewater Treatment Plant Load | MWTP | P lbs yr^-1^ | P kg yr^-1^ | CAST Loads Report | CAST Model Documentation |
| Industrial Wastewater Treatment Plant Load | IWTP | P lbs yr^-1^ | P kg yr^-1^ | CAST Loads Report | CAST Model Documentation |
| Combined Sewer Overflow Loads | CSO | P lbs yr^-1^ | P kg yr^-1^ | CAST Loads Report | CAST Model Documentation |
| Septic | SEPTIC | P lbs yr^-1^ | P kg yr^-1^ | CAST Loads Report | CAST Model Documentation |
| Total point source load (MWTP+IWTP+CSO+Septic) | TotalPS | P lbs yr^-1^ | P kg yr^-1^ | CAST Loads Report | Derived Variable |
| Total P Removed by Crop Including Pasture | PCropRemoval | P lbs yr^-1^ | P kg yr^-1^ | CAST Loads Report (with linear scalar adjustment) | CAST Model Documentation |
| Pasture P Removal | PPastureCropRemoval | P lbs yr^-1^ | P kg yr^-1^ | CAST Loads Report (with linear scalar adjustment) | CAST Loads Report (with linear scalar adjustment) |
| Atmospheric Inorganic P Deposition (deposited onto water) | PO4LbsPerAcre | P lbs yr^-1^ | P kg yr^-1^ | CAST Atmospheric Deposition Report | CAST Model Documentation |
| Atmospheric Organic P Deposition (deposited onto water only) | ORGPLbsPerAcre | P lbs yr^-1^ | P kg yr^-1^ | CAST Atmospheric Deposition Report | CAST Model Documentation |
| Total Atmospheric P Deposition (PO4LbsPerAcre+ORGLbsPerAcre | TPLbsPerAcre | P lbs yr^-1^ | P kg yr^-1^ | CAST Atmospheric Deposition Report | CAST Model Documentation |
| Poultry manure applied | PoultryManureApplied | P lbs yr^-1^ | P kg yr^-1^ | CAST Atmospheric Deposition Report | CAST Model Documentation |
| Livestock manure applied | LivestockManureApplied | P lbs yr^-1^ | P kg yr^-1^ | CAST Nutrients Available Report | CAST Model Documentation |
| Poultry and Livestock manure applied (Poultry + Livestock) | PoultryLivestockManureApplied | P lbs yr^-1^ | P kg yr^-1^ | CAST Nutrients Available Report | CAST Model Documentation |
| Livestock manure deposited into stream/riparian area | RiparianPastureNutrient | P lbs yr^-1^ | P kg yr^-1^ | CAST Nutrients Available Report | CAST Model Documentation |
| Agriculture Biosolids | AgBiosolids | P lbs yr^-1^ | P kg yr^-1^ | CAST Nutrients Available Report | CAST Model Documentation |
| Urban Biosolids | DevBiosolids | P lbs yr^-1^ | P kg yr^-1^ | CAST Nutrients Available Report | CAST Model Documentation |
| Direct manure deposited on pasture | DirectDepositManureP | P lbs yr^-1^ | P kg yr^-1^ | CAST Nutrients Available Report | CAST Model Documentation |
| Agriculture Fertilizer | AgFertilizer | P lbs yr^-1^ | P kg yr^-1^ | CAST Nutrients Available Report | CAST Model Documentation |
| Urban Fertilizer | UrbanFertilizerPLbsApplied | P lbs yr^-1^ | P kg yr^-1^ | CAST Nutrients Available Report | CAST Model Documentation |
| Total Agricultural Application (PoultryLivestock + ag fert) | TotalAgApplication | P lbs yr^-1^ | P kg yr^-1^ | CAST Nutrients Available Report | CAST Model Documentation |
| Mass of P in the Soil | PSoilLbs | P lbs yr^-1^ | P kg yr^-1^ | CAST Nutrients Available Report | CAST Model Documentation |
| Total Phosphorus Inputs (PoultryLivestock+ RiparianPastureNutrient+ TotalPS+ TotalPDep+ UrbanFertilizerP+ AgFertilizerNP) | TP | P lbs yr^-1^ | P kg yr^-1^ | CAST Nutrients Available Report | CAST Model Documentation |
| Agricultural Surplus (PoultryLivestock+ AgFertilizerP- (PCropRemoval- Pasture P Removal)) | AgSurplus | P lbs yr^-1^ | P kg yr^-1^ | CAST Nutrients Available Report | Derived Variable |
| Phosphorus Use Efficiency (PCropRemoval/(PoultryLivestock+ AgFertilizerP)) | PUE | Unitless | P kg yr^-1^ | CAST Nutrients Available Report | Derived Variable |


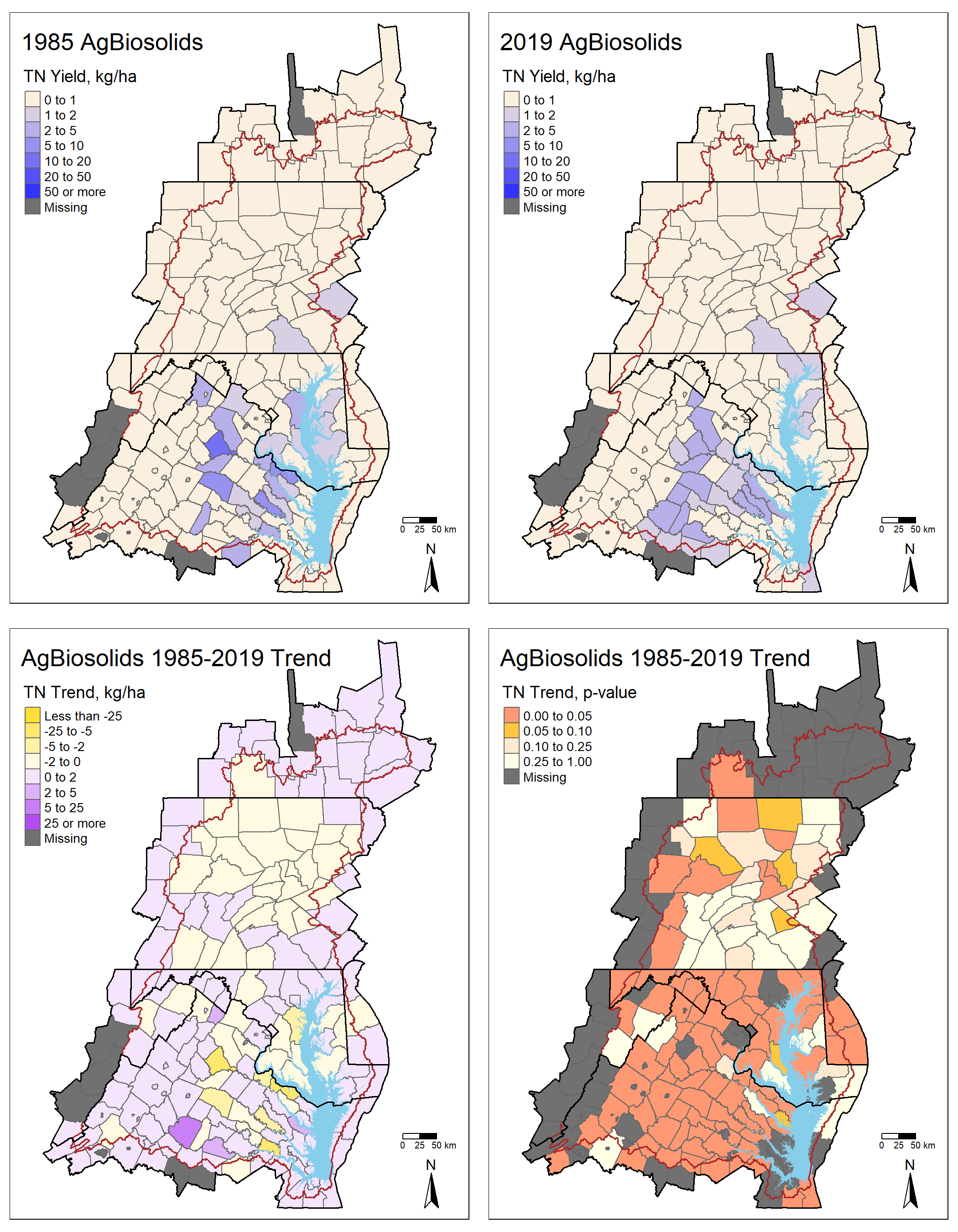


Figure S1. For nitrogen, 1985 and 2019 biosolids applied to agricultural land (top row), the estimated Sen linear slope change in biosolid application from 1985-2019 (bottom left), and the significance of trend results by county (bottom right).
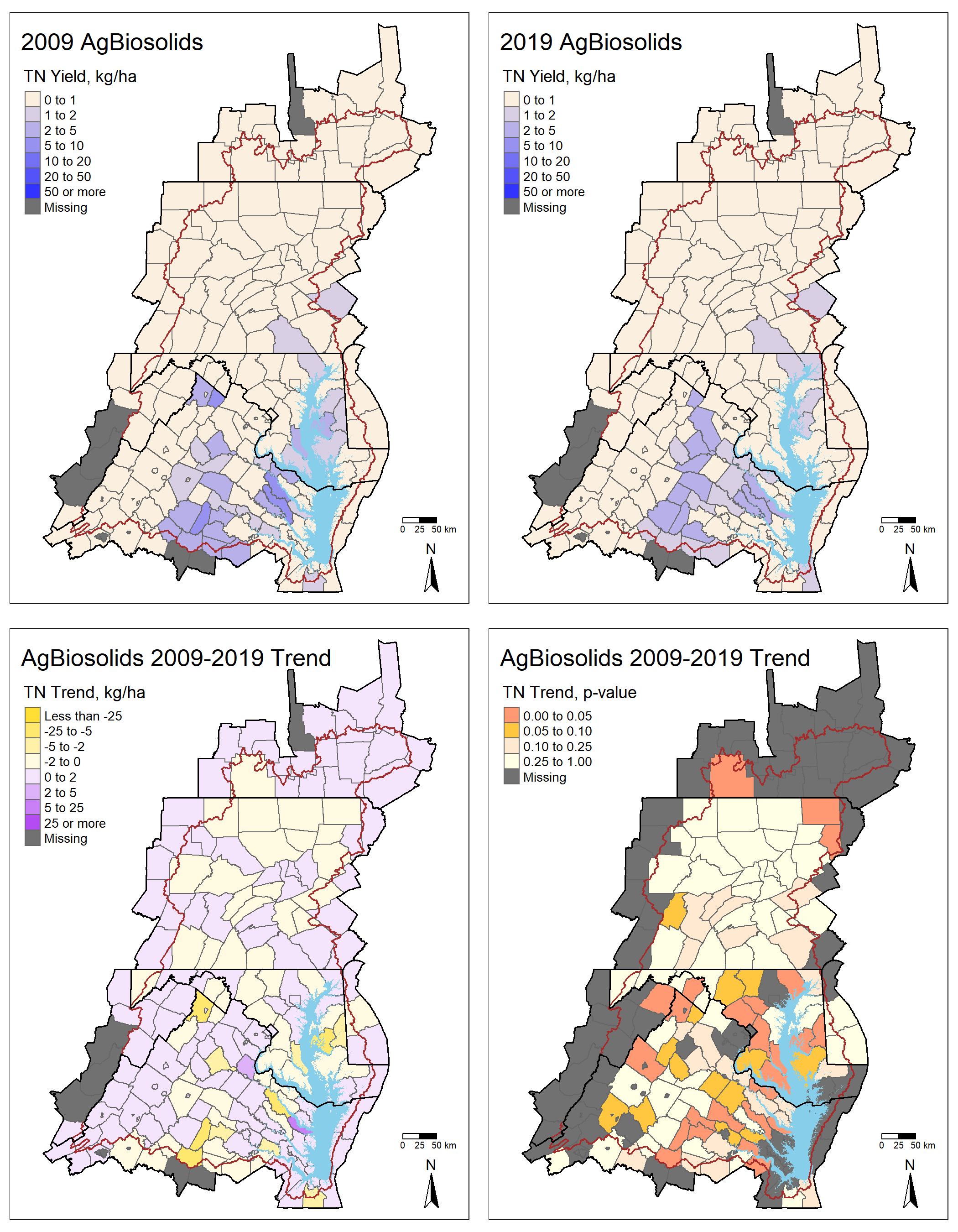
 Figure S2. For nitrogen, 2009 and 2019 biosolids applied to agricultural land (top row), the estimated Sen linear slope change in biosolid application from 2009-2019 (bottom left), and the significance of trend results by county (bottom right).


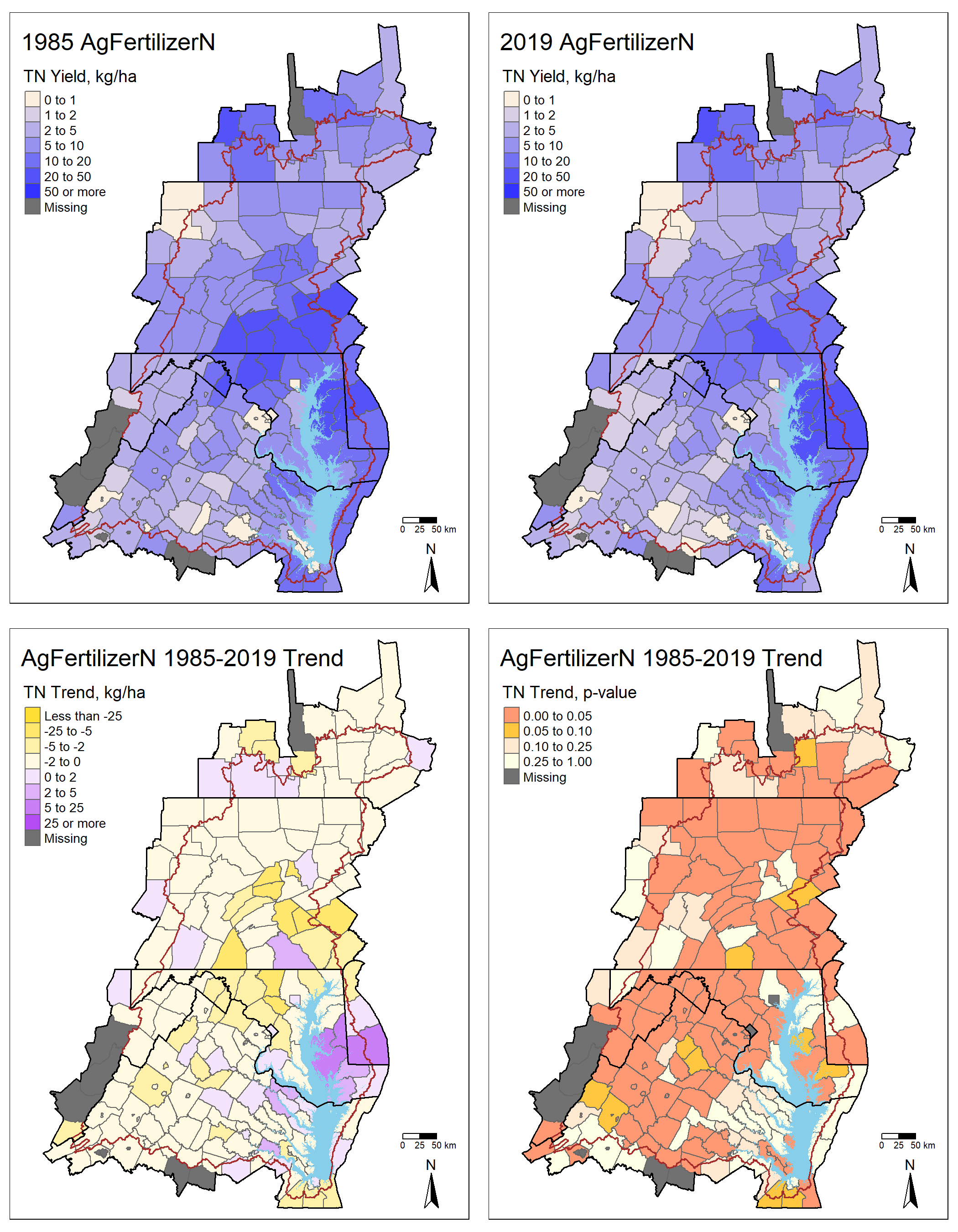


Figure S3. For nitrogen, 1985 and 2019 fertilizer applied to agricultural land (top row), the estimated Sen linear slope change in fertilizer application from 1985-2019 (bottom left), and the significance of trend results by county (bottom right).
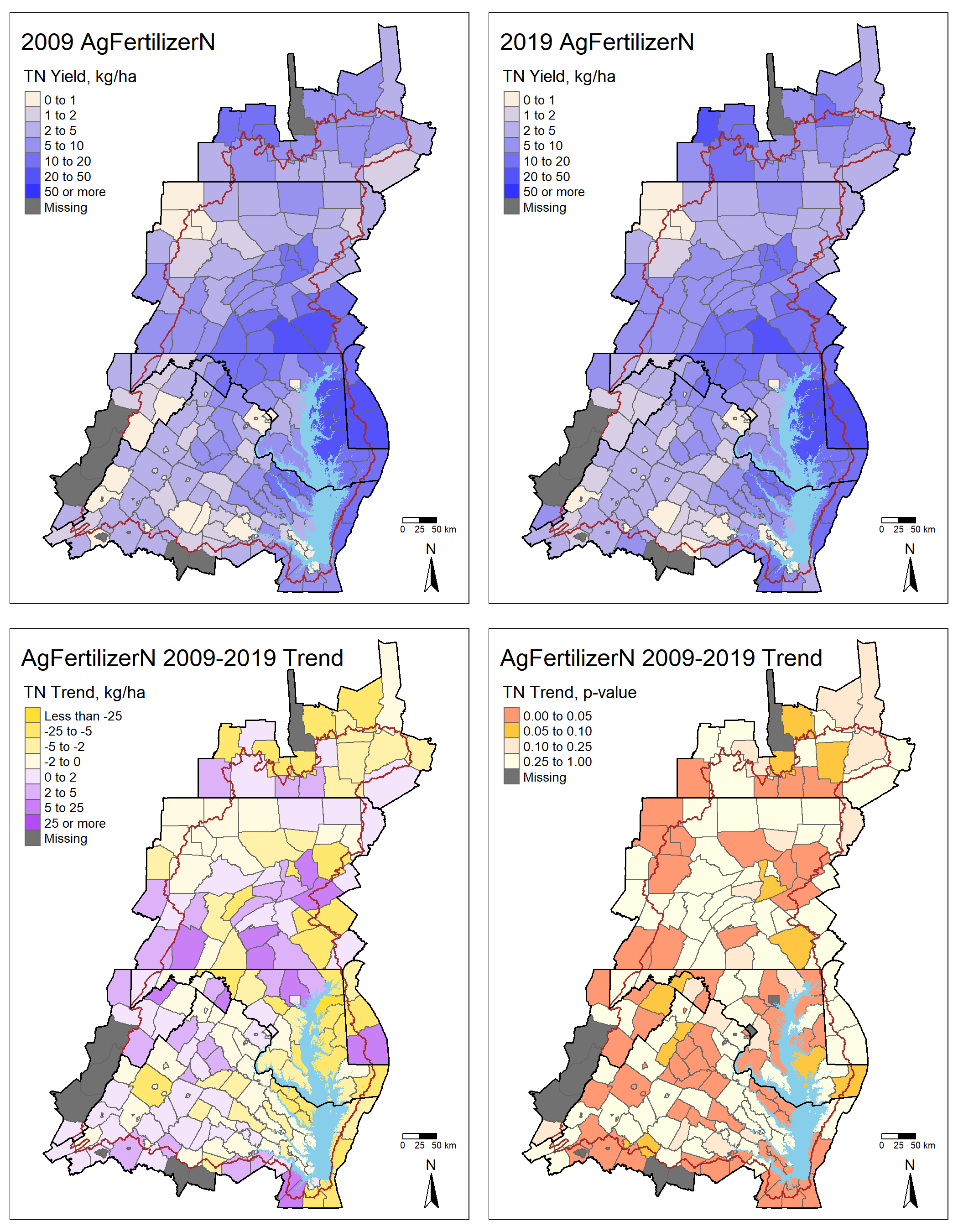
 Figure S4. For nitrogen, 2009 and 2019 fertilizer applied to agricultural land (top row), the estimated Sen linear slope change in fertilizer application from 2009-2019 (bottom left), and the significance of trend results by county (bottom right).
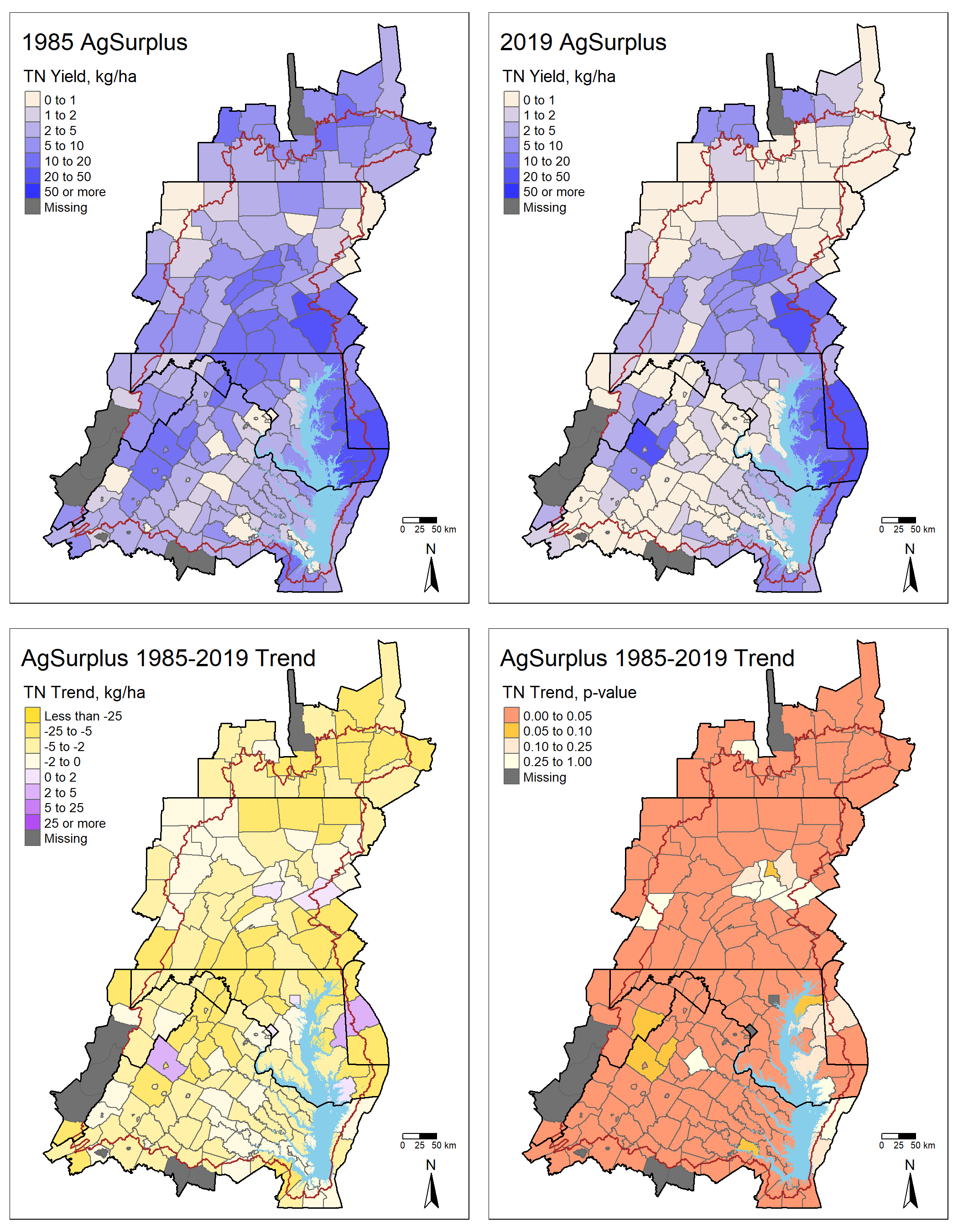
 Figure S5. For nitrogen, 1985 and 2019 agricultural surplus (top row), the estimated Sen linear slope change in agricultural surplus from 1985-2019 (bottom left), and the significance of trend results by county (bottom right).
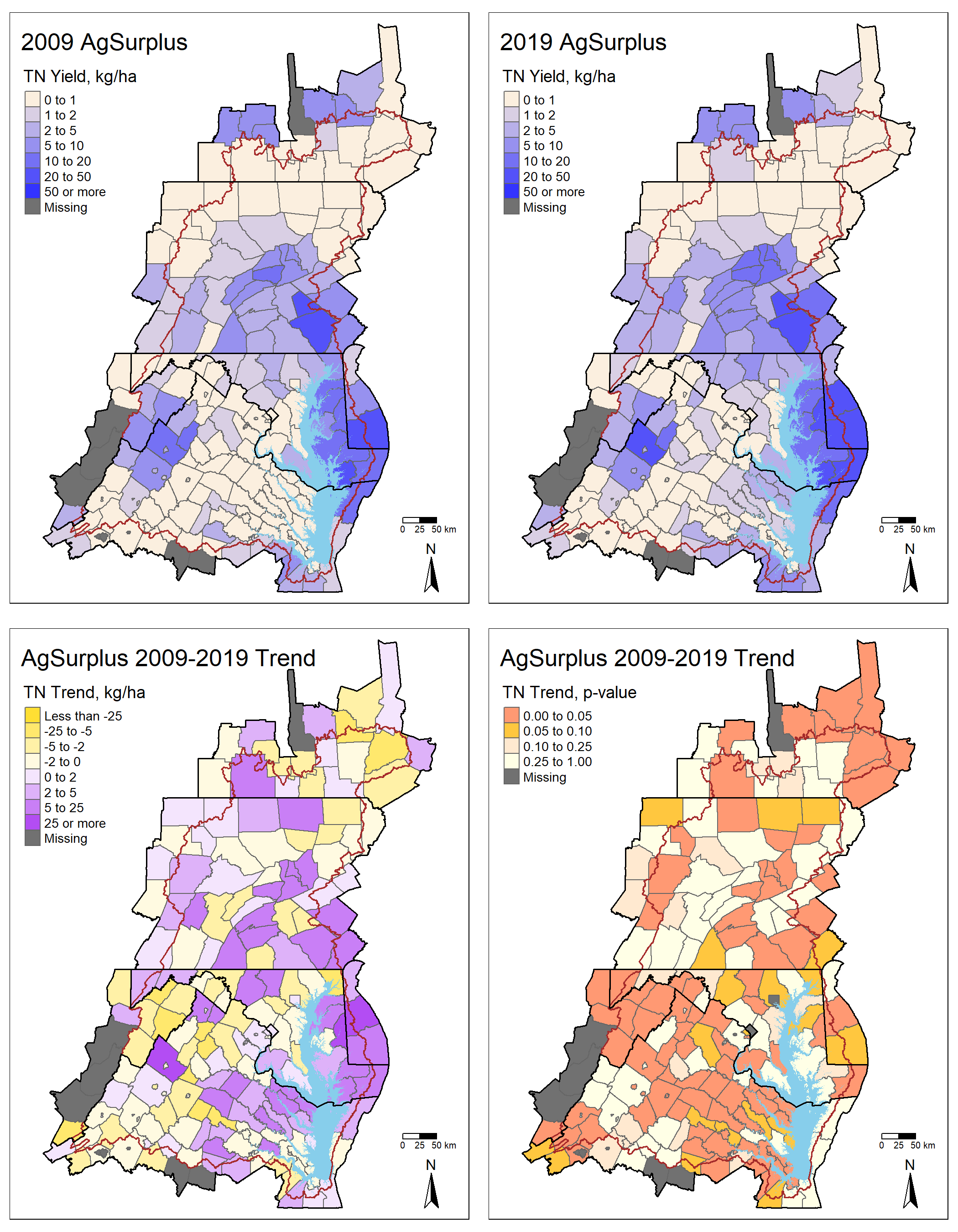
 Figure S6. For nitrogen, 2009 and 2019 agricultural surplus (top row), the estimated Sen linear slope change in agricultural surplus from 2009-2019 (bottom left), and the significance of trend results by county (bottom right).
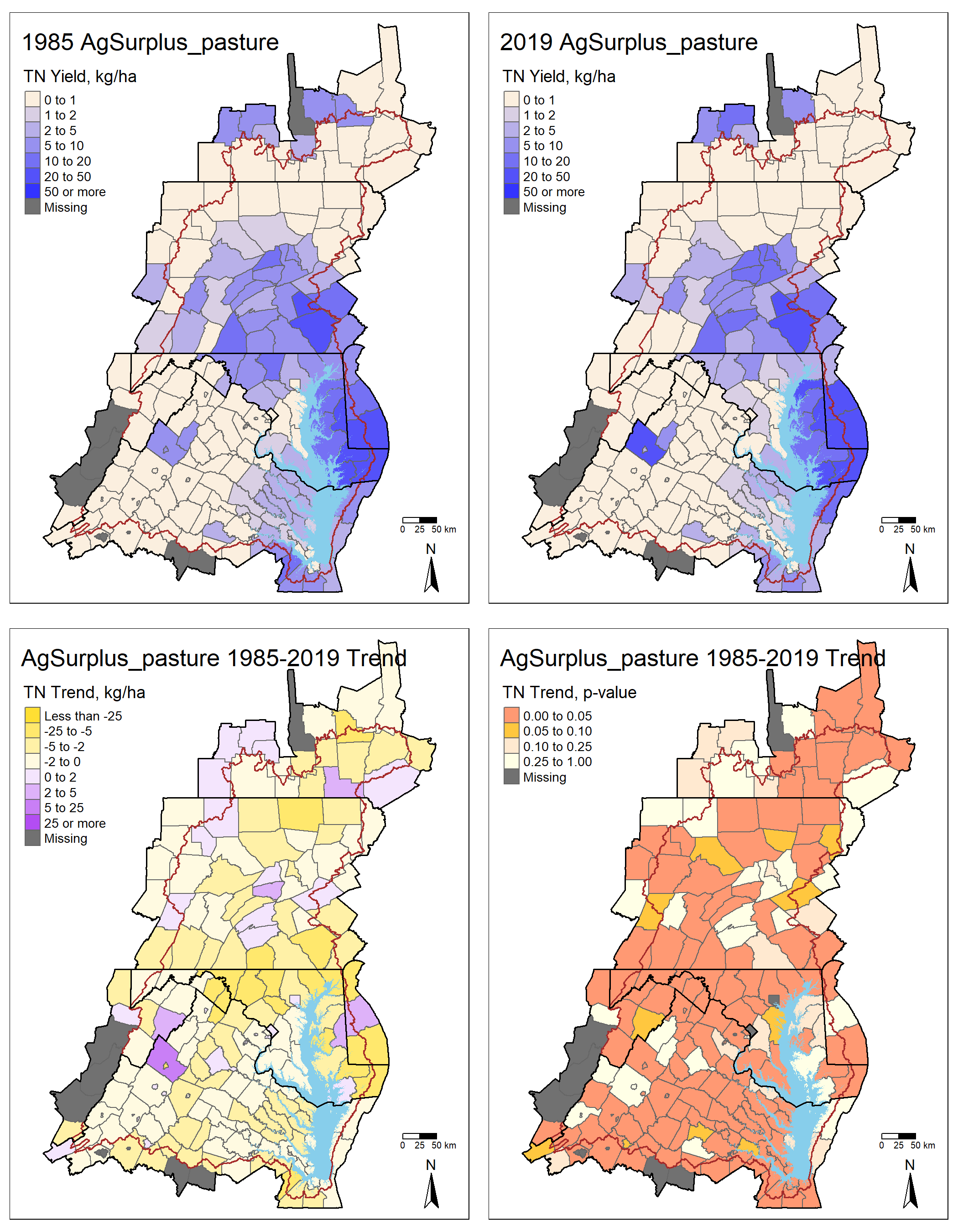
 Figure S7. For nitrogen, 1985 and 2019 agricultural surplus with pasture terms (top row), the estimated Sen linear slope change in agricultural surplus with pasture terms from 1985-2019 (bottom left), and the significance of trend results by county (bottom right).
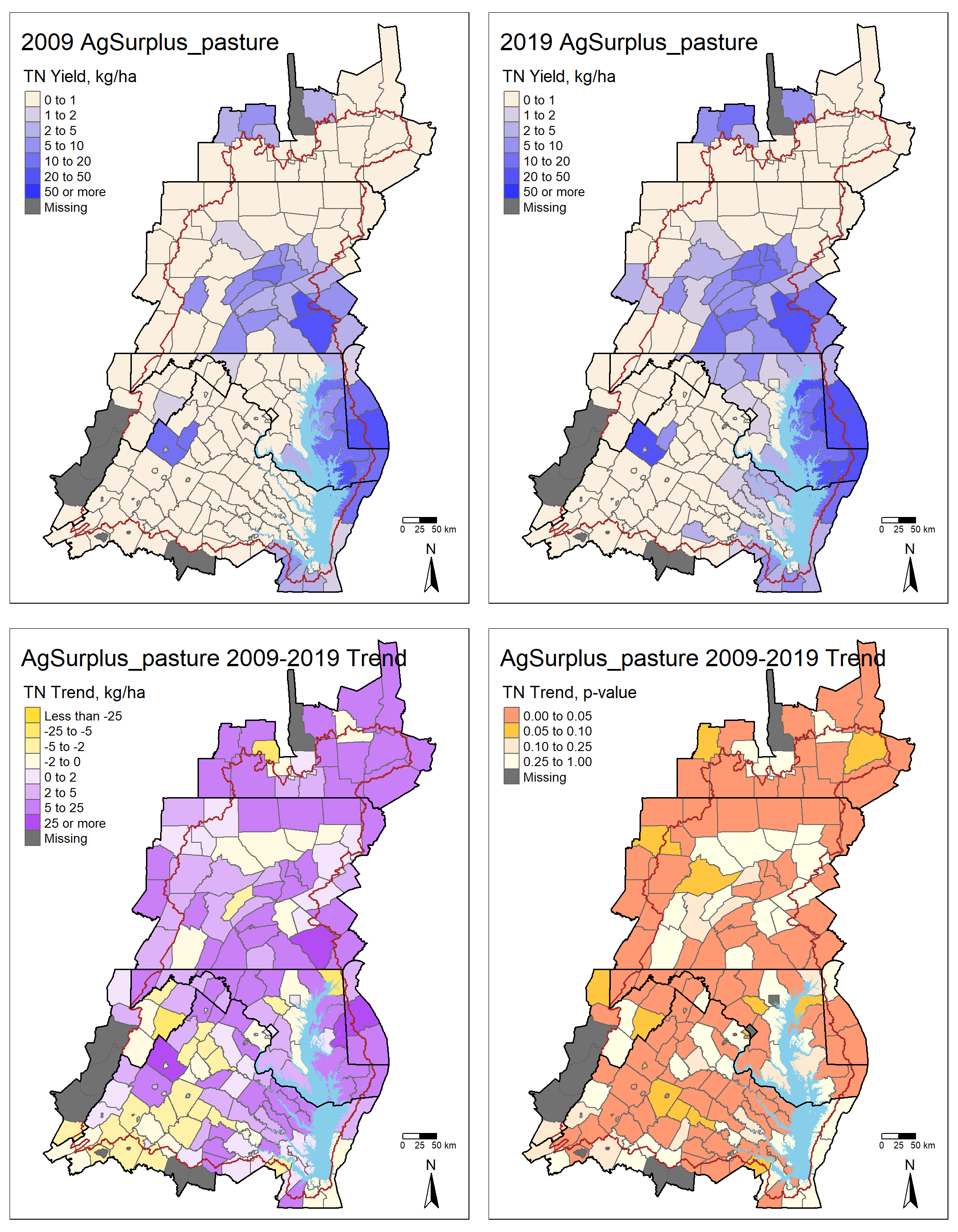
 Figure S8. For nitrogen, 2009 and 2019 agricultural surplus with pasture terms (top row), the estimated Sen linear slope change in agricultural surplus with pasture terms from 2009-2019 (bottom left), and the significance of trend results by county (bottom right).
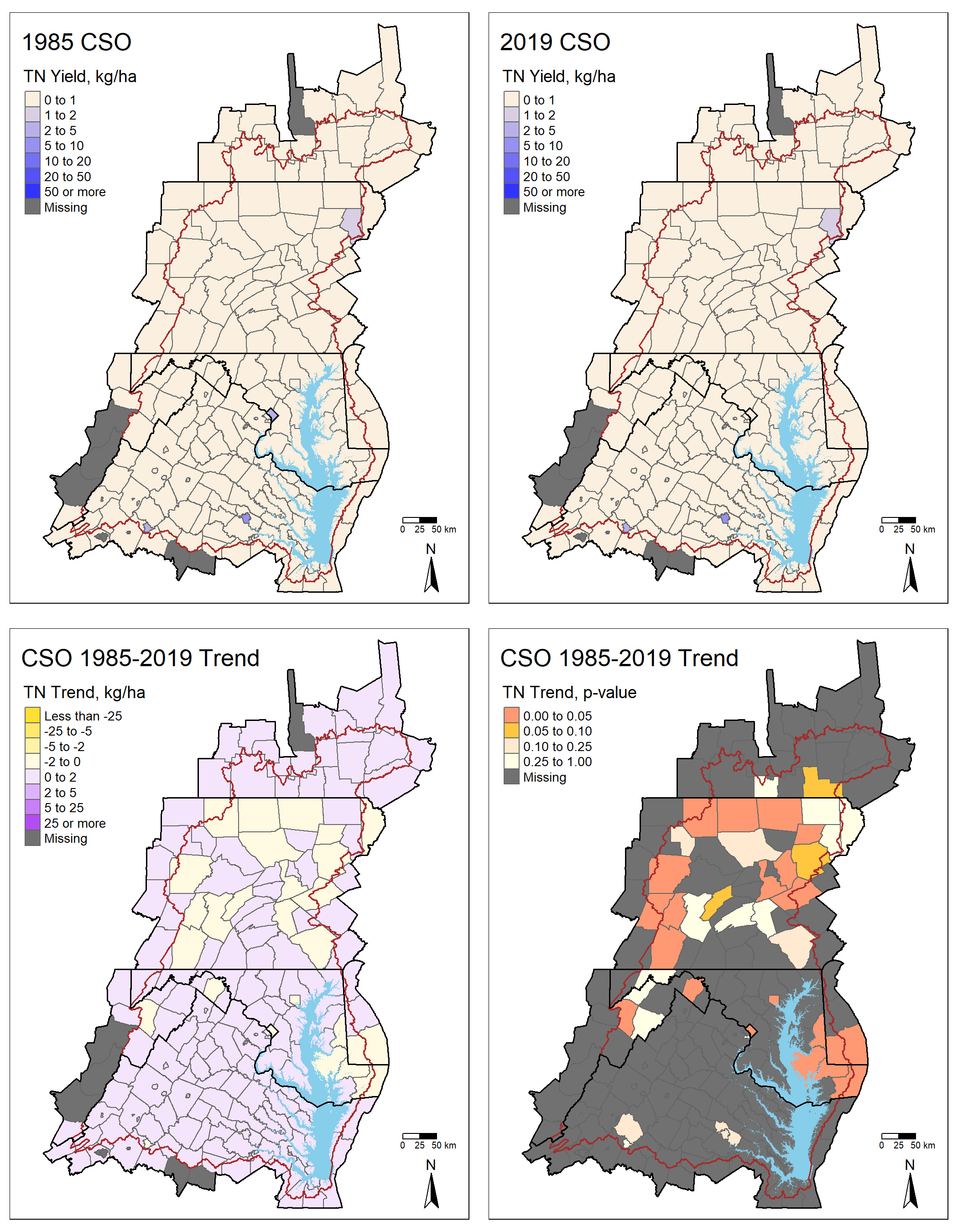
 Figure S9. For nitrogen, 1985 and 2019 combined sewage overflow loads (top row), the estimated Sen linear slope change in combined sewage overflow loads from 1985-2019 (bottom left), and the significance of trend results by county (bottom right).
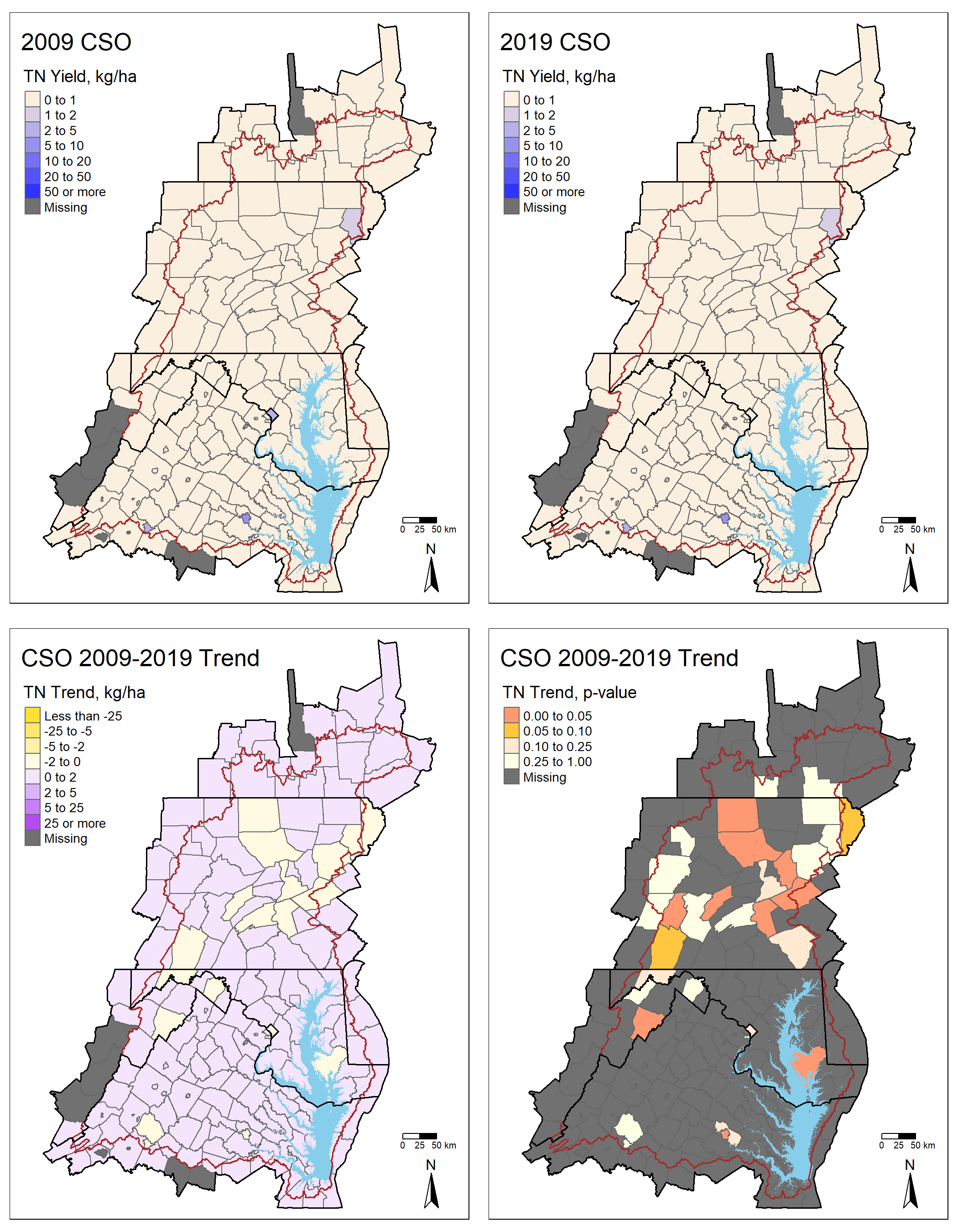
Figure S10. For nitrogen, 2009 and 2019 combined sewage overflow loads (top row), the estimated Sen linear slope change in combined sewage overflow loads from 2009-2019 (bottom left), and the significance of trend results by county (bottom right).
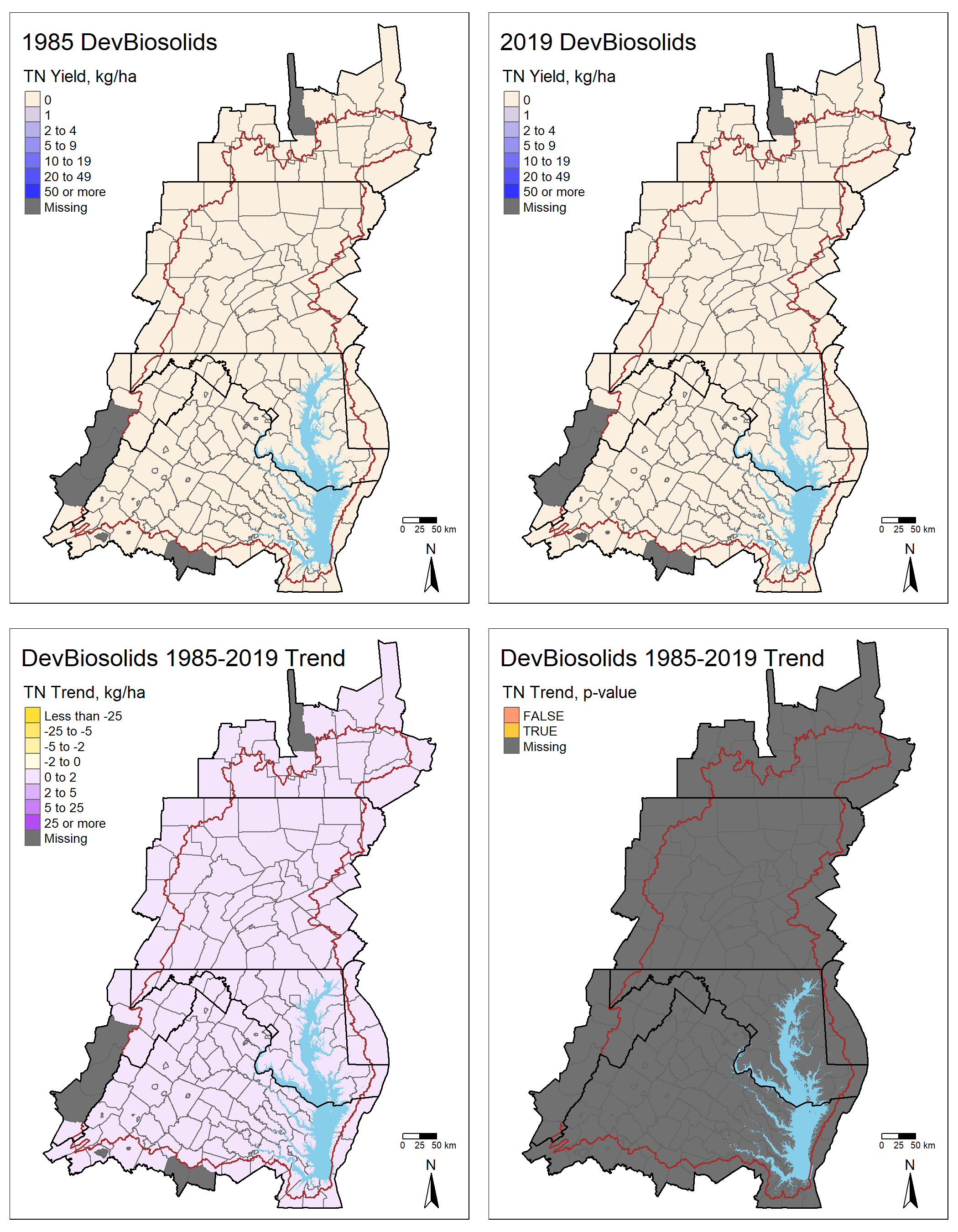
 Figure S11. For nitrogen, 1985 and 2019 biosolids applied to developed land (top row), the estimated Sen linear slope change in biosolid application from 1985-2019 (bottom left), and the significance of trend results by county (bottom right).
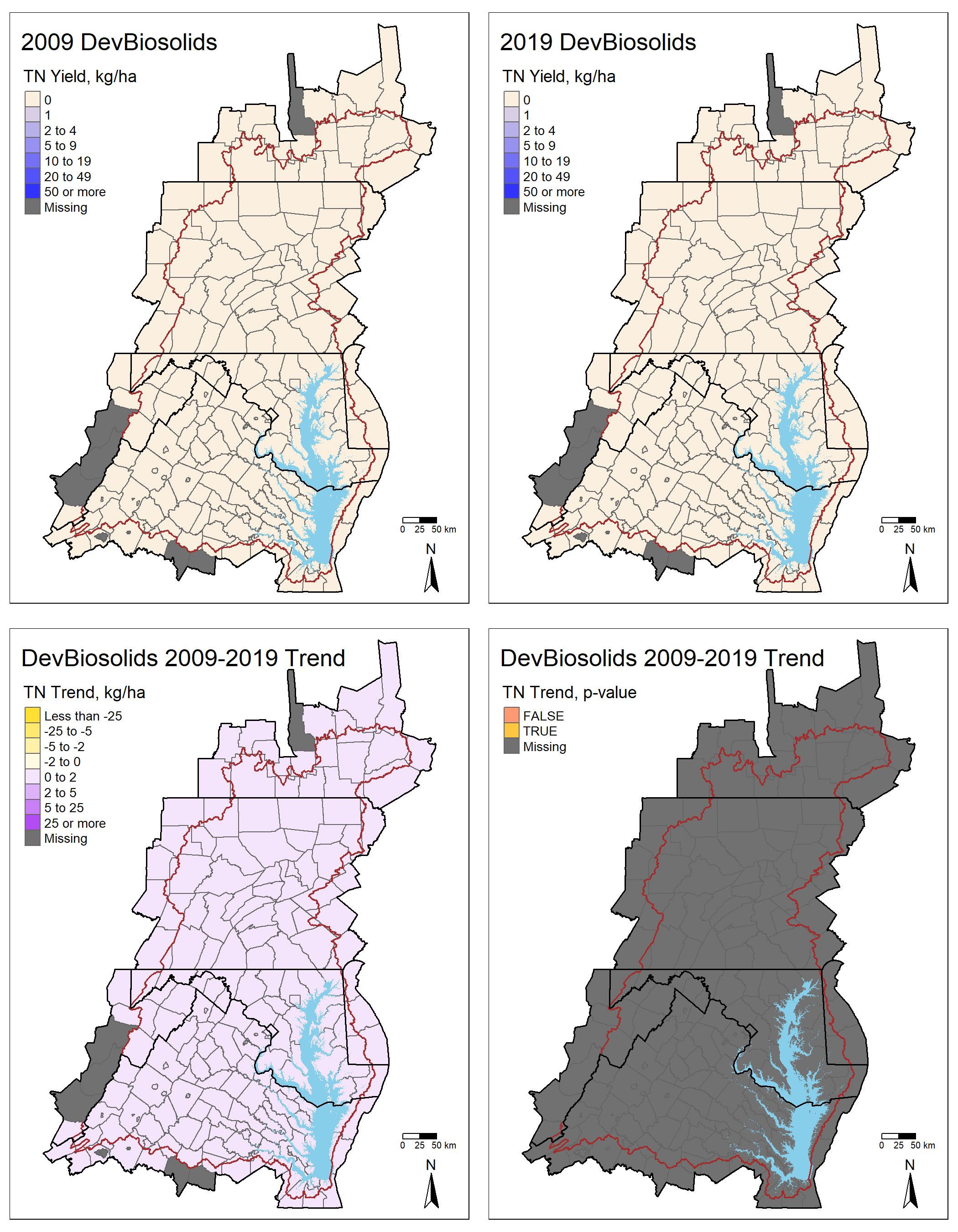
 Figure S12. For nitrogen, 2009 and 2019 biosolids applied to developed land (top row), the estimated Sen linear slope change in biosolid application from 2009-2019 (bottom left), and the significance of trend results by county (bottom right).


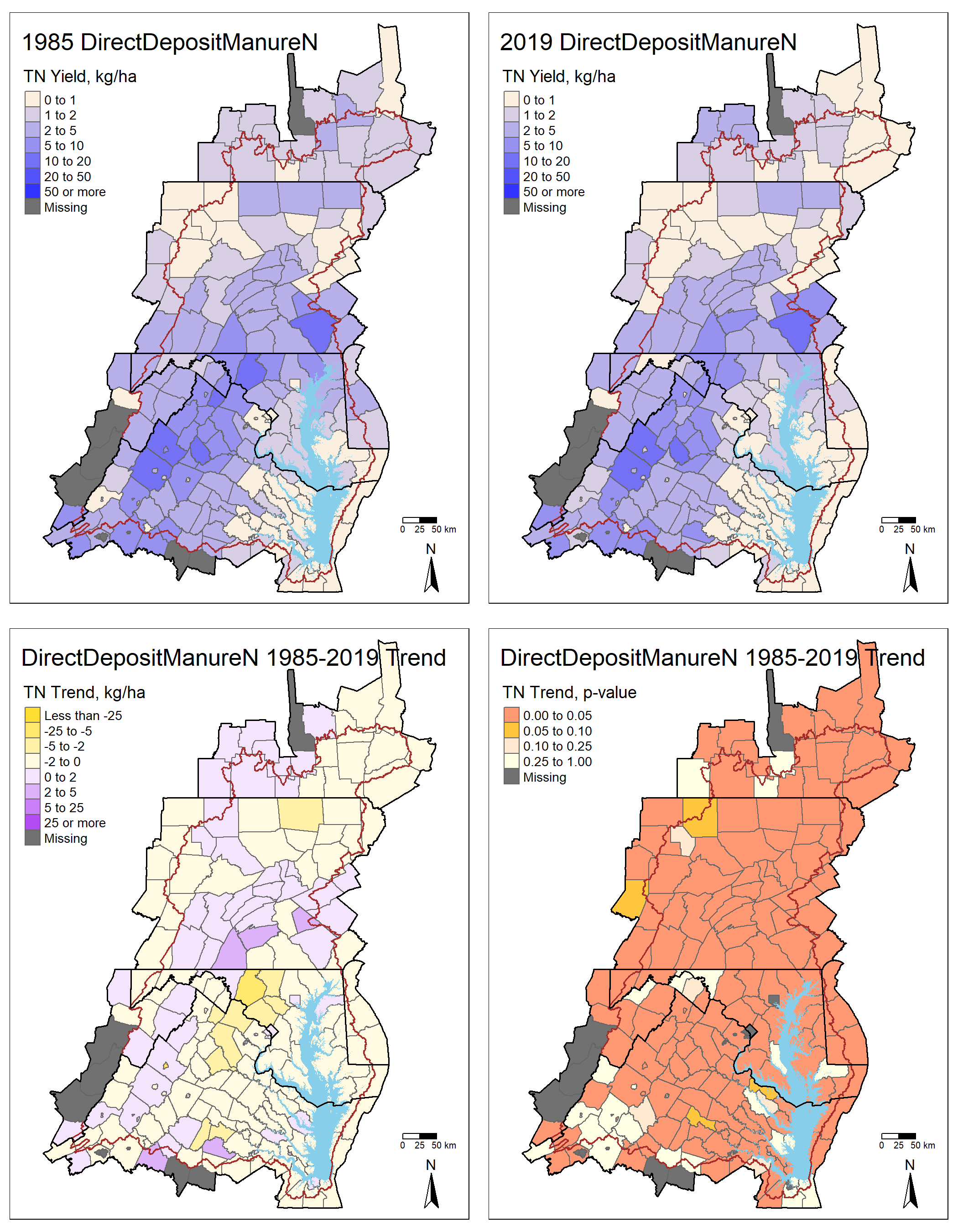
Figure S13. For nitrogen, 1985 and 2019 direct manure deposited on pasture (top row), the estimated Sen linear slope change in direct manure deposited on pasture from 1985-2019 (bottom left), and the significance of trend results by county (bottom right).
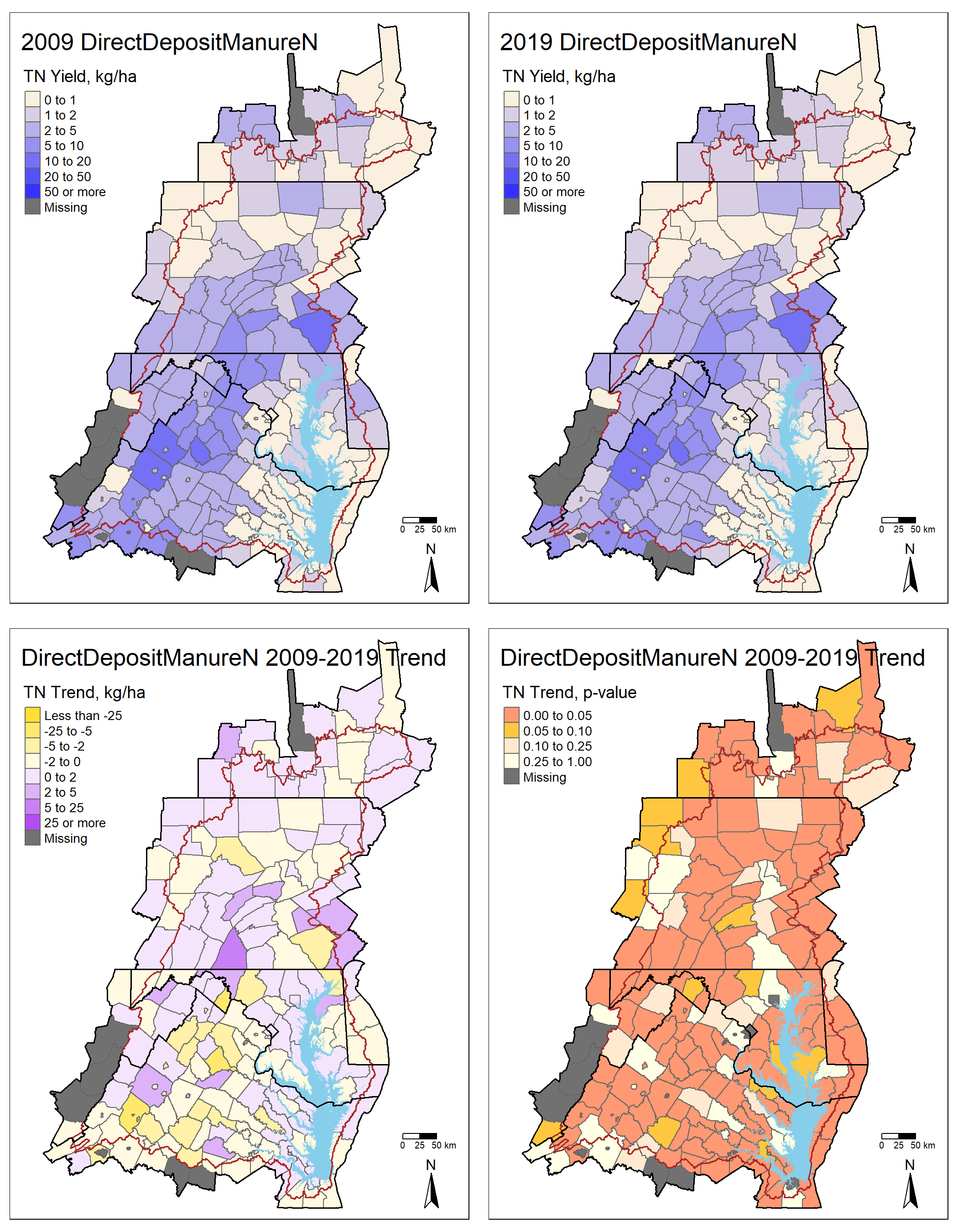
 Figure S14. For nitrogen, 2009 and 2019 direct manure deposited on pasture (top row), the estimated Sen linear slope change in direct manure deposited on pasture from 2009-2019 (bottom left), and the significance of trend results by county (bottom right).
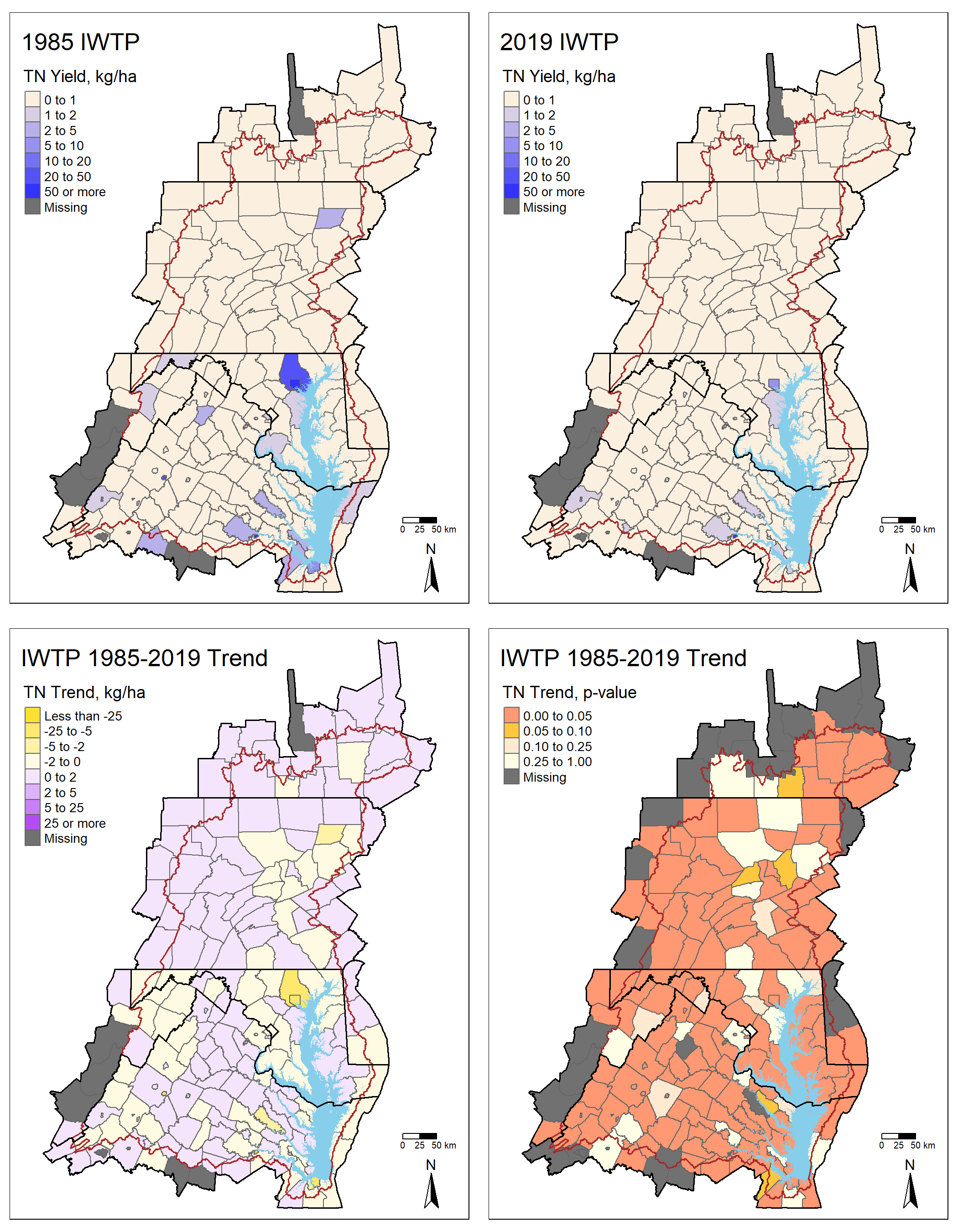
 Figure S15. For nitrogen, 1985 and 2019 industrial wastewater treatment plant load (top row), the estimated Sen linear slope change in industrial wastewater treatment plant load from 1985-2019 (bottom left), and the significance of trend results by county (bottom right).
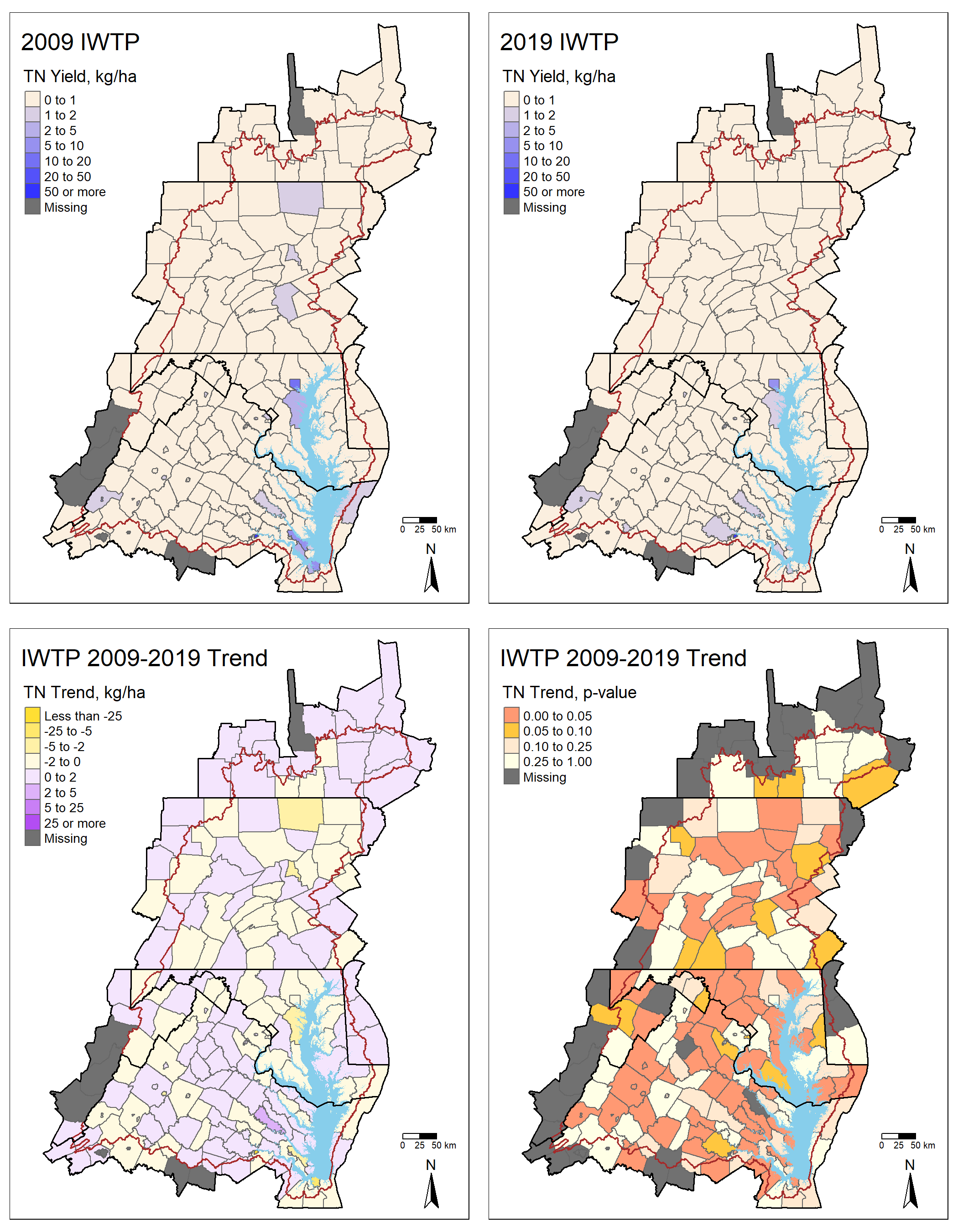
 Figure S16. For nitrogen, 2009 and 2019 industrial wastewater treatment plant load (top row), the estimated Sen linear slope change in industrial wastewater treatment plant load from 2009-2019 (bottom left), and the significance of trend results by county (bottom right).
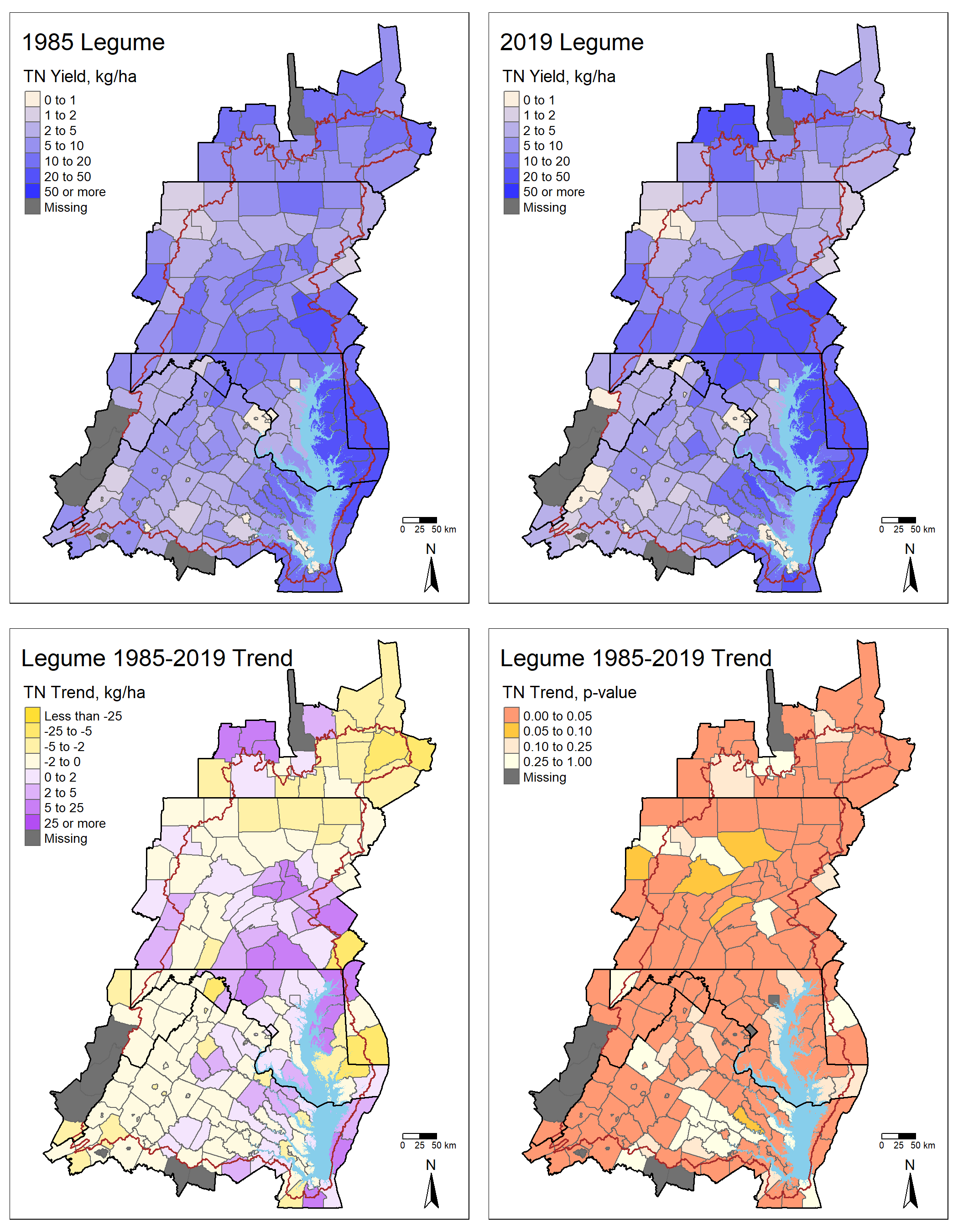
 Figure S17. For nitrogen, 1985 and 2019 total N fixed by legumes (top row), the estimated Sen linear slope change in total N fixed by legumes from 1985-2019 (bottom left), and the significance of trend results by county (bottom right).
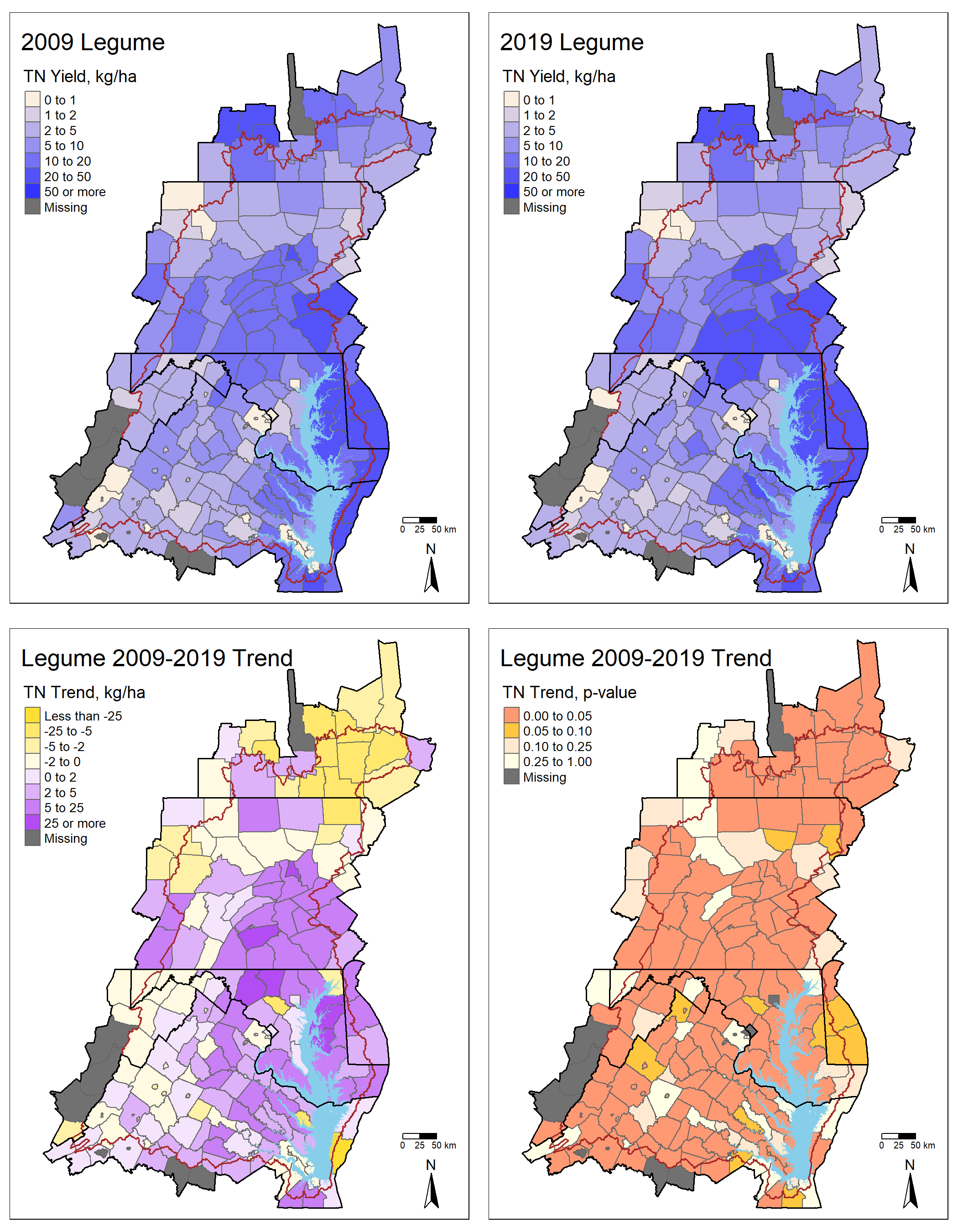
 Figure S18. For nitrogen, 2009 and 2019 total N fixed by legumes (top row), the estimated Sen linear slope change in total N fixed by legumes from 2009-2019 (bottom left), and the significance of trend results by county (bottom right).
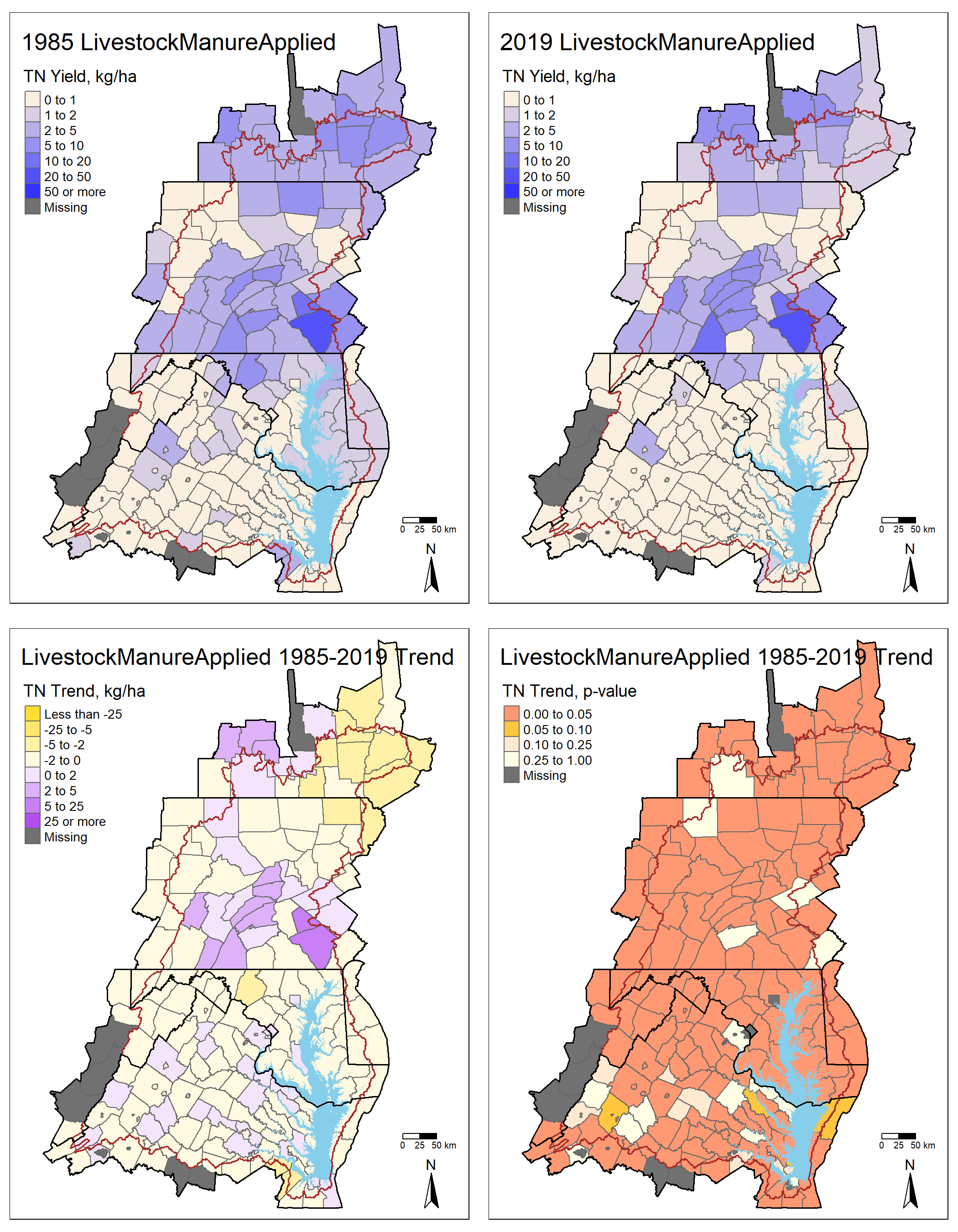
 Figure S19. For nitrogen, 1985 and 2019 livestock manure applied (top row), the estimated Sen linear slope change in livestock manure applied from 1985-2019 (bottom left), and the significance of trend results by county (bottom right).
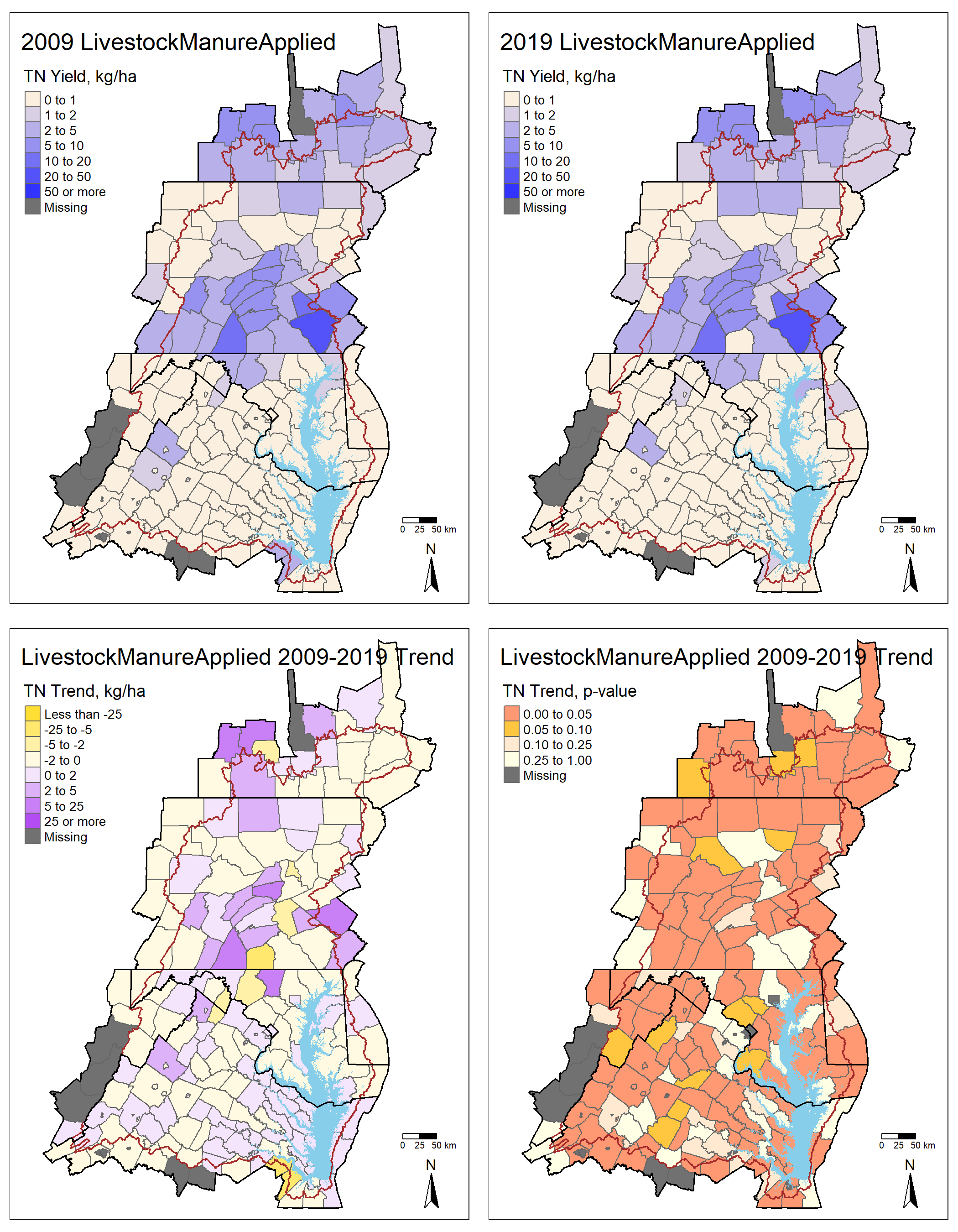
 Figure S20. For nitrogen, 2009 and 2019 livestock manure applied (top row), the estimated Sen linear slope change in livestock manure applied from 2009-2019 (bottom left), and the significance of trend results by county (bottom right).
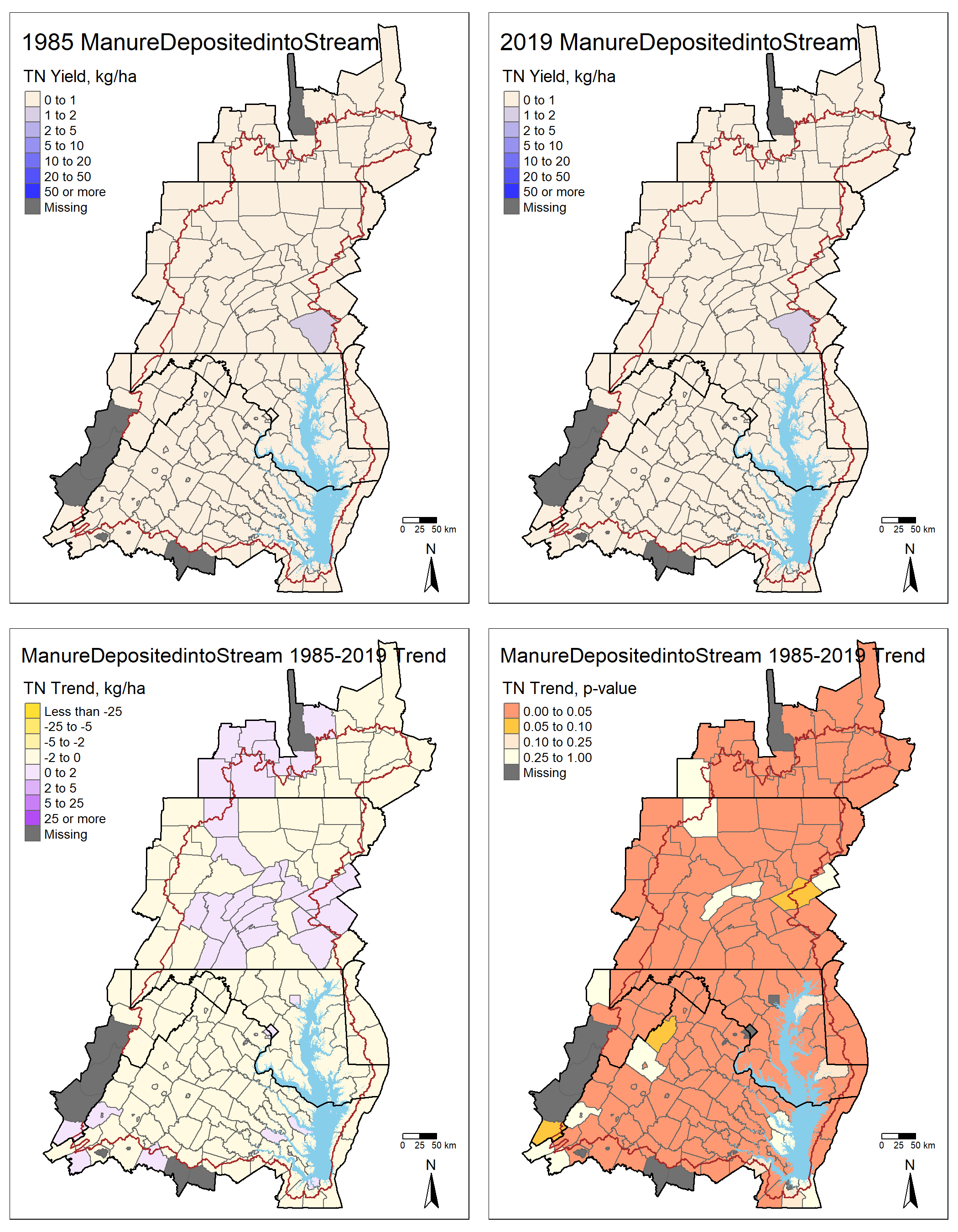
 Figure S21. For nitrogen, 1985 and 2019 livestock manure deposited into stream/riparian areas (top row), the estimated Sen linear slope change in livestock manure deposited into stream/riparian areas from 1985-2019 (bottom left), and the significance of trend results by county (bottom right).
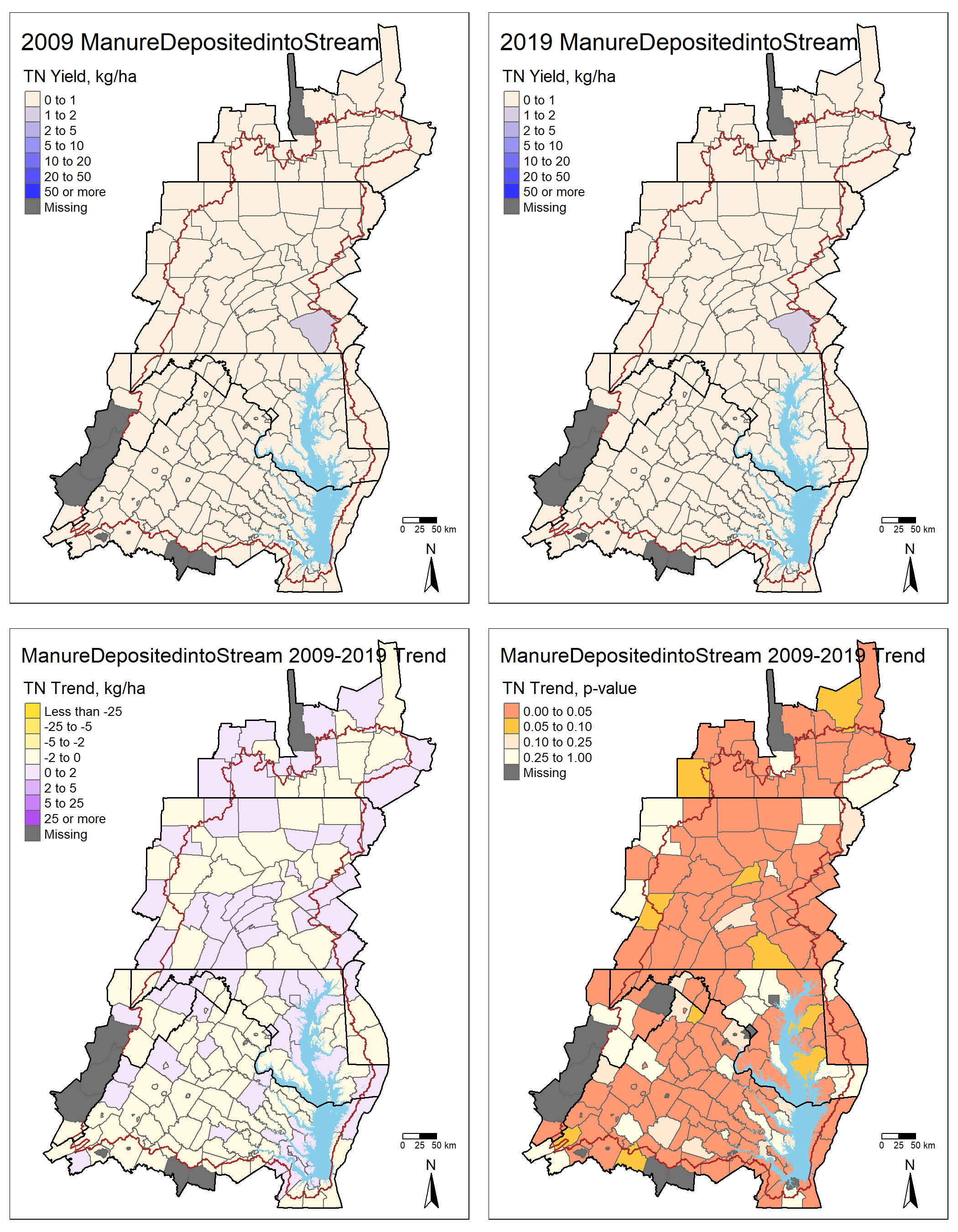
 Figure S22. For nitrogen, 2009 and 2019 livestock manure deposited into stream/riparian areas (top row), the estimated Sen linear slope change in livestock manure deposited into stream/riparian areas from 2009-2019 (bottom left), and the significance of trend results by county (bottom right).
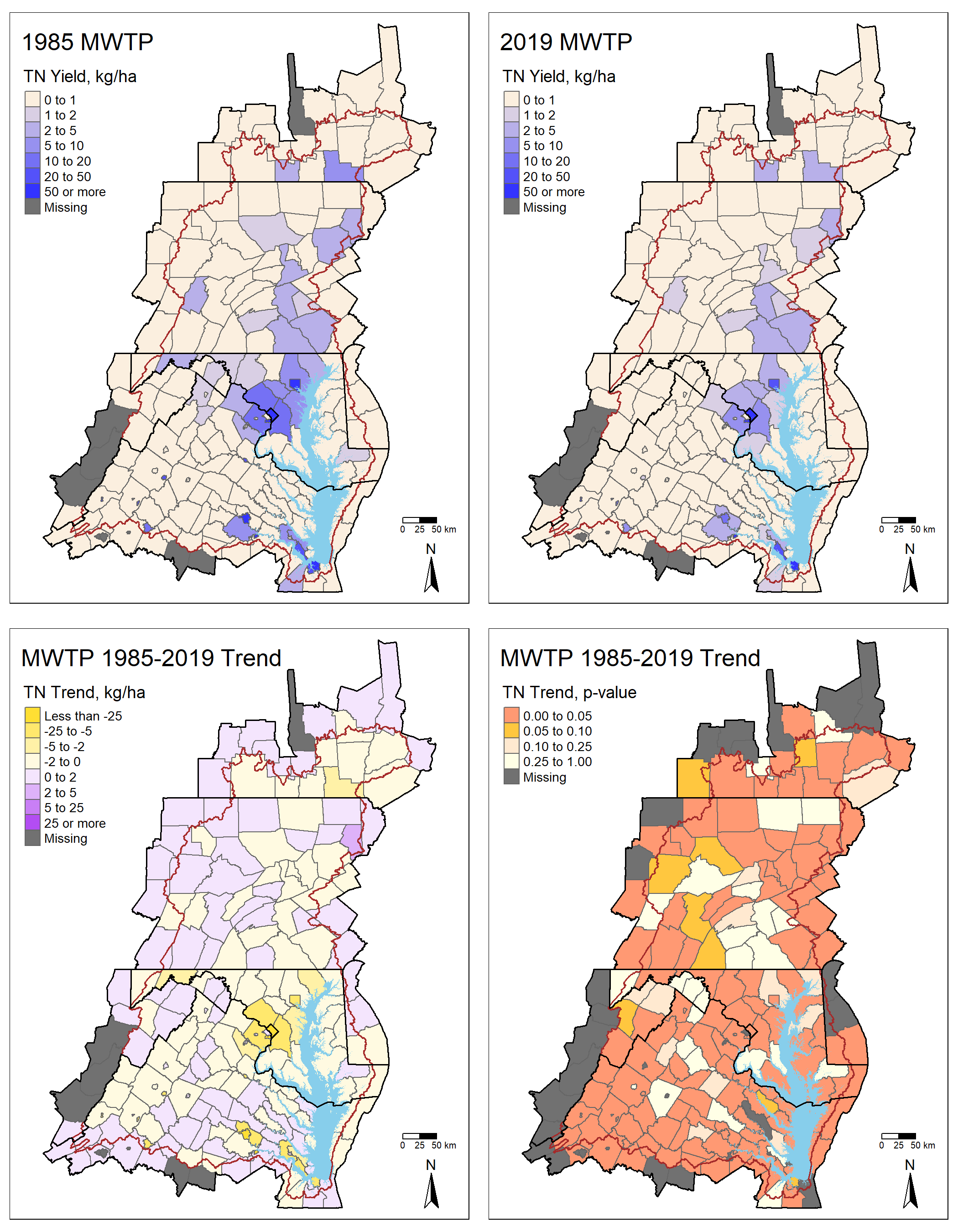
 Figure S23. For nitrogen, 1985 and 2019 municipal wastewater treatment plant load (top row), the estimated Sen linear slope change in municipal wastewater treatment plant load from 1985-2019 (bottom left), and the significance of trend results by county (bottom right).
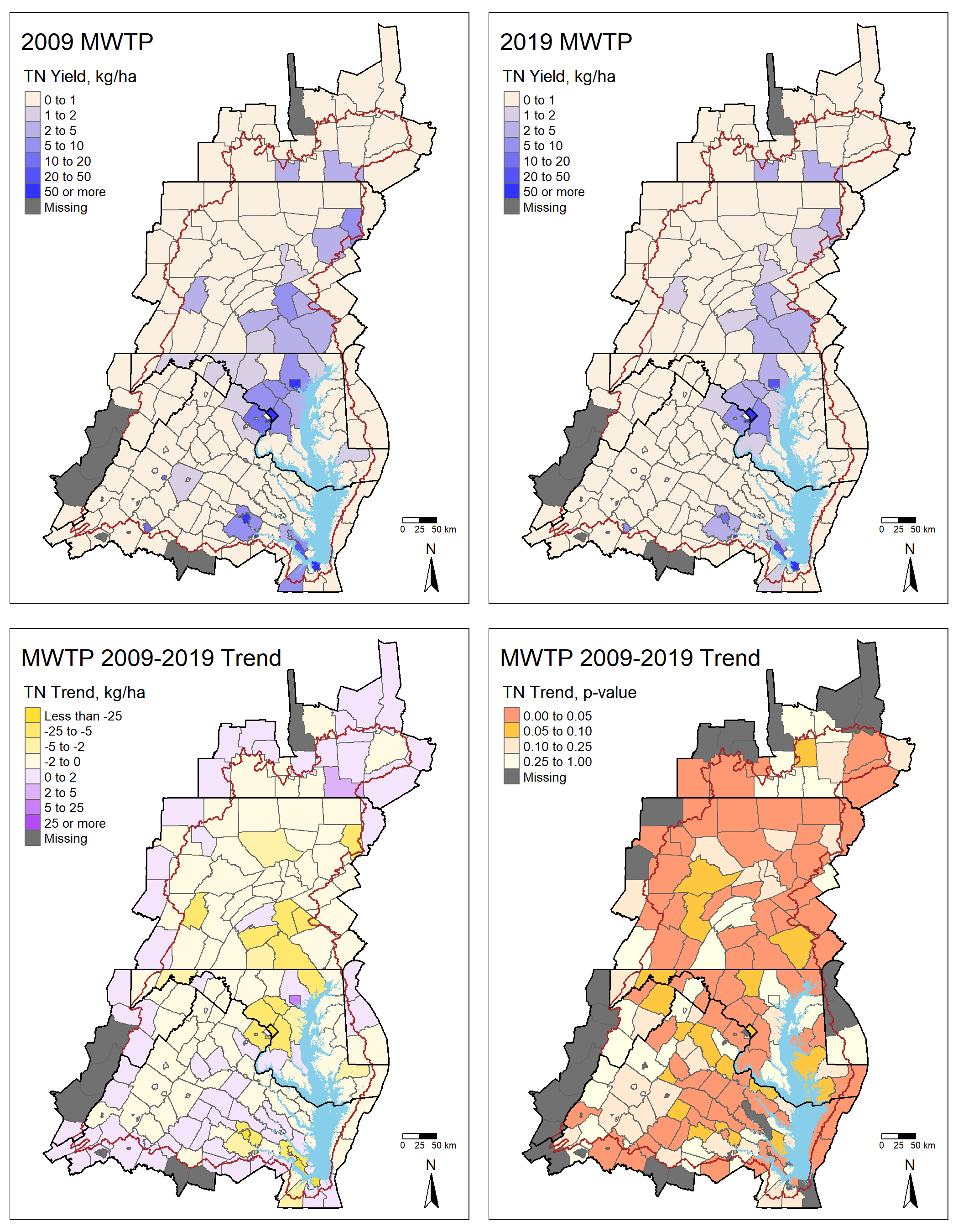
 Figure S24. For nitrogen, 2009 and 2019 municipal wastewater treatment plant load (top row), the estimated Sen linear slope change in municipal wastewater treatment plant load from 2009-2019 (bottom left), and the significance of trend results by county (bottom right).
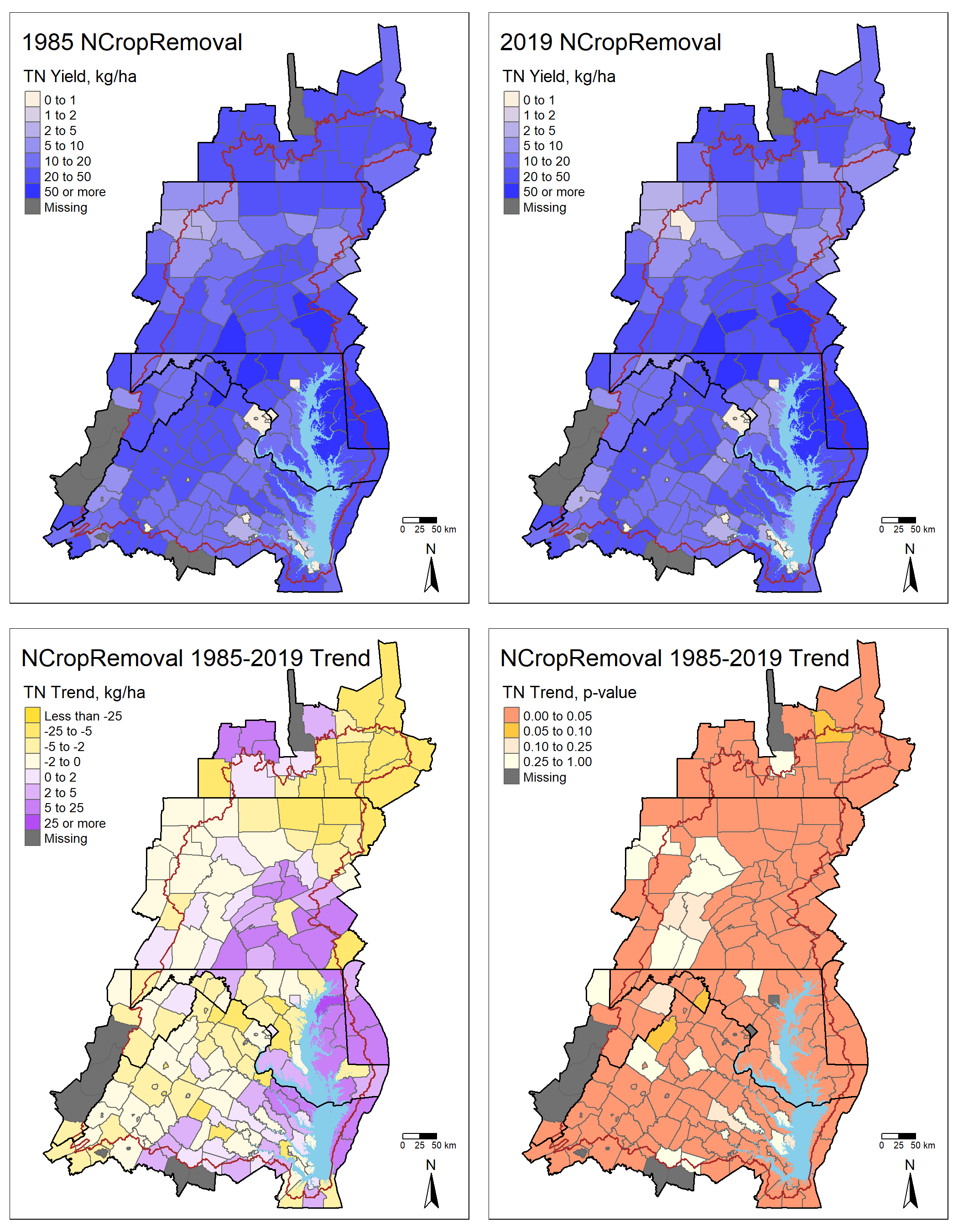
 Figure S25. For nitrogen, 1985 and 2019 crop nitrogen removal (top row), the estimated Sen linear slope change in crop nitrogen removal from 1985-2019 (bottom left), and the significance of trend results by county (bottom right).
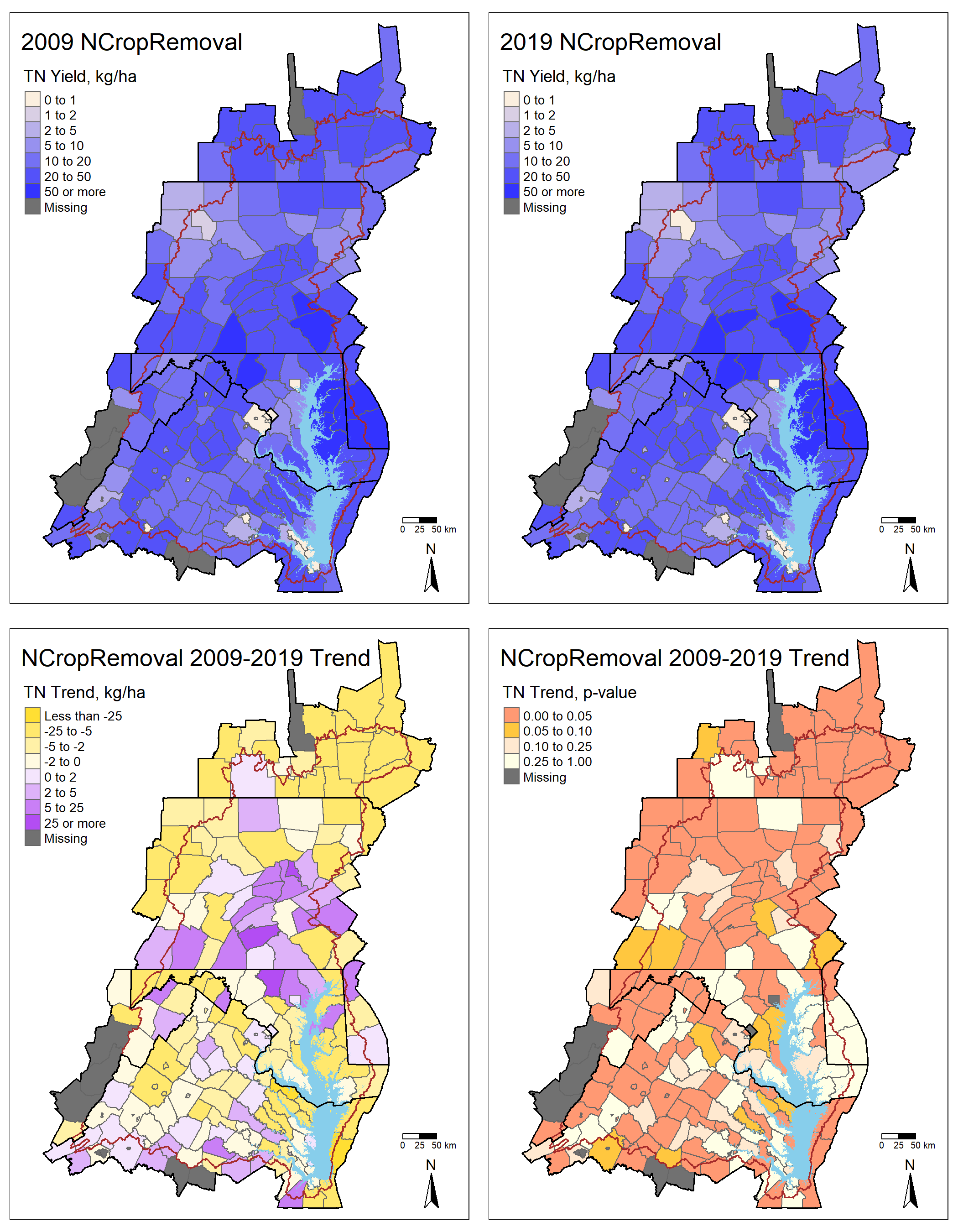
 Figure S26. For nitrogen, 2009 and 2019 crop nitrogen removal (top row), the estimated Sen linear slope change in crop nitrogen removal from 2009-2019 (bottom left), and the significance of trend results by county (bottom right).
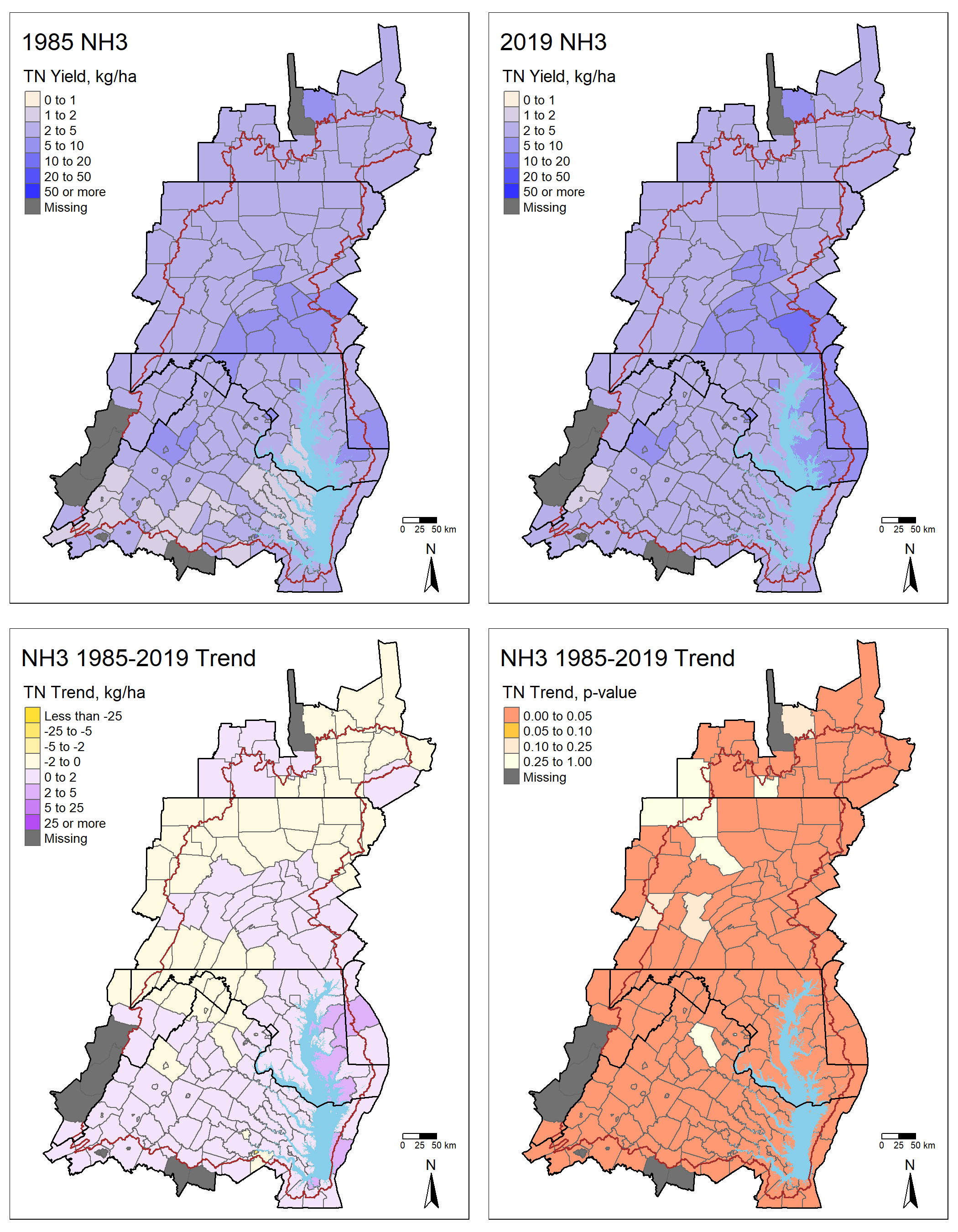
 Figure S27. 1 For nitrogen, 1985 and 2019 in reduced forms of atmospheric deposition (top row), the estimated Sen linear slope change in reduced forms of atmospheric deposition from 1985-2019 (bottom left), and the significance of trend results by county (bottom right).
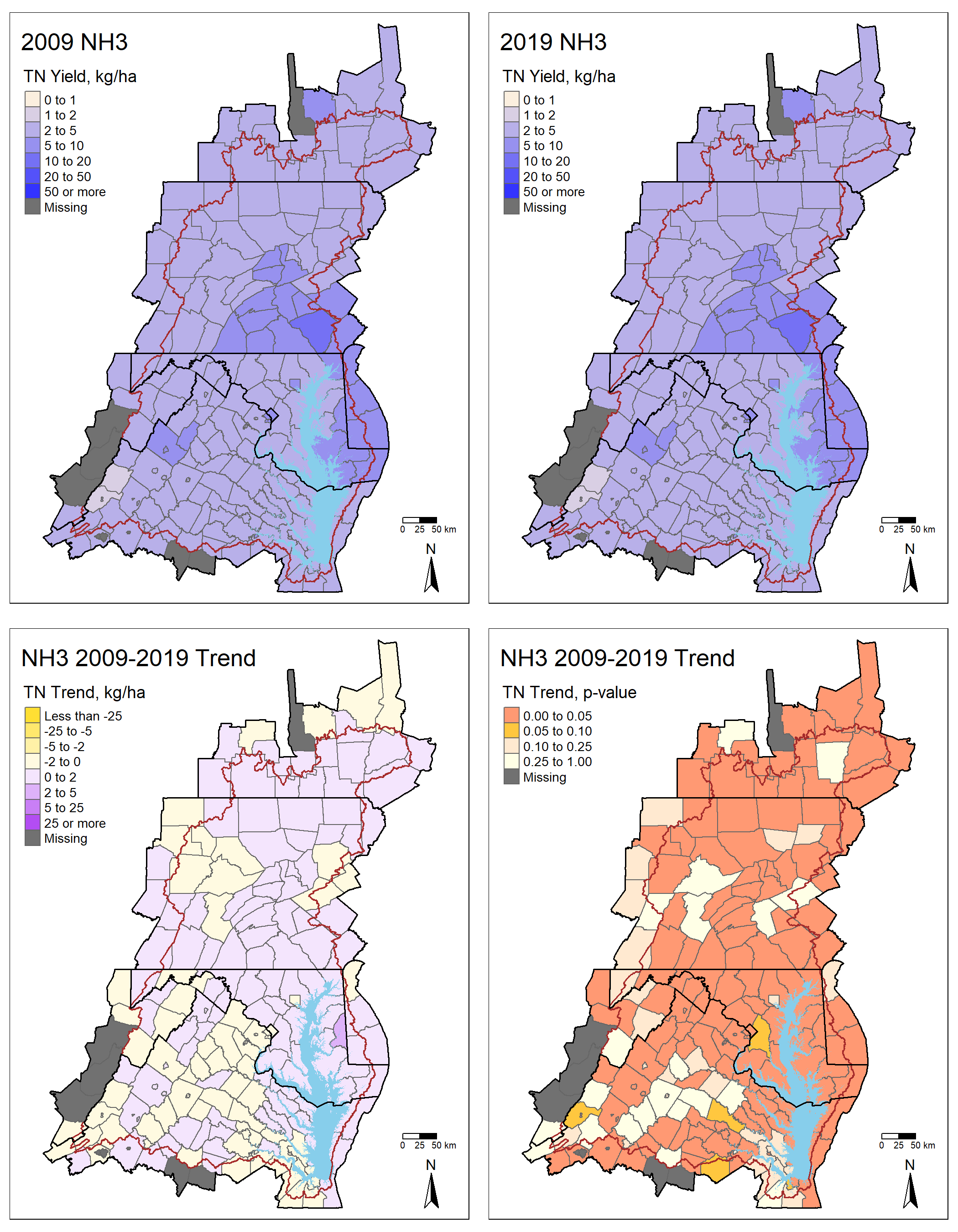
 Figure S28. For nitrogen, 2009 and 2019 reduced forms of atmospheric deposition (top row), the estimated Sen linear slope change in reduced forms of atmospheric deposition from 2009-2019 (bottom left), and the significance of trend results by county (bottom right).
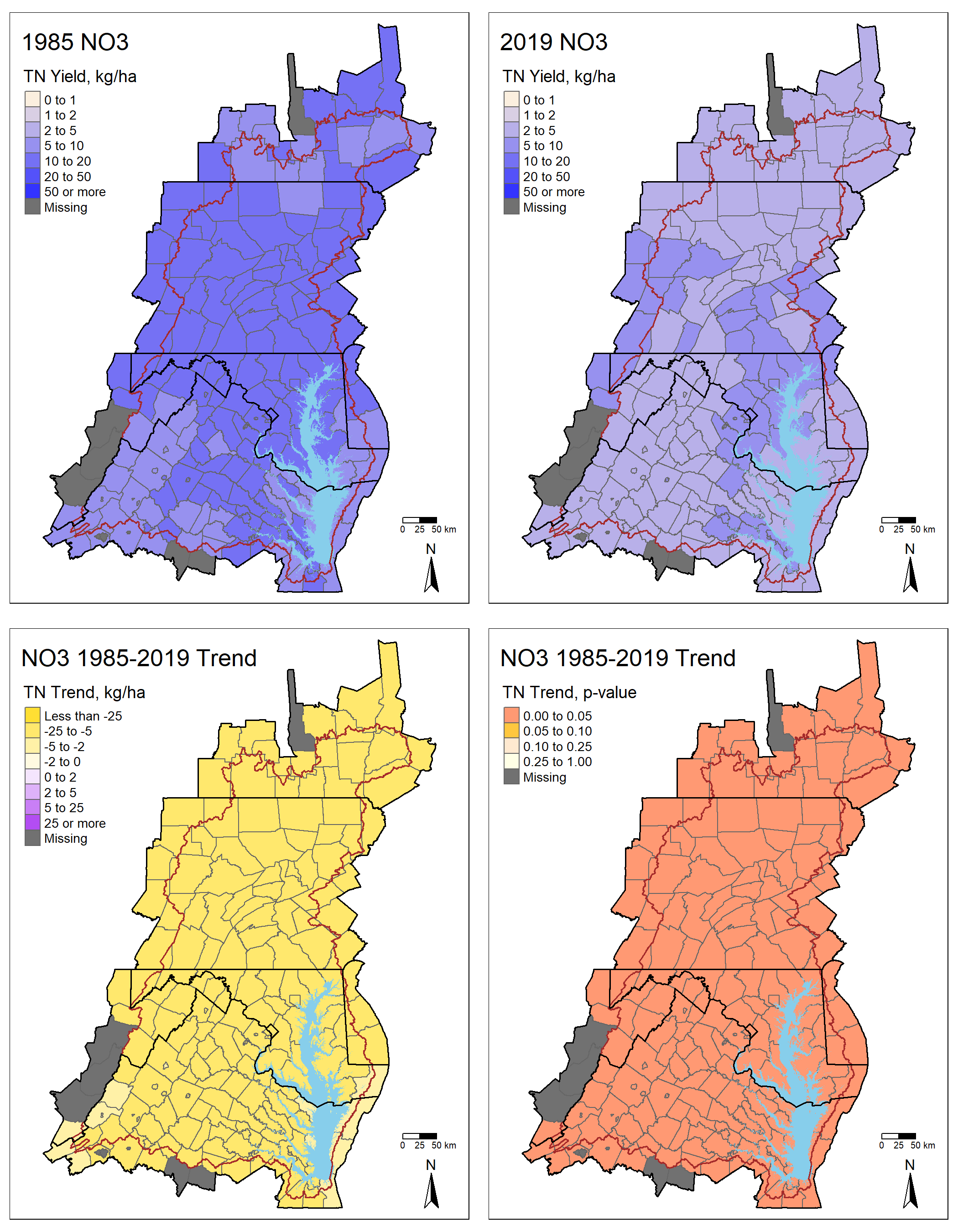
 Figure S29. For nitrogen, 1985 and 2019 oxidized forms of atmospheric deposition (top row), the estimated Sen linear slope change in oxidized forms of atmospheric deposition from 1985-2019 (bottom left), and the significance of trend results by county (bottom right).
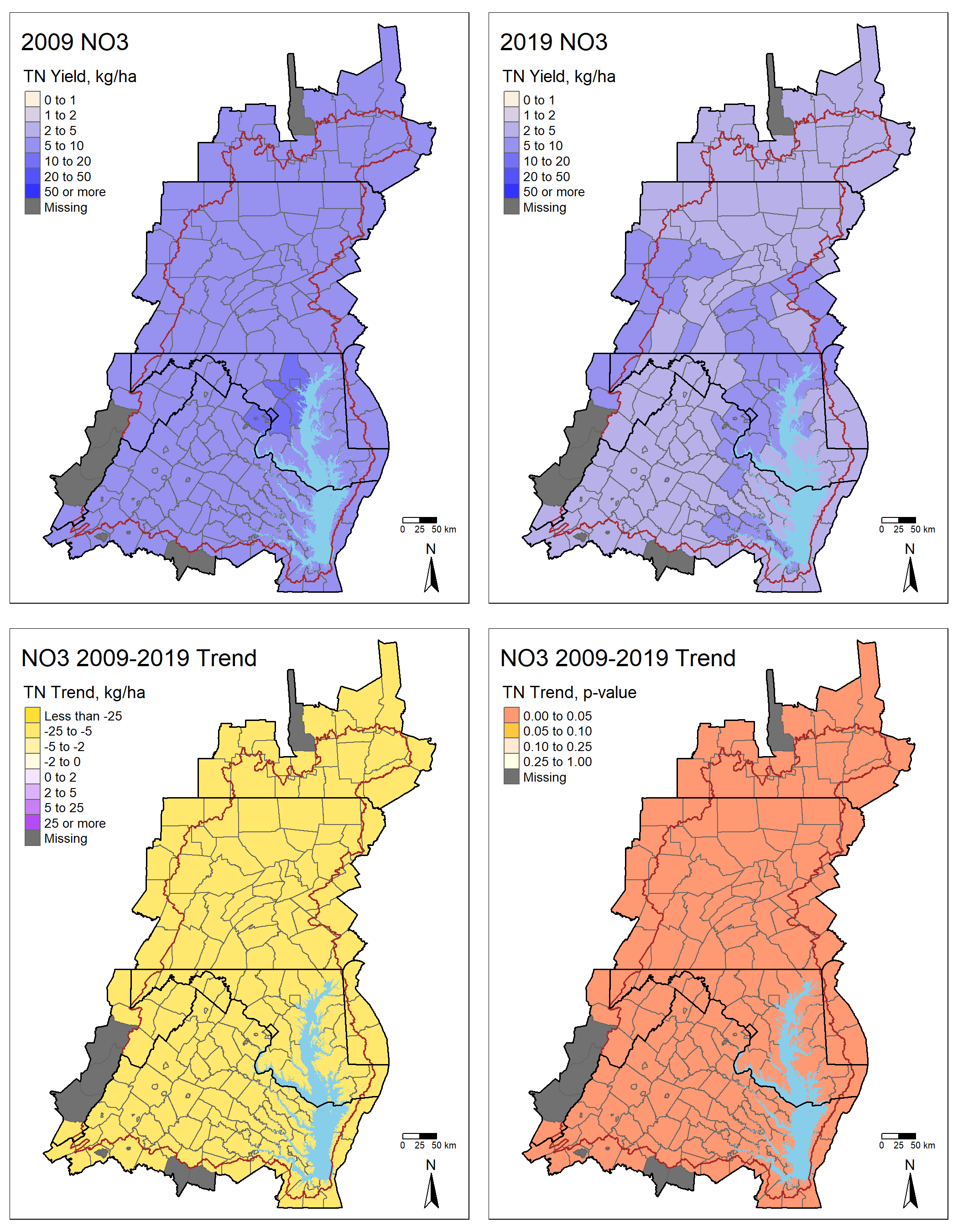
 Figure S30. For nitrogen, 2009 and 2019 oxidized forms of atmospheric deposition (top row), the estimated Sen linear slope change in oxidized forms of atmospheric deposition from 2009-2019 (bottom left), and the significance of trend results by county (bottom right).
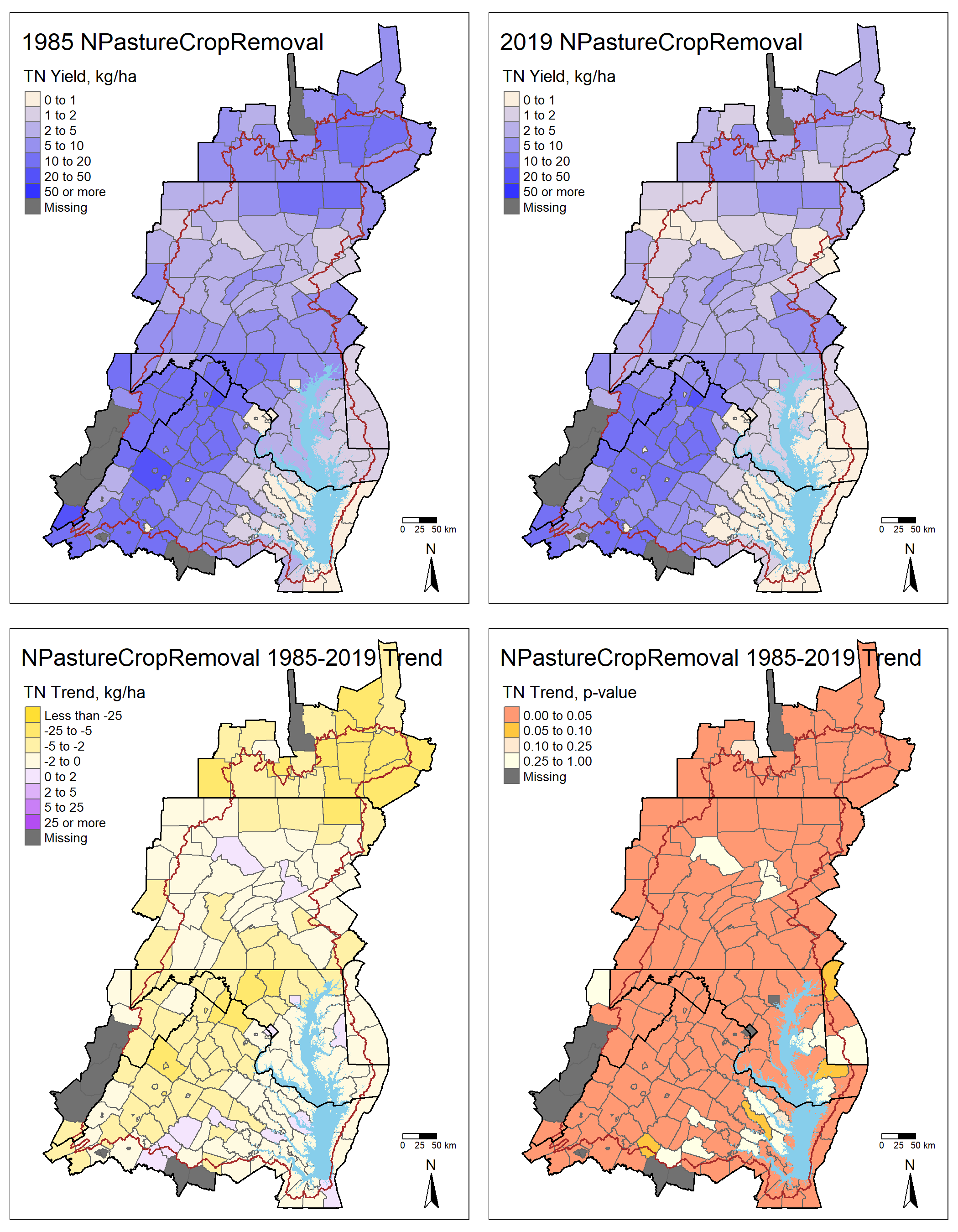
Figure S31. For nitrogen, 1985 and 2019 pasture nitrogen removal (top row), the estimated Sen linear slope change in pasture nitrogen removal from 1985-2019 (bottom left), and the significance of trend results by county (bottom right).
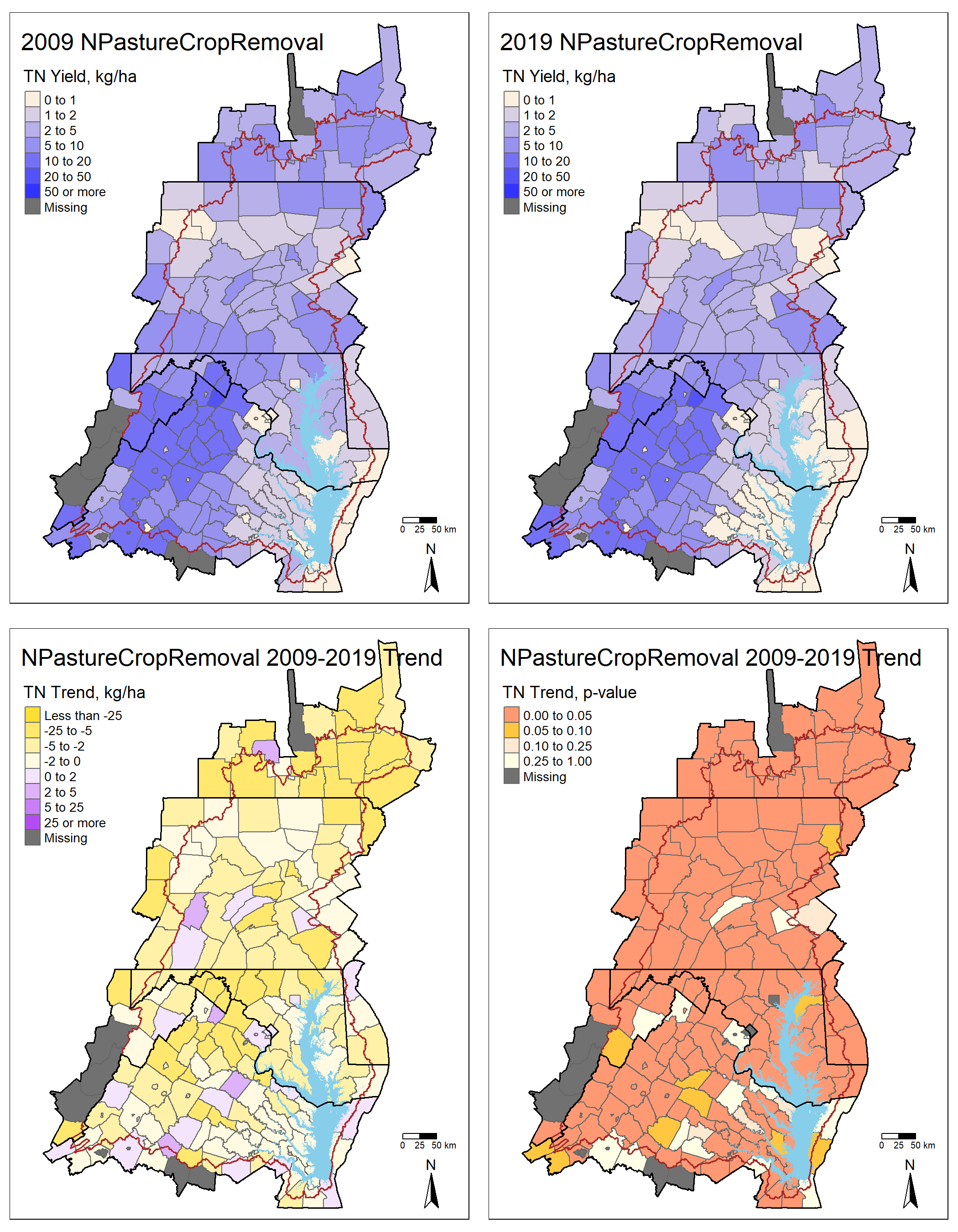
Figure S32. For nitrogen, 2009 and 2019 pasture nitrogen removal (top row), the estimated Sen linear slope change in pasture nitrogen removal from 2009-2019 (bottom left), and the significance of trend results by county (bottom right).
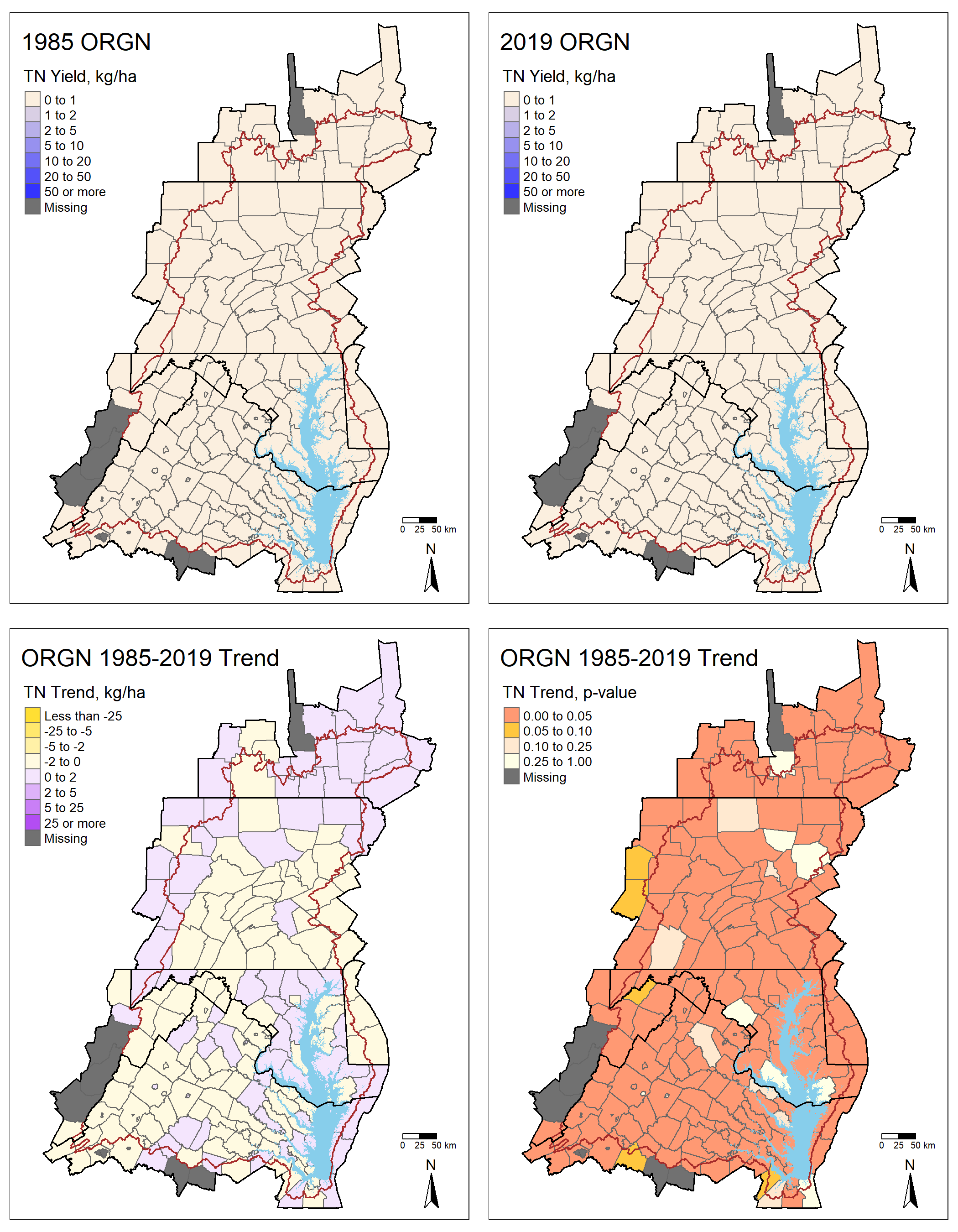
 Figure S33. For nitrogen, 1985 and 2019 atmospheric organic nitrogen deposition (top row), the estimated Sen linear slope change in atmospheric organic nitrogen deposition from 1985-2019 (bottom left), and the significance of trend results by county (bottom right).
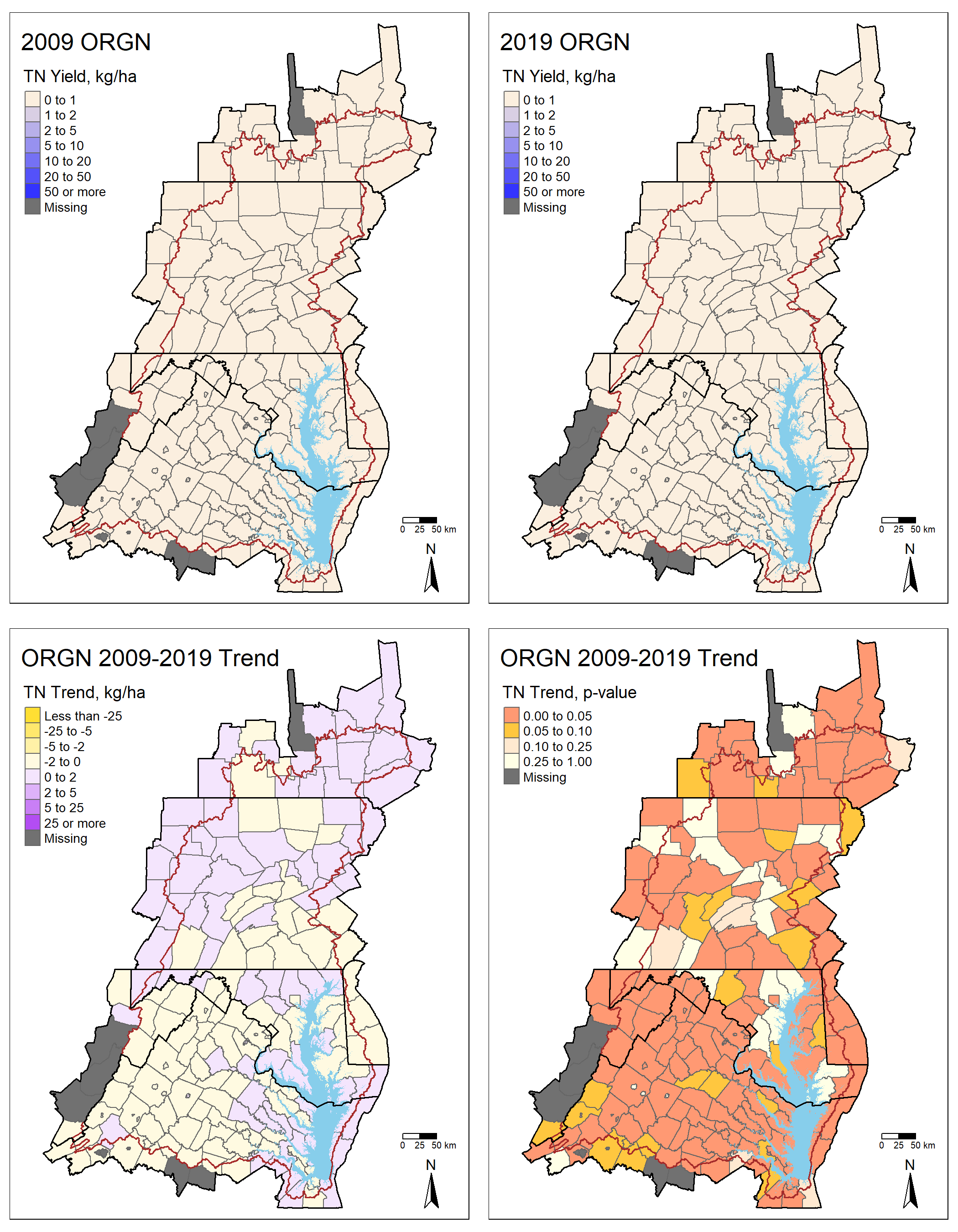
 Figure S34. For nitrogen, 2009 and 2019 atmospheric organic nitrogen deposition (top row), the estimated Sen linear slope change in atmospheric organic nitrogen deposition from 2009-2019 (bottom left), and the significance of trend results by county (bottom right).
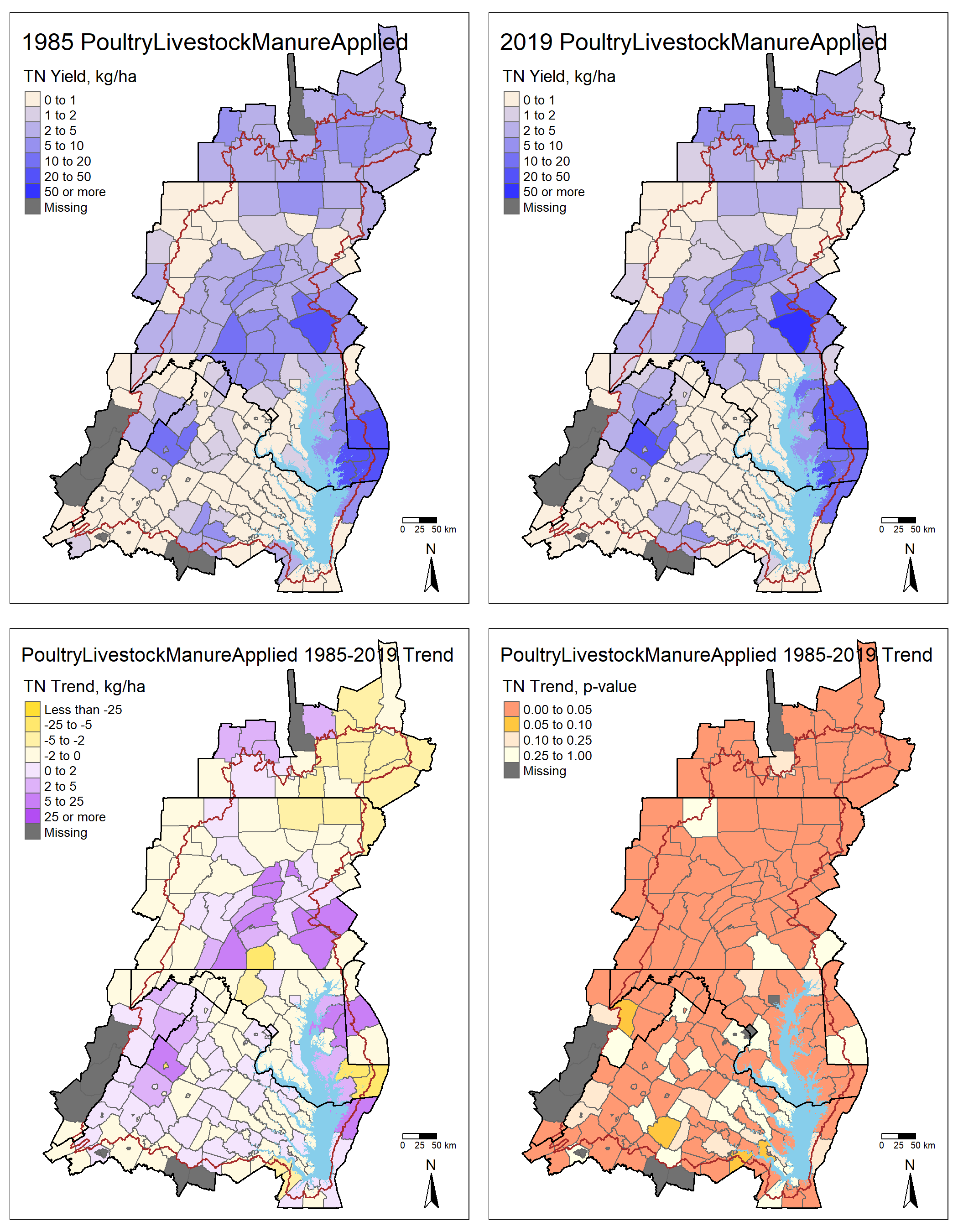
 Figure S35. For nitrogen, 1985 and 2019 poultry and livestock manure applied to agricultural land (top row), the estimated Sen linear slope change in poultry and livestock manure applied to agricultural land from 1985-2019 (bottom left), and the significance of trend results by county (bottom right).
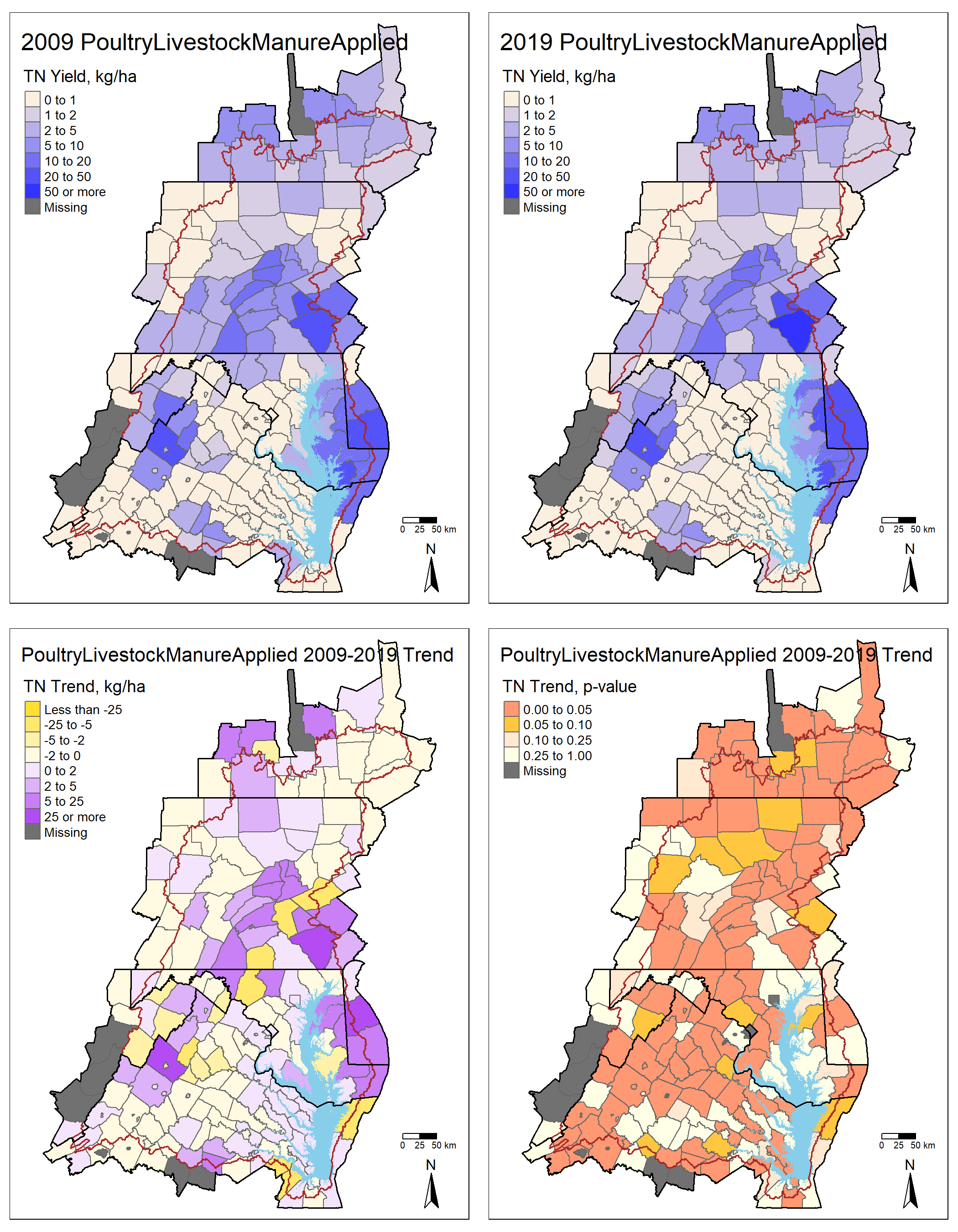
 Figure S36. For nitrogen, 2009 and 2019 poultry and livestock manure applied to agricultural land (top row), the estimated Sen linear slope change in poultry and livestock manure applied to agricultural land from 2009-2019 (bottom left), and the significance of trend results by county (bottom right).
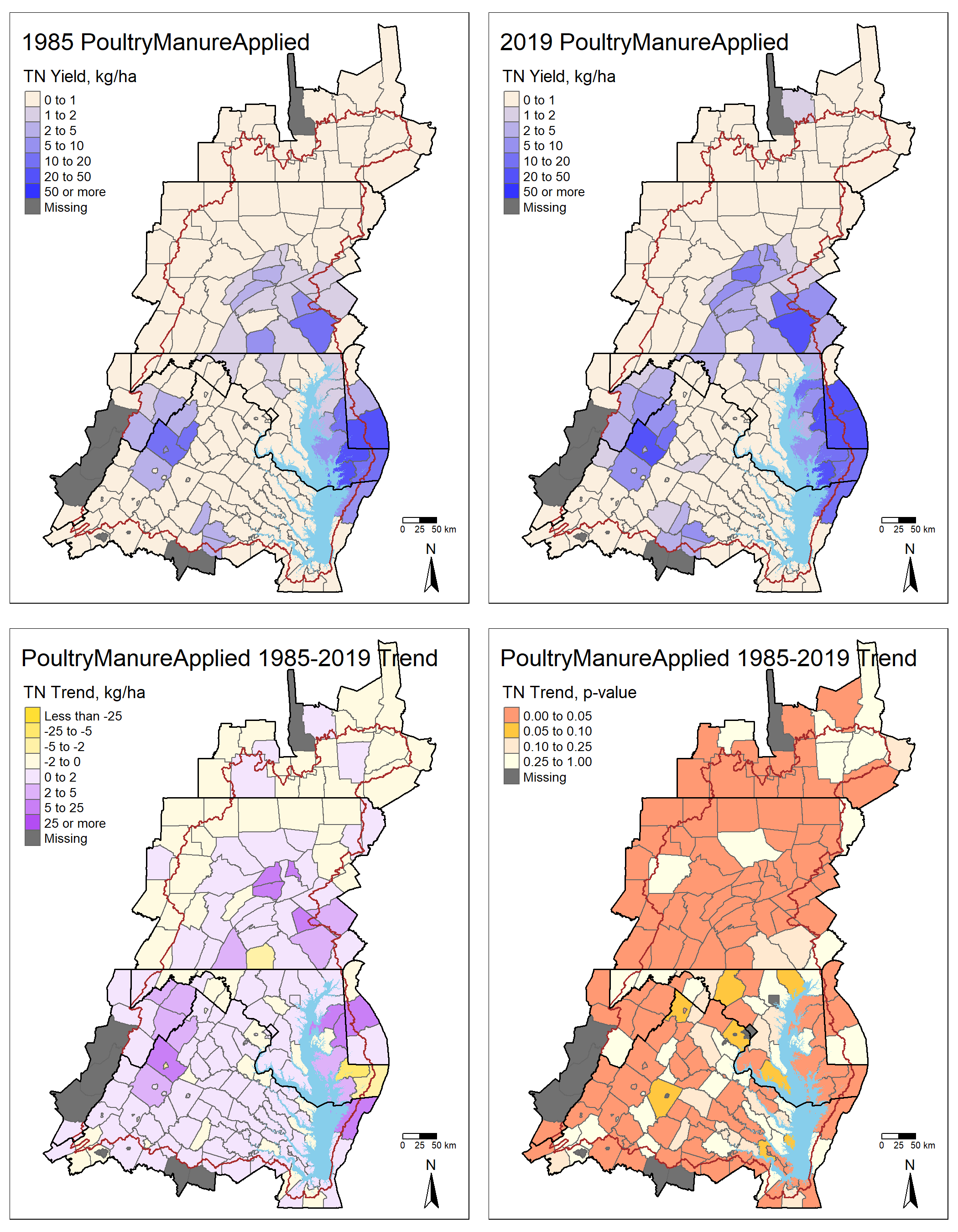
 Figure S37. For nitrogen, 1985 and 2019 poultry manure applied to agricultural land (top row), the estimated Sen linear slope change in poultry manure applied to agricultural land from 1985-2019 (bottom left), and the significance of trend results by county (bottom right).
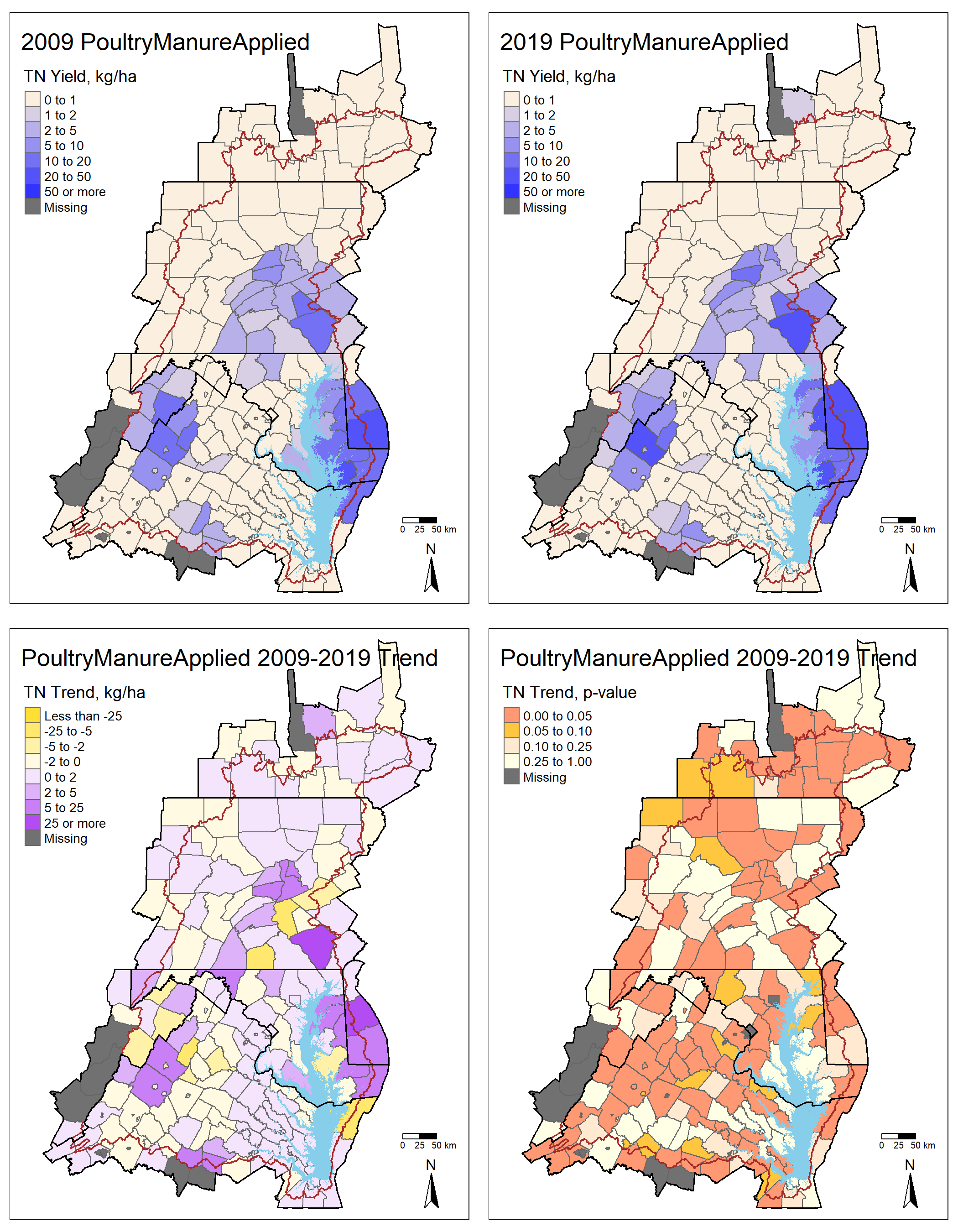
 Figure S38. For nitrogen, 2009 and 2019 poultry manure applied to agricultural land (top row), the estimated Sen linear slope change in poultry manure applied to agricultural land from 2009-2019 (bottom left), and the significance of trend results by county (bottom right).
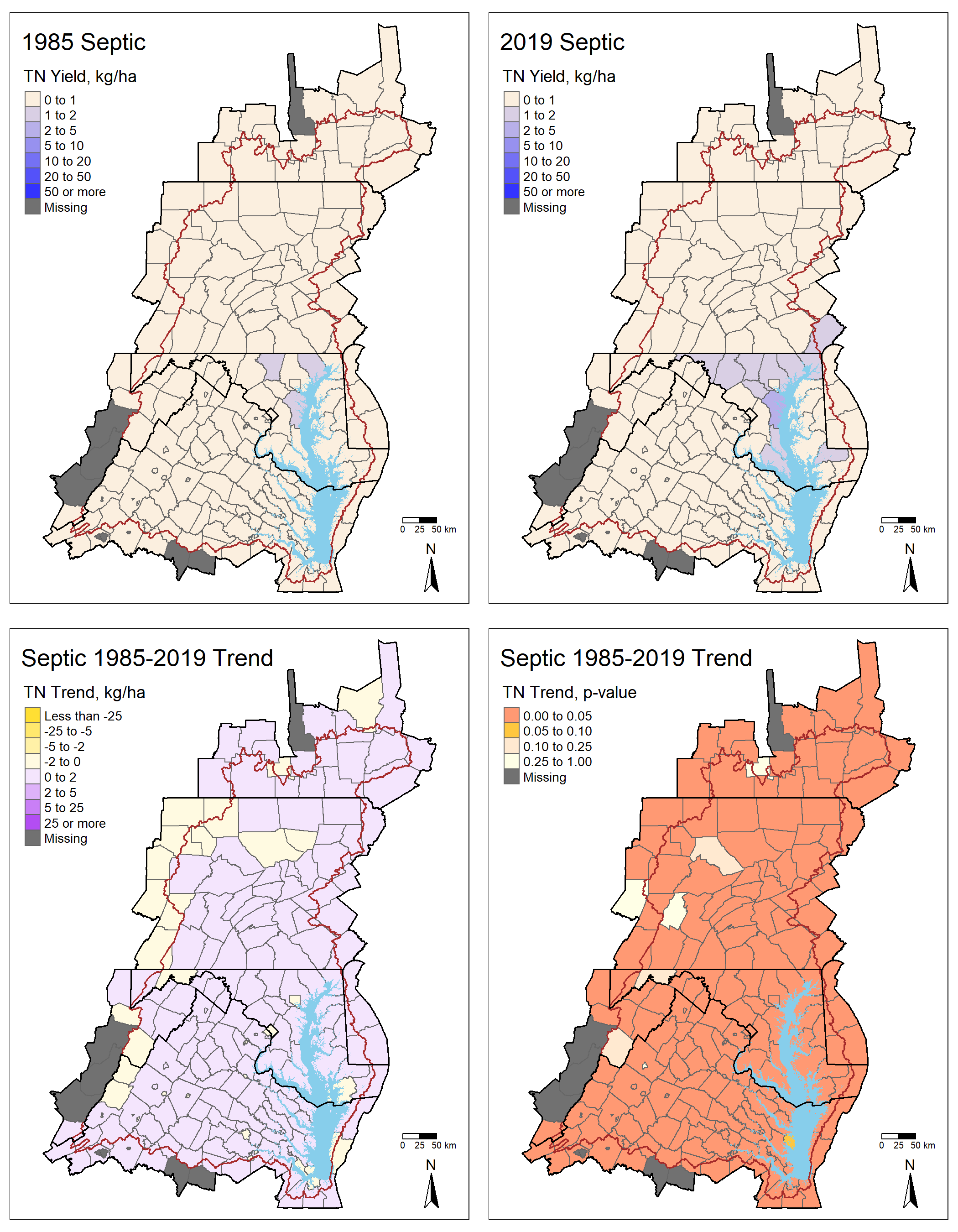
 Figure S39. For nitrogen, 1985 and 2019 septic loads (top row), the estimated Sen linear slope change in septic loads from 1985-2019 (bottom left), and the significance of trend results by county (bottom right).
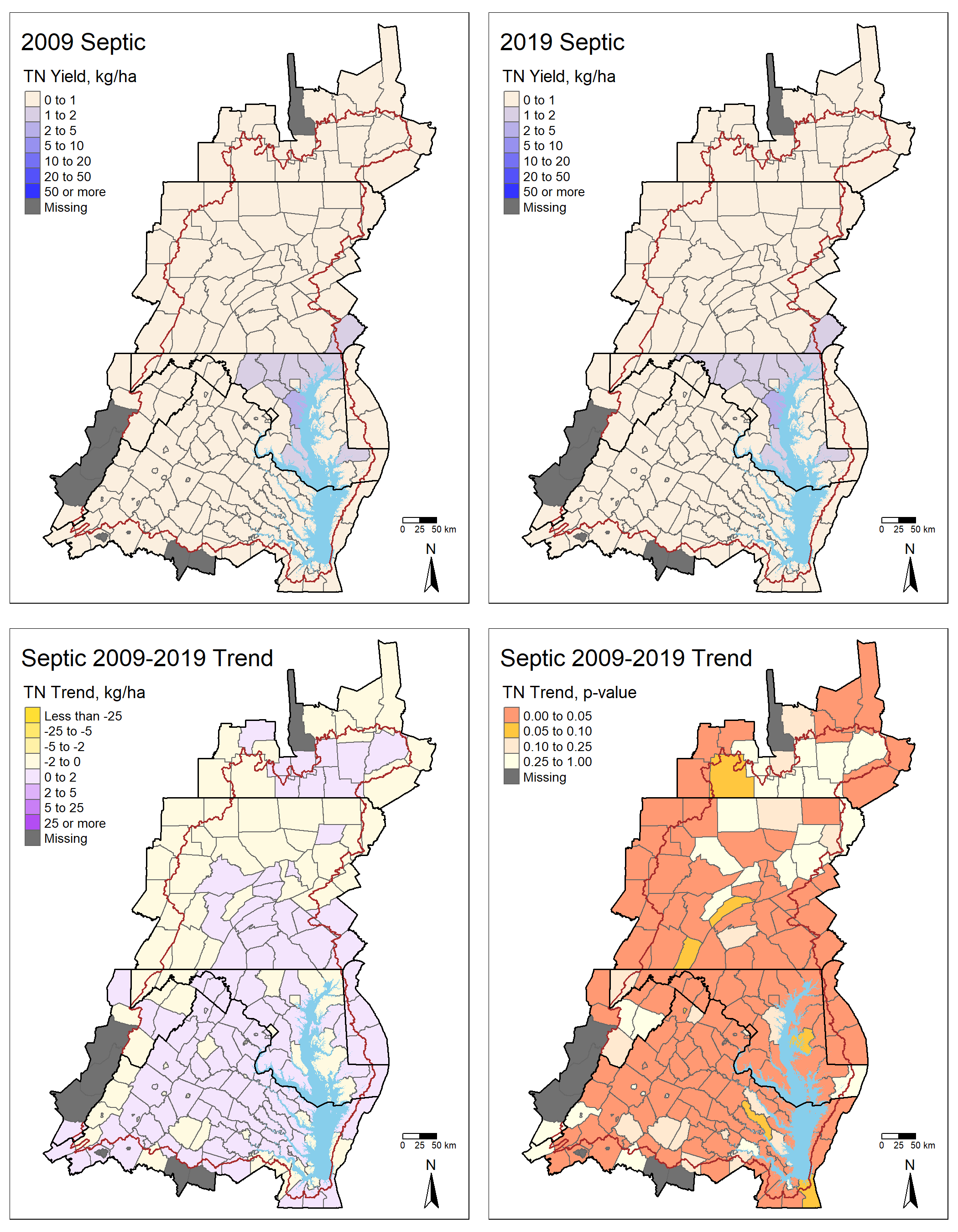
 Figure S40. For nitrogen, 2009 and 2019 septic loads (top row), the estimated Sen linear slope change in septic loads from 2009-2019 (bottom left), and the significance of trend results by county (bottom right).
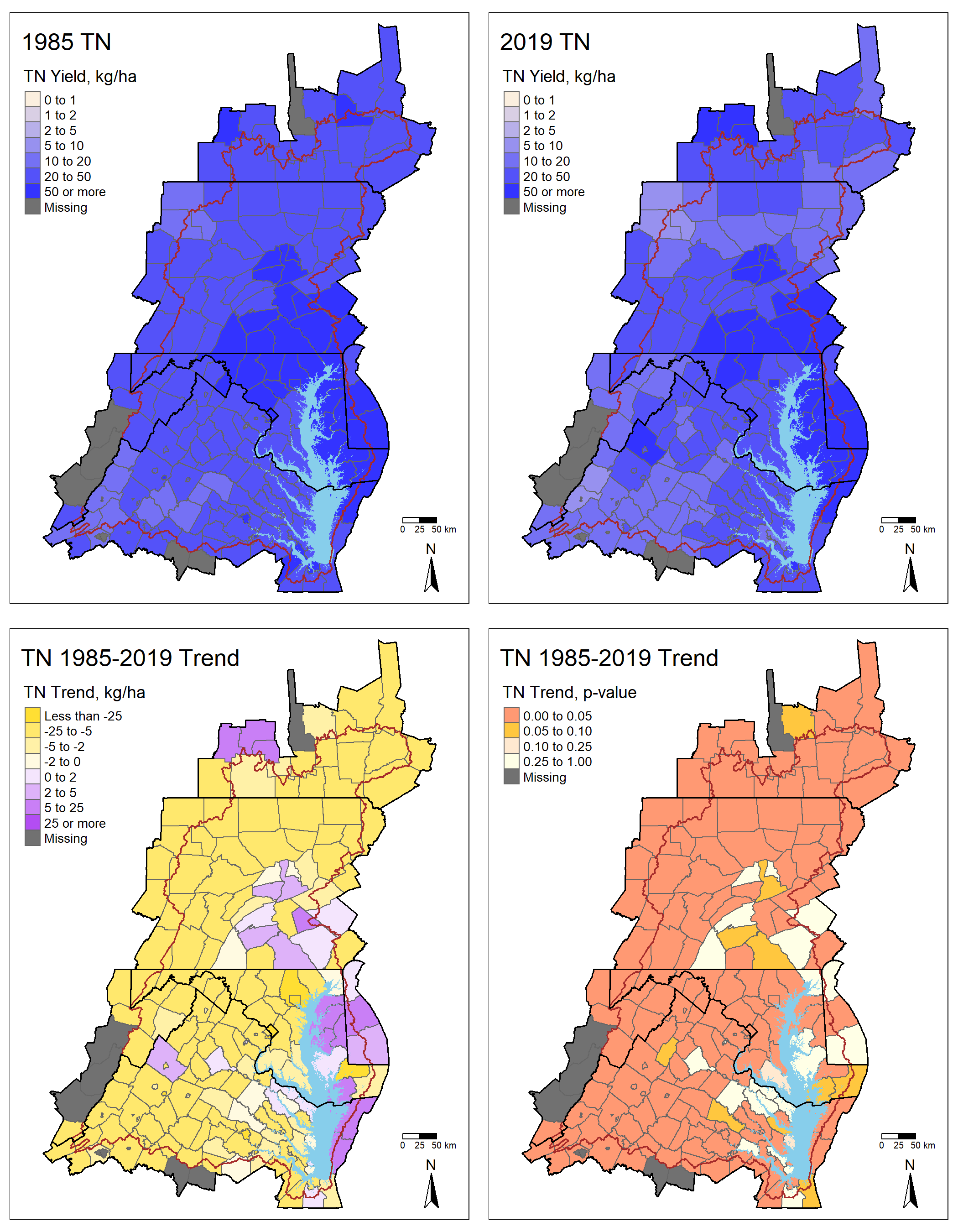
 Figure S41. For nitrogen, 1985 and 2019 total nitrogen inputs (top row), the estimated Sen linear slope change in total nitrogen inputs from 1985-2019 (bottom left), and the significance of trend results by county (bottom right).
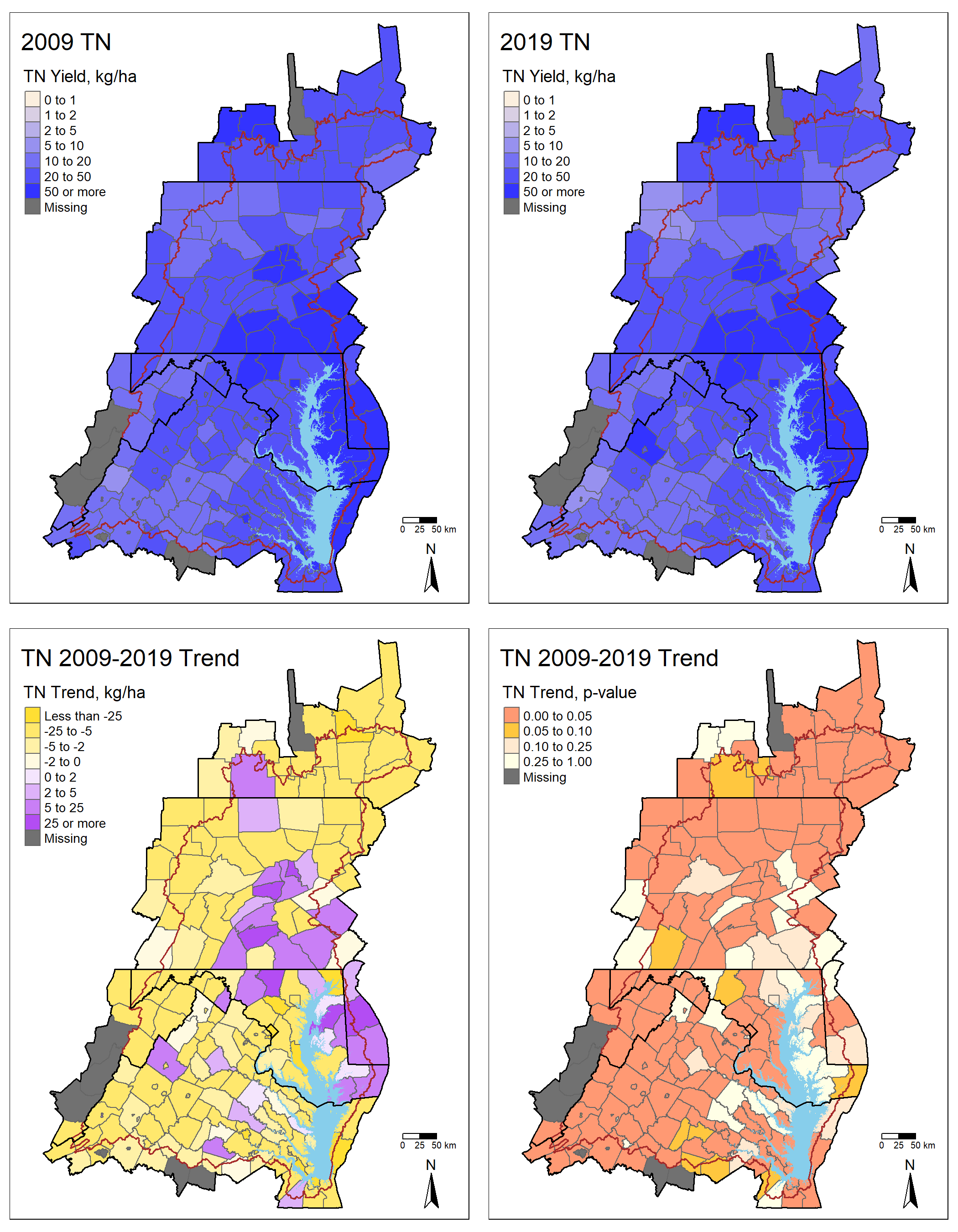
 Figure S42. For nitrogen, 2009 and 2019 total nitrogen inputs (top row), the estimated Sen linear slope change in total nitrogen inputs from 2009-2019 (bottom left), and the significance of trend results by county (bottom right).
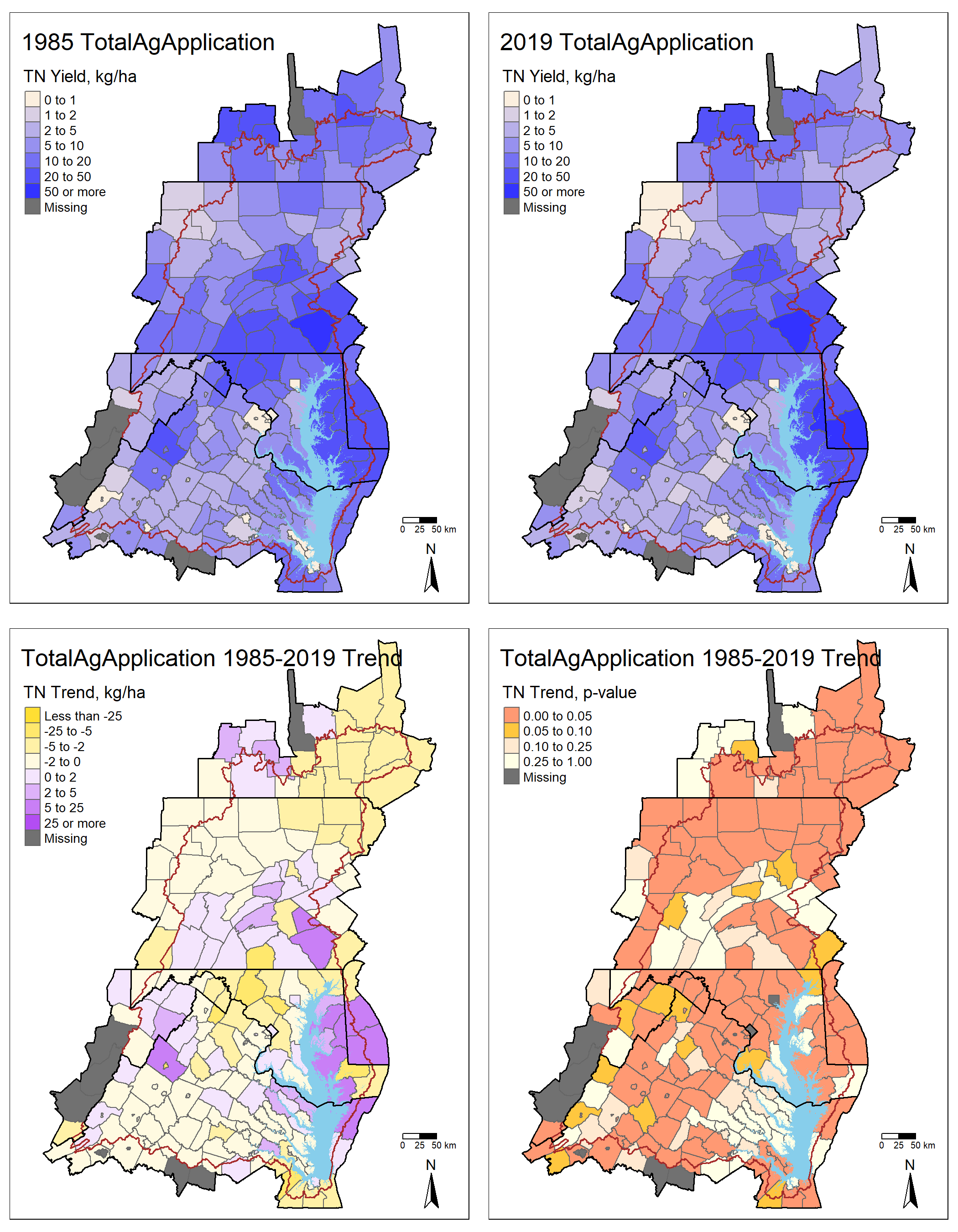
 Figure S43. For nitrogen, 1985 and 2019 total agricultural application (top row), the estimated Sen linear slope change in total agricultural application from 1985-2019 (bottom left), and the significance of trend results by county (bottom right).
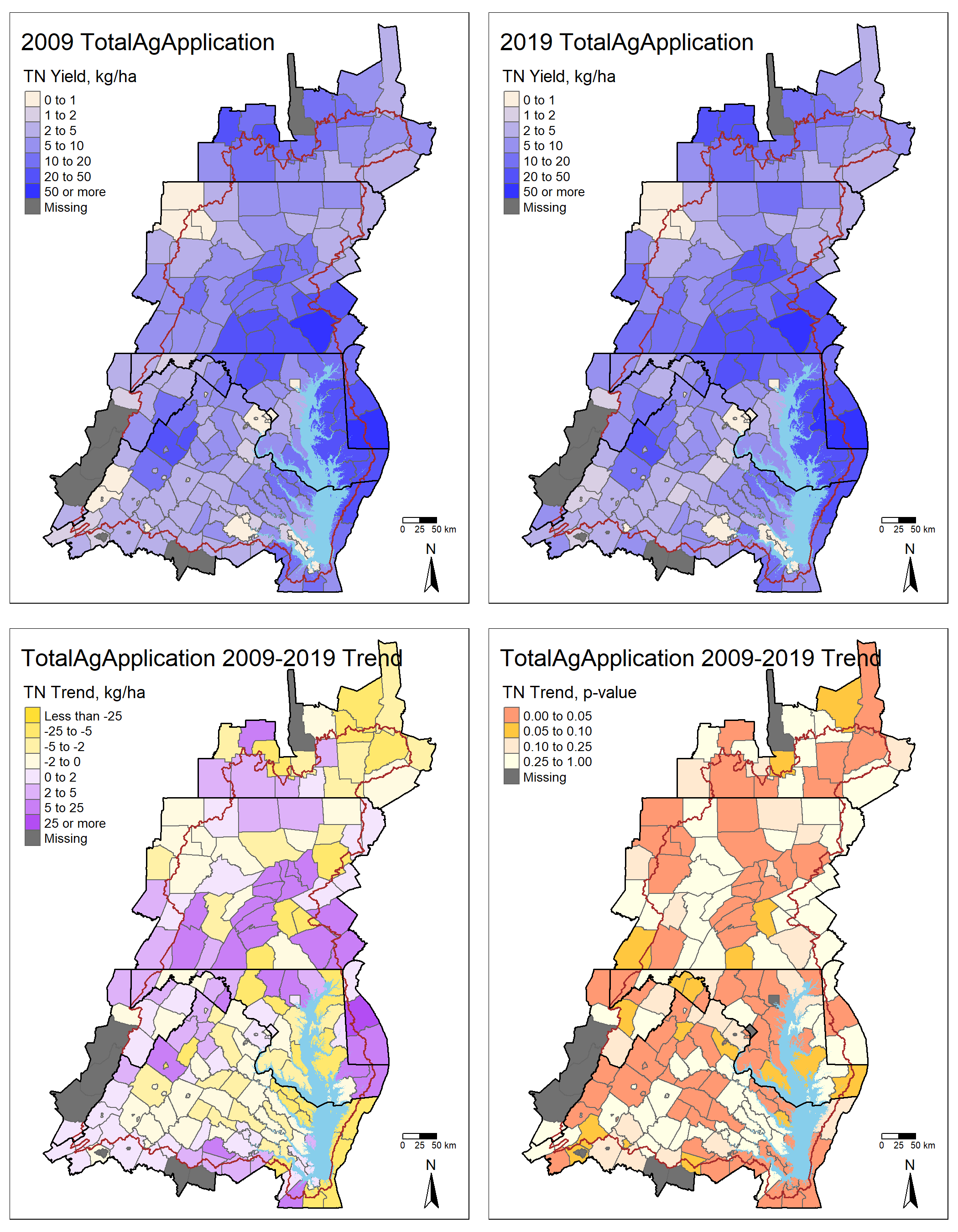
 Figure S44. For nitrogen, 2009 and 2019 total agricultural application (top row), the estimated Sen linear slope change in total agricultural application from 2009-2019 (bottom left), and the significance of trend results by county (bottom right).
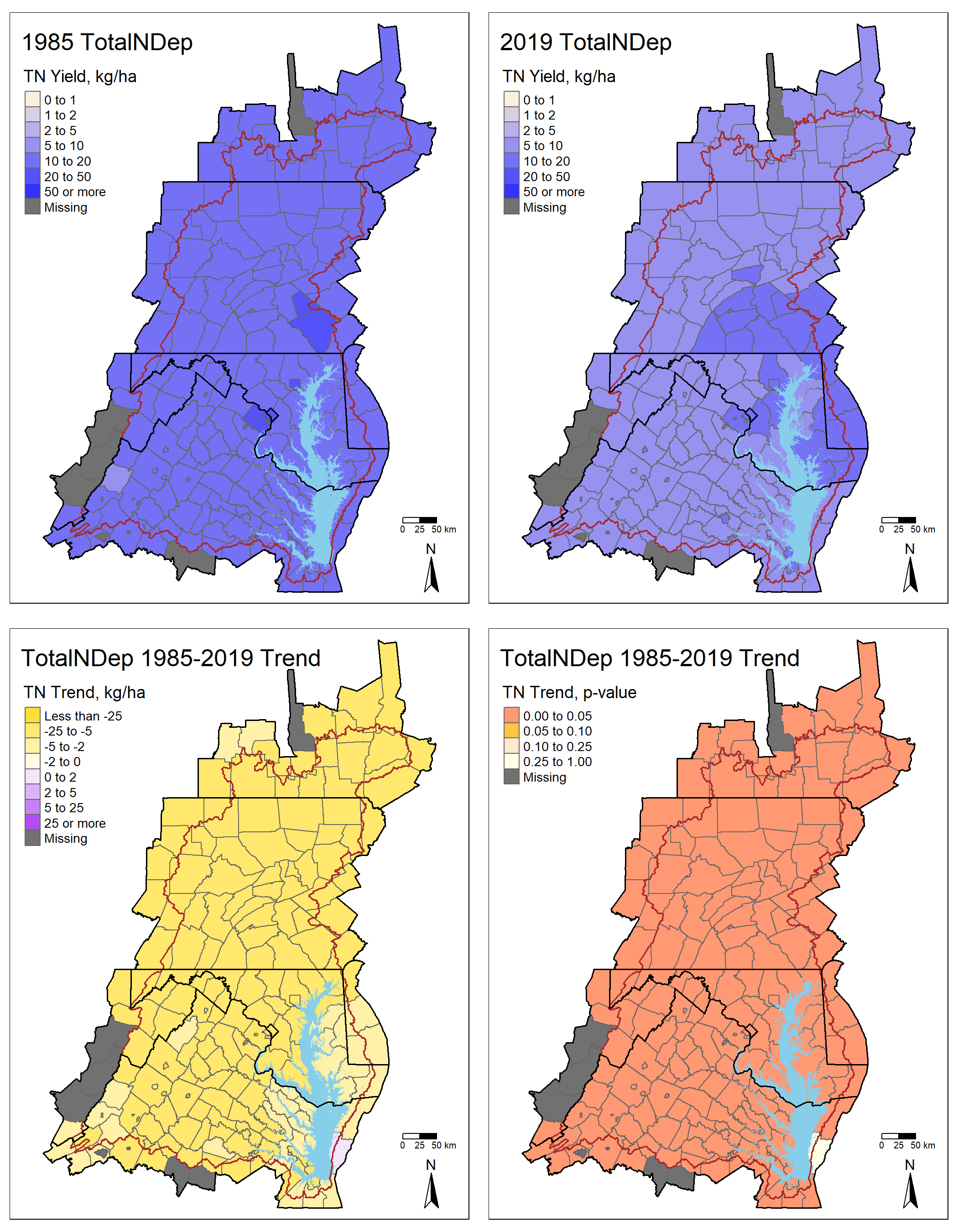
 Figure S45. For nitrogen, 1985 and 2019 total atmospheric deposition (top row), the estimated Sen linear slope change in total atmospheric deposition from 1985-2019 (bottom left), and the significance of trend results by county (bottom right).
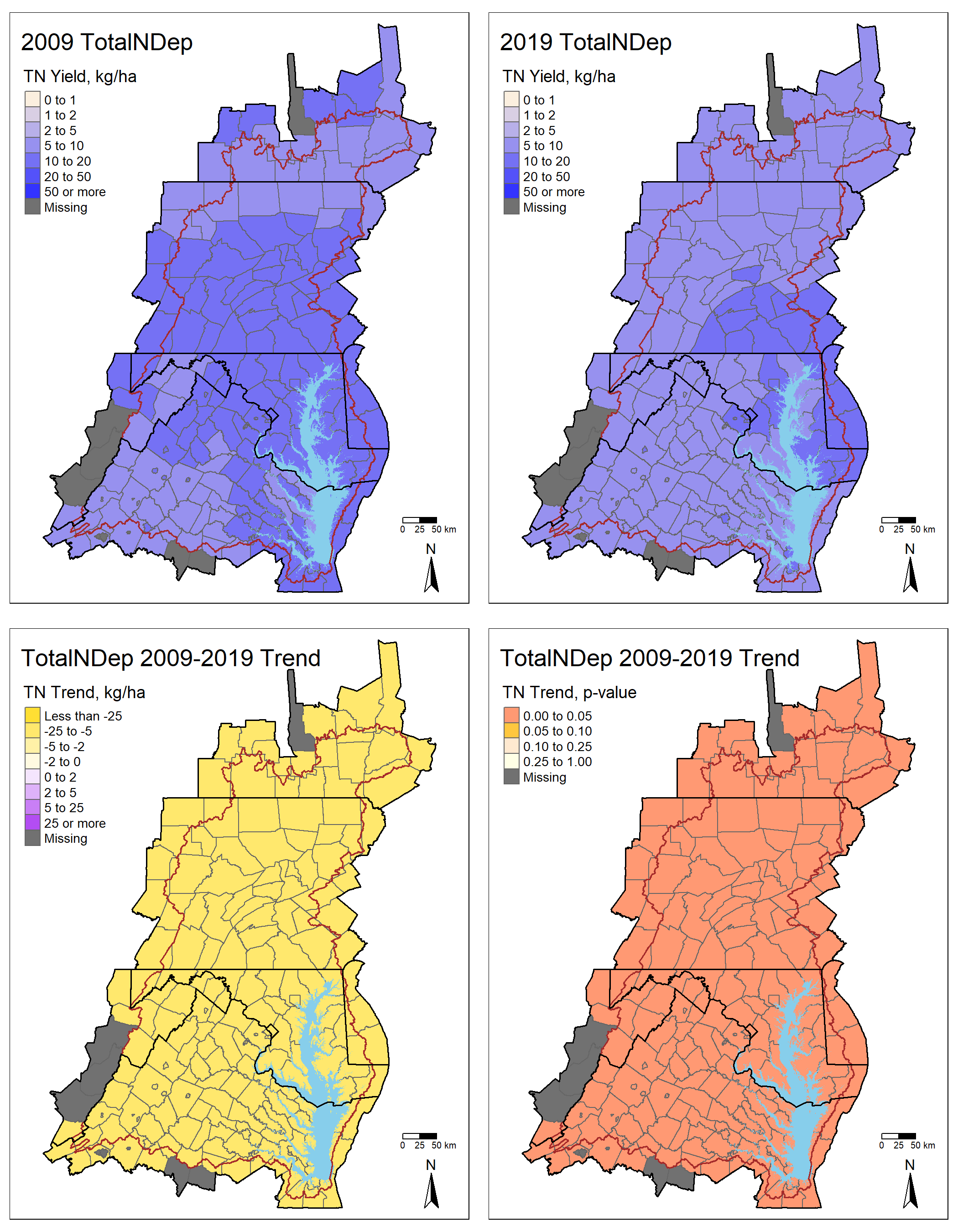
 Figure S46. For nitrogen, 2009 and 2019 total atmospheric N deposition (top row), the estimated Sen linear slope change in total atmospheric N deposition from 2009-2019 (bottom left), and the significance of trend results by county (bottom right).
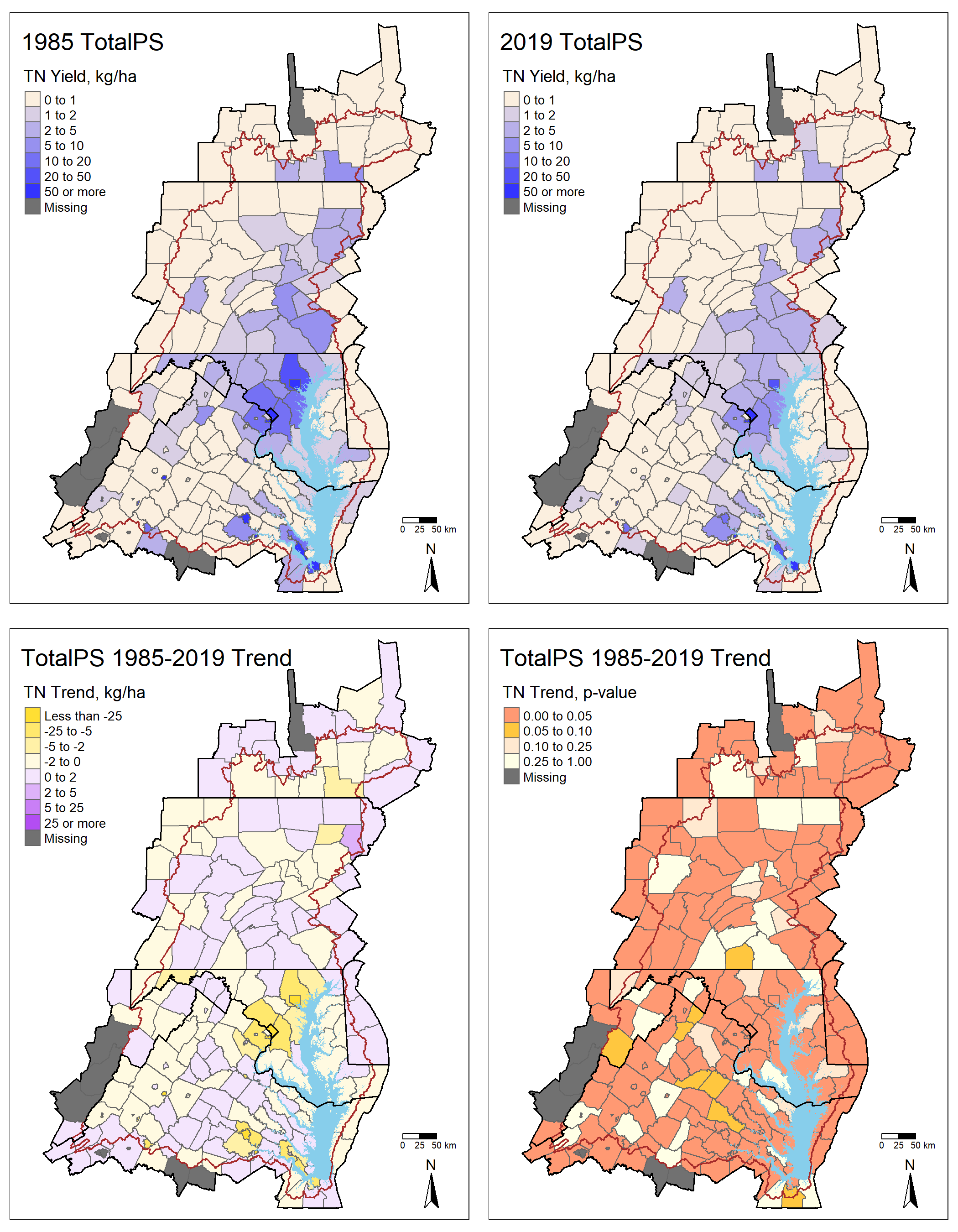
 Figure S47. For nitrogen, 1985 and 2019 total point source loads (top row), the estimated Sen linear slope change in total point source loads from 1985-2019 (bottom left), and the significance of trend results by county (bottom right).
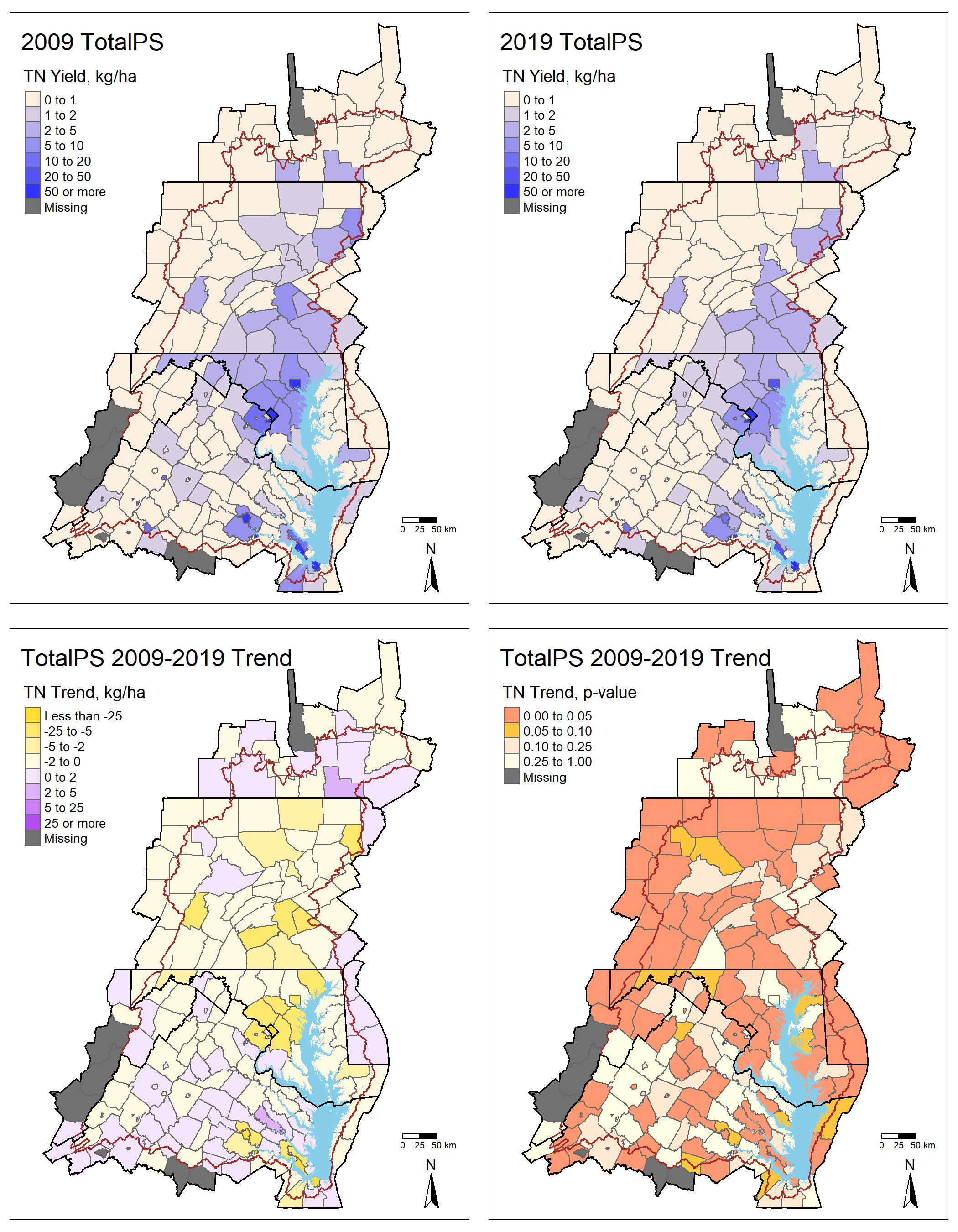
 Figure S48. For nitrogen, 2009 and 2019 total point source loads (top row), the estimated Sen linear slope change in total point source loads from 2009-2019 (bottom left), and the significance of trend results by county (bottom right).
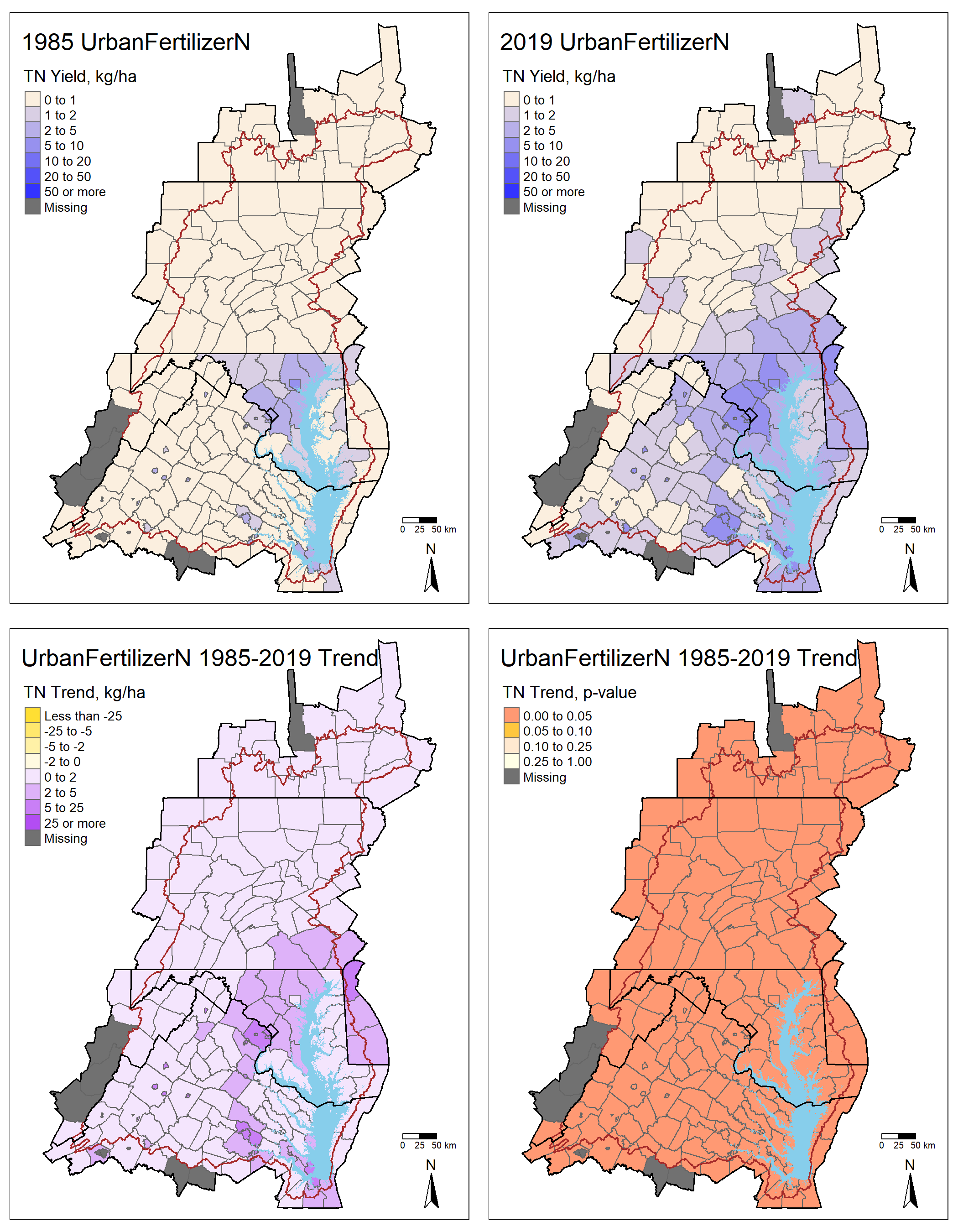
 Figure S49. For nitrogen, 1985 and 2019 urban fertilizer (top row), the estimated Sen linear slope change in urban fertilizer from 1985-2019 (bottom left), and the significance of trend results by county (bottom right).
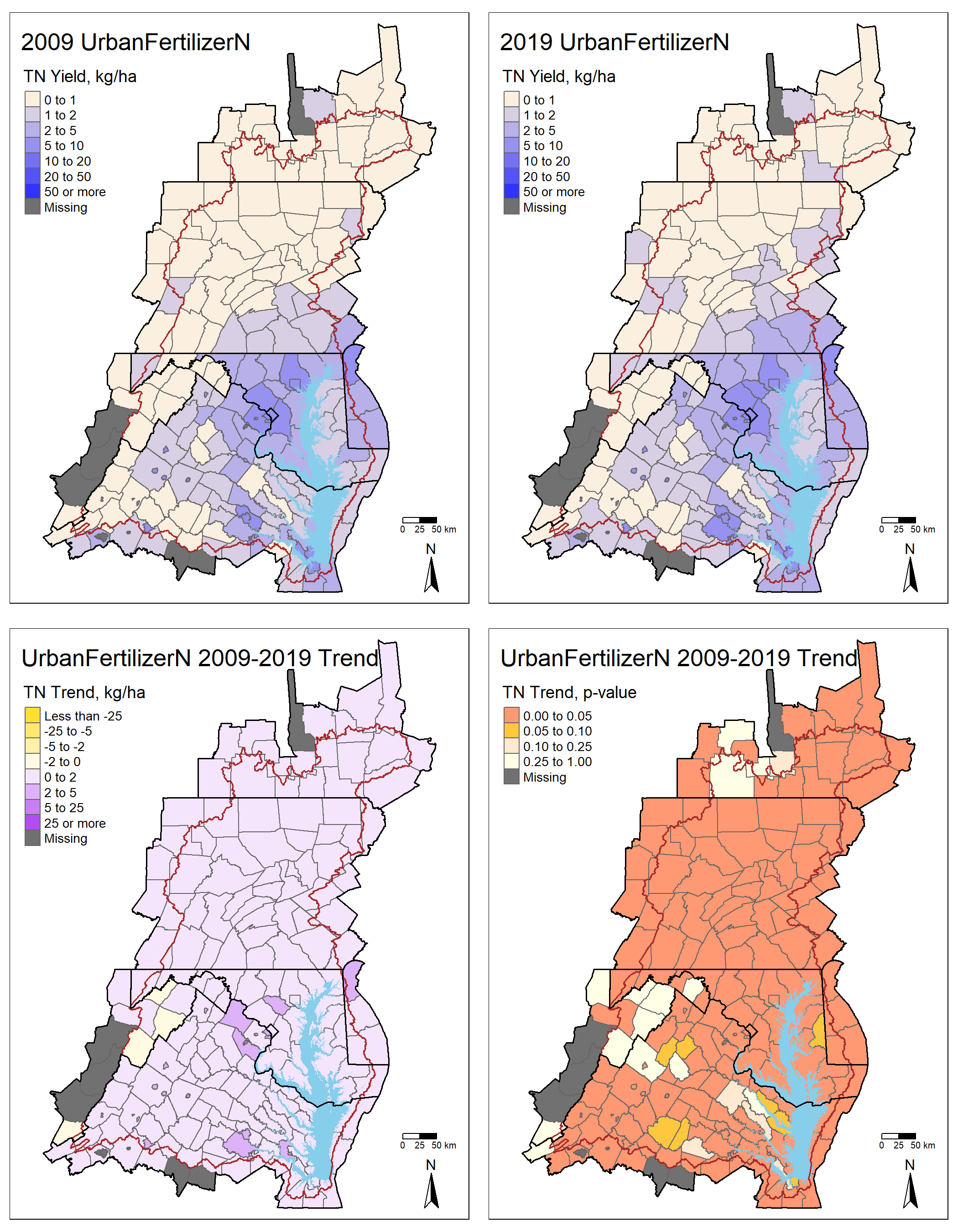
 Figure S50. For nitrogen, 2009 and 2019 urban fertilizer (top row), the estimated Sen linear slope change in urban fertilizer from 2009-2019 (bottom left), and the significance of trend results by county (bottom right).
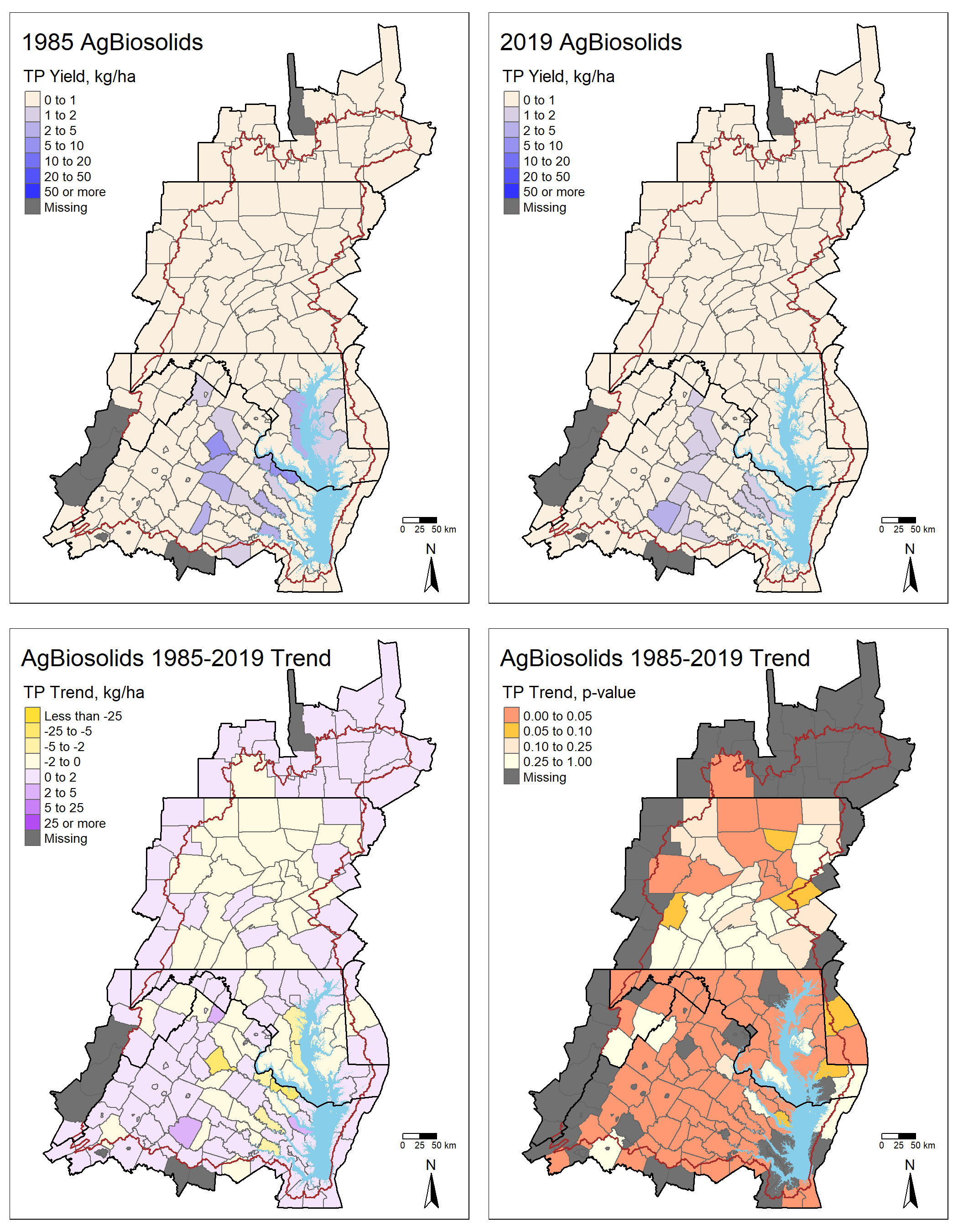
 Figure S51. For phosphorus, 1985 and 2019 biosolids applied to agricultural land (top row), the estimated Sen linear slope change in biosolid application from 1985-2019 (bottom left), and the significance of trend results by county (bottom right).
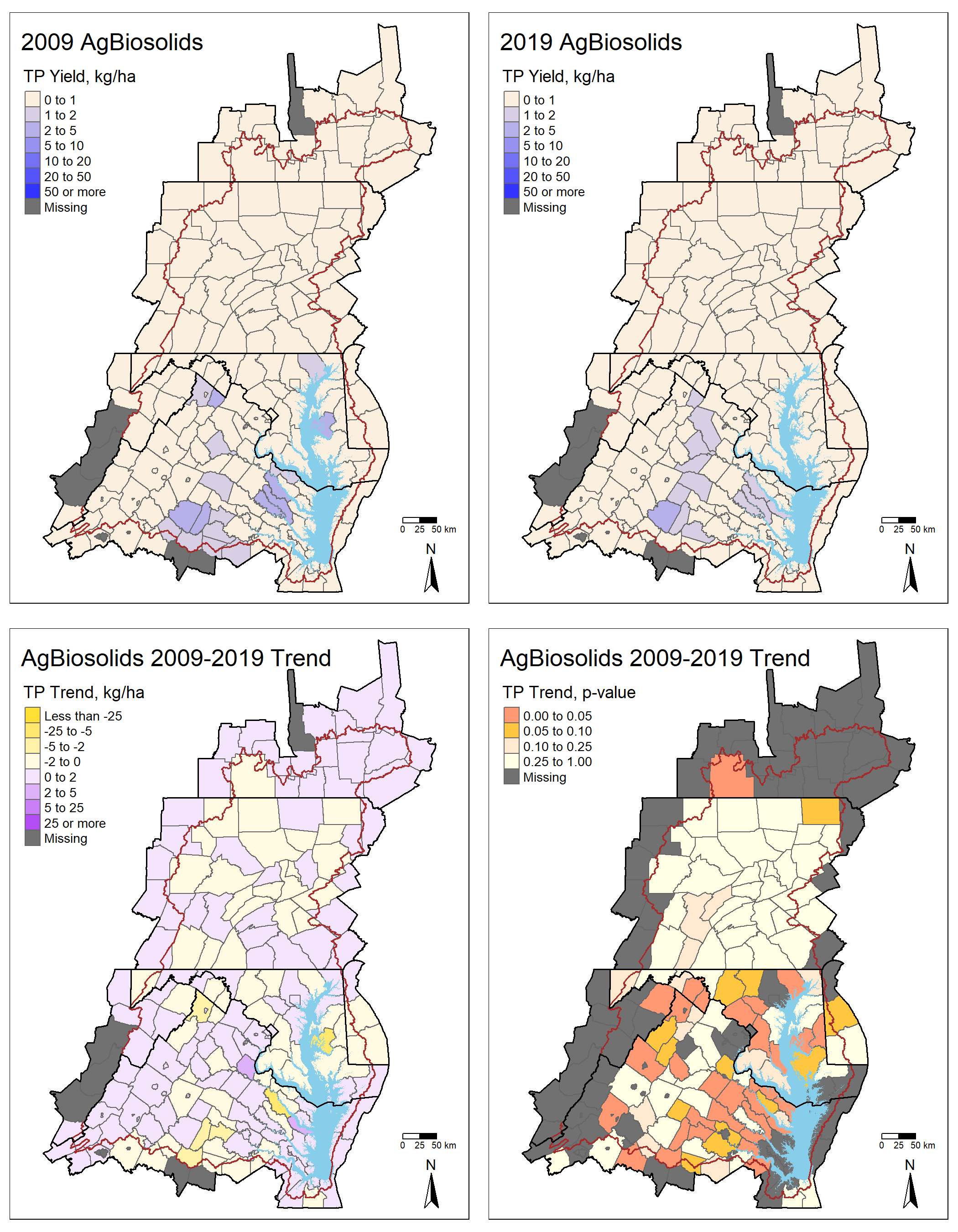
 Figure S52. For phosphorus, 2009 and 2019 biosolids applied to agricultural land (top row), the estimated Sen linear slope change in biosolid application from 2009-2019 (bottom left), and the significance of trend results by county (bottom right).
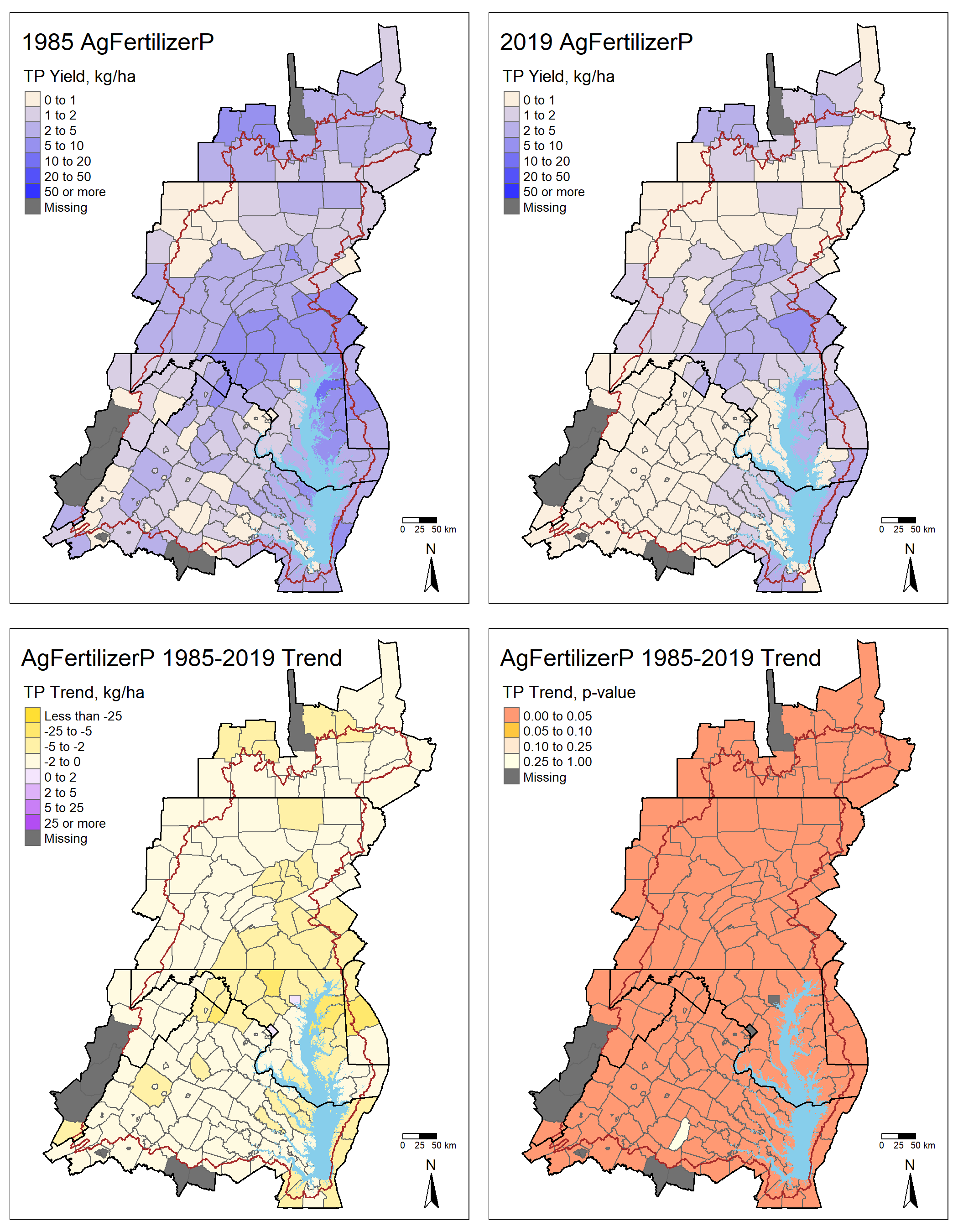
 Figure S53. For phosphorus, 1985 and 2019 fertilizer applied to agricultural land (top row), the estimated Sen linear slope change in fertilizer application from 1985-2019 (bottom left), and the significance of trend results by county (bottom right).
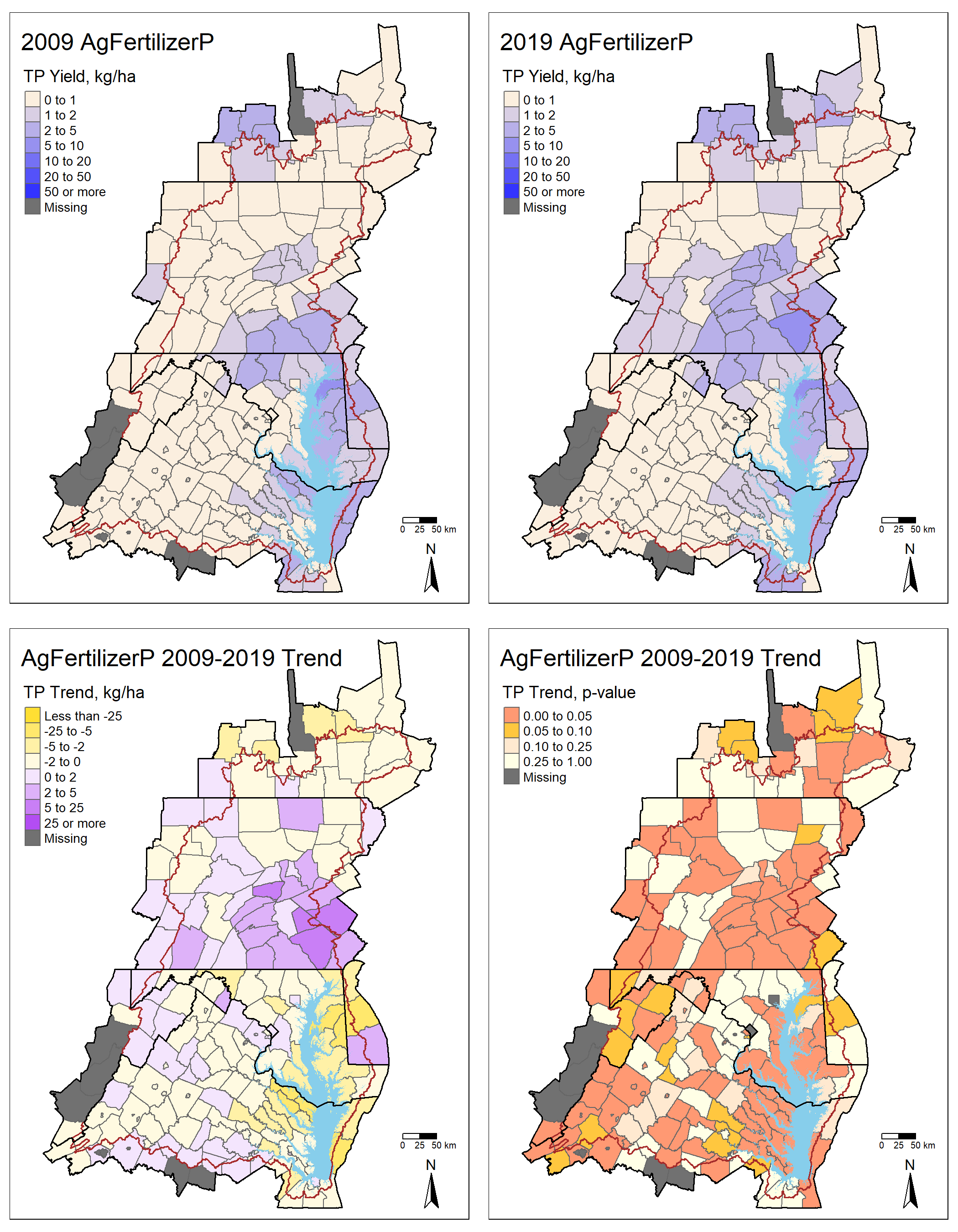
 Figure S54. For phosphorus, 2009 and 2019 fertilizer applied to agricultural land (top row), the estimated Sen linear slope change in fertilizer application from 2009-2019 (bottom left), and the significance of trend results by county (bottom right).
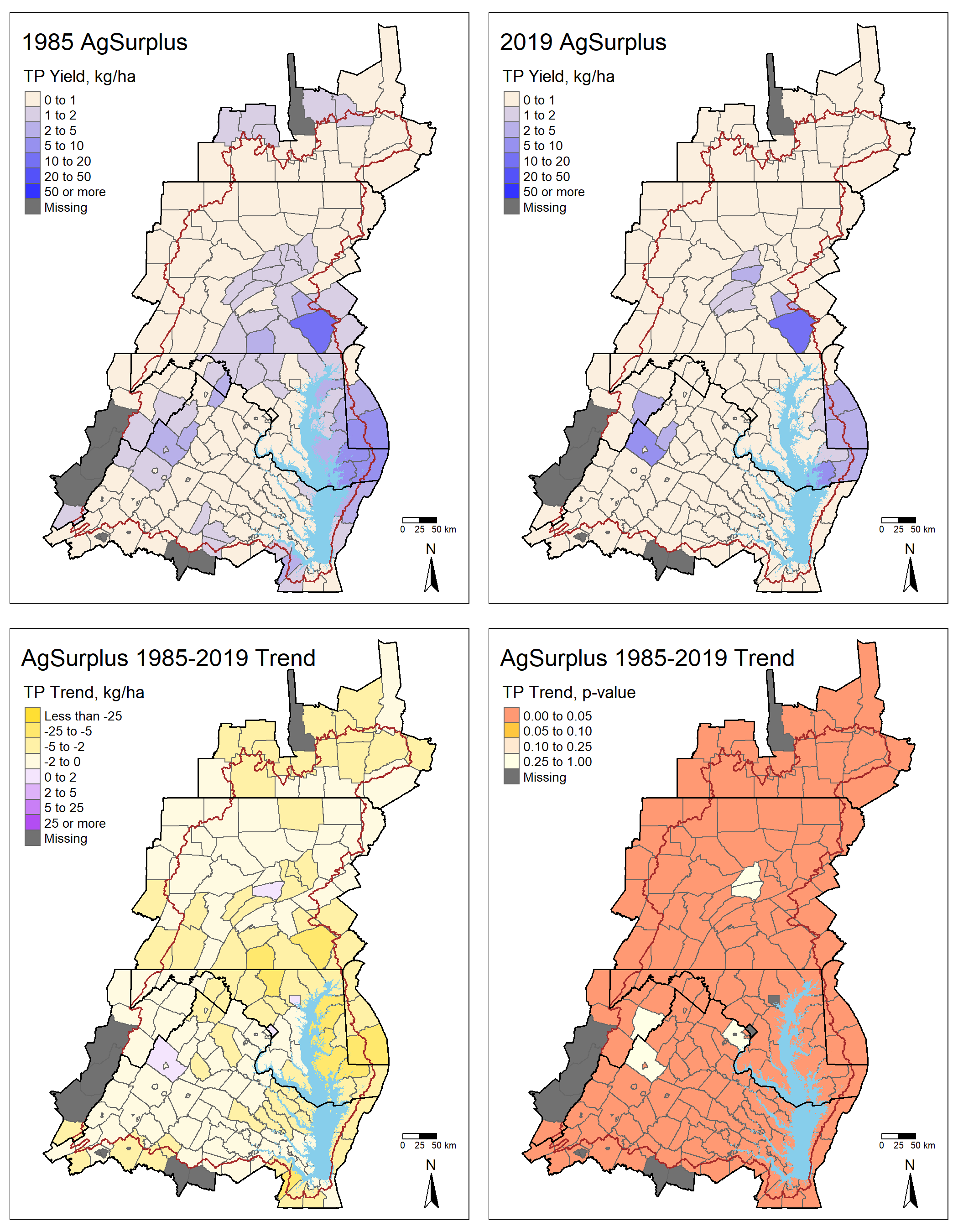
 Figure S55. For phosphorus, 1985 and 2019 agricultural surplus (top row), the estimated Sen linear slope change in agricultural surplus from 1985-2019 (bottom left), and the significance of trend results by county (bottom right).
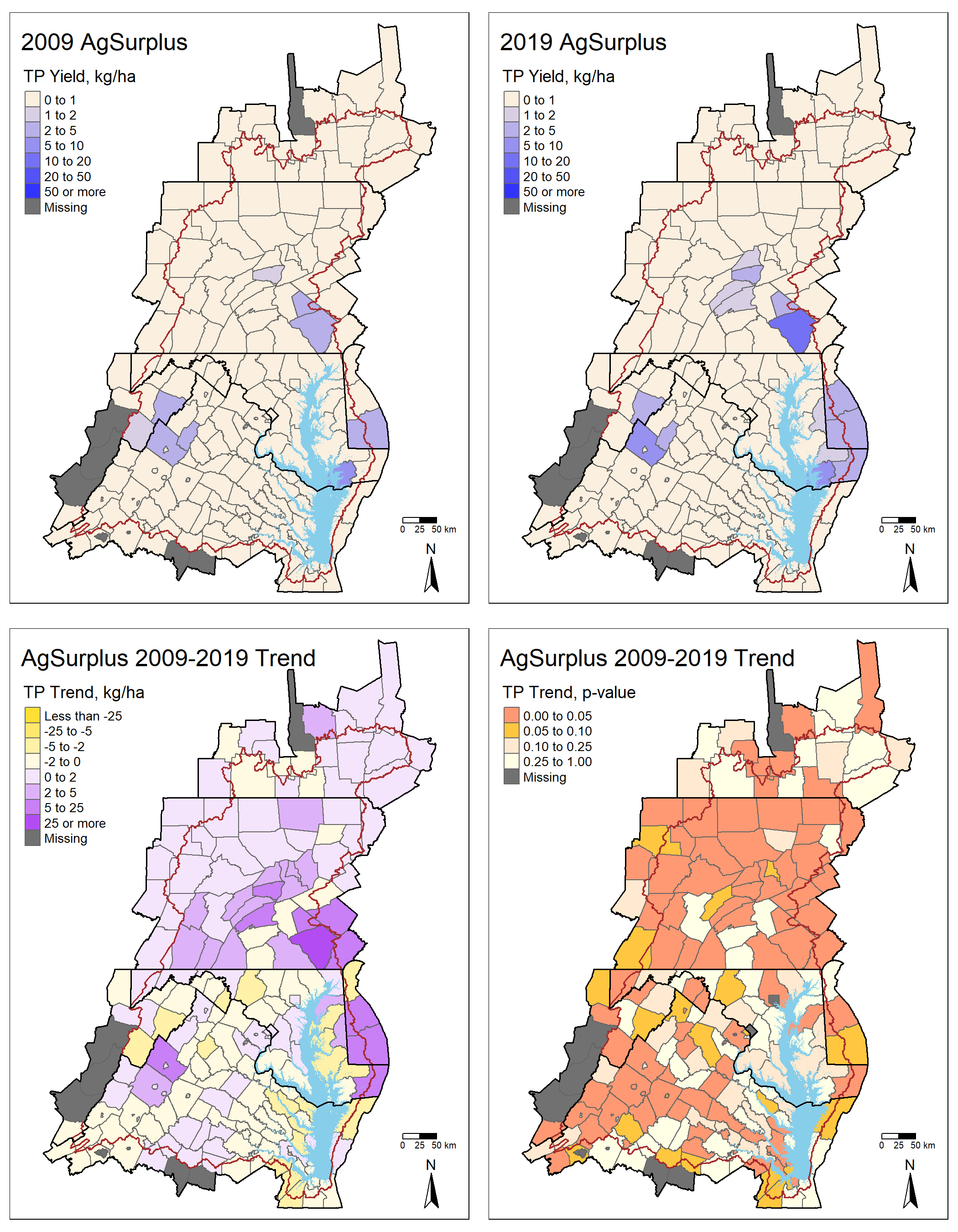
 Figure S56. For phosphorus, 2009 and 2019 agricultural surplus (top row), the estimated Sen linear slope change in agricultural surplus from 2009-2019 (bottom left), and the significance of trend results by county (bottom right).
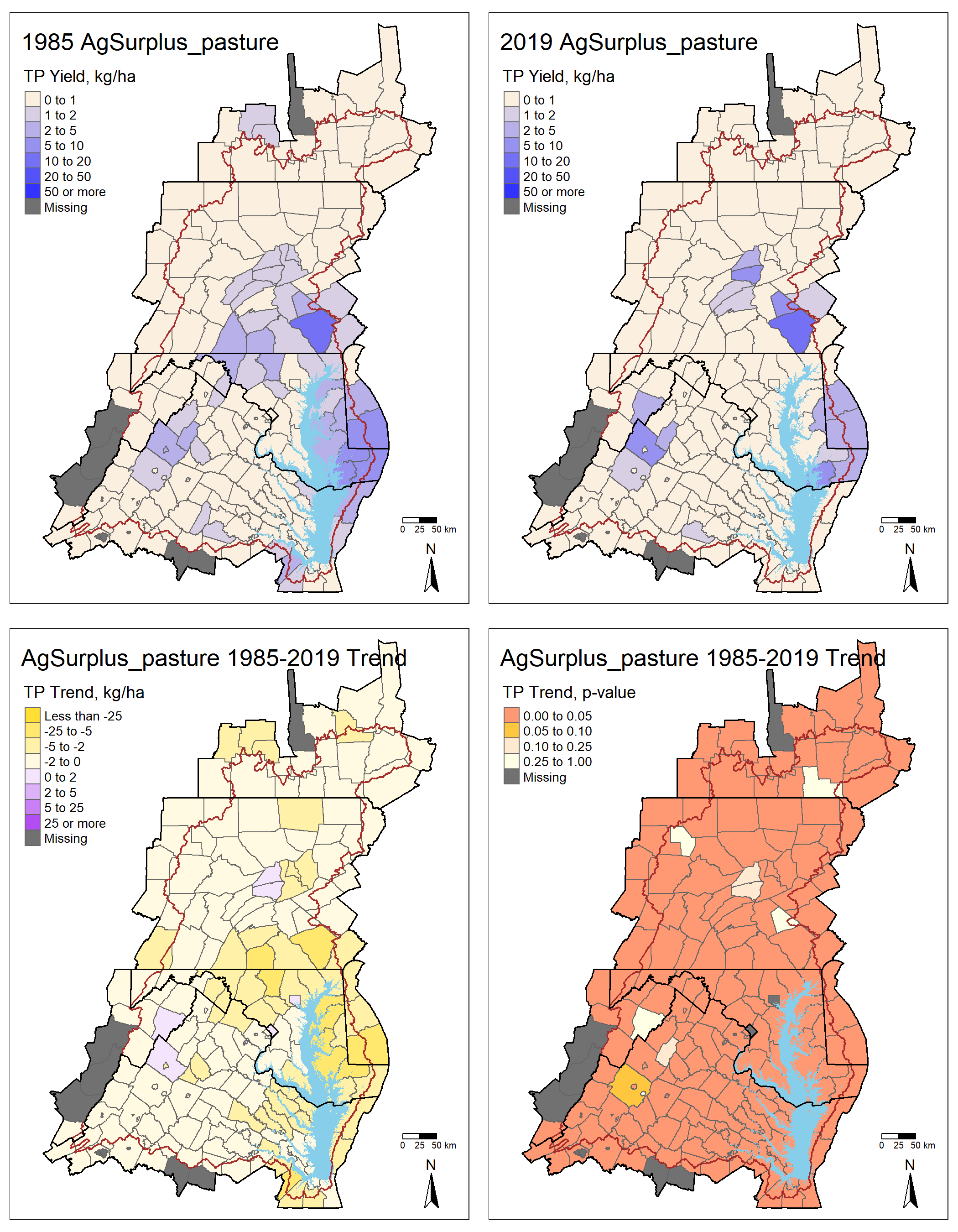
 Figure S57. For phosphorus, 1985 and 2019 agricultural surplus with pasture terms (top row), the estimated Sen linear slope change in agricultural surplus with pasture terms from 1985-2019 (bottom left), and the significance of trend results by county (bottom right).
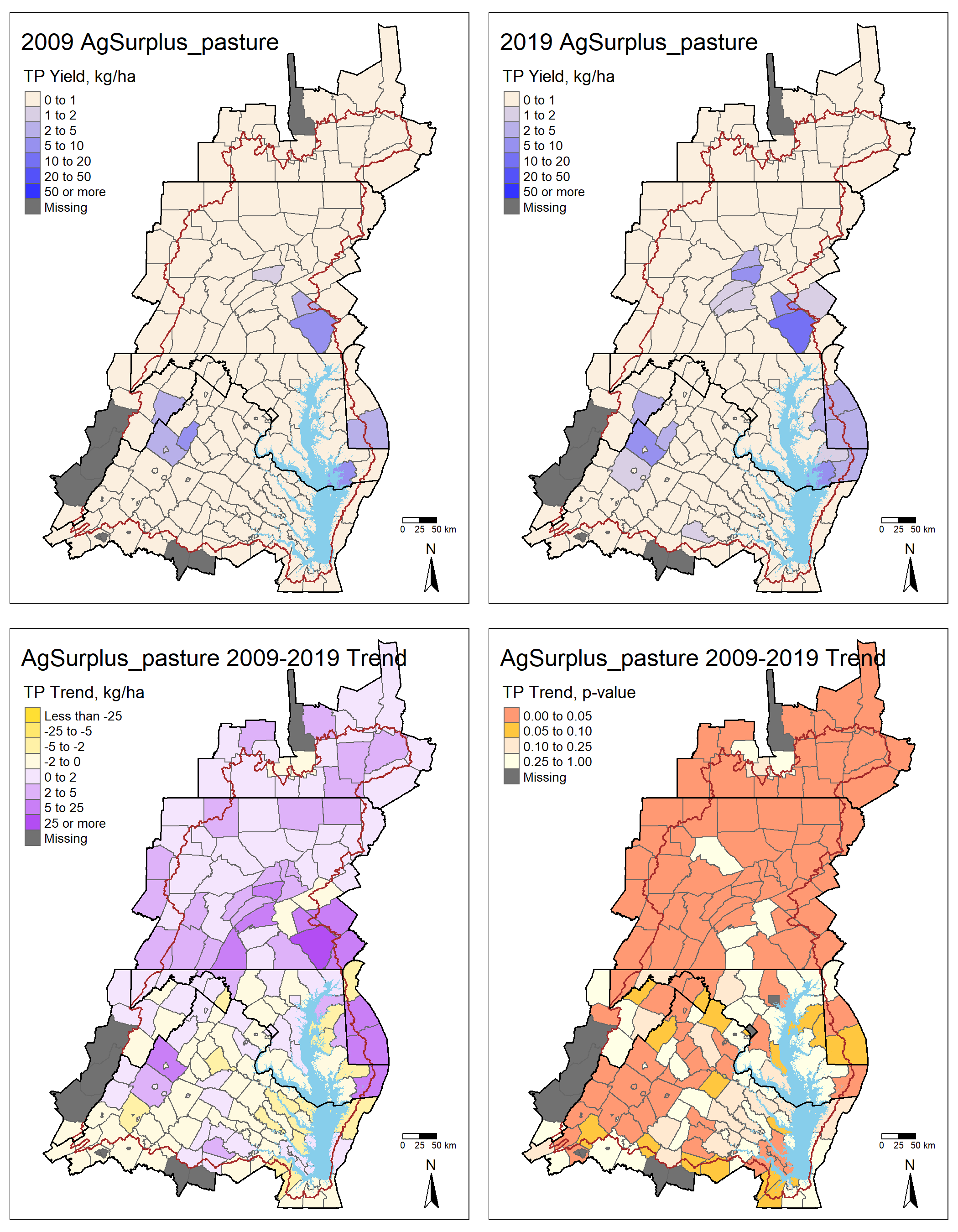
 Figure S58. For phosphorus, 2009 and 2019 agricultural surplus with pasture terms (top row), the estimated Sen linear slope change in agricultural surplus with pasture terms from 2009-2019 (bottom left), and the significance of trend results by county (bottom right).
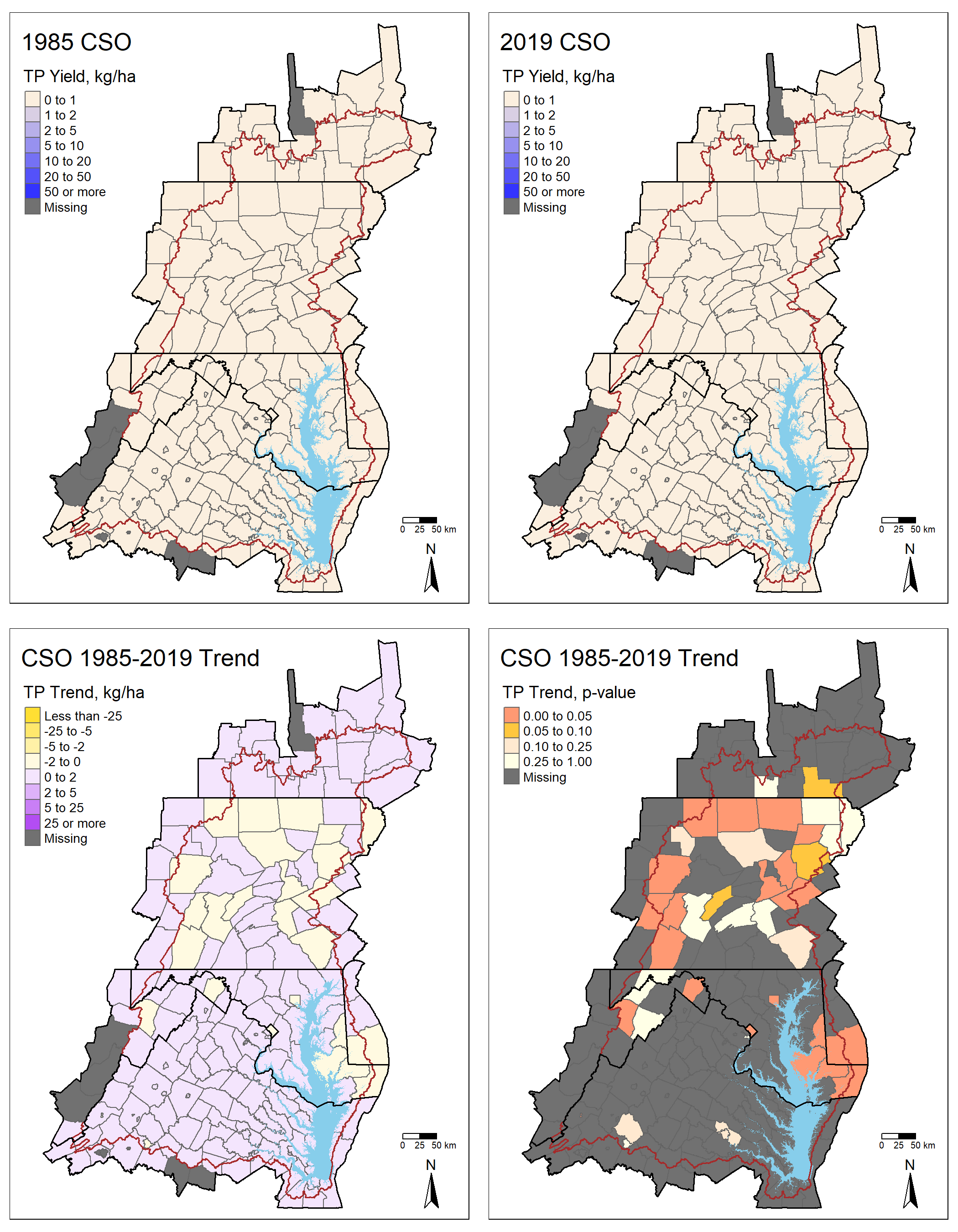
 Figure S59. For phosphorus, 1985 and 2019 combined sewage overflow loads (top row), the estimated Sen linear slope change in combined sewage overflow loads from 1985-2019 (bottom left), and the significance of trend results by county (bottom right).
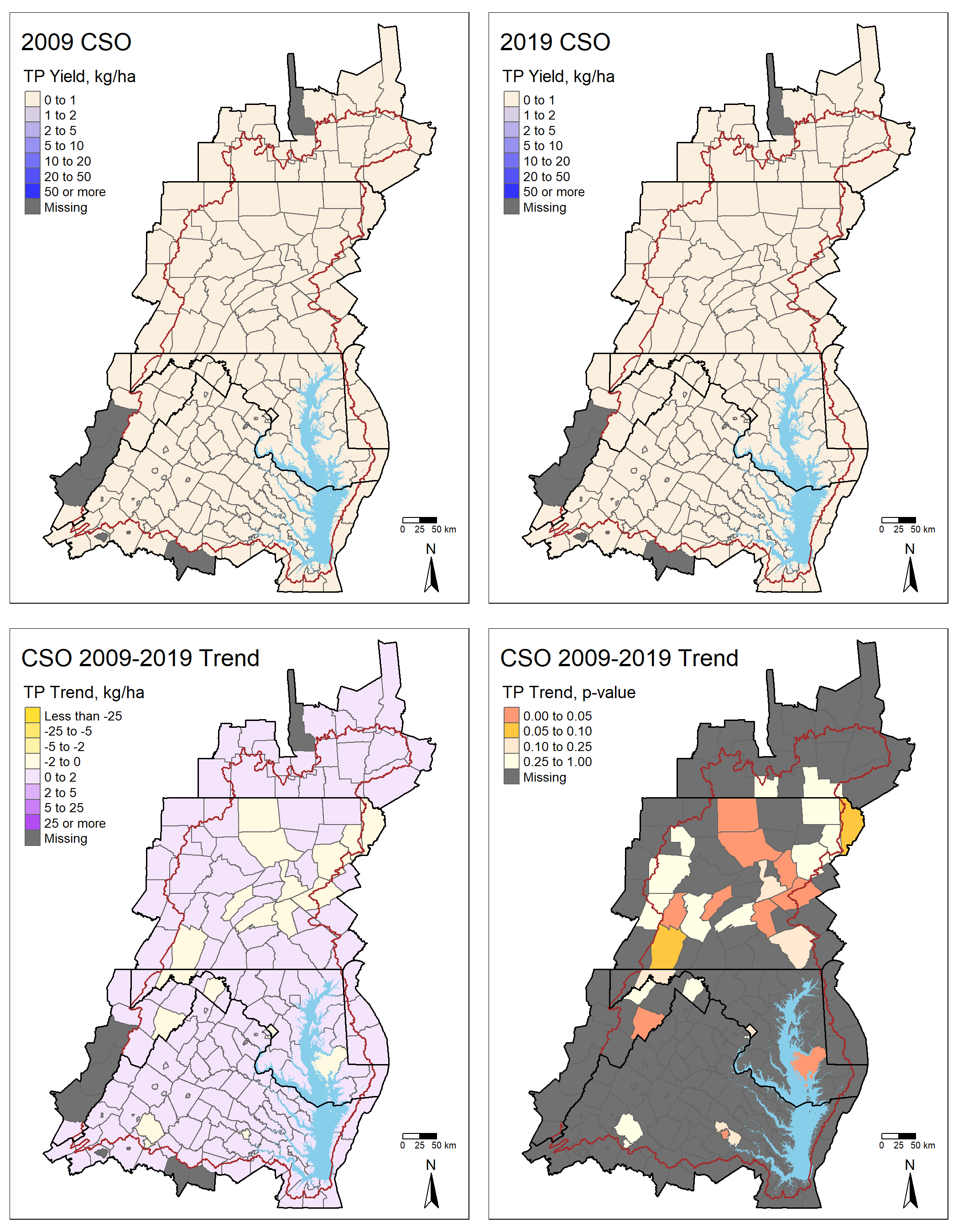
 Figure S60. For phosphorus, 2009 and 2019 combined sewage overflow loads (top row), the estimated Sen linear slope change in combined sewage overflow loads from 2009-2019 (bottom left), and the significance of trend results by county (bottom right).
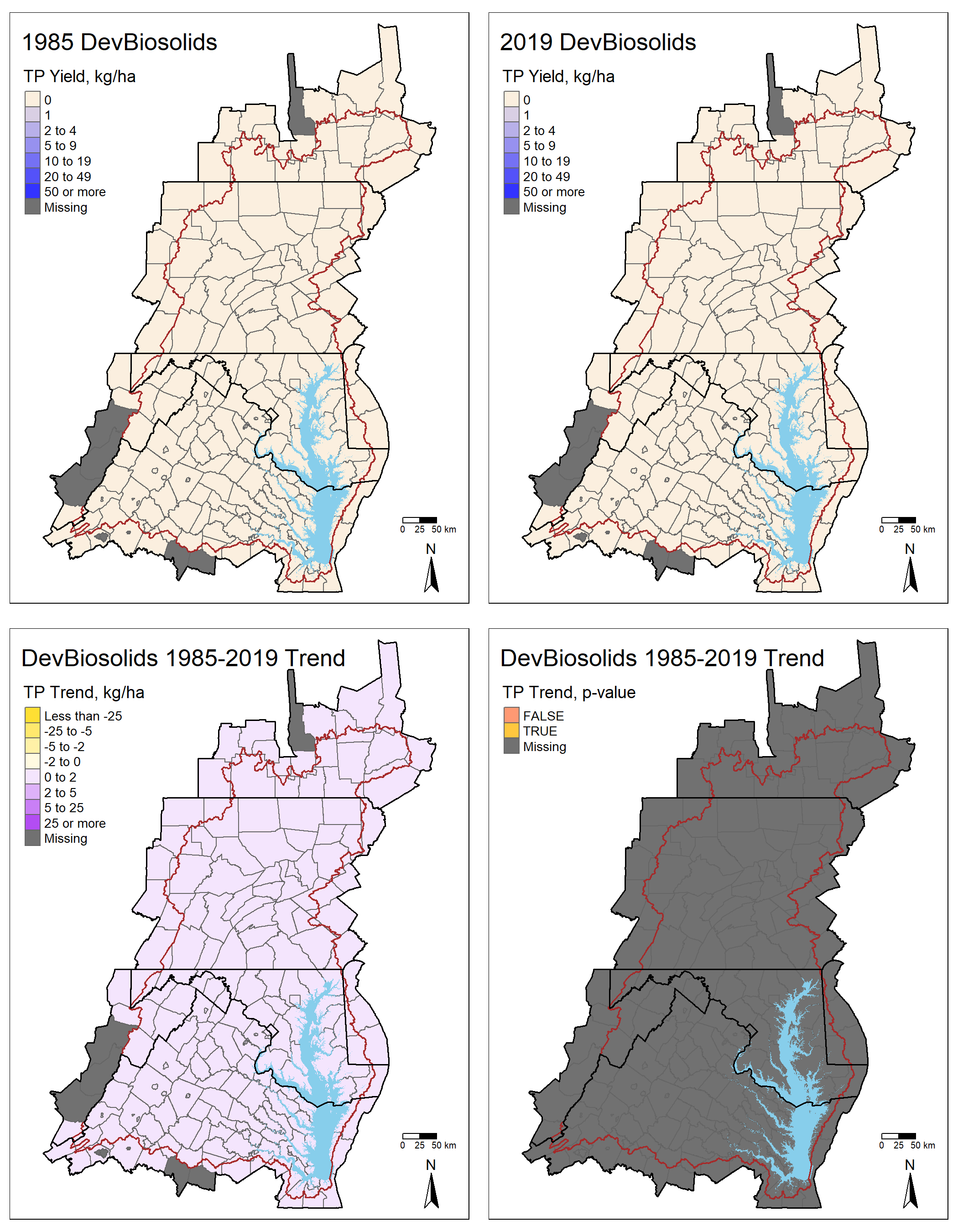
 Figure S61. For phosphorus, 1985 and 2019 biosolids applied to developed land (top row), the estimated Sen linear slope change in biosolid application from 1985-2019 (bottom left), and the significance of trend results by county (bottom right).
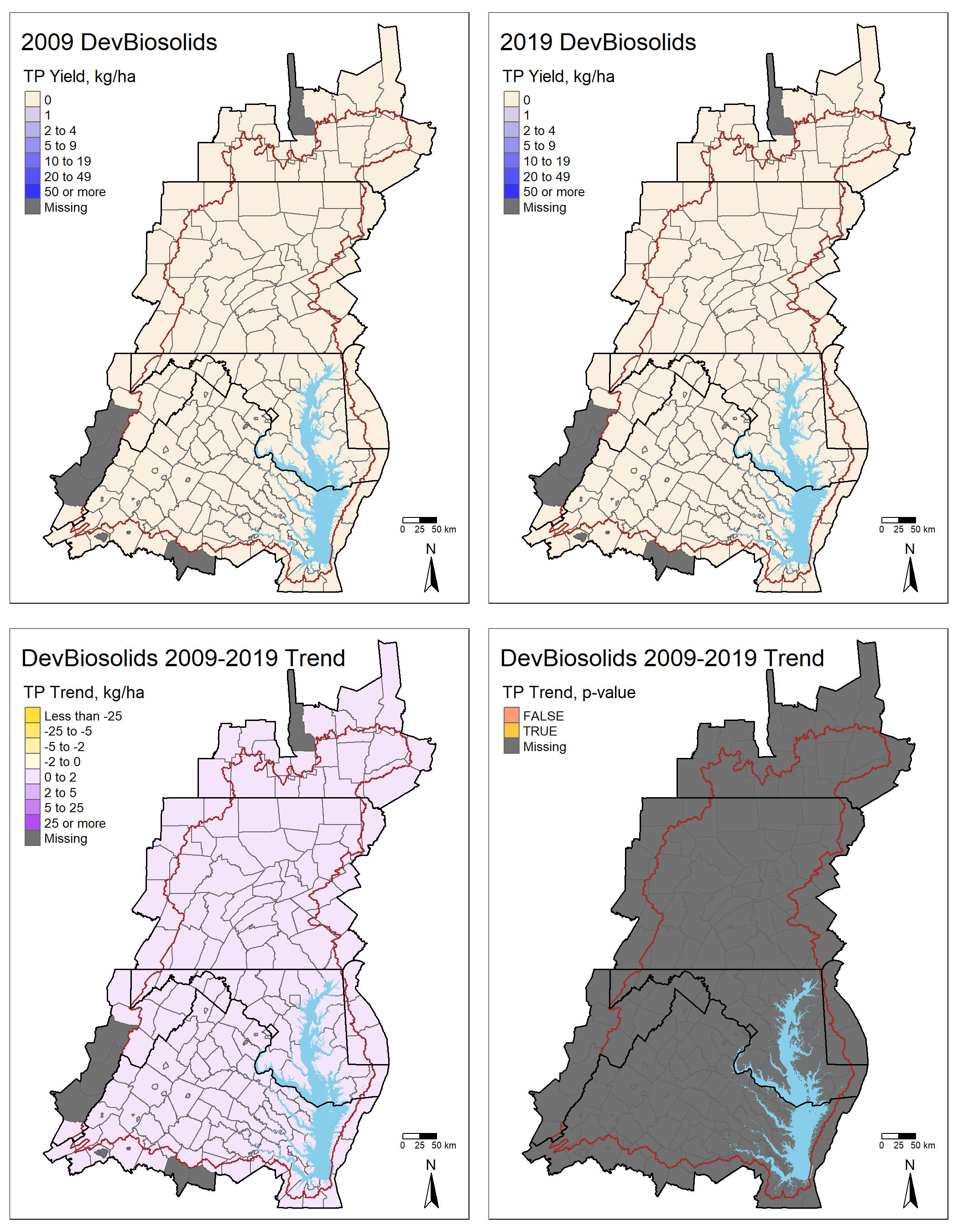
 Figure S62. For phosphorus, 2009 and 2019 biosolids applied to developed land (top row), the estimated Sen linear slope change in biosolid application from 2009-2019 (bottom left), and the significance of trend results by county (bottom right).
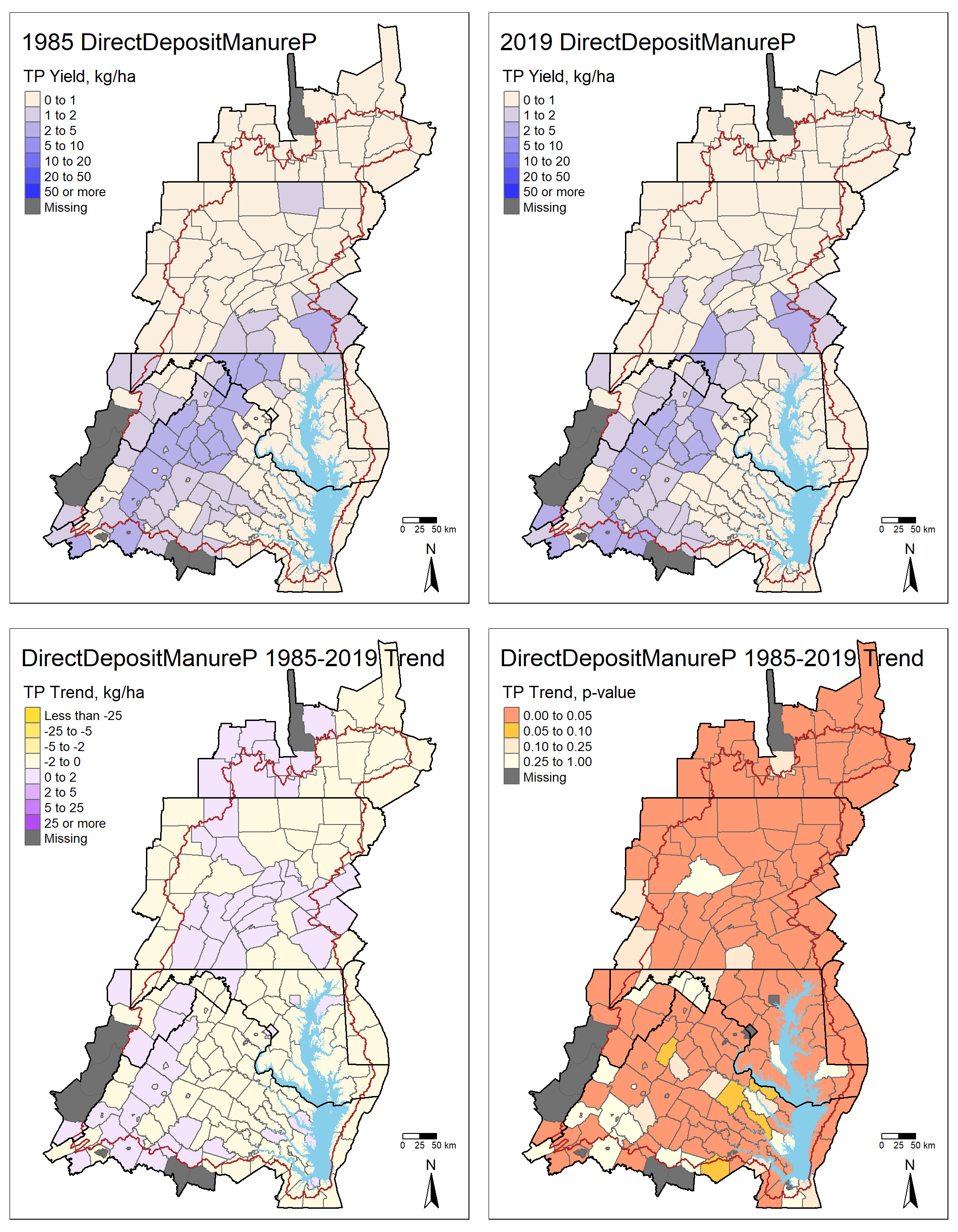
 Figure S63. For phosphorus, 1985 and 2019 direct manure deposited on pasture (top row), the estimated Sen linear slope change in direct manure deposited on pasture from 1985-2019 (bottom left), and the significance of trend results by county (bottom right).
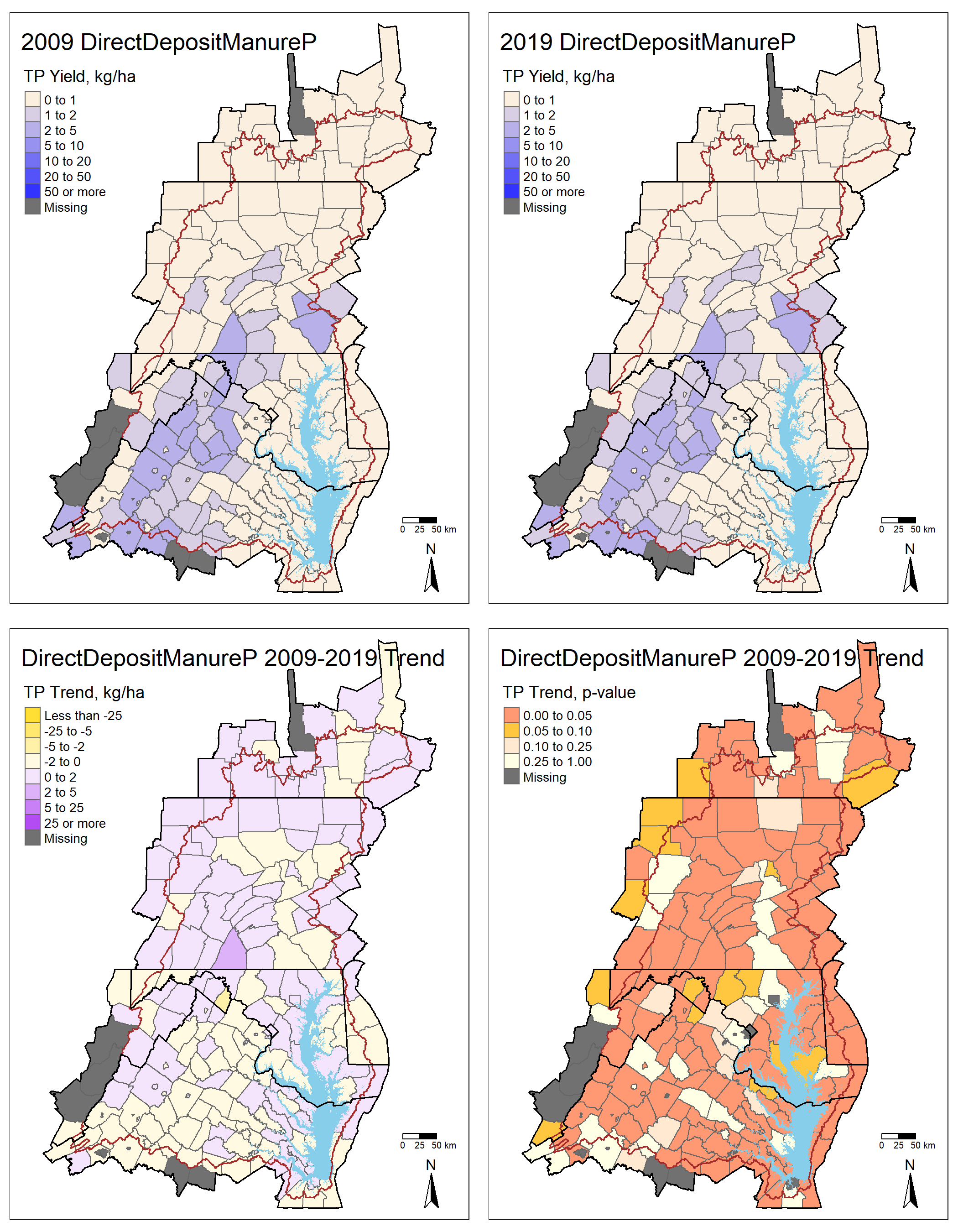
 Figure S64. For phosphorus, 2009 and 2019 direct manure deposited on pasture (top row), the estimated Sen linear slope change in direct manure deposited on pasture from 2009-2019 (bottom left), and the significance of trend results by county (bottom right).
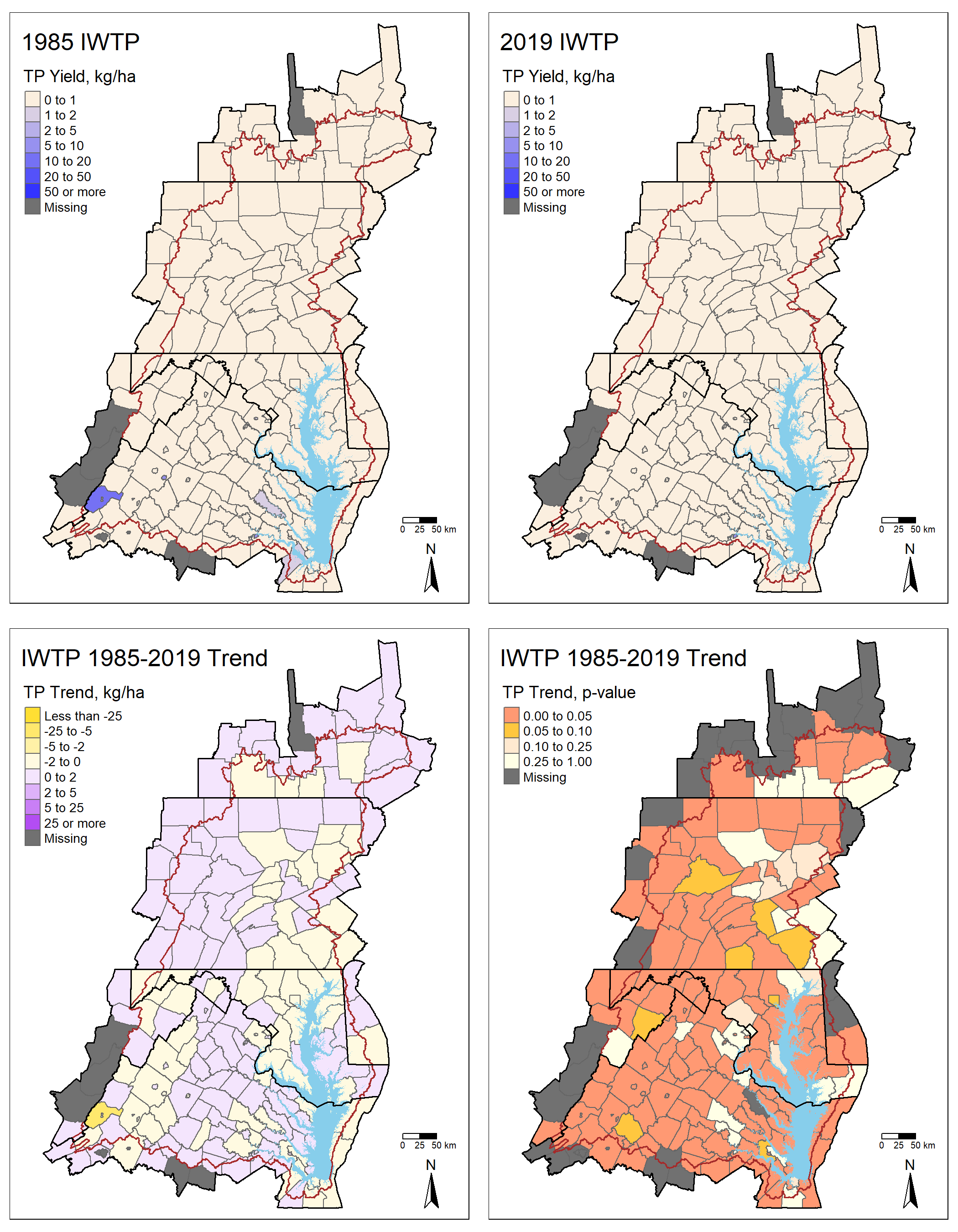
 Figure S65. For phosphorus, 1985 and 2019 industrial wastewater treatment plant load (top row), the estimated Sen linear slope change in industrial wastewater treatment plant load from 1985-2019 (bottom left), and the significance of trend results by county (bottom right).
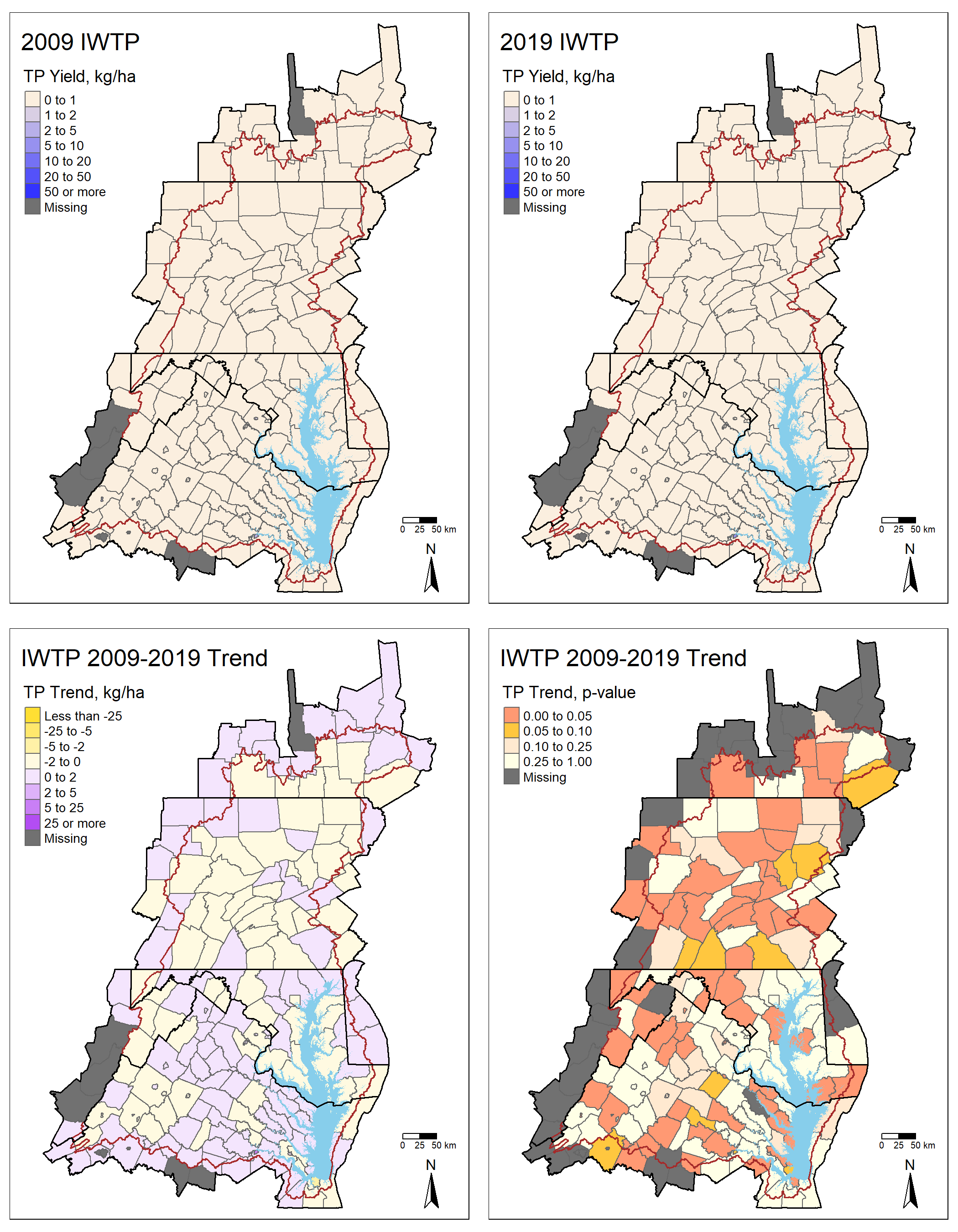
 Figure S66. For phosphorus, 2009 and 2019 industrial wastewater treatment plant load (top row), the estimated Sen linear slope change in industrial wastewater treatment plant load from 2009-2019 (bottom left), and the significance of trend results by county (bottom right).
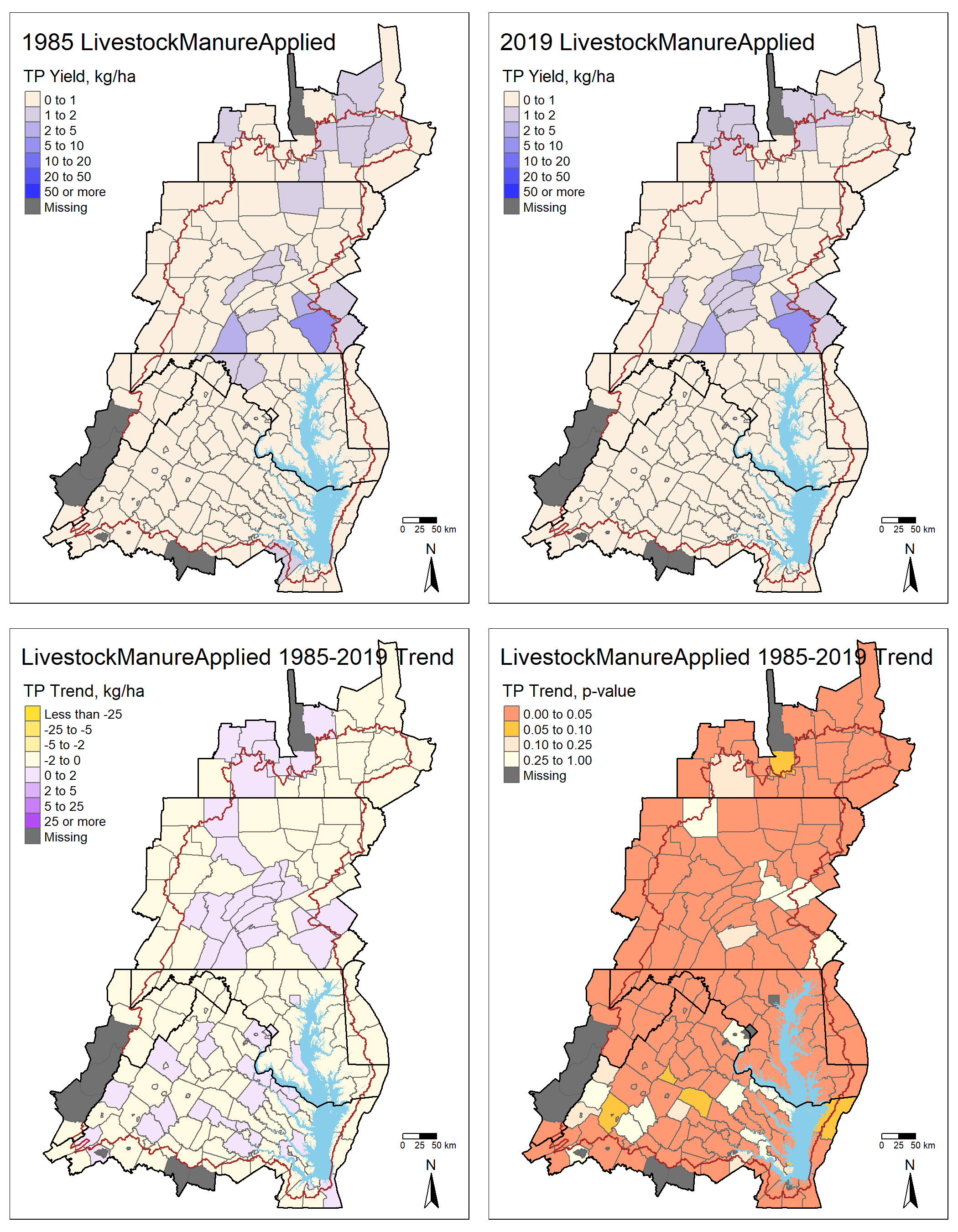
 Figure S67. For phosphorus, 1985 and 2019 livestock manure applied (top row), the estimated Sen linear slope change in livestock manure applied from 1985-2019 (bottom left), and the significance of trend results by county (bottom right).
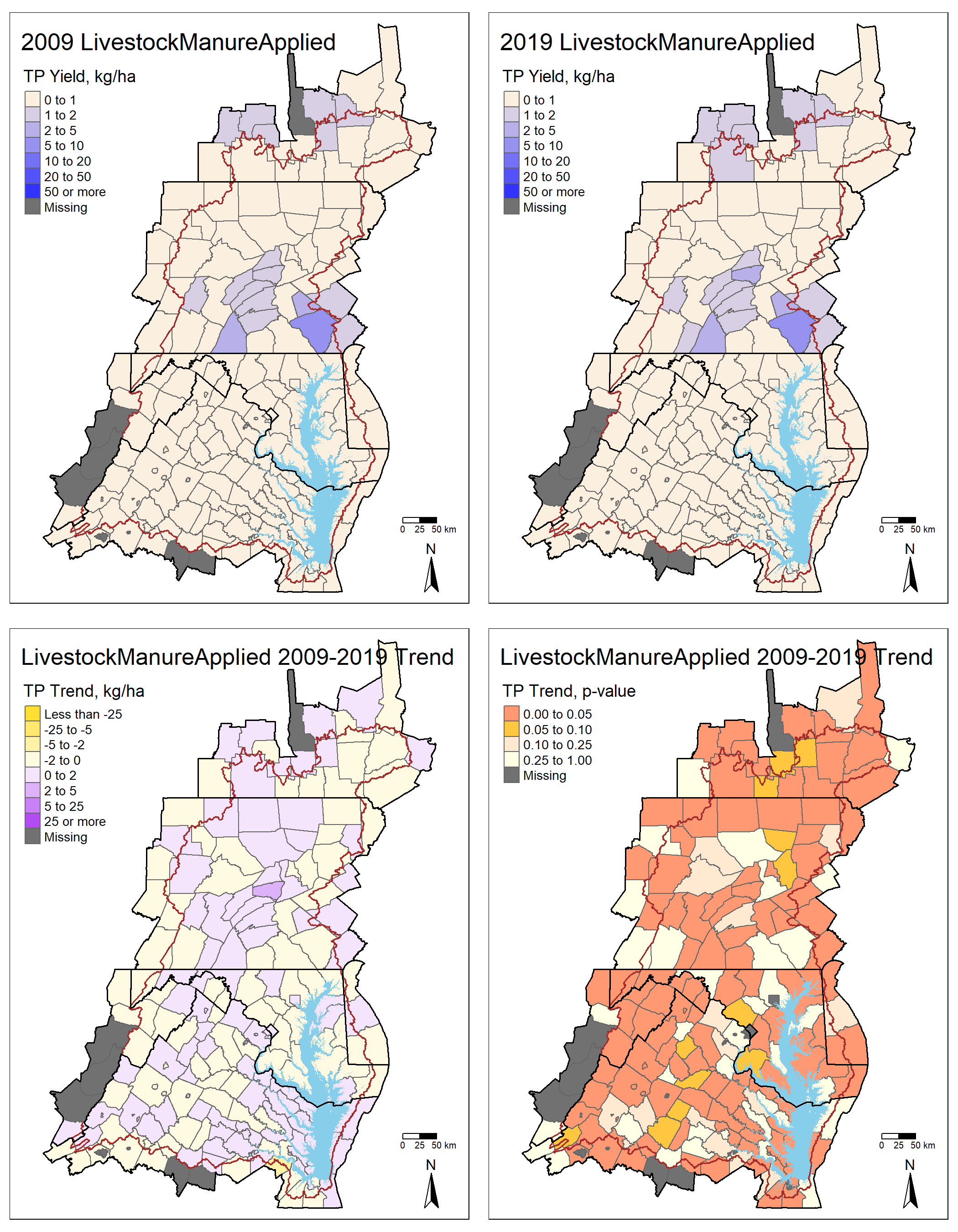
 Figure S68. For phosphorus, 2009 and 2019 livestock manure applied (top row), the estimated Sen linear slope change in livestock manure applied from 2009-2019 (bottom left), and the significance of trend results by county (bottom right).
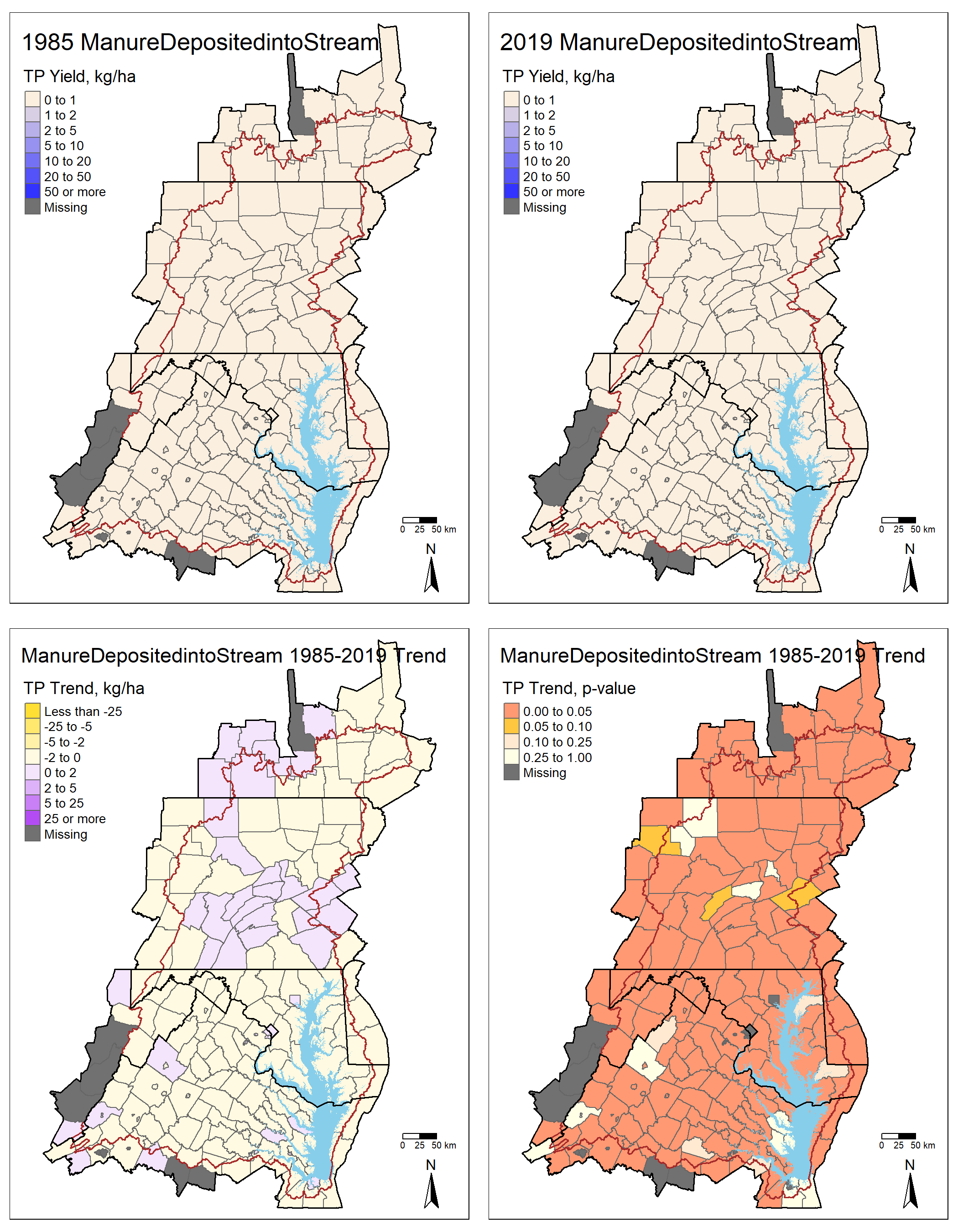
 Figure S69. For phosphorus, 1985 and 2019 livestock manure deposited into stream/riparian areas (top row), the estimated Sen linear slope change in livestock manure deposited into stream/riparian areas from 1985-2019 (bottom left), and the significance of trend results by county (bottom right).
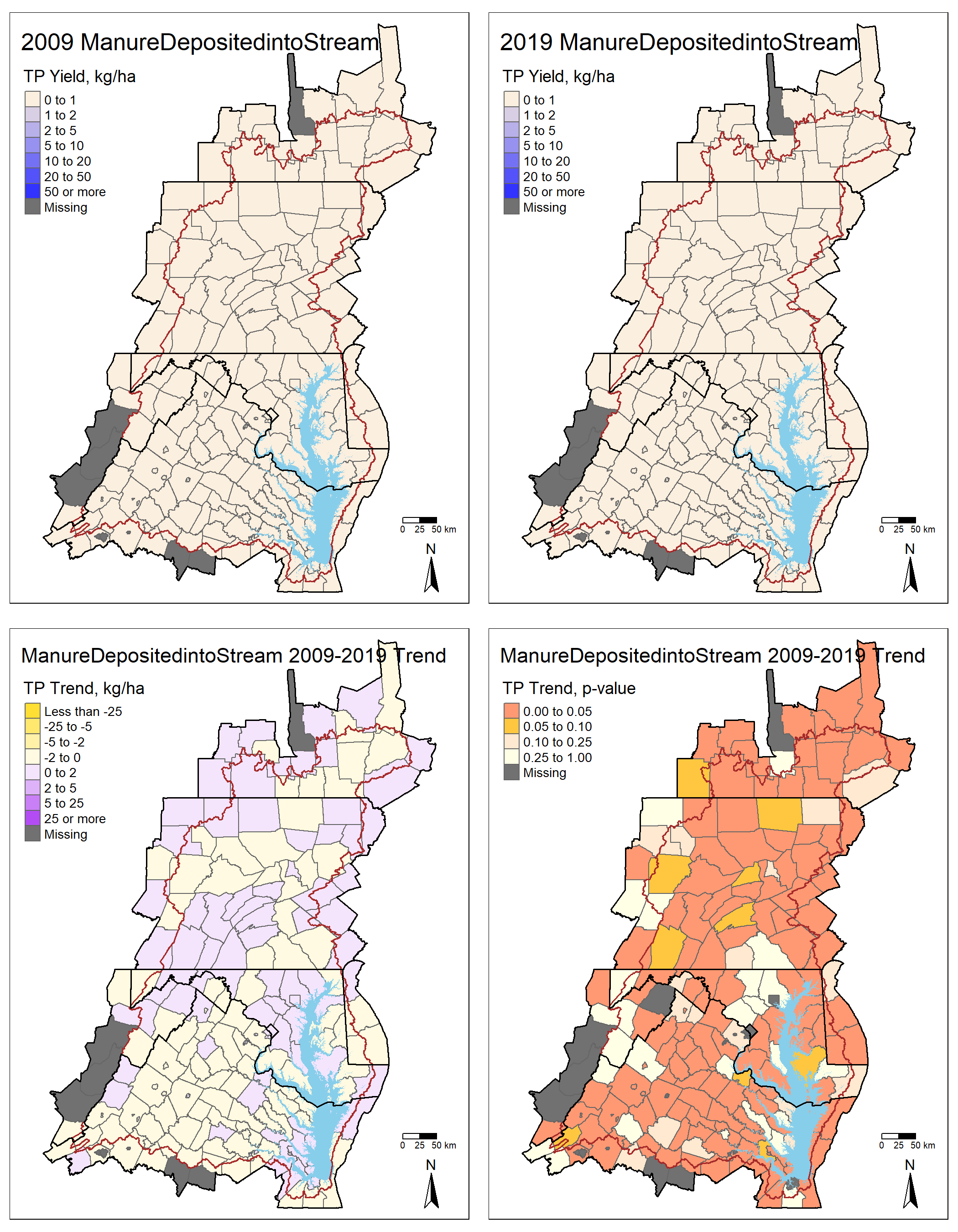
 Figure S70. For phosphorus, 2009 and 2019 livestock manure deposited into stream/riparian areas (top row), the estimated Sen linear slope change in livestock manure deposited into stream/riparian areas from 2009-2019 (bottom left), and the significance of trend results by county (bottom right).
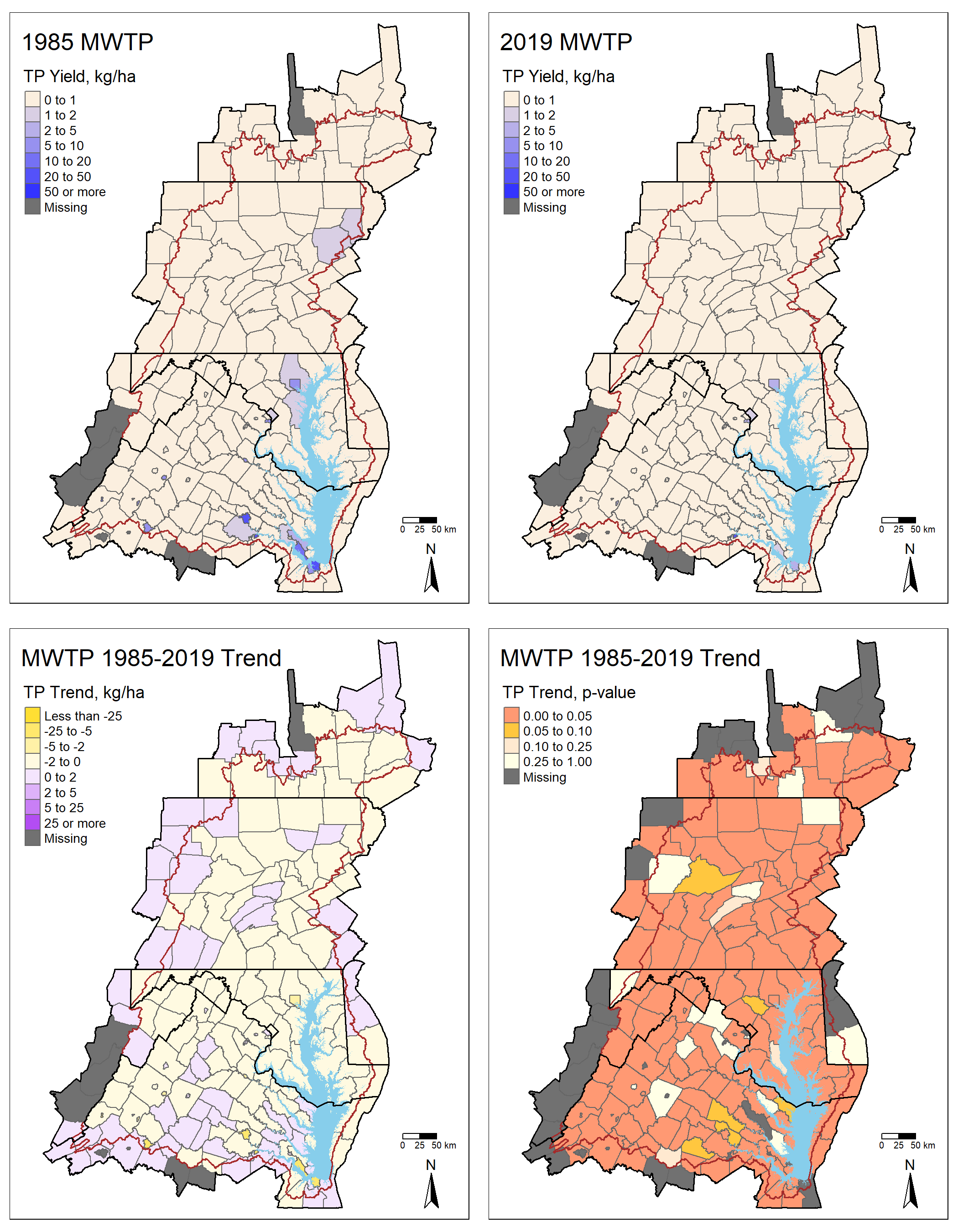
 Figure S71. For phosphorus, 1985 and 2019 municipal wastewater treatment plant load (top row), the estimated Sen linear slope change in municipal wastewater treatment plant load from 1985-2019 (bottom left), and the significance of trend results by county (bottom right).
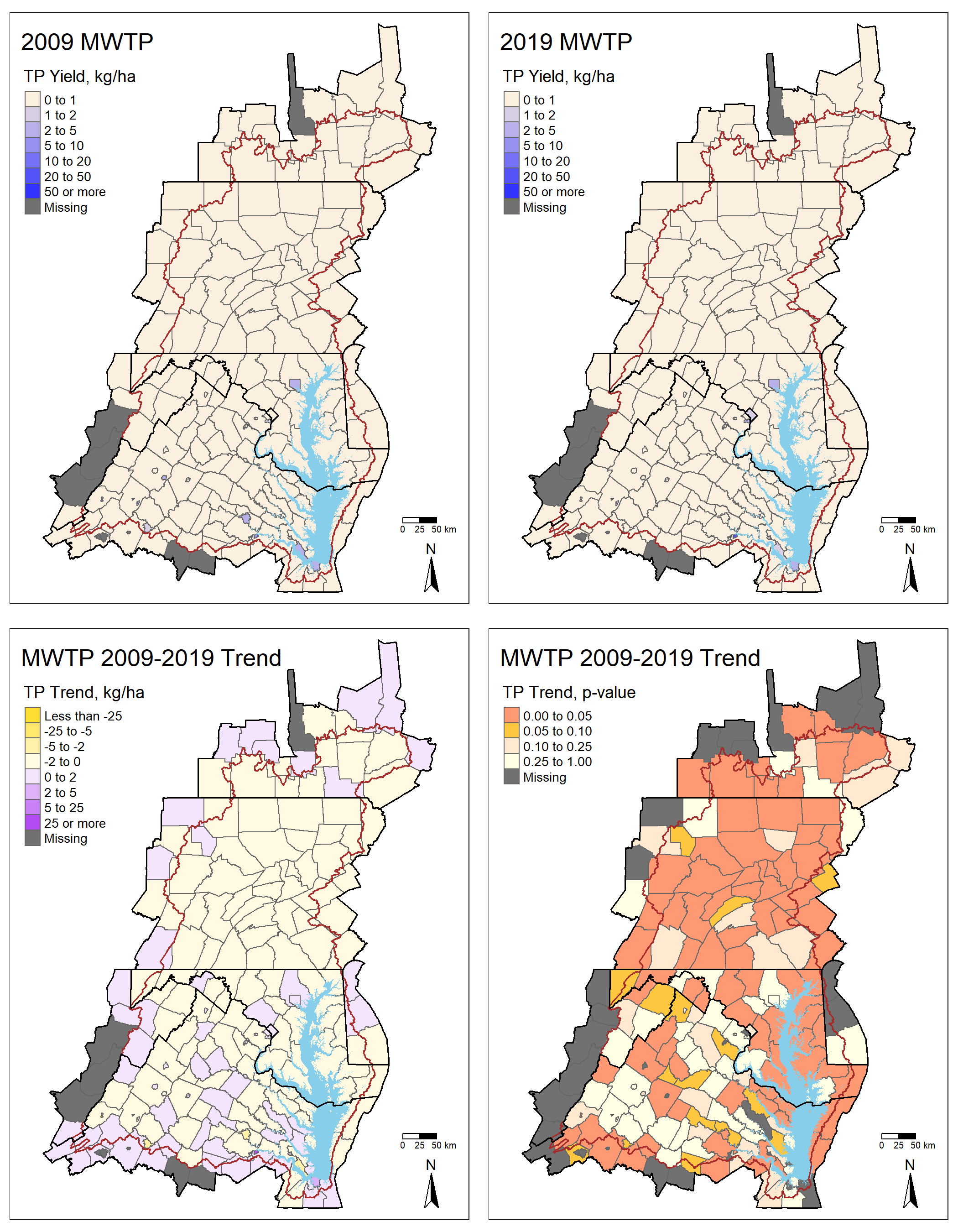
 Figure S72. For phosphorus, 2009 and 2019 municipal wastewater treatment plant load (top row), the estimated Sen linear slope change in municipal wastewater treatment plant load from 2009-2019 (bottom left), and the significance of trend results by county (bottom right).
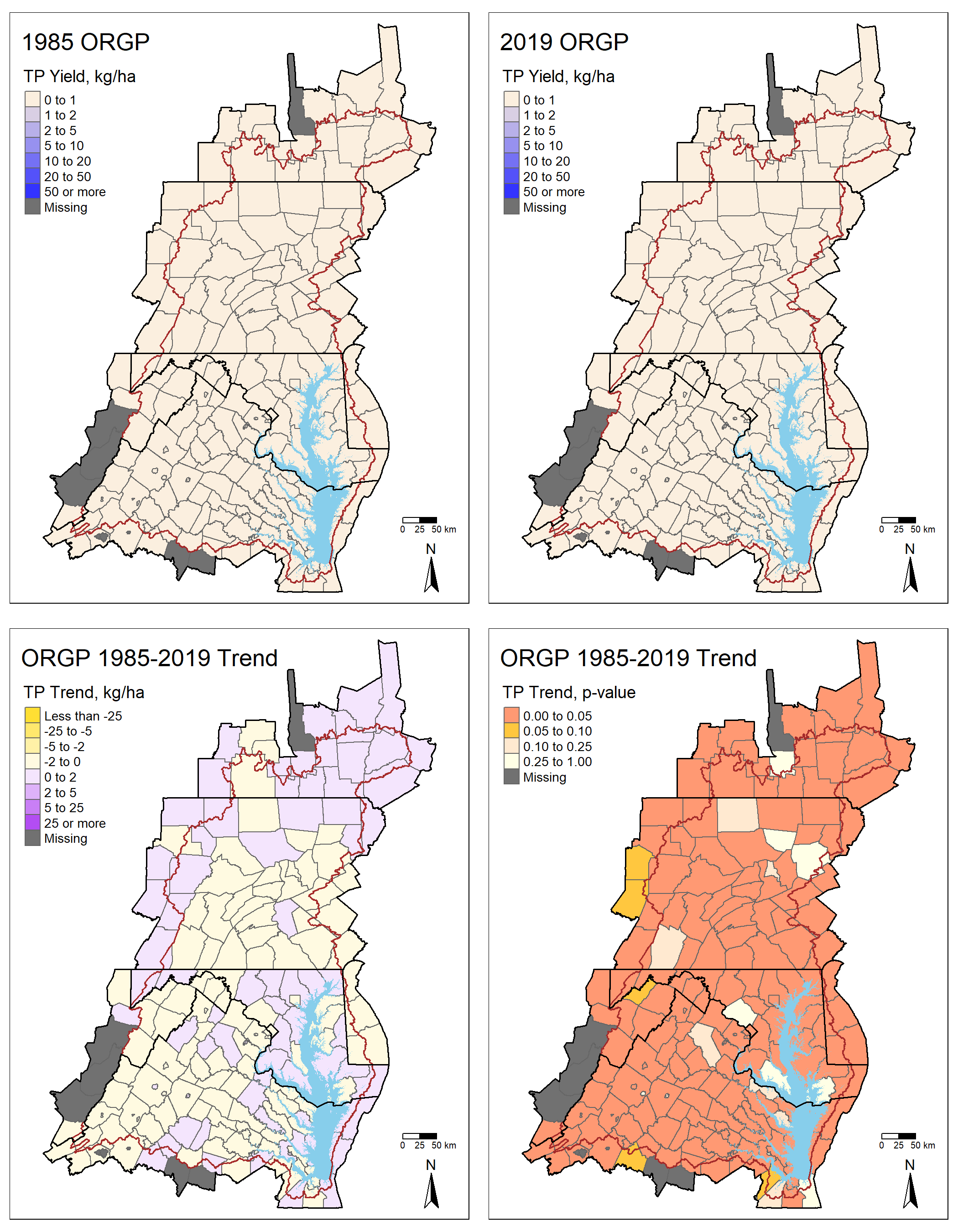
 Figure S73. For phosphorus, 1985 and 2019 atmospheric organic deposition (top row), the estimated Sen linear slope change in atmospheric organic deposition from 1985-2019 (bottom left), and the significance of trend results by county (bottom right).
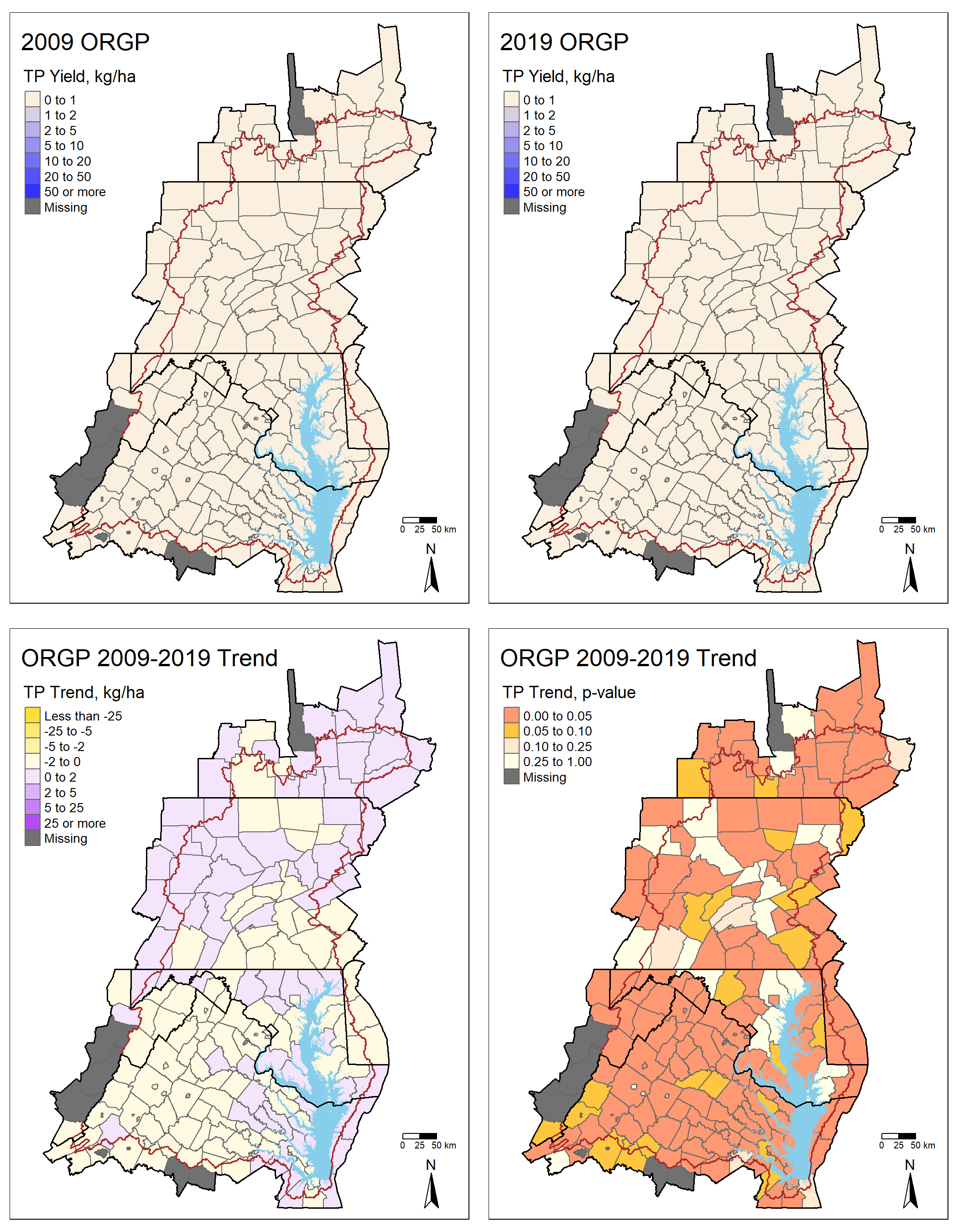
 Figure S74. For phosphorus, 2009 and 2019 atmospheric organic deposition (top row), the estimated Sen linear slope change in atmospheric organic deposition from 2009-2019 (bottom left), and the significance of trend results by county (bottom right).
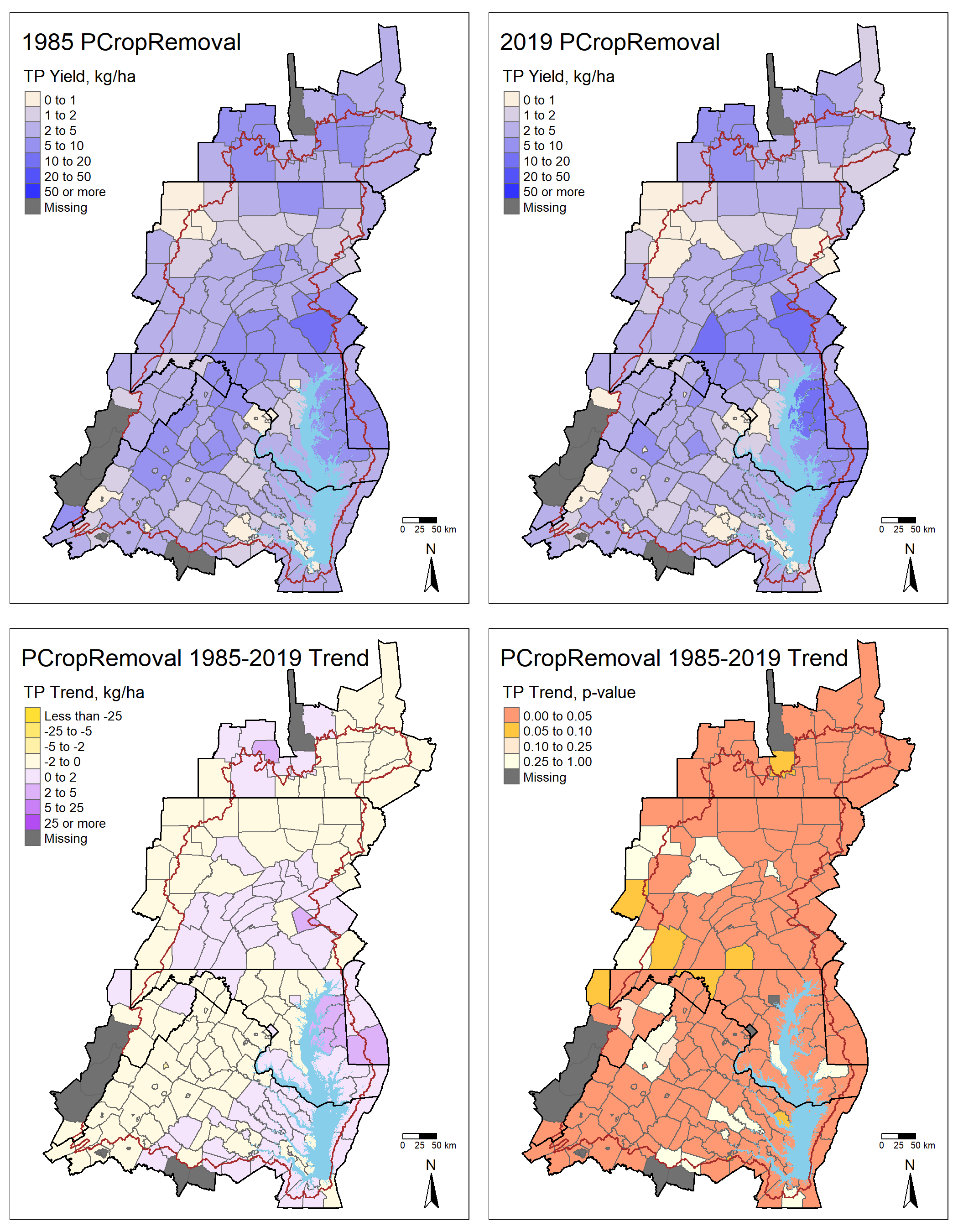
 Figure S75. For phosphorus, 1985 and 2019 crop removal (top row), the estimated Sen linear slope change in crop removal from 1985-2019 (bottom left), and the significance of trend results by county (bottom right).
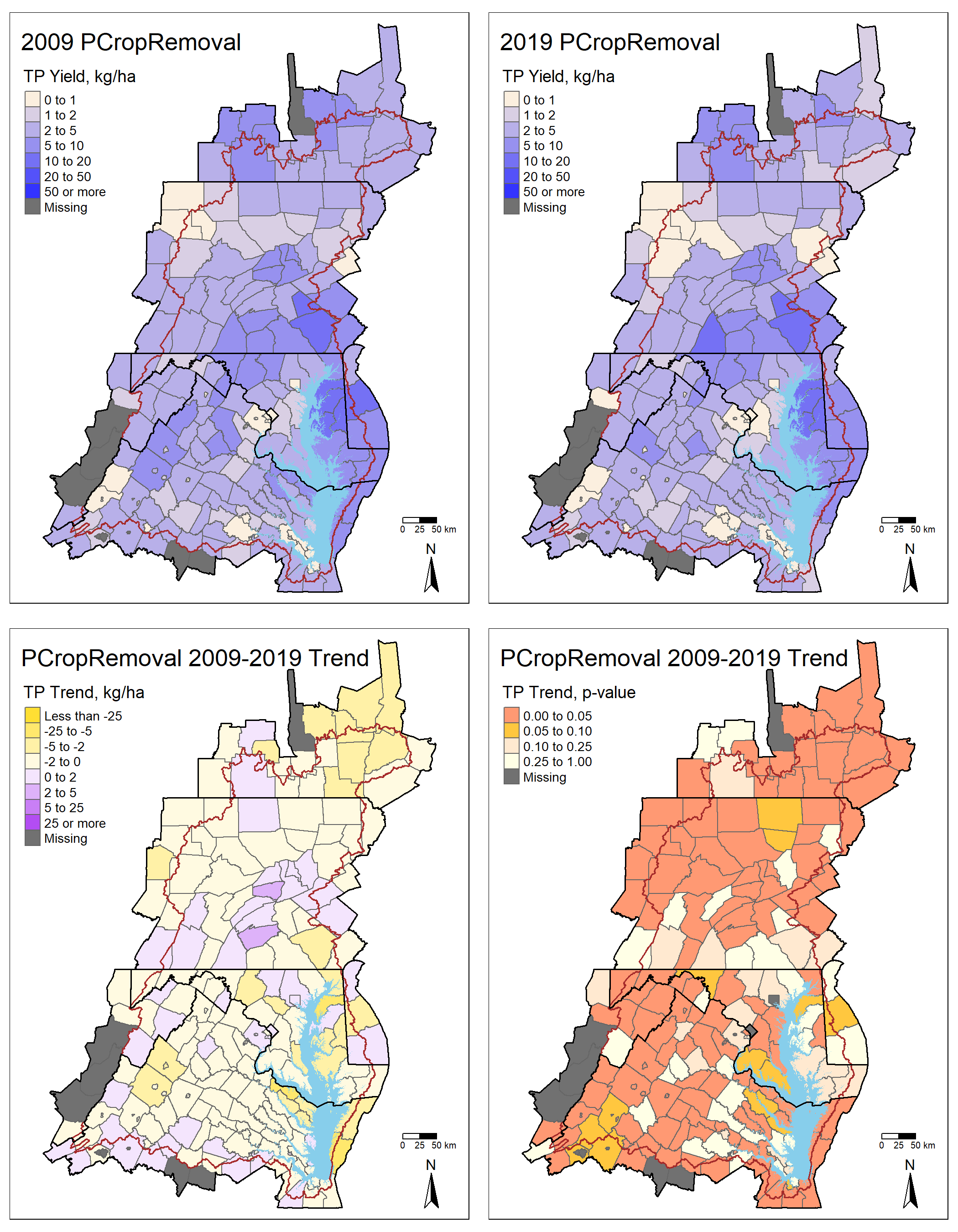
 Figure S76. For phosphorus, 2009 and 2019 crop removal (top row), the estimated Sen linear slope change in crop removal from 2009-2019 (bottom left), and the significance of trend results by county (bottom right).
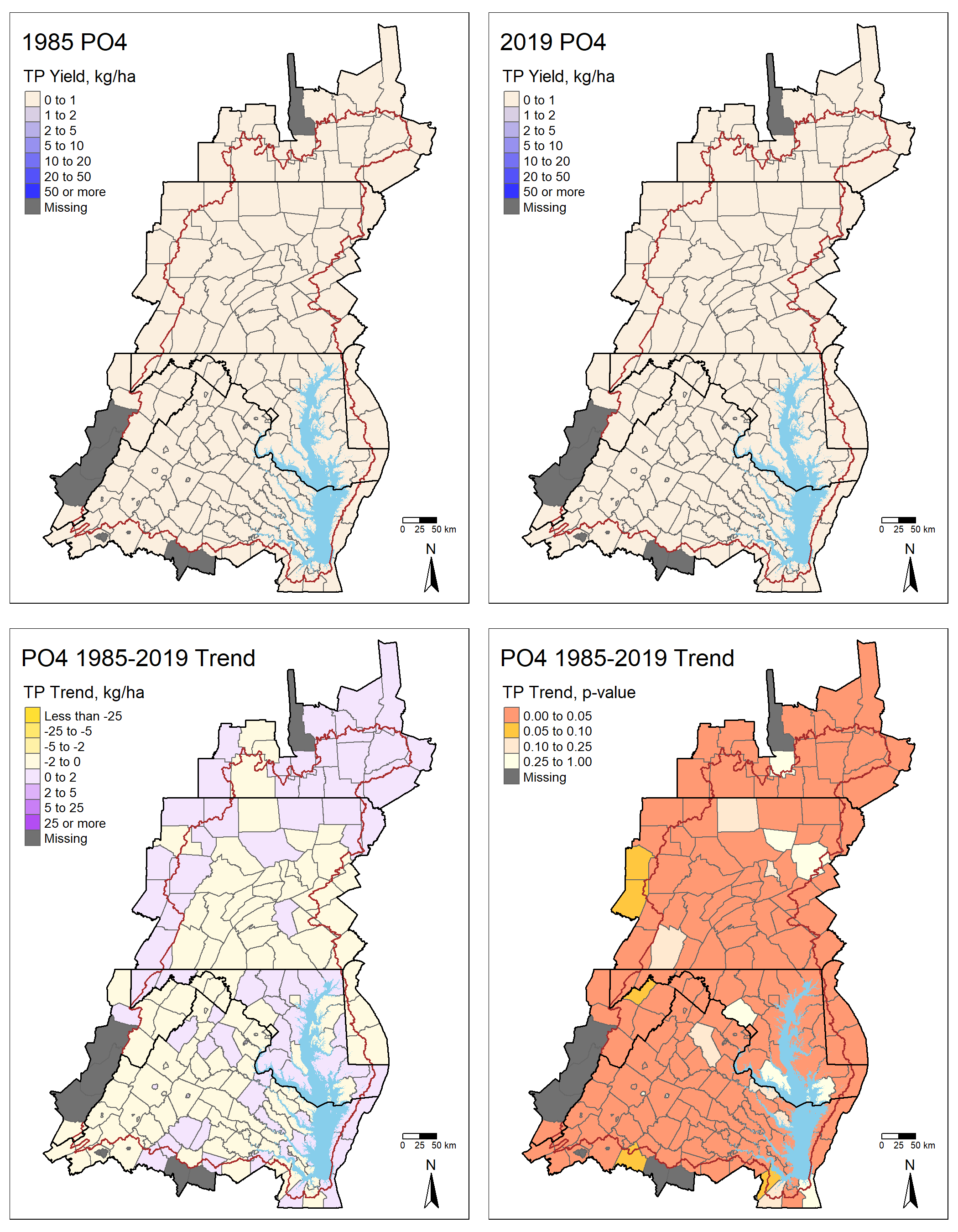
 Figure S77. For phosphorus, 1985 and 2019 atmospheric inorganic deposition (top row), the estimated Sen linear slope change in atmospheric inorganic deposition from 1985-2019 (bottom left), and the significance of trend results by county (bottom right).
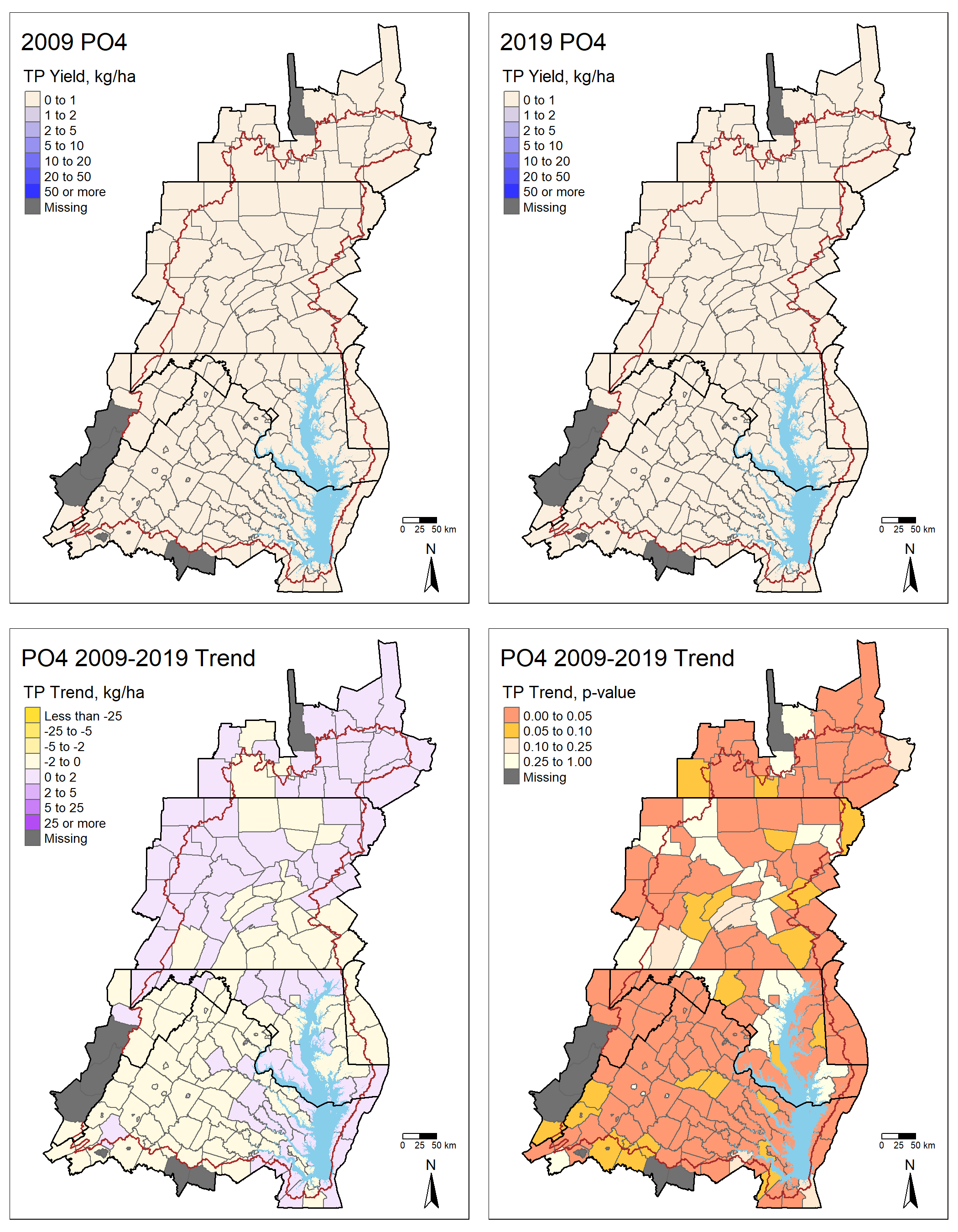
 Figure S78. For phosphorus, 2009 and 2019 atmospheric inorganic deposition (top row), the estimated Sen linear slope change in atmospheric inorganic deposition from 2009-2019 (bottom left), and the significance of trend results by county (bottom right).
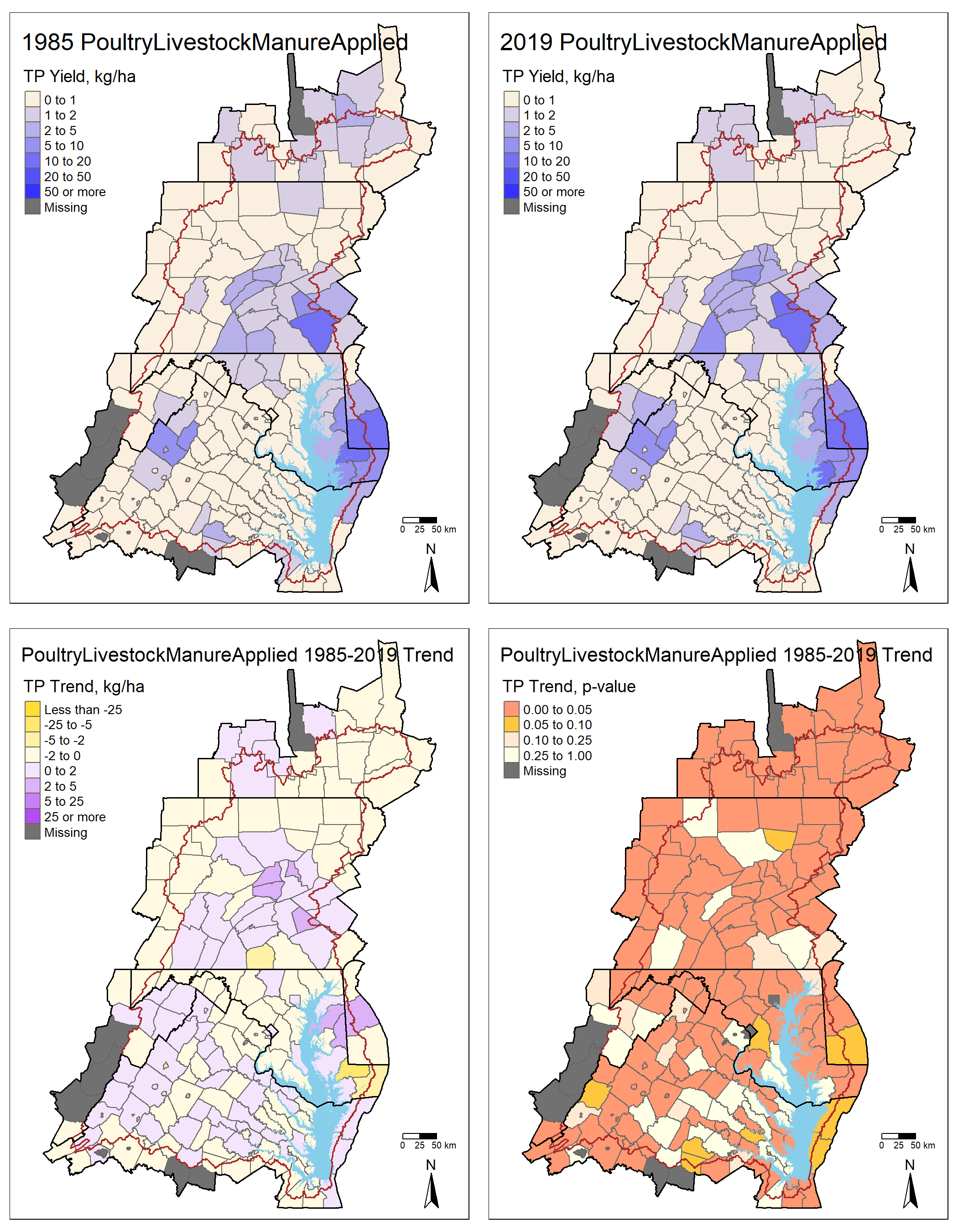
 Figure S79. For phosphorus, 1985 and 2019 poultry and livestock manure applied to agricultural land (top row), the estimated Sen linear slope change in poultry and livestock manure applied to agricultural land from 1985-2019 (bottom left), and the significance of trend results by county (bottom right).
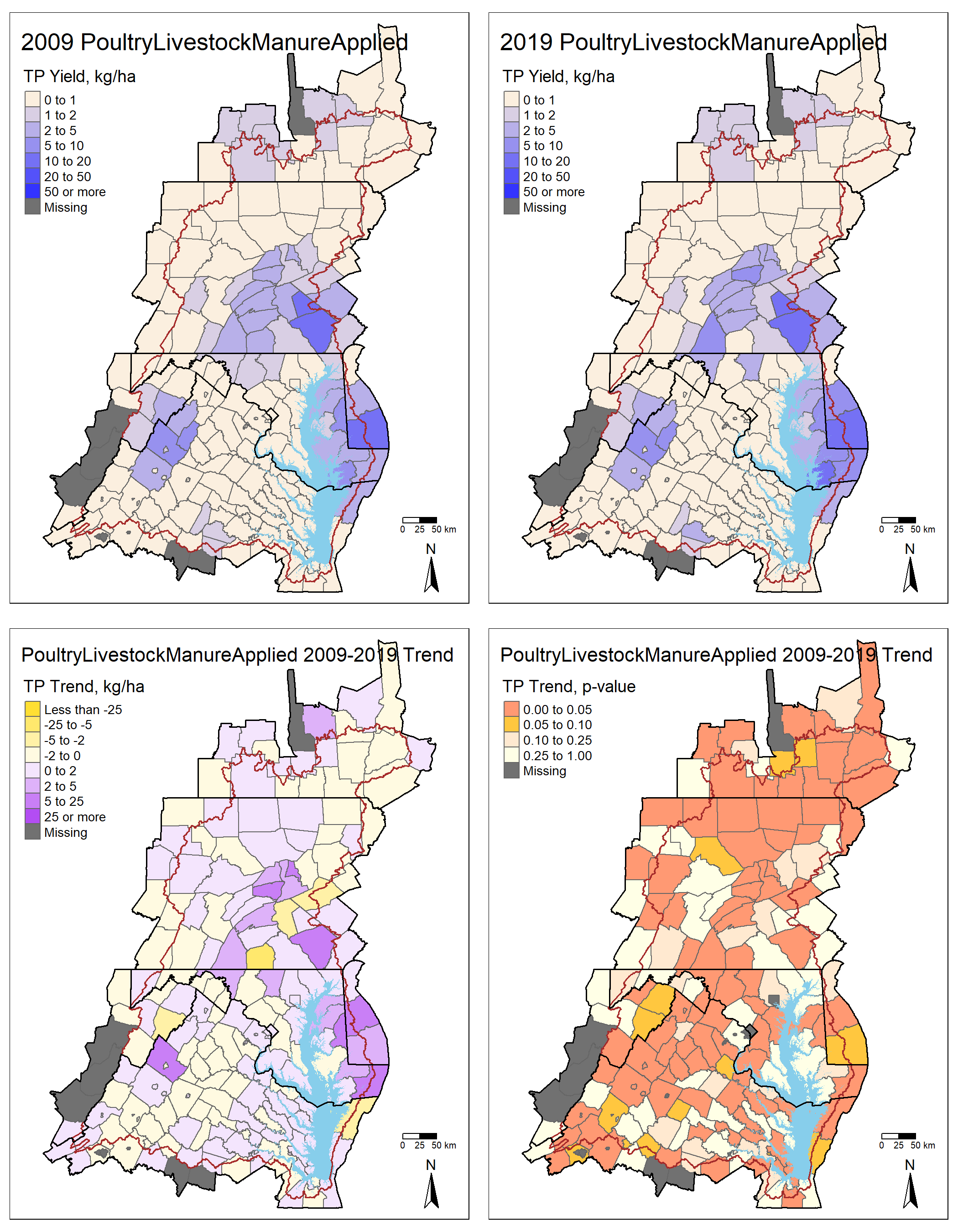
 Figure S80. For phosphorus, 2009 and 2019 poultry and livestock manure applied to agricultural land (top row), the estimated Sen linear slope change in poultry and livestock manure applied to agricultural land from 2009-2019 (bottom left), and the significance of trend results by county (bottom right).
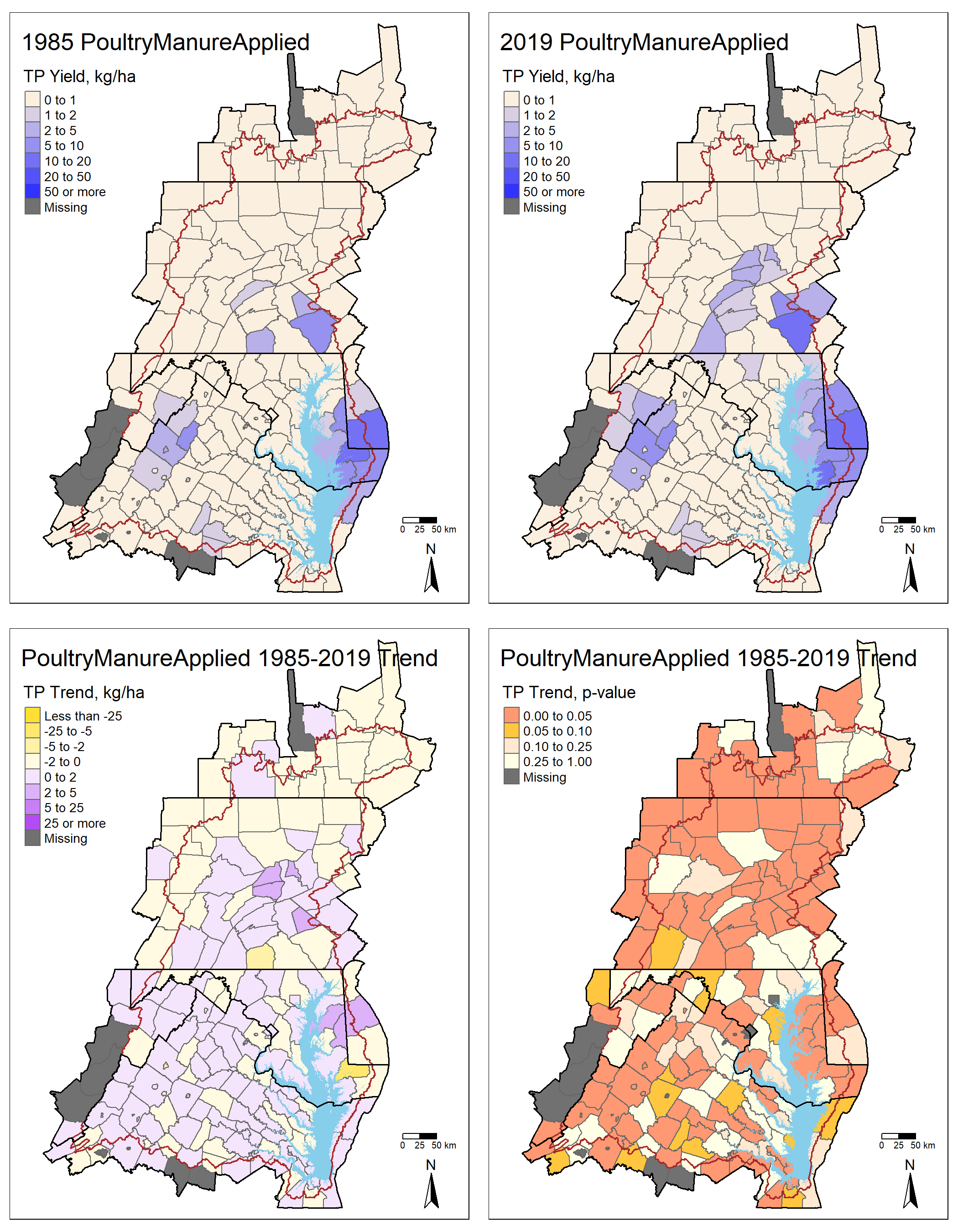
 Figure S81. For phosphorus, 1985 and 2019 poultry manure applied to agricultural land (top row), the estimated Sen linear slope change in poultry manure applied to agricultural land from 1985-2019 (bottom left), and the significance of trend results by county (bottom right).
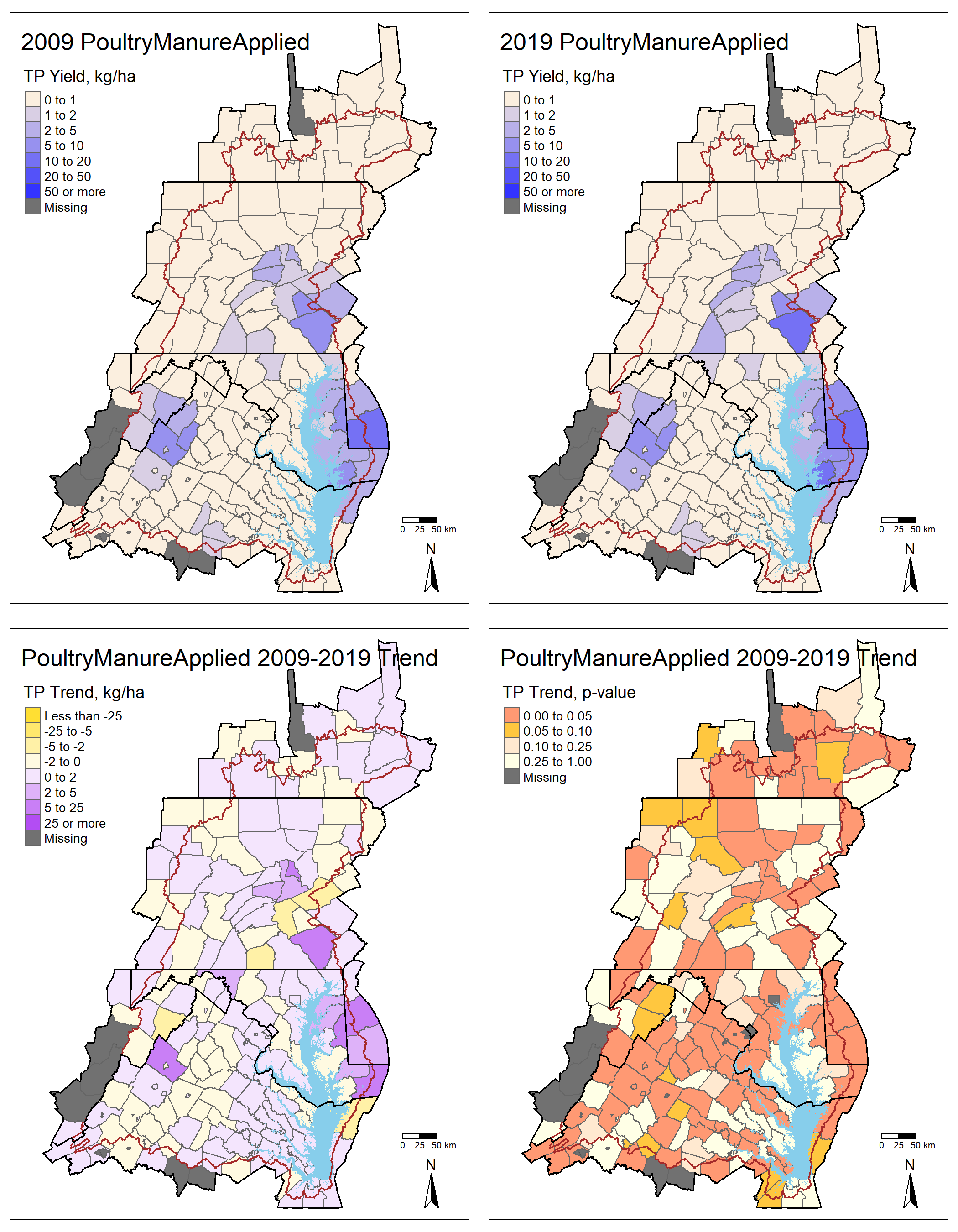
 Figure S82. For phosphorus, 2009 and 2019 poultry manure applied to agricultural land (top row), the estimated Sen linear slope change in poultry manure applied to agricultural land from 2009-2019 (bottom left), and the significance of trend results by county (bottom right).
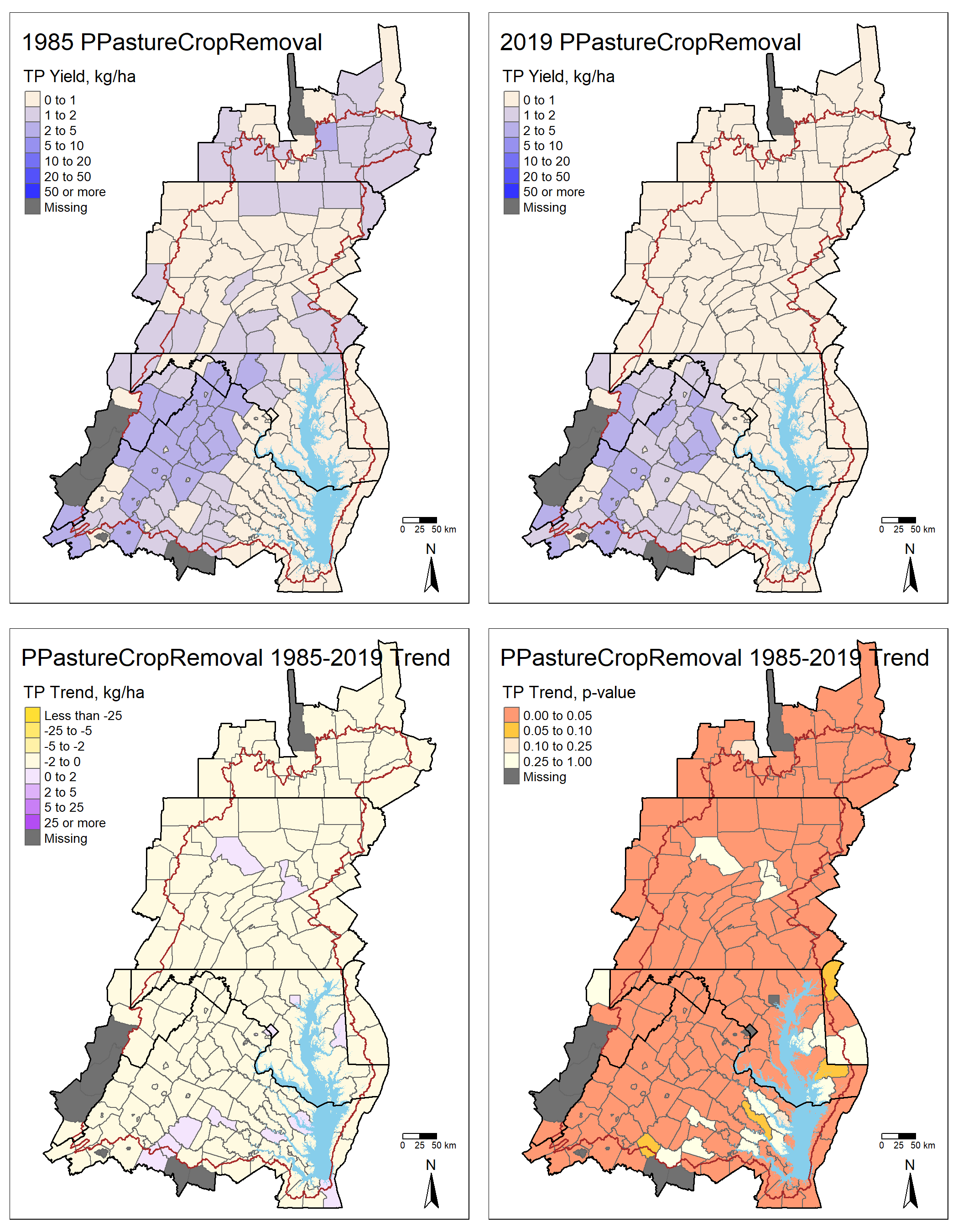
 Figure S83. For phosphorus, 1985 and 2019 pasture removal (top row), the estimated Sen linear slope change in pasture removal from 1985-2019 (bottom left), and the significance of trend results by county (bottom right).
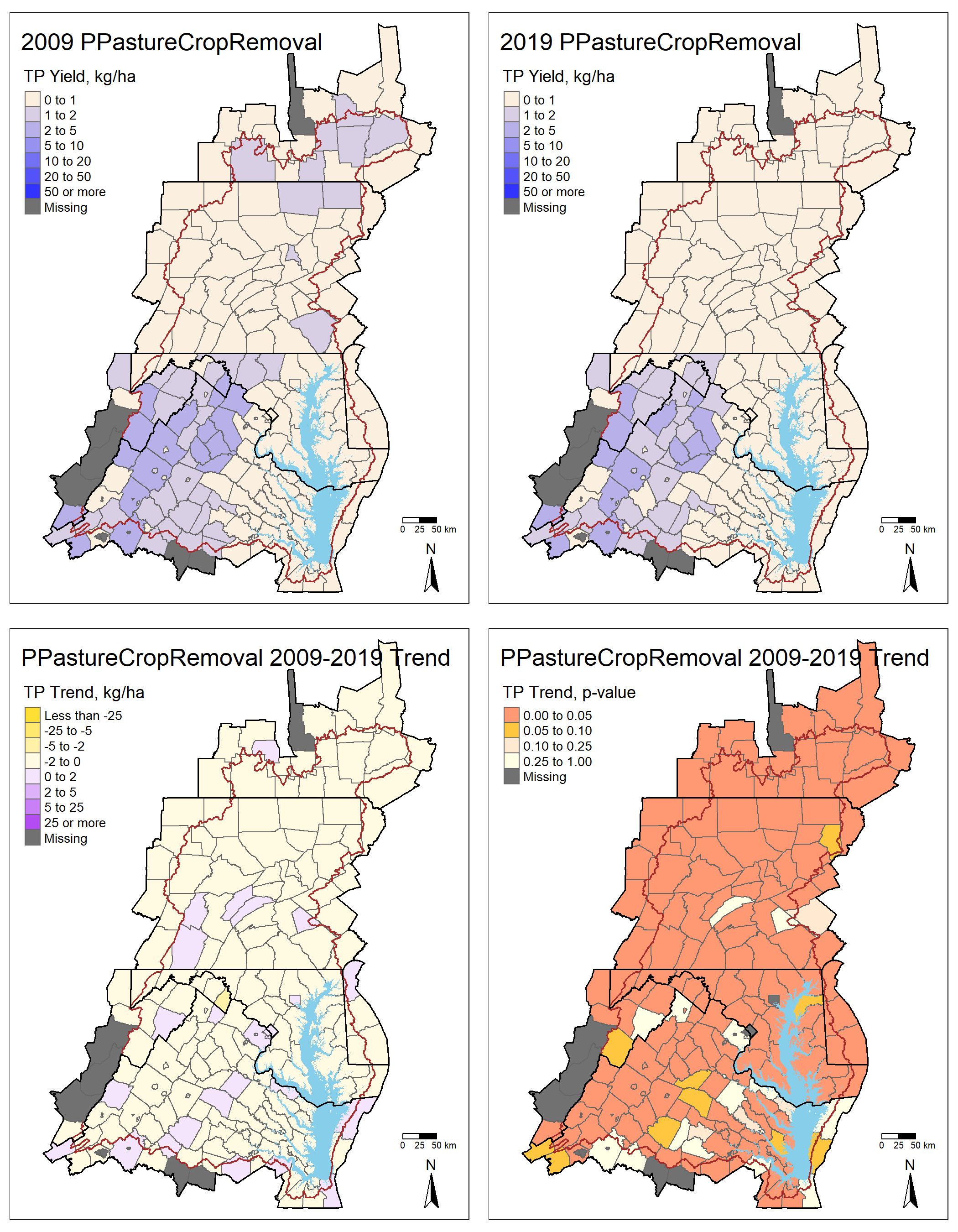
 Figure S84. For phosphorus, 2009 and 2019 pasture removal (top row), the estimated Sen linear slope change in pasture removal from 2009-2019 (bottom left), and the significance of trend results by county (bottom right).
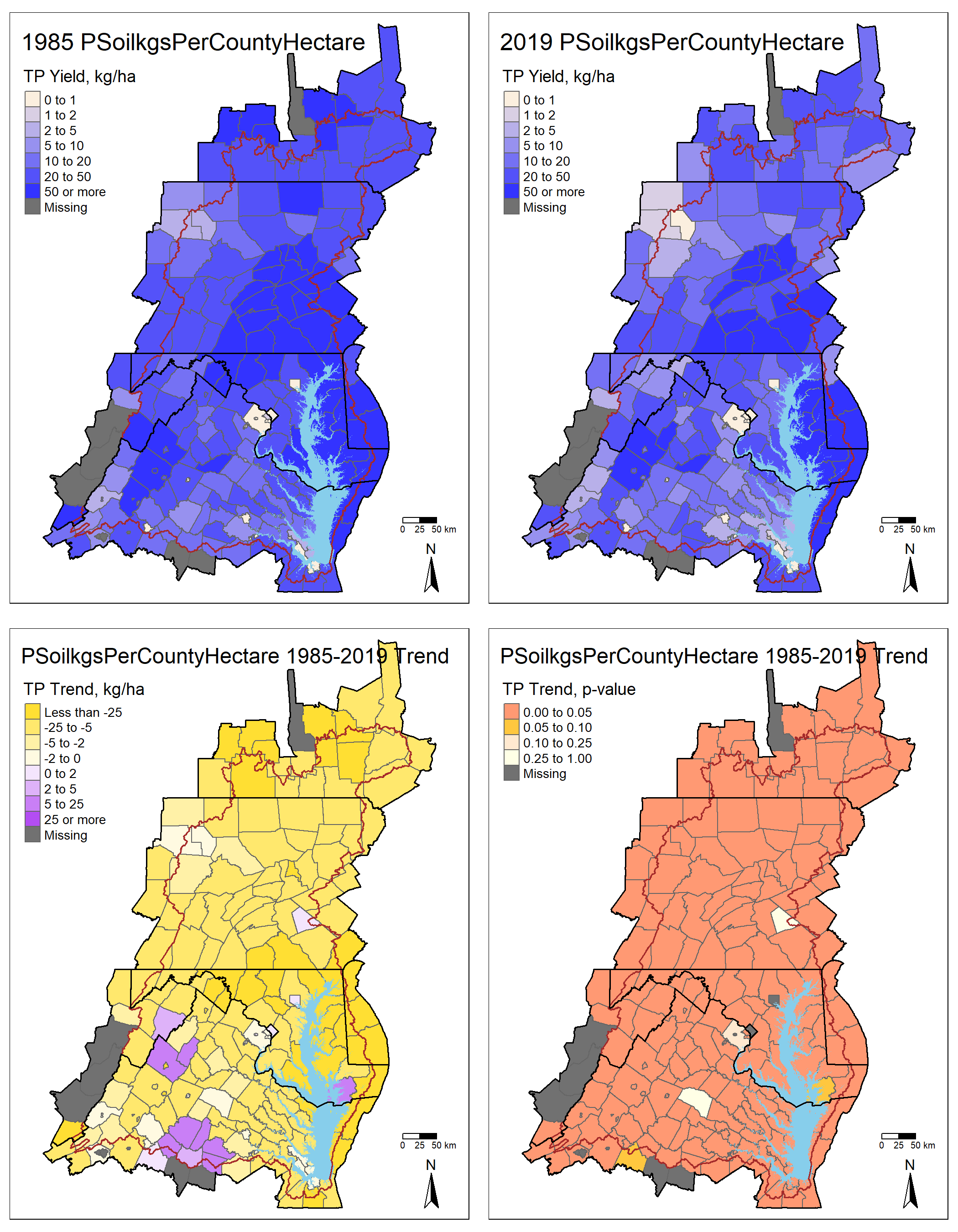
 Figure S85. For phosphorus, 1985 and 2019 agricultural soil phosphorus per million kilograms of soil (top row), the estimated Sen linear slope change in agricultural soil phosphorus per million kilograms of soil from 1985-2019 (bottom left), and the significance of trend results by county (bottom right).
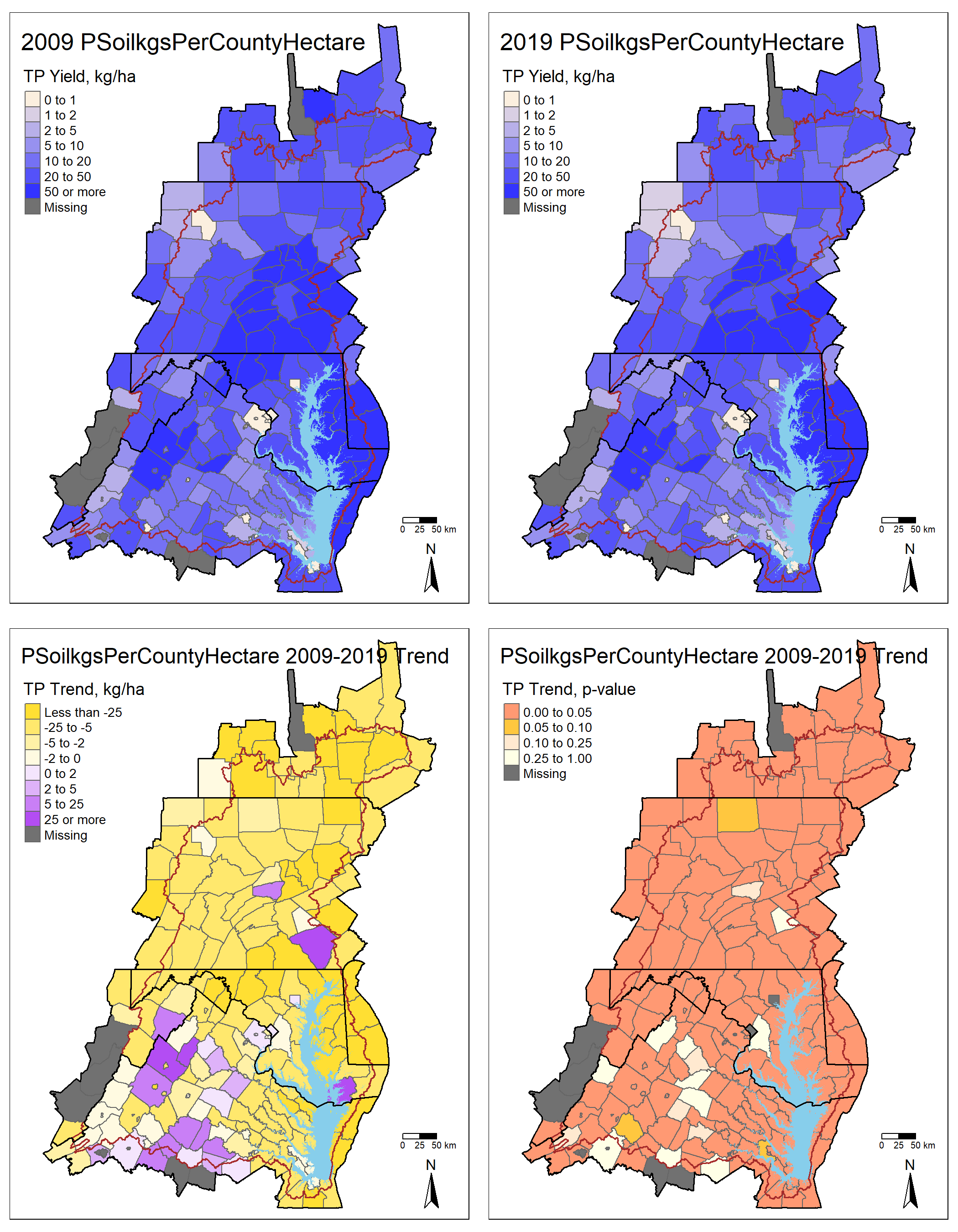
 Figure S86. For phosphorus, 2009 and 2019 agricultural soil phosphorus per million kilograms of soil (top row), the estimated Sen linear slope change in agricultural soil phosphorus per million kilograms of soil from 2009-2019 (bottom left), and the significance of trend results by county (bottom right).
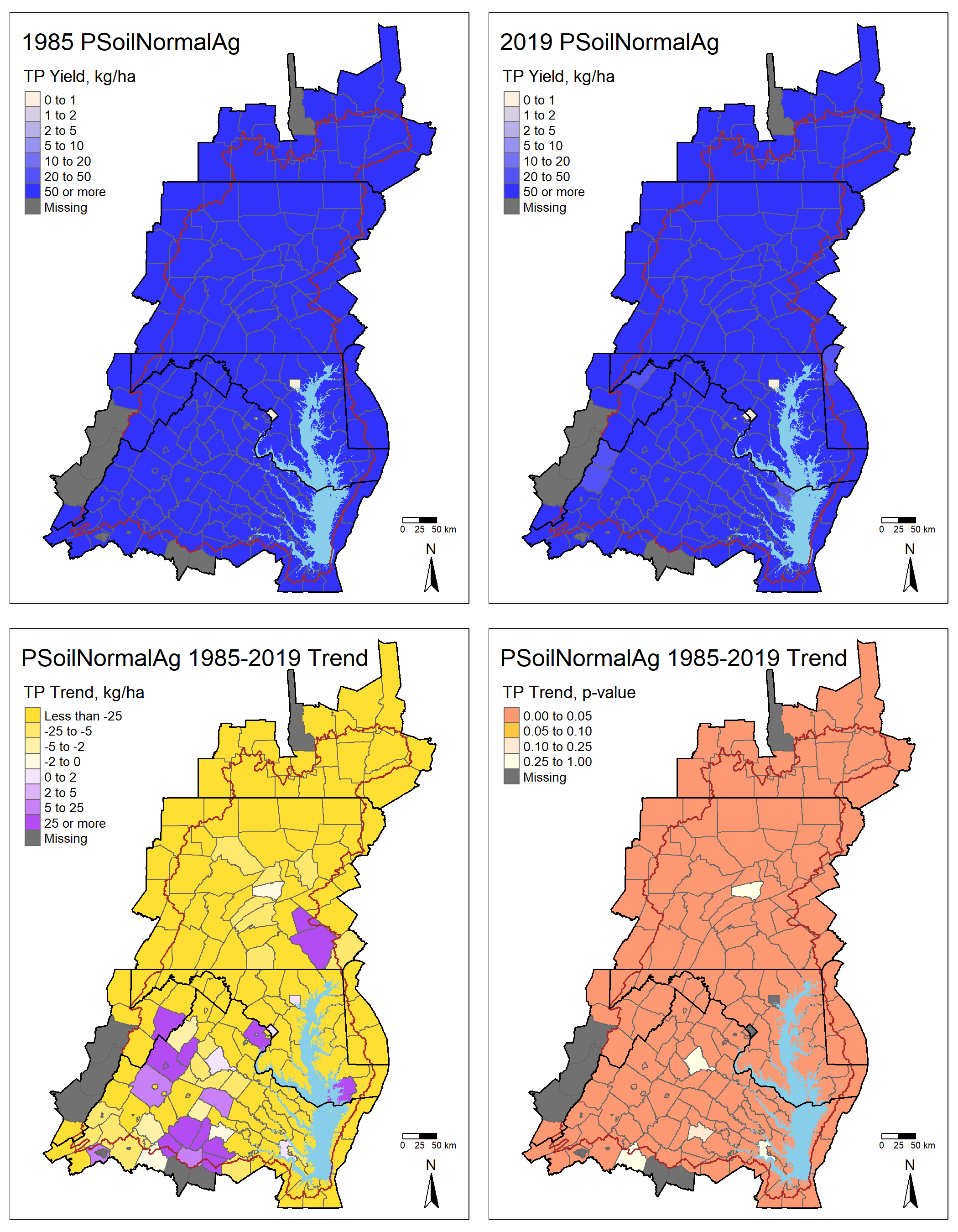
 Figure S87. For phosphorus, 1985 and 2019 agricultural soil phosphorus pools (top row), the estimated Sen linear slope change in agricultural soil phosphorus pools from 1985-2019 (bottom left), and the significance of trend results by county (bottom right).
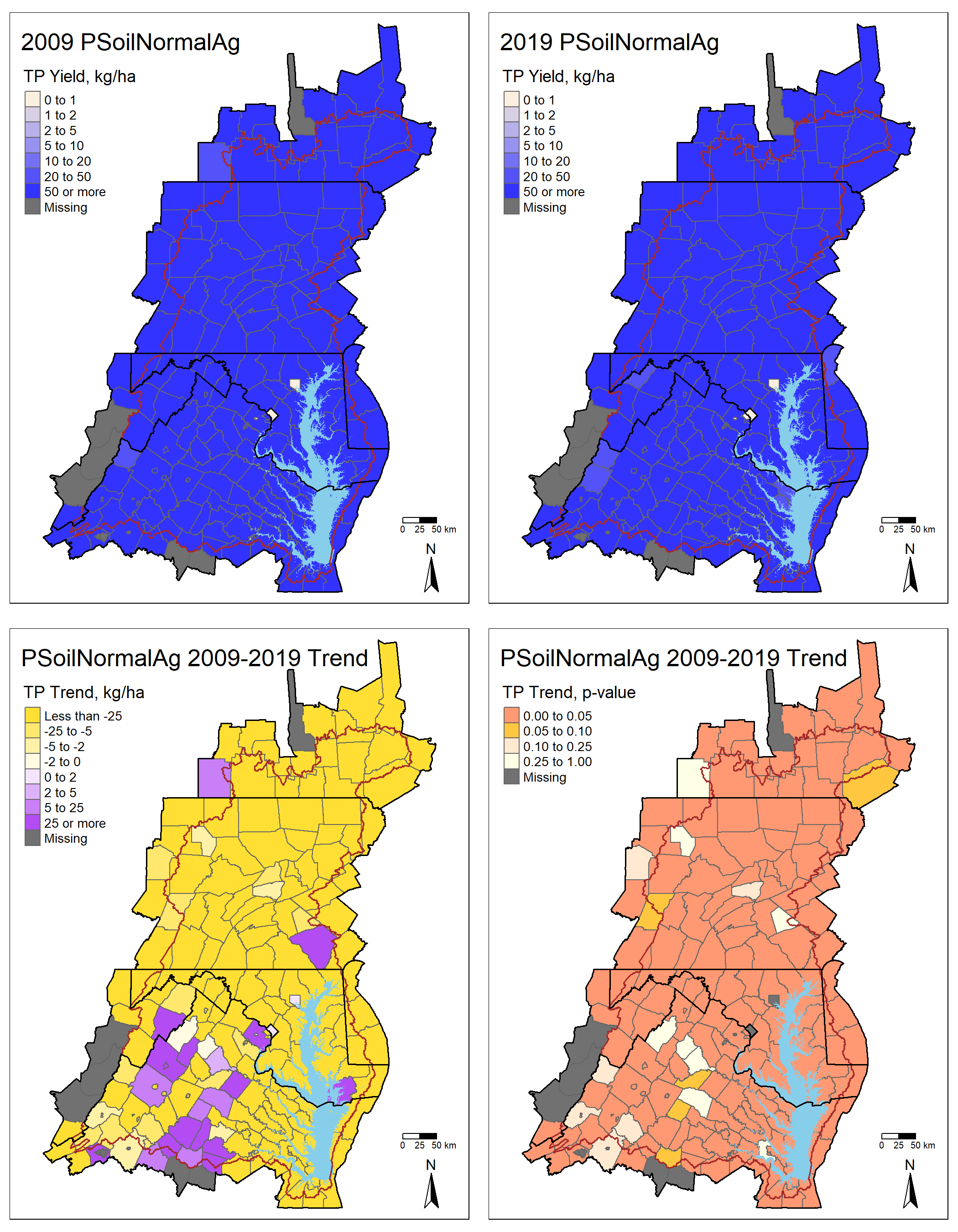
 Figure S88. For phosphorus, 2009 and 2019 agricultural soil phosphorus pools (top row), the estimated Sen linear slope change in agricultural soil phosphorus pools from 2009-2019 (bottom left), and the significance of trend results by county (bottom right).
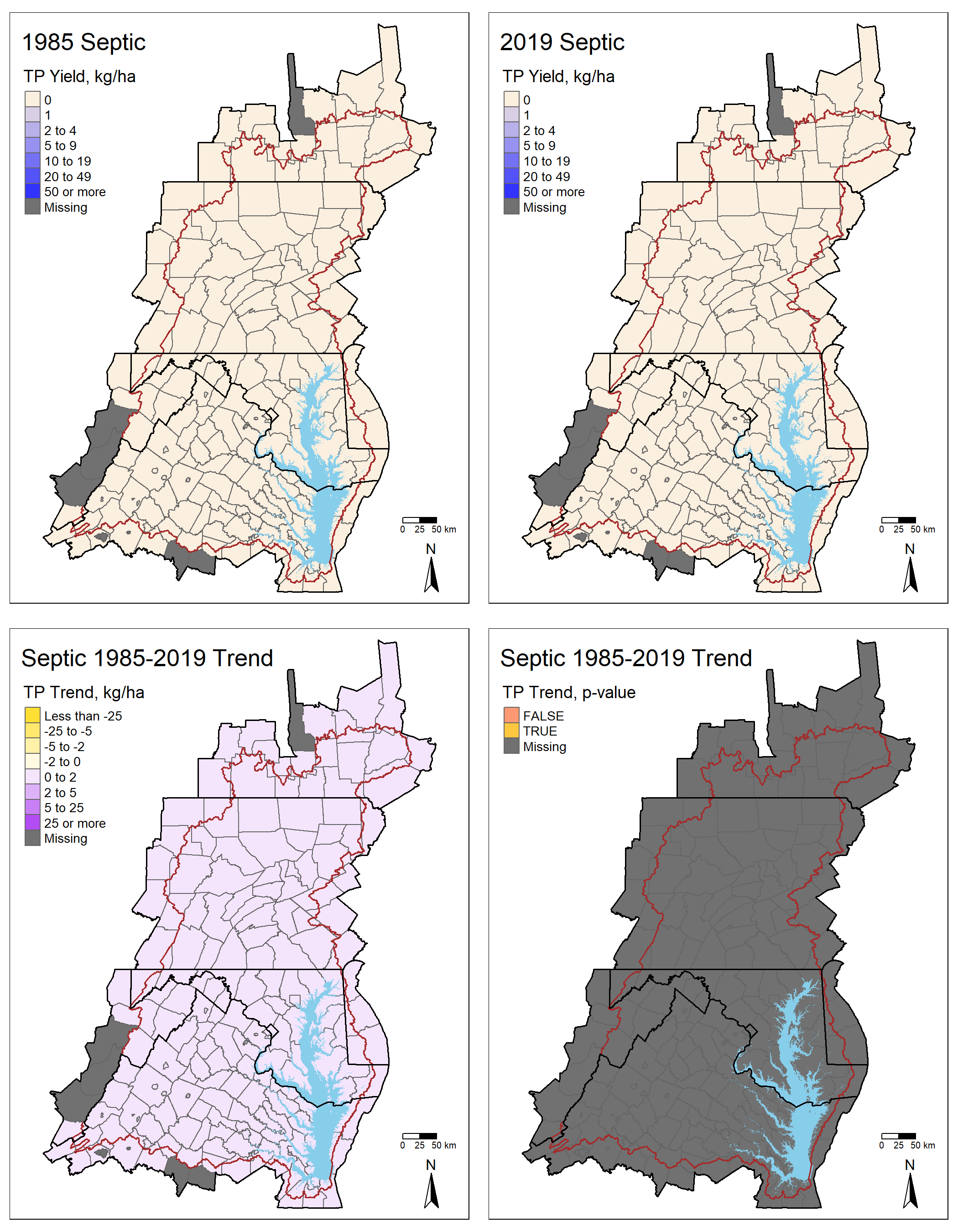
 Figure S89. For phosphorus, 1985 and 2019 septic loads (top row), the estimated Sen linear slope change in septic loads from 1985-2019 (bottom left), and the significance of trend results by county (bottom right).
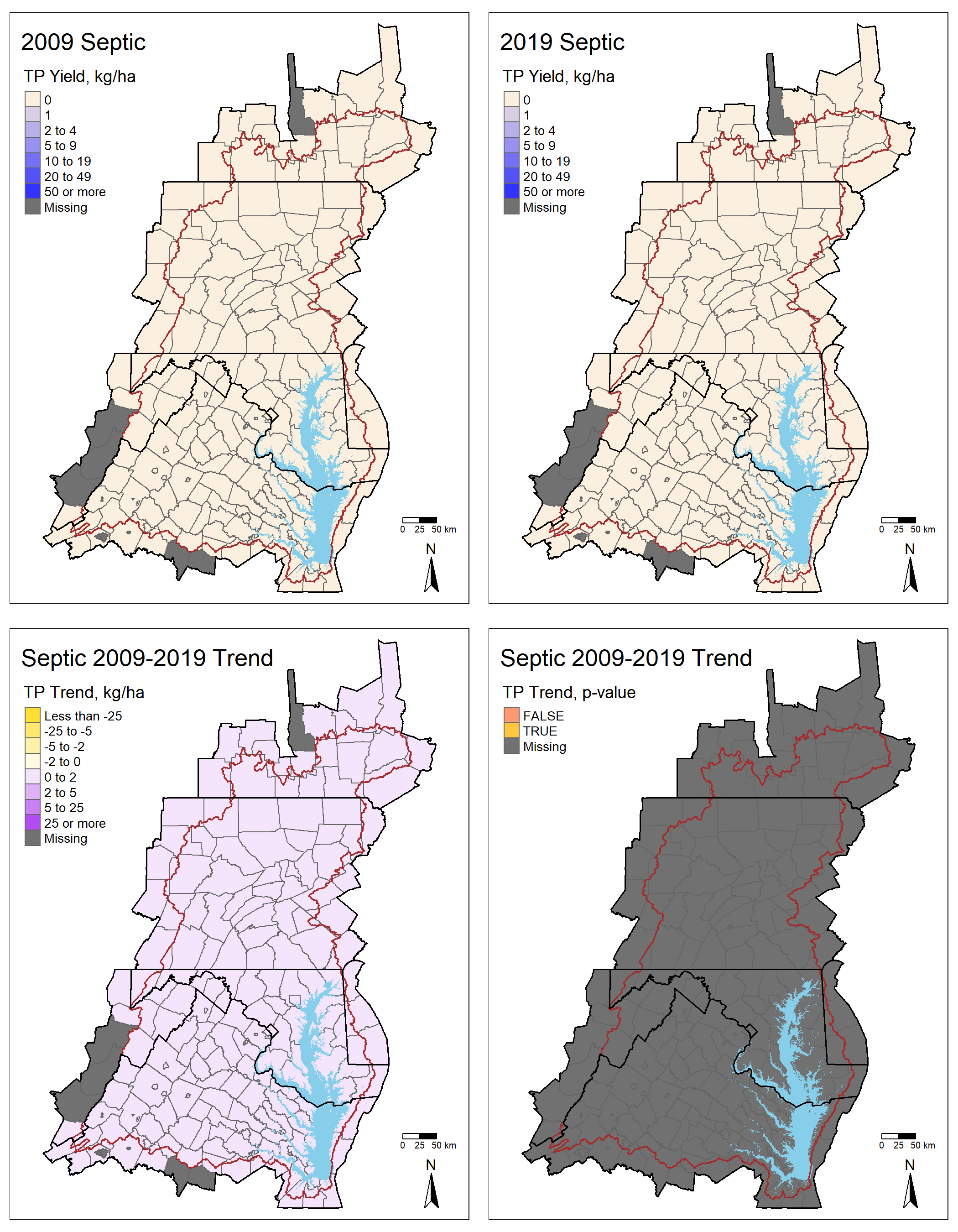
 Figure S90. For phosphorus, 2009 and 2019 septic loads (top row), the estimated Sen linear slope change in septic loads from 2009-2019 (bottom left), and the significance of trend results by county (bottom right).
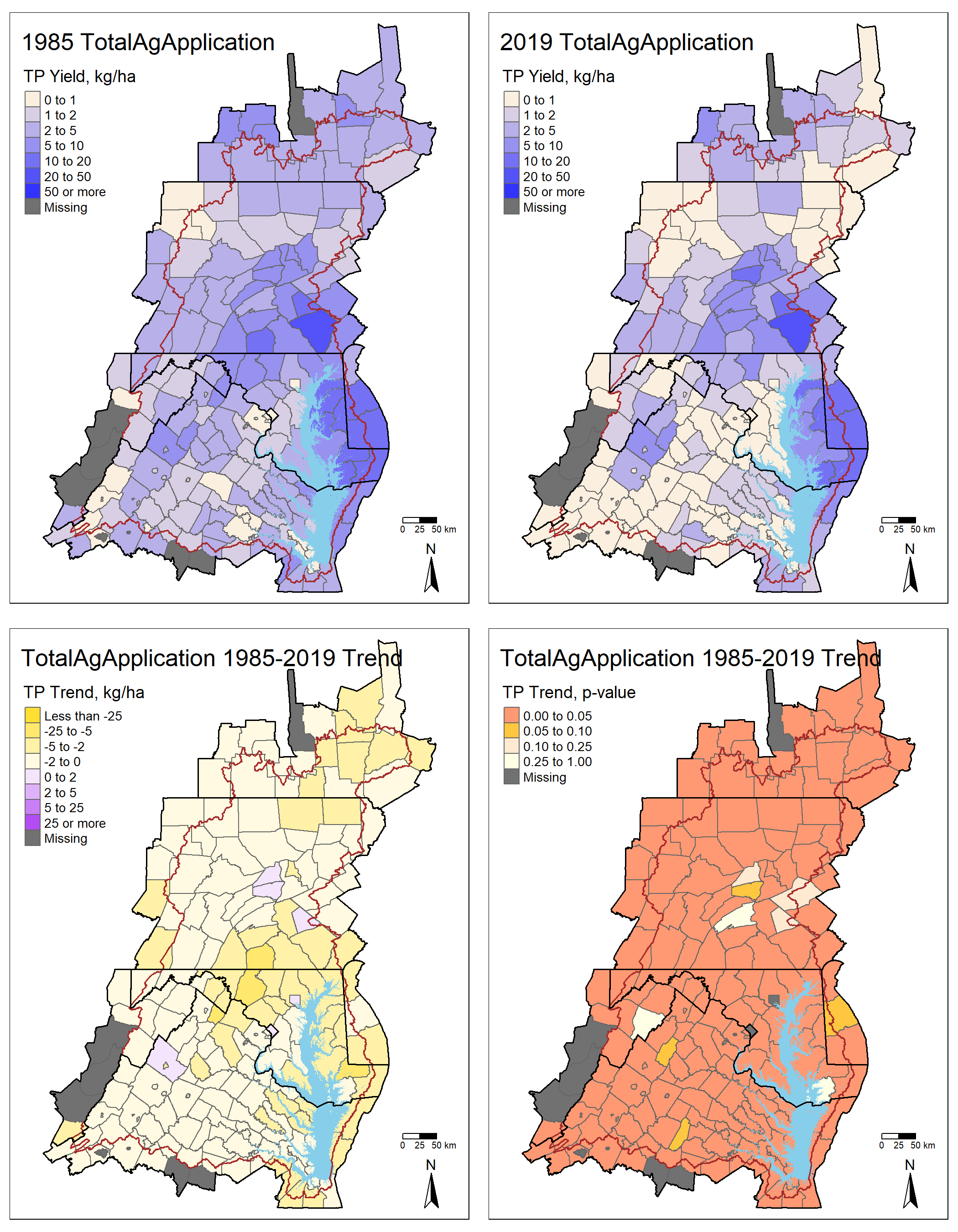
 Figure S91. For phosphorus, 1985 and 2019 total agricultural application (top row), the estimated Sen linear slope change in total agricultural application from 1985-2019 (bottom left), and the significance of trend results by county (bottom right).
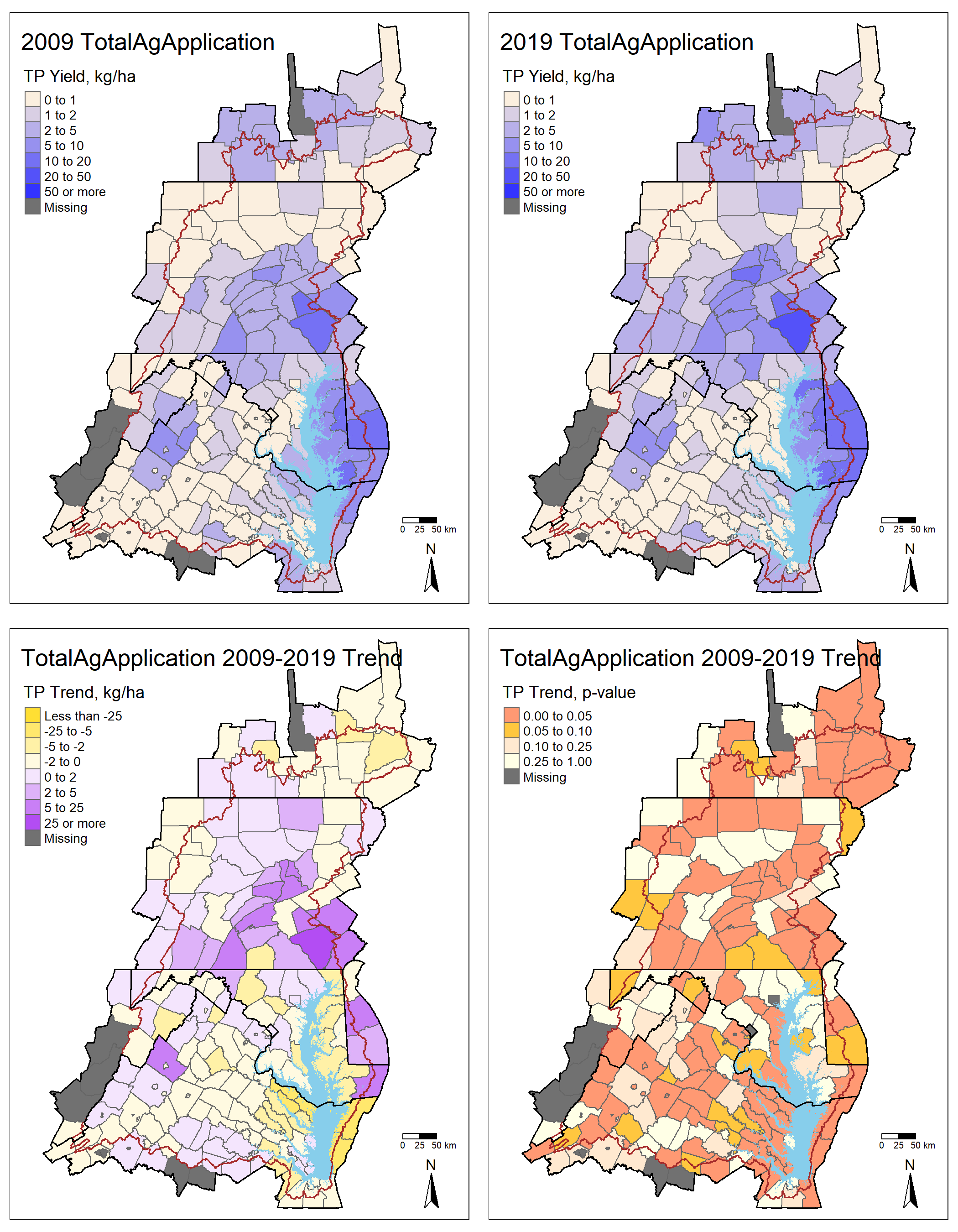
 Figure S92. For phosphorus, 2009 and 2019 total agricultural application (top row), the estimated Sen linear slope change in total agricultural application from 2009-2019 (bottom left), and the significance of trend results by county (bottom right).
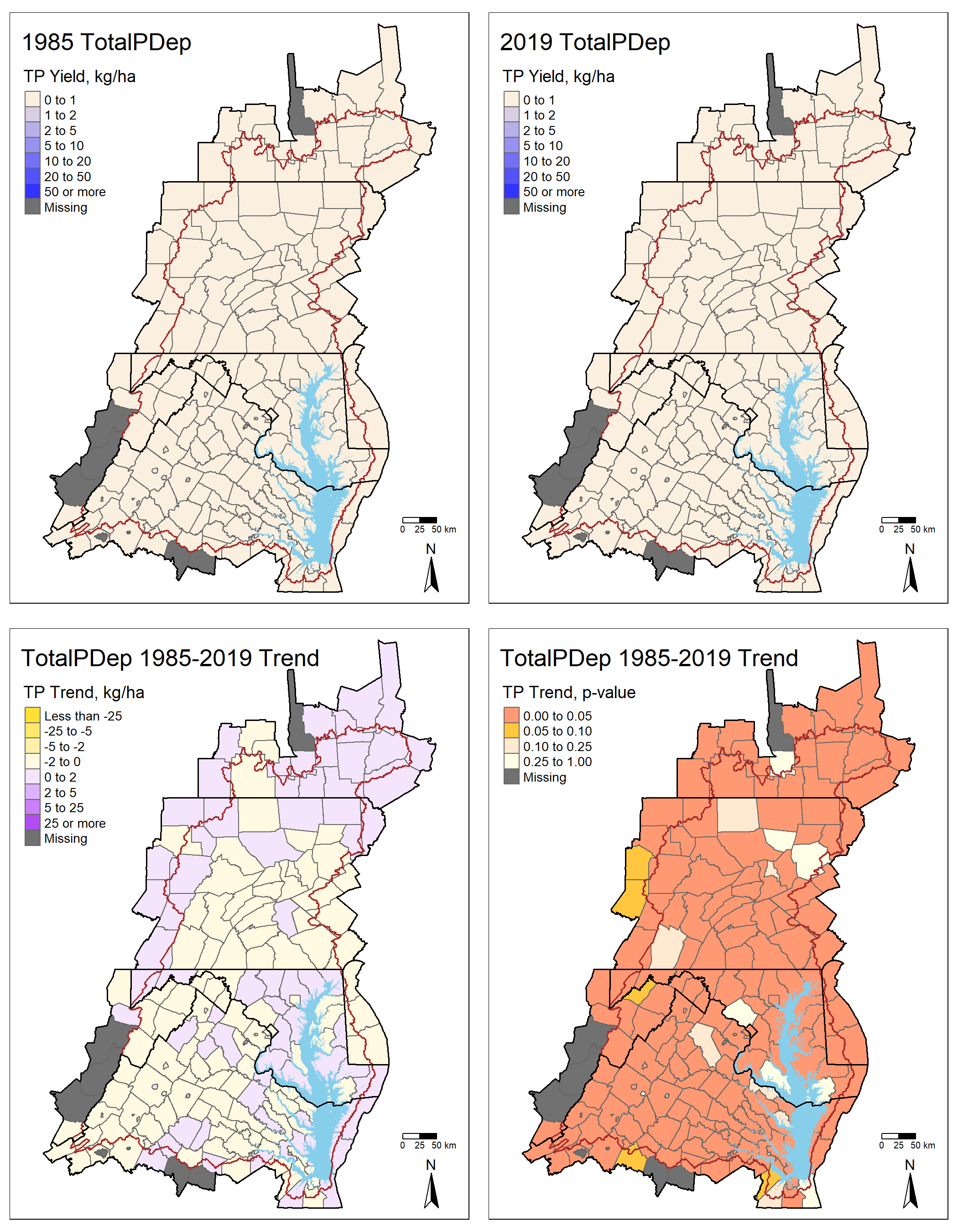
 Figure S93. For phosphorus, 1985 and 2019 total atmospheric deposition (top row), the estimated Sen linear slope change in total atmospheric deposition from 1985-2019 (bottom left), and the significance of trend results by county (bottom right).
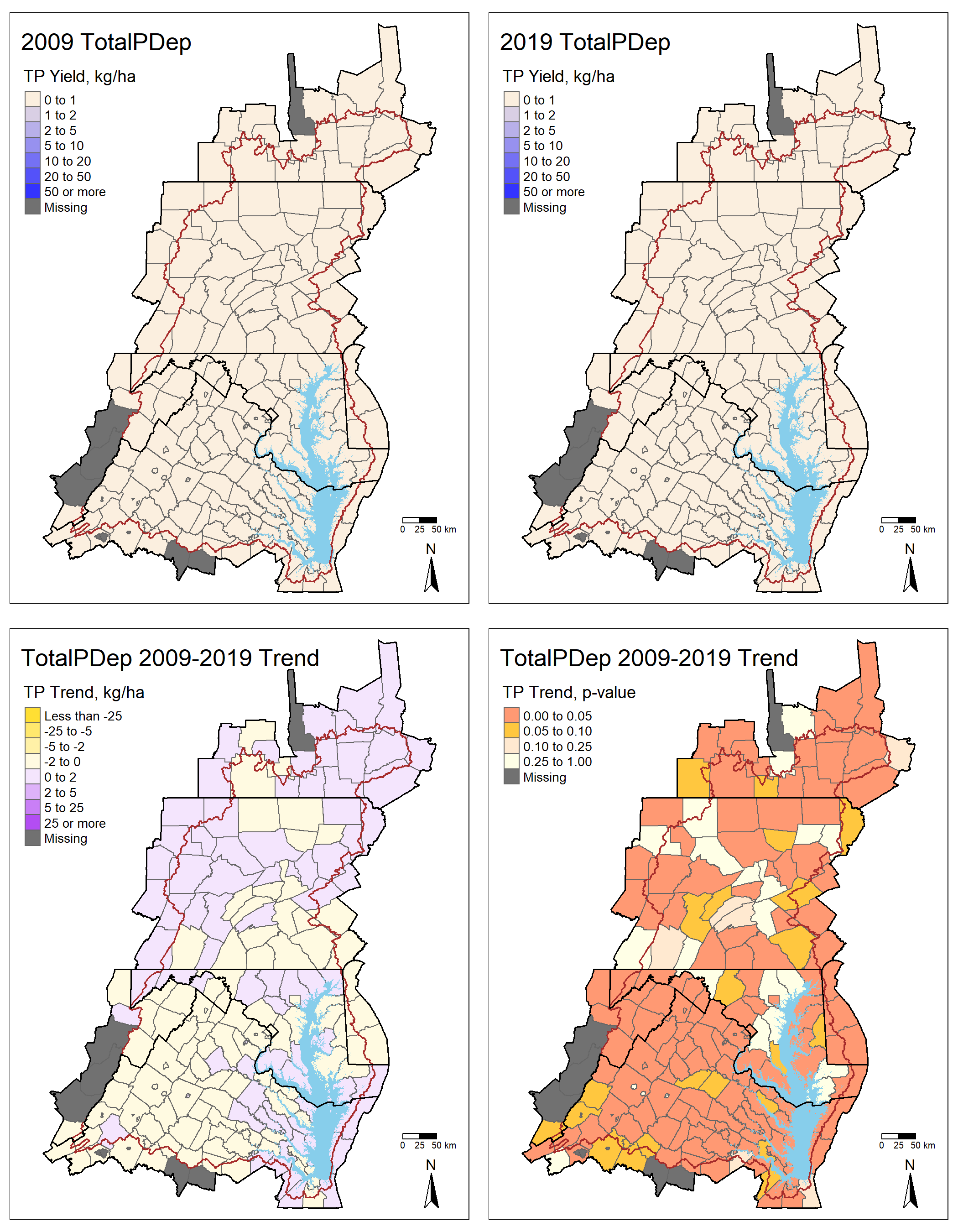
 Figure S94. For phosphorus, 2009 and 2019 total atmospheric deposition (top row), the estimated Sen linear slope change in total atmospheric deposition from 2009-2019 (bottom left), and the significance of trend results by county (bottom right).
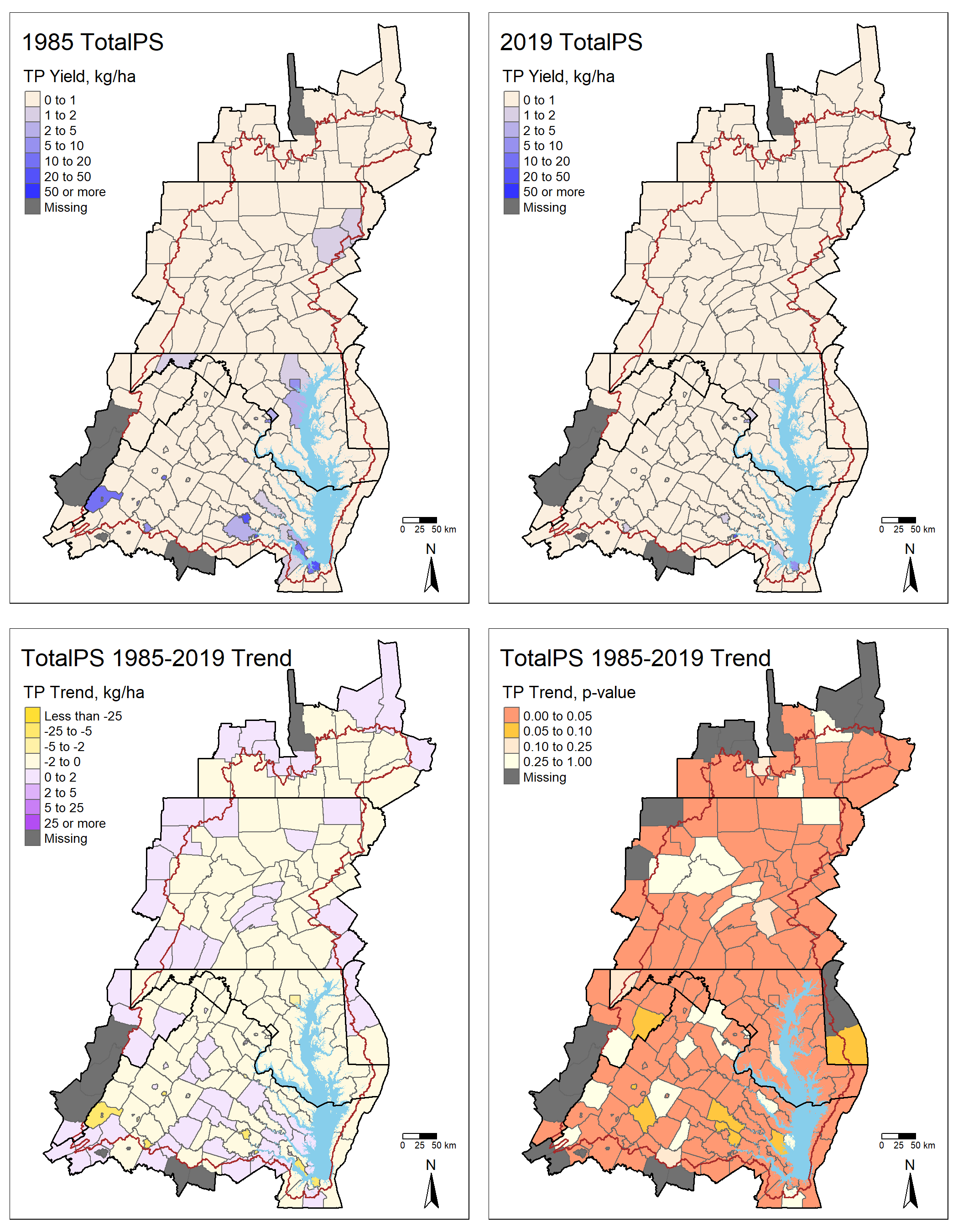
 Figure S95. For phosphorus, 1985 and 2019 total point source loads (top row), the estimated Sen linear slope change in total point source loads from 1985-2019 (bottom left), and the significance of trend results by county (bottom right).
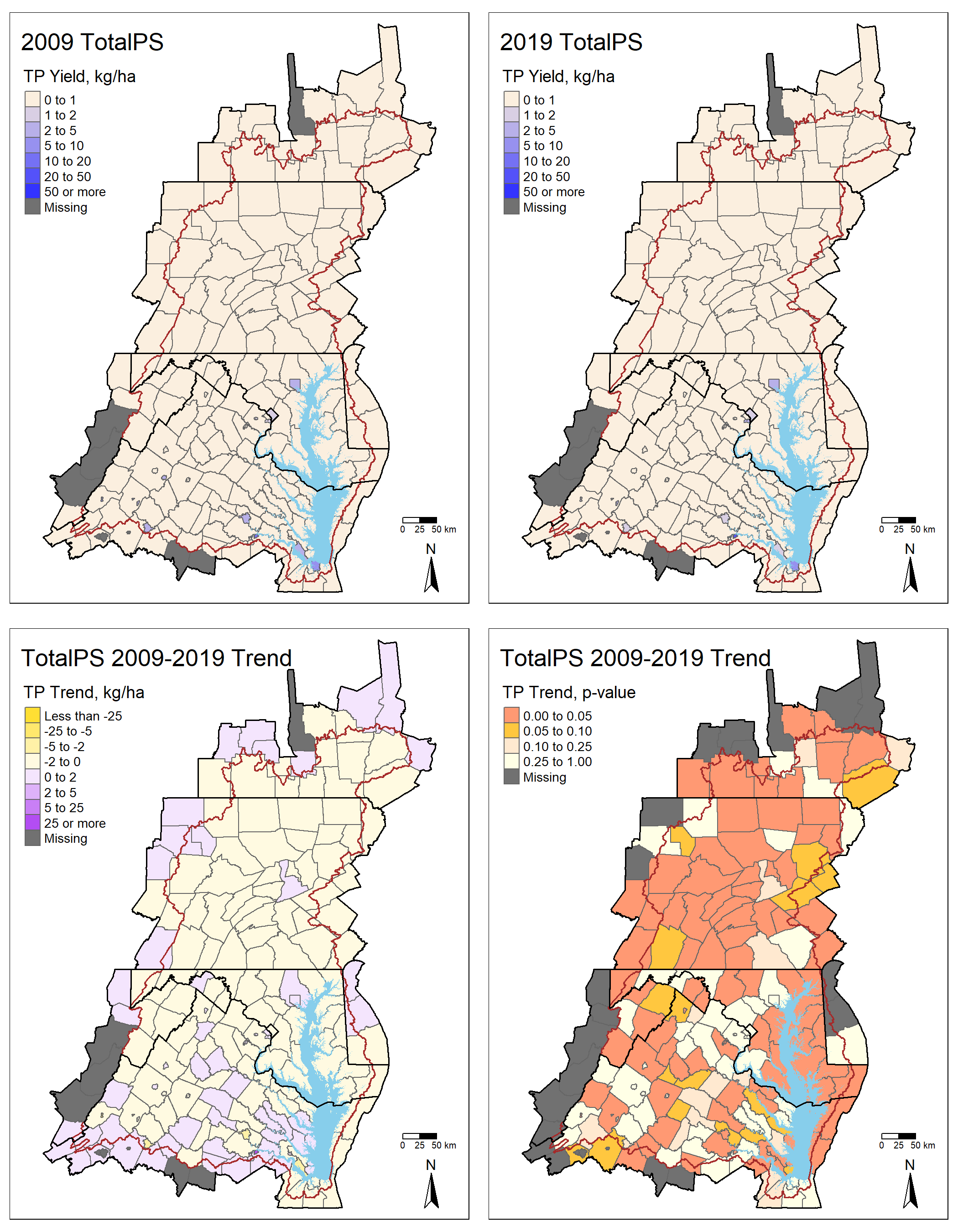
 Figure S96. For phosphorus, 2009 and 2019 total point source loads (top row), the estimated Sen linear slope change in total point source loads from 2009-2019 (bottom left), and the significance of trend results by county (bottom right).
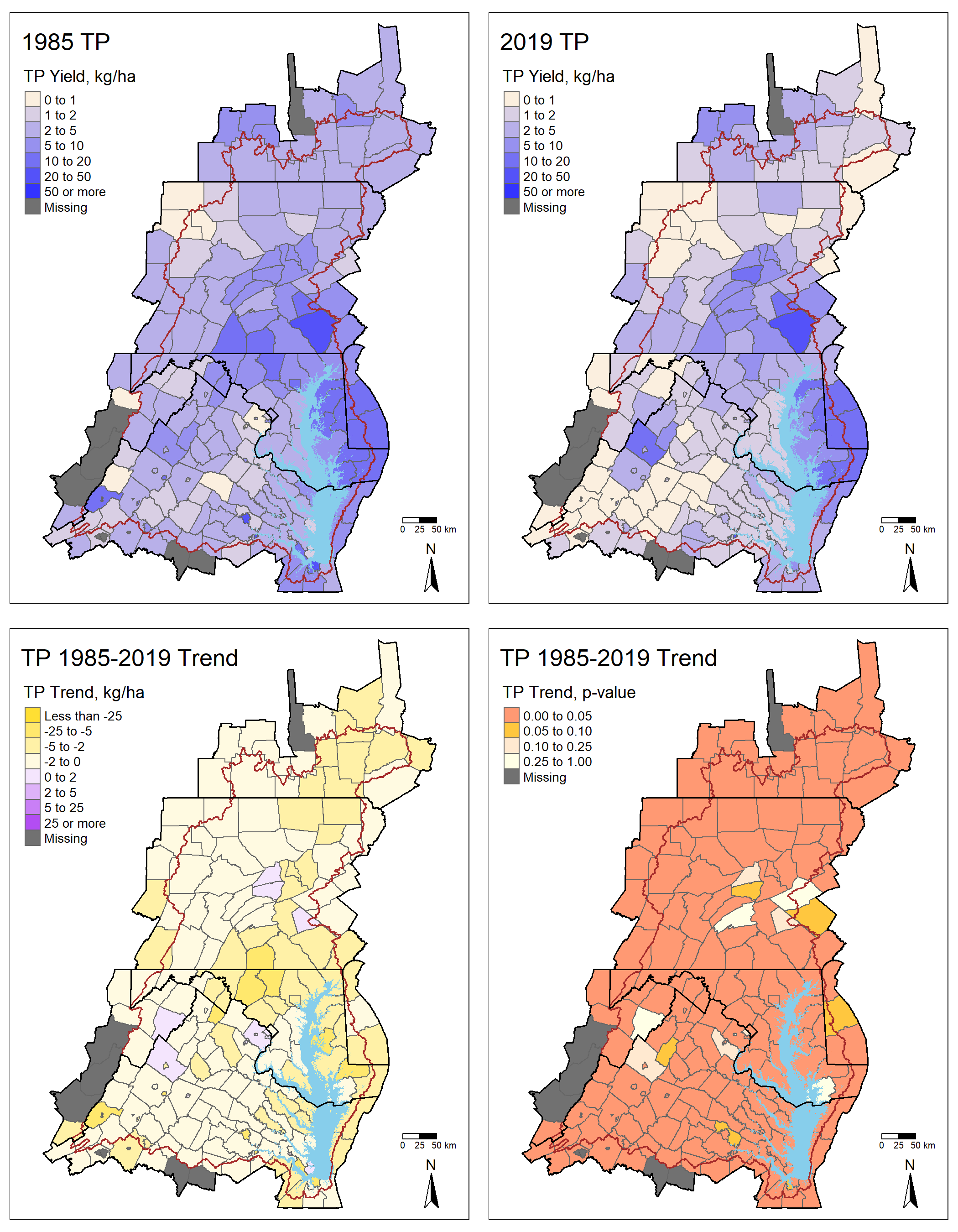
 Figure S97. For phosphorus, 1985 and 2019 total inputs (top row), the estimated Sen linear slope change in total inputs from 1985-2019 (bottom left), and the significance of trend results by county (bottom right).
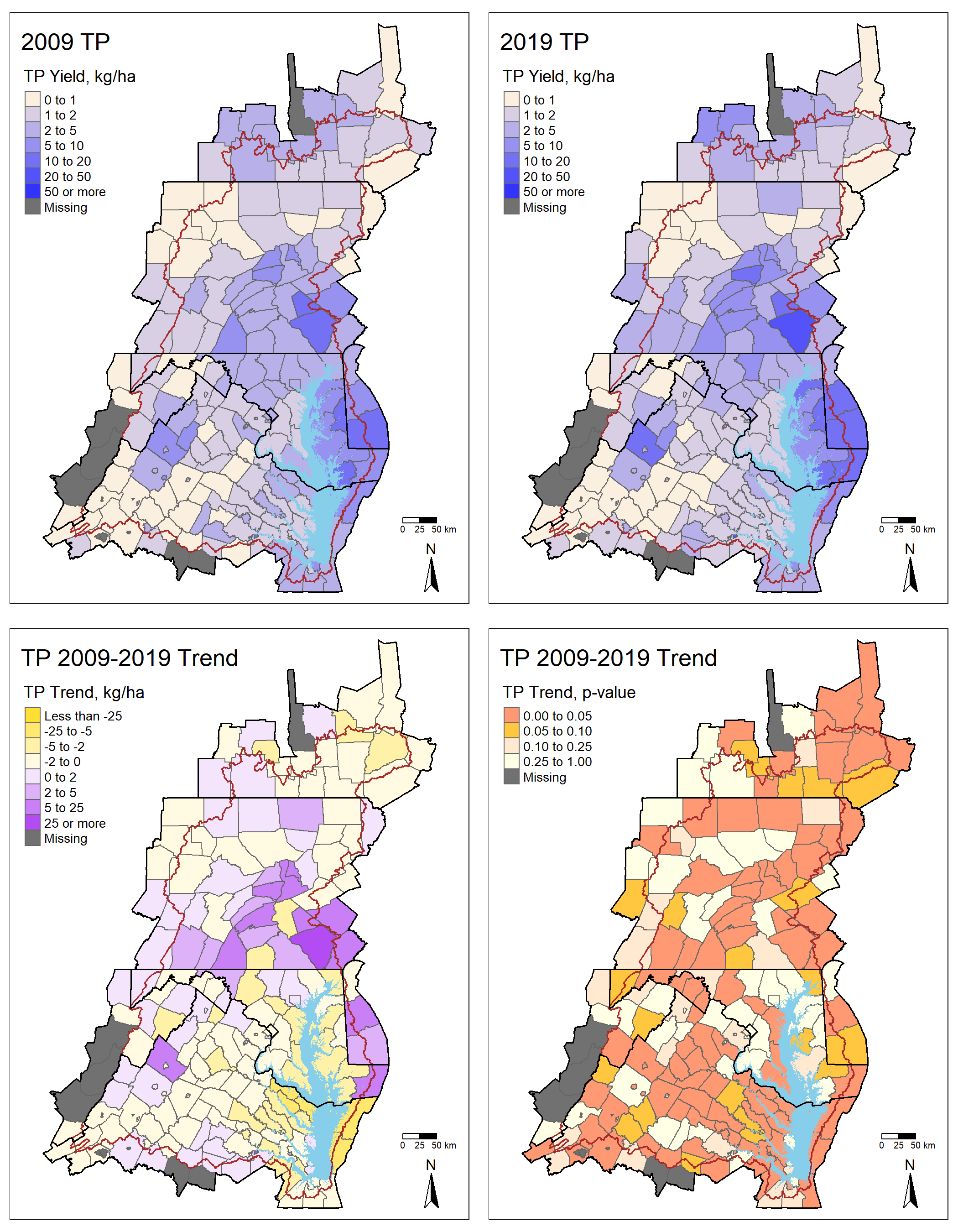
 Figure S98. For phosphorus, 2009 and 2019 total inputs (top row), the estimated Sen linear slope change in total nitrogen from 2009-2019 (bottom left), and the significance of trend results by county (bottom right).
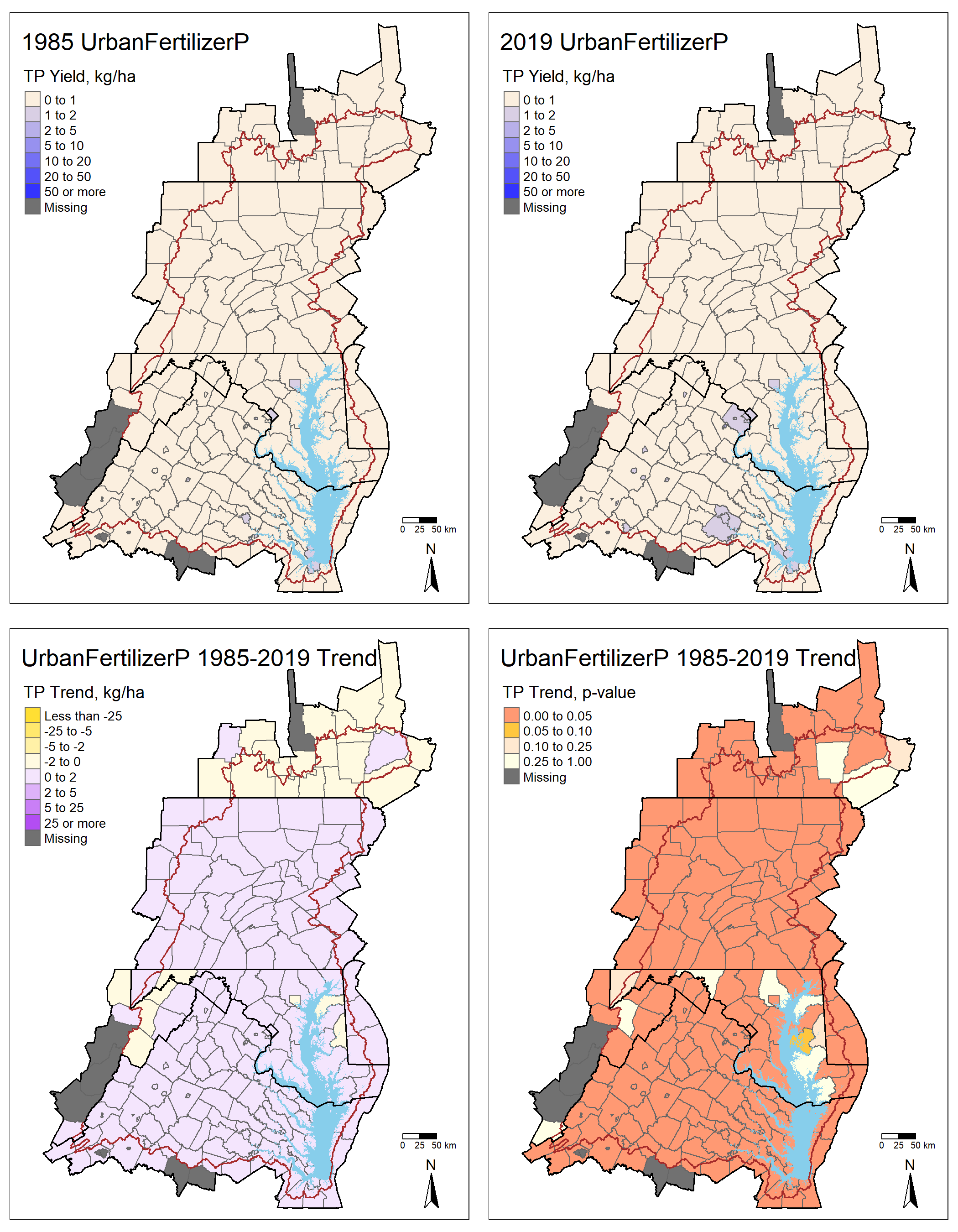
 Figure S99. For phosphorus, 1985 and 2019 urban fertilizer (top row), the estimated Sen linear slope change in urban fertilizer from 1985-2019 (bottom left), and the significance of trend results by county (bottom right).
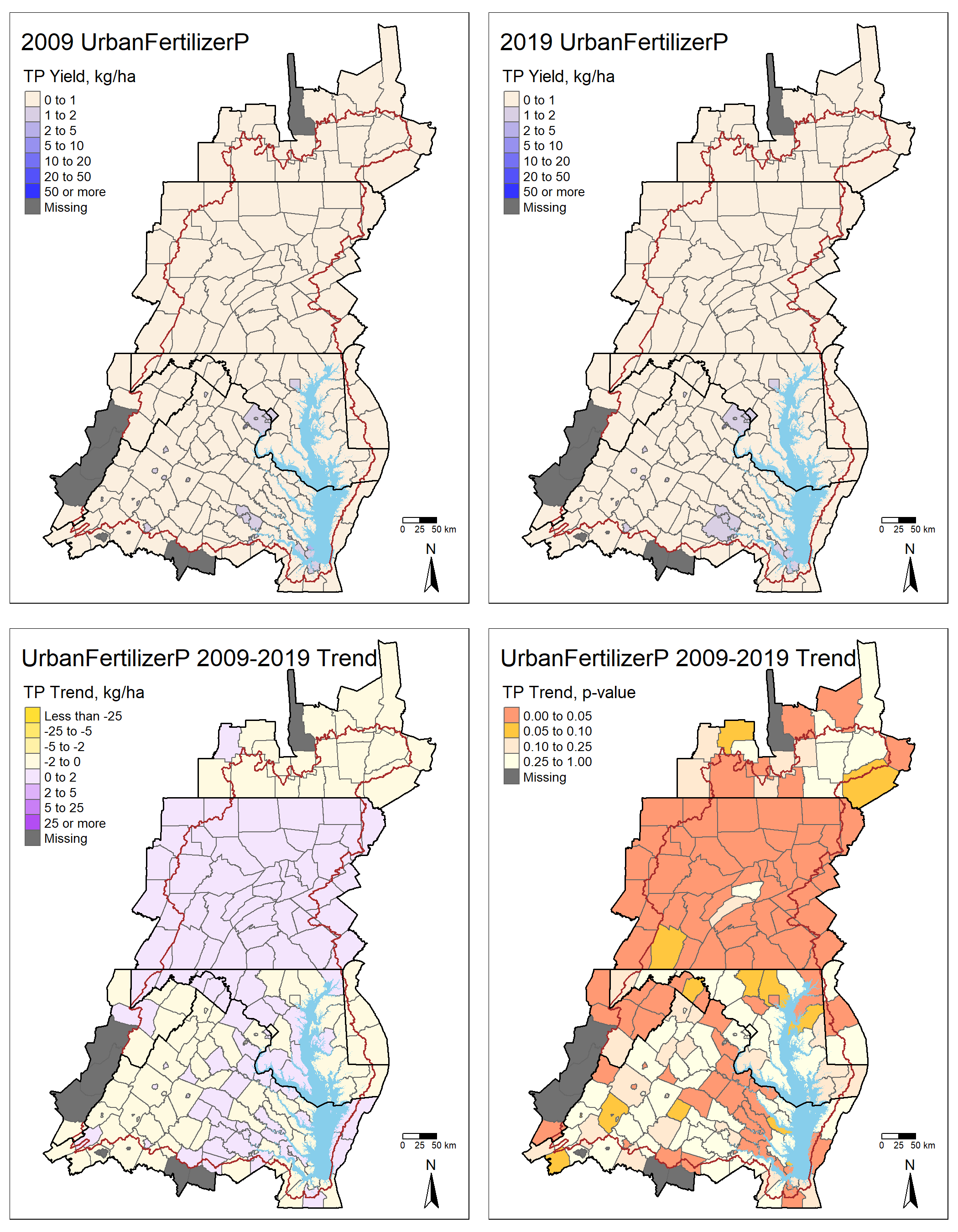
 Figure S100. For phosphorus, 2009 and 2019 urban fertilizer (top row), the estimated Sen linear slope change in urban fertilizer from 2009-2019 (bottom left), and the significance of trend results by county (bottom right). Figure S101. For nitrogen, 1985 and 2019 agricultural nutrient use efficiency (top row), the estimated Sen linear slope change in agricultural nutrient use efficiency from 1985-2019 (bottom left), and the significance of trend results by county (bottom right).

Figure S102. For nitrogen, 2009 and 2019 agricultural nutrient use efficiency (top row), the estimated Sen linear slope change in agricultural nutrient use efficiency from 2009-2019 (bottom left), and the significance of trend results by county (bottom right). Figure S103. For nitrogen, 1985 and 2019 agricultural nutrient use efficiency with pasture terms (top row), the estimated Sen linear slope change in agricultural nutrient use efficiency with pasture terms from 1985-2019 (bottom left), and the significance of trend results by county (bottom right). Figure S104. For nitrogen, 2009 and 2019 agricultural nutrient use efficiency with pasture terms (top row), the estimated Sen linear slope change in agricultural nutrient use efficiency with pasture terms from 2009-2019 (bottom left), and the significance of trend results by county (bottom right). Figure S105. For phosphorus, 1985 and 2019 agricultural nutrient use efficiency (top row), the estimated Sen linear slope change in agricultural nutrient use efficiency from 1985-2019 (bottom left), and the significance of trend results by county (bottom right). Figure S105. For phosphorus, 2009 and 2019 agricultural nutrient use efficiency (top row), the estimated Sen linear slope change in agricultural nutrient use efficiency from 2009-2019 (bottom left), and the significance of trend results by county (bottom right). Figure S107. For phosphorus, 1985 and 2019 agricultural nutrient use efficiency with pasture terms (top row), the estimated Sen linear slope change in agricultural nutrient use efficiency with pasture terms from 1985-2019 (bottom left), and the significance of trend results by county (bottom right).Figure S108. For phosphorus, 2009 and 2019 agricultural nutrient use efficiency with pasture terms (top row), the estimated Sen linear slope change in agricultural nutrient use efficiency with pasture terms from 2009-2019 (bottom left), and the significance of trend results by county (bottom right).

Figure S109. Time series of atmospheric N deposition as well as agricultural surplus and point source loads for nitrogen and phosphorus for the Chesapeake Bay. For phosphorus, negative surplus values indicate crop removal rates were greater than fertilizer and manure inputs in a given year (resulting in nutrient mining). County level estimates of soil phosphorus are available in the supplemental database, and the magnitude and long/short-term trends in these estimated pools are illustrated in Figures S85-S88.
